# Origin and evolution of lysyl oxidases

**DOI:** 10.1038/srep10568

**Published:** 2015-05-29

**Authors:** Xavier Grau-Bové, Iñaki Ruiz-Trillo, Fernando Rodriguez-Pascual

**Affiliations:** 1 Institut de Biologia Evolutiva (CSIC-Universitat Pompeu Fabra), Barcelona, Spain; 2 Centro de Biología Molecular “Severo Ochoa” Consejo Superior de Investigaciones Científicas (C.S.I.C.) / Universidad Autónoma de Madrid (Madrid), Madrid, Spain; 3 Departament de Genètica, Universitat de Barcelona, Barcelona, Spain; 4 Institució Catalana de Recerca i Estudis Avançats (ICREA), Barcelona, Spain

## Abstract

Lysyl oxidases (LOX) are copper-dependent enzymes that oxidize primary amine substrates to reactive aldehydes. The best-studied role of LOX enzymes is the remodeling of the extracellular matrix (ECM) in animals by cross-linking collagens and elastin, although intracellular functions have been reported as well. Five different LOX enzymes have been identified in mammals, LOX and LOX-like (LOXL) 1 to 4, showing a highly conserved catalytic carboxy terminal domain and more divergence in the rest of the sequence. Here we have surveyed a wide selection of genomes in order to infer the evolutionary history of LOX. We identified LOX proteins not only in animals, but also in many other eukaryotes, as well as in bacteria and archaea – which reveals a pre-metazoan origin for this gene family. LOX genes expanded during metazoan evolution resulting in two superfamilies, LOXL2/L3/L4 and LOX/L1/L5. Considering the current knowledge on the function of mammalian LOX isoforms in ECM remodeling, we propose that LOXL2/L3/L4 members might have preferentially been involved in making cross-linked collagen IV-based basement membrane, whereas the diversification of LOX/L1/L5 forms contributed to chordate/vertebrate-specific ECM innovations, such as elastin and fibronectin. Our work provides a novel view on the evolution of this family of enzymes.

Lysyl oxidases (LOX) are a family of copper-dependent amino oxidases for which important roles in cancer and vascular and fibrotic diseases have been proposed^1^. Five different LOX enzymes have been identified in mammals (LOX, and LOX-like 1 to 4), showing a high degree of homology in the catalytic carboxy terminal end and more divergence in the rest of the sequence^2^. While intracellular functions have been reported for LOX proteins, the primary role of this family of enzymes is the remodeling of the extracellular matrix (ECM), due to their capacity to convert lysine and hydroxylysine residues in collagens and elastin into highly reactive aldehydes, which eventually condense with other oxidized groups or intact lysines to form a variety of inter- and intrachain cross-linkages. The fundamental role of LOX proteins in ECM homeostasis has been demonstrated in experiments with mice lacking the LOX gene, which die just before or soon after birth by severe cardiovascular malformations, most likely involving defective elastogenesis^3^. Moreover, mice deficient in LOXL1, the closest mammal paralog of LOX, exhibit also cardiovascular defects, although they are perfectly viable and show a normal life span^4^. The remaining members (LOXL2-4) share the presence of four scavenger receptor cysteine-rich (SRCR) domains, a unique class of ancient, highly conserved polypeptide module present in a number of soluble and membrane-bound proteins for which no unifying function has been so far defined^5^. Recent work has described the capacity of LOXL2 and LOXL4 to enhance collagen IV deposition and assembly^6^,^7^. Nevertheless, it remains to be defined how this ECM remodeling capabilities fit together with the intracellular actions described for some of these SRCR-containing LOX members, such as the role of LOXL2 in the regulation of gene transcription^8^,^9^.

It is beyond doubt that the numerous evolutionary transitions from unicellular to multicellular organisms that occurred within eukaryotes could have never happened without their organization into extracellular structures. In contrast to sessile algae, fungi, and plants, which acquired a comparatively uniform composition in their cell walls, animals exhibit a complex and heterogeneous ECM, with multiple protein families involved in the construction of intricate structural networks, as well as many protein complexes devoted to intercellular adhesion and communication^10^. Recent genome data have revealed that some of the large, secreted, multidomain ECM components, including basement membrane-forming collagen IV and fibrillar collagens appear to be specific to the Metazoa^11^. Nevertheless, important domains from ECM proteins have a pre-metazoan origin. For instance, the filasterean *Capsaspora owczarzaki,* a close relative of Metazoa, has protein domains related to laminin and fibronectin, as well as a complete integrin adhesome^12^,^13^,^14^. Furthermore, choanoflagellates harbor many collagen motifs and domains otherwise specific to animals, such as the repeated GXY triple helical motif (even though these organisms lack fibrillar collagen)^15^. Domain shuffling of ancestral, premetazoan domains on the metazoan stem lineage have been proposed to give rise to the fibril-forming collagens, which are conserved throughout the metazoan evolutionary tree^16^,^17^. The same is true for collagen IV^18^,^19^. From these “founder genes”, rounds of gene duplication and domain or exon shuffling have resulted in the formation of different classes, comprising currently 28 collagen genes in vertebrates, which play structural roles in soft tissues or act as templates for biomineralisation in bone or teeth^17^,^20^. However, this family expansion has not been universal for all metazoans. For example, *Drosophila* lacks any fibrillar collagens that were most likely secondarily lost^21^. Remarkably, chordates and, specifically, vertebrates have witnessed a significant number of ECM innovations, including not only the duplication of pre-existing deuterostome genes but also the generation of complex forms of collagen (transmembrane collagens, FACIT collagens, among others) or of specific protein innovations^22^. In particular, elastin is one of the vertebrate-specific ECM novelties, and has played a fundamental role in the evolution of a high-pressure, pulsatile blood circulation system^23^.

Very limited information is available about the existence of LOX isoforms in non-bilaterian animals or other organisms. LOX-generated cross-links have been isolated from a sponge (*Haliclona oculata*), a sea urchin (*Strongylocentrotus droebachensis*), a sea cucumber (*Thyone briarius*), as well as from several annelids, echinodermates and molluscs^24^,^25^. Additionally, arthropodes like *Drosophila* have been reported to have two distinct LOX-like genes, whereas some chordates such as the cyprinidae *Danio rerio* (zebrafish) present up to 10 LOX genes^26^,^27^,^28^. A preliminary phylogenetic analysis of LOX genes revealed that human LOX and LOXL1 share a common ancestor and form an independent group from LOXL2, LOXL3 and LOXL4, being likely related to the *Ciona intestinalis* LOX1 and LOX2, respectively^22^. However, we lack an understanding of the evolutionary origin of the members of the LOX family, and how they relate to the evolution of the main ECM components such as collagens and elastin.

We here have surveyed a wide selection of genomes representing all the major eukaryotic and prokaryotic clades, aiming to reconstruct the evolutionary history of LOX enzymes. Our phylogenetic analyses, based on the conserved lysyl oxidase domain of LOX enzymes, show that LOX sequences are identifiable not only in animals, but also in many other eukaryotes, as well as in bacteria and archaea. This points at a much older origin than previously thought for LOX enzymes, preceding the origin of animals^21^. Our phylogenetic analyses show a significant expansion of LOX types during metazoan evolution, giving rise to three LOX families in Porifera (sponges) and two superfamilies in Eumetazoa (bilaterians and cnidarians). The LOXL2/L3/L4 superfamily is typically associated with SRCR domains, whereas LOX/L1/L5 display distinct N-terminal domains, and is related to the mammalian LOX and LOXL1. Based on the existing knowledge on the evolution of collagens and elastin, we propose here that LOXL2/L3/L4 members might contribute to the cross-linking of basement membrane collagen IV, whereas LOX/L1/L5 proteins may have evolved to cover the requirements of more sophisticated ECM in chordate/vertebrate phyla.

## Results

### The prokaryotic history of LOX enzymes

**Figure 1:**
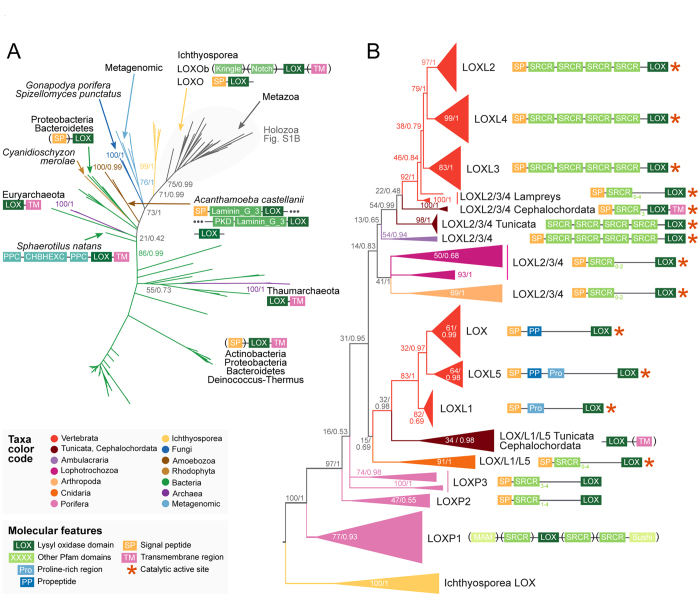
Phylogenetic trees of LOX enzymes in eukaryotes and prokaryotes. ** A**) Unrooted tree of 154 LOX domains from eukaryotic and prokaryotic genomes as inferred by bayesian inference. **B**) Rooted tree of 129 LOX domains from an expanded selection of holozoans (animals and their unicellular relatives, see grey-shadowed area of part A), as inferred by bayesian inference. Nodal support values are shown at key branches (Maximum likelihood bootstrap support/Bayesian posterior probabilities). Sequences are color-coded according to their taxonomic assignment. The consensus protein domain architectures of each LOX family are shown adjacent to each phylogeny, including Pfam domains (green boxes), proline-rich and propeptide regions (blue), transmembrane regions (pink), signal peptide motifs (orange) and the Interpro 019828 motif (red asterisk). The trees are not to scale. See supplementary Figures S1, S2, S3 and S4 for detailed versions of these phylogenies, including scaled branches and complete nodal support.

**Figure 2:**
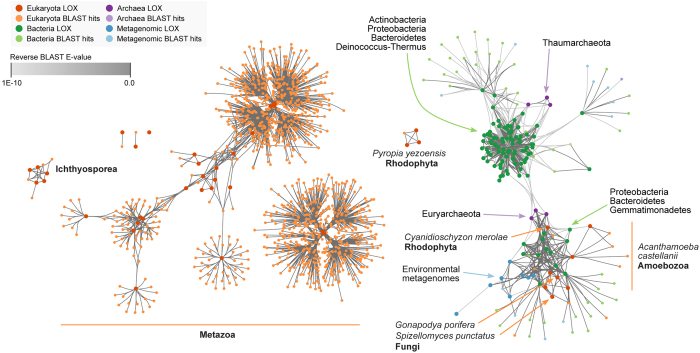
Network of reciprocal BLAST searches for LOX enzymes. Each node represents a LOX-containing protein. Nodes are connected by edges when they are reciprocal BLAST hits of each other (see Methods). Nodes are color-coded according to their taxonomic assignment (for some clusters of interest, further taxonomic details are also shown). Edges are color-coded according to the E-value of each BLAST hit.

Figure 1 shows phylogenetic analysis of LOX enzymes in prokaryotes and eukaryotes (panel A, unrooted tree) and Holozoa only (panel B, using ichthyosporean LOX as tree root). The network of reciprocal blast hits with indication of their score is shown in Fig. 2. Complete phylogenies are shown in Supplementary Files S1 to S4, sequences in Files S5 and S6.

Besides the eukaryotic LOX enzymes, our survey identifies for the first time LOX in both Archaea and Bacteria. In particular, LOX-coding genes are widely distributed in Bacteria, being present in five major clades: Bacteroidetes, Actinobacteria, Proteobacteria, Gemmatimonadetes and Deinococcus-Thermus (Fig. 1A). In contrast, the archaeal LOX homologs cluster into two separate groups of thaumarchaeotes and euryarchaeotes (Fig. 1A). In fact, each of these archaeal groups are associated to bacterial LOX and appear to be composed of sequences from phylogenetically close organisms (Supplementary Fig. S1 and S2). This suggests that thaumarchaeotes and euryarchaeotes could have acquired LOX through two independent horizontal gene transfer (HGT) events from bacteria (Figs. 1A and 2), although identification of the bacterial donors is required to confirm this hypothesis.

Finally, it is interesting to note that, in contrast to eukaryotic LOX, most (except three) of the identified prokaryotic sequences exhibit simple protein domain architectures with just the LOX domain, with or without signal peptide and/or transmembrane region.

### LOX in unicellular eukaryotes

Our data also show the presence of LOX enzymes in different eukaryotic non-metazoan lineages (Fig. 1). Specifically, we identified LOX genes in the genomes of some Amorphea/Unikonta taxa (including animals, fungi and a number of unicellular clades), as well as from the Rhodophyta (red algae, from the Diaphoratickes supergroup).

The phylogenetic analysis of LOX recovers a major clade that includes opisthokont LOX homologs (all known animal enzymes, fungi and ichthyosporeans) together with a number of environmental metagenomic sequences (Fig. 1; BS 73%, BPP 0.99). Within fungi, we identify LOX homologs in the chytrid *Spizellomyces punctatus* and the monoblepharidomycete *Gonapodya prolifera*. Ichthyosporeans, which are a group of unicellular organisms closely related to animals^29^, have also the most animal-like LOX genes according to our phylogeny (Fig. 1). They have two sets of LOX, one of which (LOXOb) has acquired C-terminal Kringle, PLAT and Notch protein domains (Fig. 1A). While the function of LOX in ichthyosporeans is at present unknown, the occurrence of the transmembrane region of Notch suggests some membrane-associated role akin to the SRCR-containing LOX of animals.

We also identified LOX homologs in the unicellular amoebozoan *Acanthamoeba castellanii* and the rhodophytes *Cyanidioschyzon merolae* (unicellular algae) and *Pyropia yezoensis* (multicellular seaweed). However, they could not be unambiguously classified to any specific group, probably due to either low statistical support (*A. castellanii* and *C. merolae*) or insufficient data (*P. yezoensis*). According to the network of reciprocal BLAST (Fig. 2), the *C. merolae* LOX and the 4 copies of *A. castellanii* (BS 98%, BPP 0.99) seem to be related to prokaryotic, environmental or fungal sequences, whereas *P. yezoensis’* proteins cluster separately from the rest of the known LOX enzymes.

It is interesting to note that neither *A. castellanii* nor fungi have collagen-based ECM structures equivalent to those of animals. As for the multicellular seaweeds, they do have complex polysaccharide-based ECM, but do not possess collagen-based structures.

### LOX diversification in animals

It is within animals where we found the greatest variety of LOX forms, with many duplications and frequent rearrangements of protein domain architectures (Fig. 1B).

We identified three groups of LOX enzymes specific to Porifera (sponges), termed LOXP1-3 (pink branches in Fig. 1B). Each of them has different protein domain architectures based on transmembrane SRCR domains, both N- and C-terminal. The LOXP1 family is only present in calcareous sponges (*Sycon ciliatum* and *Leucosolenia complicata*) and contains proteins with multiple domains, including not only SRCR but also MAM or Sushi. Given that LOXP1 is the earliest family present in animals, this means that the association between LOX and SRCR domains was already present at the origin of animals. LOXP2 and P3 families, both with the canonical N-terminal SRCR repeats, are present in demosponges (*Amphimedon queenslandica*), homoscleromorph (*Oscarella carmela*) and calcareous sponges.

A duplication event at the origin of eumetazoans gave birth to two animal LOX superfamilies that although not statistically supported, are recovered by both Maximum Likelihood and Bayesian inference analyses: LOX/L1/L5 (composed of homologs of human canonical LOX and LOXL1, plus the fish-specific LOXL5) and LOXL2/L3/L4 (homologs of human LOXL2, LOXL3 and LOXL4).

**Figure 3:**
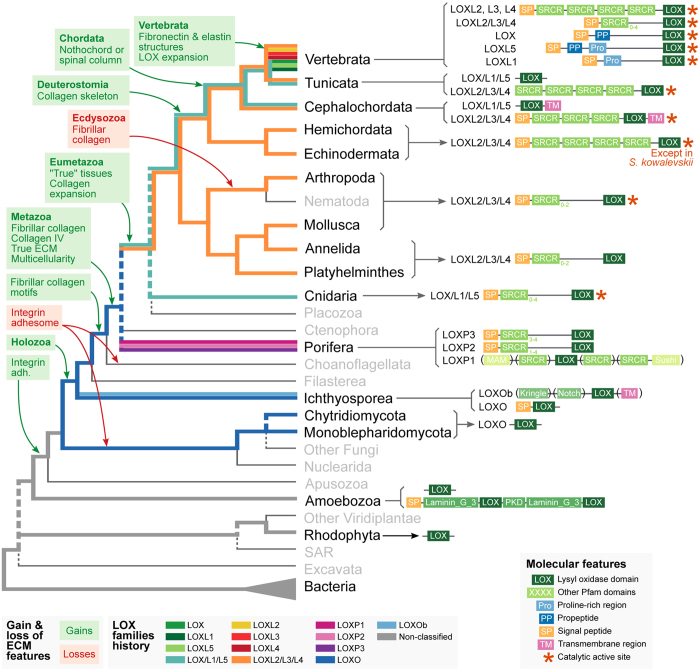
Reconstruction of the evolutionary history of LOX enzymes and ECM across the tree of life. The cladogram represents a consensus view of the eukaryotic tree of life (see Methods) with bacteria as outgroup. Each bold, colored line represents a LOX family (as indicated in the legend); its route along the tree represents their pattern of appearance and loss in each taxonomic group. Dashed lines represent unclear phylogenetic relationships. Green- and red-colored boxes represent gains and losses of ECM features, respectively. The consensus protein domain architectures of each LOX family are shown adjacent to each taxonomic group, including Pfam domains (green boxes), proline-rich and propeptide regions (blue), transmembrane regions (pink), signal peptide motifs (orange) and the Interpro 019828 motif (red asterisk).

The LOX/L1/L5 superfamily (BS 15%, BPP 0.69) is present in cnidarians (dark orange branch in Fig. 1B), that have the ancestral SRCR-containing form, and chordates (red and dark red branches), that lack SRCR domains (Fig. 1B, see also a cladogram with domain gain/loss in Fig. 3). At the origin of vertebrates, this superfamily gives rise to the LOX, LOXL1 and LOXL5 (exclusive to various fish clades) gene families. LOXL1 enzymes have a N-terminal proline-rich region, also conserved in LOXL5 but lost in canonical LOX. Canonical LOX and LOXL5, in turn, share an exclusive propeptide region (Fig. 1B).

The LOXL2/L3/L4 superfamily (BS 14%, BPP 0.83) was lost in cnidarians and is only present in bilaterian genomes (Figs. 1B and 3). All the families retain the ancestral SRCR-containing form, with variations in the number of repeats (Fig. 1B). This is the only LOX family present in protostomes (arthropods, molluscs, annelids and platyhelminths) and ambulacrarian deuterostomes (hemichordates and echinoderms). It is also present in tunicates and cephalochordates. The vertebrate-specific LOXL2, LOXL3 and LOXL4 families originated after the divergence of *Petromyzon marinus* (sea lamprey), which retains the ancestral type. All of them have four N-terminal SRCR repeats.

Overall, vertebrates have the highest count of LOX enzyme types among eukaryotes, with five widespread families (canonical LOX, LOXL1, LOXL2, LOXL3 and LOXL4), one family specific to fishes (LOXL5, found in actinopterygian, sarcopterygian and cartilaginous fishes) and one specific to lampreys (LOXL2/L3/L4). These LOX types display five different protein domain architectures (Fig. 1B).

We could not identify any LOX gene in nematodes, nor in the placozoan *Trichoplax adhaerens* or the ctenophore *Mnemiopsis leidyi*.

### Assessment of the catalytic activity of novel LOX homologs

**Figure 4:**
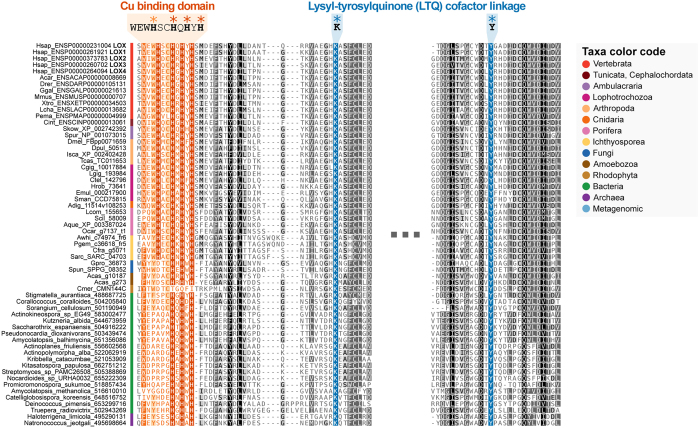
Multiple sequence alignment of catalytic LOX domains. 60 LOX proteins representing all of the groups analyzed in our study were aligned in order to inspect for conserved residues involved in the catalysis. Conserved residues highlighted in red constitute the cores of histidines forming the copper binding site within the InterPro 019828 motif (Lysyl oxidase). Note that the histidine depicted in orange within this motif is conserved in animals and ichthyosporeans, but not present in bacterial, fungal or amebozoan sequences. Strictly conserved lysine and tyrosine residues involved in LTQ cofactor linkage are highlighted in blue.

The presence of LOX domains in previously unreported eukaryotes and prokaryotes raises the question of whether they are enzymatically active proteins or not. It has been demonstrated that LOX catalytic activity relies on the C-terminal domain of the protein, where two features are needed. First, the core of histidines forming the copper binding site, the so-called “copper-talon”, which matches the conserved motif Interpro 019828 (WEW**H**SC**H**Q**H**Y**H**SMD in human LOX, Hsap_ENSP00000231004)^30^. Second, the lysine and tyrosine residues involved in the association with the lysyl tyrosyl quinone (LTQ) cofactor (K320 and Y355 in Hsap_ENSP00000231004)^31^. These key amino acids are widely conserved in all the groups analyzed in our study (Fig. 4, see also Supplementary Files S7 and S8) with the exception of the rhodophyte *C. merolae*, which lacks the histidine core. This observation predicts that these LOX homologs can be enzymatically competent to oxidize substrates. Interestingly, the first histidine residue within the copper binding site (H289 in Hsap_ENSP00000231004) is conserved in animals and ichthyosporeans, but is not present in bacterial, fungal or amebozoan sequences. Recent experimental evidence have provided useful information about whether the loss of this histidine residue can compromise the binding of copper, and therefore, the catalytic activity^32^. These authors sequentially mutated the histidine into alanines (being incapable to bind copper), and showed that the substitution of the first histidine did not significantly alter the ability of the enzyme to bind copper and oxidize substrates. Based on this report, it can be predicted that LOX domains identified in our work would display catalytic activity as they possess the core of the three essential histidines and the residues implicated in the LTQ linkage.

## Discussion

Our results provide the most comprehensive up-to-date phylogenetic analysis of the family of LOX enzymes. A main conclusion is that the LOX domains are more widely distributed than previously thought, as we identify clear homologs in animals and other eukaryotes, as well as bacteria and archaea^22^.

Based on our phylogenetic analyses with a wide taxon sampling, we can reconstruct the evolution and diversification of LOX enzyme families in eukaryotes and prokaryotes. With respect to the eukaryotic LOX enzymes, we identify a group of ichthyosporean and fungal LOX homologs as the closest relatives to the known animal enzymes (Fig. 1). This clearly indicates that this amino oxidase enzyme family was already present in the opisthokont ancestor, thus predating the origin of metazoans. Different scenarios could explain the origin of this opisthokont LOX according to our results. First, it could have been derived from an ancestral eukaryotic homolog from which the *A. castellanii* and *C. merolae* copies could have derived as well. Second, it could have been acquired by a horizontal gene transfer (HGT) event from bacteria to an ancestral opisthokont.

In order to understand the evolutionary history of LOX enzymes outside opisthokonts, we need to understand how LOX enzymes first appeared (in eukaryotes or prokaryotes) and whether HGT events took place (and when). However, the distribution of LOX cannot be conclusively explained by our phylogeny, as several non-exclusive scenarios would fit. For example, a potential explanation would be a bacterial origin of LOX, followed by a later transfer to eukaryotes (either by HGT or during the process of eukaryogenesis) and multiple secondary losses. Another possibility would be a later eukaryotic origin followed by a number of HGT events between eukaryotes and prokaryotes, and within prokaryotes as well.

In support of the HGT-driven scenarios, the genomes of *A. castellanii* and *C. merolae* are both known to have experienced multiple HGTs from bacteria, and the same is true for amoebozoan genes being transferred to prokaryotes^33^,^34^,^35^ It is worth noting that HGT of metabolic genes from prokaryotes is an important factor underlying the diversification of eukaryotes, particularly in the case of amoebas such as *A. castellanii* or a hypothetical amorphean ancestor^12^,^33^,^36^. If this were the case, the acquisition of LOX by an ancestral microbial eukaryote would have had an important, delayed effect in the evolution of the ECM, as it eased the appearance of the current enzyme types essential for its formation.

The presence of LOX enzymes in bacteria raises the question of the function of LOX within these organisms. Several collagen-like proteins have been identified in bacteria, and for some of them, the formation of a stable triple helix has been demonstrated^21^,^37^. Some of the best characterized bacterial collagen-like proteins are the streptococcal Scl1 and Scl2, which are expressed on the cell surface of group A *Streptococcus* and contribute to bacterial pathogenicity through the binding to host ECM components including integrins and fibronectin^38^,^39^. Our analysis did not identify LOX isoforms in members of the *Streptococcus* genus, but, for example, in a number of *Streptomyces* species, for which collagen-like sequences have also been genome-annotated (see, for instance, Uniprot entries: D9WI30 or D6B4A5, www.uniprot.org). Nevertheless, a higher order structure reminiscent of intra- or interchain covalent association has not yet been described for bacterial collagen-like proteins, therefore making unlikely that LOX may cross-link bacterial collagenous material. While more studies are needed to elucidate the function of bacterial LOX enzymes, it can be hypothesized that LOX proteins may be a component of the enzymatic repertoire of bacterial metabolism transferred to eukaryotes and adapted to new functions, as suggested to have occurred, for instance, with the epigenetic machinery^40^. Interestingly, collagen-like proteins present in bacteria have also been proposed to originate from an HGT event from metazoans to bacteria^41^.

Current views of the evolution of the animal ECM envision its constitution as the result of a gradual appearance of specific gene families and domains in pre-metazoan lineages, followed by remarkable expansions in animals. This is best exemplified by the presence of a fully functional integrin adhesome in *C. owczarzaki*, a unicellular filasterean with aggregative behavior that also has proteins with laminin and fibronectin motifs (although with different domain architectures than their animal counterparts)^12^,^13^,^14^,^42^,^43^. This is also the case of the choanoflagellates *Monosiga brevicollis* and *Salpingoeca rosetta*, that have proteins with collagen and laminin domains (also without a clear homologs in animals)^14^,^15^,^44^. Further refinement of these pre-existing protein families and the appearance of Metazoa-specific innovations provided the chordates and vertebrates with a wider repertoire of ECM proteins to fulfill novel functions in the vasculature or in the nervous system^18^.

Our phylogenetic analysis of LOX revealed a relatively similar pattern of evolution: LOX domains were already present in unicellular eukaryotes (notably in the ichthyosporeans, that are closely related to Metazoa), and further expanded during metazoan evolution. Interestingly, unicellular organisms such as the ichthyosporeans *Sphaeroforma arctica*, *Creolimax fragrantissima*, *Pirum gemmata* and *Abeoforma whisleri* or the amoebozoan *Acanthamoeba castellanii*, display forms of LOX associated with domains thought to serve extracellular protein-protein interactions, for example PKD, Kringle or PLAT (with or without the presence of transmembrane regions), much in the same role that SRCR has been postulated to play in SRCR-containing LOX forms^2^.

According to our study, SRCR domains first associated with LOX proteins in Metazoa, specifically in sponges (see Fig. 3). The SRCR domains in sponges are present both at N- and C-terminal, with and without association with other protein architectures, such as MAM or Sushi. Adult sponges consist of two layers of cells with epithelial features supported by a central cavity, the mesohyl, consisting of rigid material. Fibrillar and basement membrane collagens have been identified in the mesohyl and in the lamina were the two layers of cells attach, respectively^45^,^46^. Therefore, sponges constitute the first class of organisms where LOX enzymatic activities might have begun to sculpt the ECM. Whether LOX may have provided Porifera with novel capabilities such as spicule biomineralization or body stiffening required for efficient water flow is at present unknown. It is worth mentioning that neither Ctenophora nor Placozoa have LOX genes. The origin of the eumetazoans witnessed the main branching of LOX isoforms, giving place to the LOXL2/L3/L4 and LOX/L1/L5 superfamilies (Fig. 1B and Fig. 3). The former kept the SRCR-LOX architecture invariably from arthropods to vertebrates, with minimal variations in the number of SRCR domains. The observation that this class of LOX is present in arthropods such as *Drosophila melanogaster*, which lacks fibrillar collagen, suggests that these LOX isoforms might preferentially (but not exclusively) cross-link basement membrane collagen IV, and thereby controlling ECM stiffness, as recently described^26^,^47^. In fact, collagen IV-cross linking activities for mammalian LOXL2 and LOXL4 have recently been reported^6^,^7^. Nevertheless, intracellular functions beyond matrix cross-linking have been also reported for LOXL enzymes, for instance transcriptional regulation or control of cell cycle and apoptosis for LOXL2^8^,^9^.

In contrast to LOXL2/L3/L4, the LOX/L1/L5 superfamily experienced significant changes in domain architecture during evolution. While forms present in cnidarians retain SRCR domains, LOX/L1/L5 from tunicates and cepholochordates show no recognizable associated domains, and chordates and vertebrates display forms with propeptide and proline-rich regions typical of mammalian LOX and LOXL1 (Fig. 1B). As shown in Fig. 3, the appearance of LOX isoforms with these domain architectures is coincident with a significant expansion of vertebrate-specific ECM innovations, a circumstance reinforcing their widely accepted role as catalyzers of lysine-derived cross-links in fibrillar collagens and elastin. To this respect, LOX and LOXL1 have been reported to interact with tropoelastin through sequences in the N-terminal pro-regions^48^. Although the specific motifs within the pro-regions of LOX and LOXL1 that drive the association with elastin are not known, significant homology exists at the N-terminal sequence to support this interaction. Additionally, strong binding has been reported between LOX and fibulin-4 and LOXL1 and fibulin-5^4^,^49^. Fibulin-4 and -5 are essential proteins for the assembly of elastic fibers, and their interaction with LOX isoforms seems to facilitate the cross-linking of tropoelastin within elastic fibers^50^. Based on these observations, it can be inferred that LOX and LOXL1 forms evolved to contribute to elastogenesis, an assumption further reinforced by the result of the inactivation of these genes in mouse models, both giving rise to vascular phenotypes due to impaired elastic fiber formation^3^,^4^.

It is interesting to mention that LOX and LOXL1 are proteolytically processed by bone morphogenic protein 1 (BMP1)/Tolloid-like metalloproteinases^51^,^52^,^53^,^54^. First identified as pro-collagen C-proteinases, this family of proteolytic enzymes has been described to cleave a wide repertoire of substrates^55^. It is worth mentioning that, with the exception of apolipoprotein 1 and gliomedin, which play unique roles in lipid metabolism and peripheral nervous system, respectively, BMP1 substrates belong to the category of ECM proteins or ECM-related factors, including fibrillar procollagens, small leucine-rich proteoglycans, basement membrane components, and mineralization factors, among many others^55^. The fact that LOX and LOXL1 forms are also cleaved by BMP1-related proteases suggests that the primary function of these LOX forms is matrix-oriented. LOX and LOXL1 needs to be processed to yield the catalytically active forms. Therefore, it is conceivable to propose that the proteolysis step serves as a quality control step to keep the LOX enzyme in a latent state until the proper substrate is encountered.

Another important vertebrate ECM innovation is fibronectin, an adhesive protein involved in many cellular responses with a significant role in wound healing^56^. In this context, the formation of a fibronectin matrix is critical for the subsequent assembly of types I and III collagen fibrils. The canonical LOX has been reported to interact with fibronectin through sequences both in the pro-region and in the C-terminal^57^. In fact, fibronectin may also contribute to the processing of the pro-enzyme, as fibronectin scaffolds support BMP1 binding through periostin^58^,^59^. Taken together, these evidences point out to a significant role for LOX and LOXL1, through their associated domains, in chordate/vertebrate-specific ECM building, particularly in the circulatory system and during tissue repair. Within these functions, it is interesting to note that LOXL5, present in early-branching vertebrate clades of fishes (Actinopterygii, Chondrichthyes and Sarcopterygii), contains both the proline-rich and propeptide regions. Thus, fishes retain both functionalities in the same enzyme, whereas its sister LOX family, present in the other vertebrates, has lost the proline-rich region. This probably reflects the specialization of the canonical LOX in particular functions in non-fish vertebrates.

In conclusion, our phylogenetic analysis of LOX proteins permits to trace the evolution of this family of enzymes, particularly in the context of the acquisition of the ECM components, collagen and elastin. Fig. 3 illustrates the appearance of LOX proteins within the elaboration of ECM components during eukaryotic evolution. Remarkable events include: 1) the presence of LOX forms in unicellular eukaryotes, associated to several domain architectures presumably serving extracellular protein-protein interactions; 2) the acquisition of SRCR domains as a specific feature of animals, presumably coincident with the appearance of true ECM in early metazoans; and 3) the generation of chordate/vertebrate LOX forms possibly supporting novel ECM innovations such as elastin and fibronectin.

## Methods

### Taxon sampling and sequence retrieval

LOX sequences were queried in complete genome or transcriptome sequences of 117 eukaryotic taxa representing all known eukaryotic supergroups, as well as all the major metazoan clades. Taxon sampling includes 37 metazoans, 10 unicellular holozoans, 24 fungi, 2 nucleariids, 1 apusozoan, 4 amoebozoans, 7 plants, 5 chlorophytes, 3 rhodophytes, 1 glaucophyte, 8 heterokonts, 6 alveolates, 1 rhizarian, 1 haptophyte, 1 cryptophyte and 6 excavates (Supplementary Tables S1 and S2, list of sequences in Files S5 and S6). Prokaryotic sequences were queried in the NCBI non-redundant database and the Microbial Dark Matter Project database^60^. The proteins with LOX domains were retrieved from the complete proteomes with HMMER^61^, using a Hidden Markov motif of the LOX domain as defined by Pfam (PF01186)^62^. These proteins were inspected using Pfamscan and manual alignments to assess the presence of protein domains including those found in mammalian LOX, such as the proline-rich and pro-peptide motifs, or scavenger receptor cysteine-rich domains^62^.

### Phylogenetic inference

The LOX domains (PF01186) of the retrieved sequences were aligned using the Mafft 7 L-INS-i algorithm, optimized for local sequence homology^63^. Two alignments were produced: 1) one containing eukaryotic, bacterial and archaeal proteins (154 sequences, 217 alignment positions; using eukaryotes from Supplementary Table S1); and 2) another one with just animal and ichthyosporean proteins (129 sequences, 283 aligned positions; using animals from Supplementary Table S2). According to ProtTest 3.4 analyses of each alignment^64^, the most suitable evolutionary models were WAG+Γ+F and LG+Γ+I, respectively (“Γ” stands for a gamma distribution of among-site rate variation with 4 discrete categories; “I” means that a proportion of invariable sites is considered; and “F” means that empirical amino acid frequencies are inferred from the alignment). The phylogenetic trees of each of these alignments were inferred using the corresponding model of evolution, with two independent methods: Maximum Likelihood (ML) and Bayesian Inference (BI). ML trees were estimated with RAxML 8, starting from 100 random trees and selecting the best inference according to the Γ-based likelihood value^65^. Statistical support for bipartitions was estimated by performing 100 bootstrap replicates, using RaxML with the same evolutionary models. BI trees were estimated with Phylobayes 3.3^66^ (which does not account for empirical amino acid frequencies nor invariable sites), running two parallel chains for each alignment. To decide when to stop the runs, we regularly performed a series of bpcomp tests on each pair of chains every 5,000 generations, consisting in burning-in the tree lists every 1% of the generations run so far. The final trees were built using the number of generations and burn-in values that yielded the lowest maxdiff statistics, sampling every 10 trees (provided it was under the 0.1 threshold recommended by Phylobayes). This resulted in 30,000 generations and 5% of burning for the animal and ichthyosporean alignment, and 60,000 and 7% for the eukaryotic and prokaryotic alignment. Bayesian posterior probabilities (BPP) were used for assessing the statistical support of each bipartition. Using these phylogenetic trees, the evolution of LOX enzymes across eukaryotes and prokaryotes was reconstructed, based on a consensus tree of life drawn from different studies^67^,^68^,^69^.

### Annotation of molecular features

The protein domain architectures of the retrieved sequences were analyzed using Pfamscan^70^. The full proteins were also analyzed with SignalIP 4.1^71^ and TMHMM 2.0^72^ to search for signal peptide cleavage sites and transmembrane helical domains, respectively (default parameters in both cases). To assess whether the identified LOX domains can have catalytic activity, the InterPro IPR019828 conserved site was searched^73^. Proline-rich and propeptide regions were manually checked in the alignments. Annotations of molecular features are provided in Supplementary Files S7 and S8.

### Assessment of horizontal gene transfers

In addition to the information provided by phylogenetic inference, the possibility of horizontal gene transfer (HGT) events between taxa was tested using a reciprocal BLAST approach. Two sequences were considered to be connected if they were reciprocal BLAST hits of each other with an e-value <10^10^, when queried against a combined database consisting of the full NCBI non-redundant protein database, the Microbial Dark Matter database and our selected eukaryotic taxon sampling (see above). The network visualizations of the reciprocal BLAST hits were generated using Cytoscape 3.1.1, clustering the nodes using the built-in force-directed algorithm^74^.

## Additional Information

**How to cite this article**: Grau-Bové, X. *et al.* Origin and evolution of lysyl oxidases. *Sci. Rep.* **5**, 10568; doi: 10.1038/srep10568 (2015).

## Supplementary Material

Supporting Information

File S5

File S6

File S7

File S8

## References

[b1] Maki J. M. Lysyl oxidases in mammalian development and certain pathological conditions. Histol Histopathol 24, 651–660 (2009). 19283672 10.14670/HH-24.651

[b2] Csiszar K. Lysyl oxidases: a novel multifunctional amine oxidase family. Progress in nucleic acid research and molecular biology 70, 1–32 (2001). 11642359 10.1016/s0079-6603(01)70012-8

[b3] Maki J. M. *et al.* Inactivation of the lysyl oxidase gene Lox leads to aortic aneurysms, cardiovascular dysfunction, and perinatal death in mice. Circulation 106, 2503–2509 (2002). 12417550 10.1161/01.cir.0000038109.84500.1e

[b4] Liu X. *et al.* Elastic fiber homeostasis requires lysyl oxidase-like 1 protein. Nat Genet 36, 178–182 (2004). 14745449 10.1038/ng1297

[b5] Martinez V. G., Moestrup S. K., Holmskov U., Mollenhauer J. & Lozano F. The conserved scavenger receptor cysteine-rich superfamily in therapy and diagnosis. Pharmacol Rev 63, 967–1000 (2011). 21880988 10.1124/pr.111.004523

[b6] Bignon M. *et al.* Lysyl oxidase-like protein-2 regulates sprouting angiogenesis and type IV collagen assembly in the endothelial basement membrane. Blood 118, 3979–3989 (2011). 21835952 10.1182/blood-2010-10-313296

[b7] Busnadiego O. *et al.* LOXL4 is induced by TGF-beta1 through Smad and JunB/Fra2 and contributes to vascular matrix remodeling. Mol Cell Biol 33, 2388–2401 (2013). 23572561 10.1128/MCB.00036-13 PMC3700097

[b8] Herranz N. *et al.* Lysyl oxidase-like 2 deaminates lysine 4 in histone H3. Molecular cell 46, 369–376 (2012). 22483618 10.1016/j.molcel.2012.03.002

[b9] Moreno-Bueno G. *et al.* Lysyl oxidase-like 2 (LOXL2), a new regulator of cell polarity required for metastatic dissemination of basal-like breast carcinomas. EMBO molecular medicine 3, 528–544 (2011). 21732535 10.1002/emmm.201100156 PMC3377095

[b10] Engel J. & Chiquet M. in The Extracellular Matrix: an Overview (ed Mecham RP ) 1–39 (Springer-Verlag, 2011).

[b11] Ozbek S., Balasubramanian P. G., Chiquet-Ehrismann R., Tucker R. P. & Adams J. C. The evolution of extracellular matrix. Mol Biol Cell 21, 4300–4305 (2010). 21160071 10.1091/mbc.E10-03-0251 PMC3002383

[b12] Sebe-Pedros A., Roger A. J., Lang F. B., King N. & Ruiz-Trillo I. Ancient origin of the integrin-mediated adhesion and signaling machinery. Proceedings of the National Academy of Sciences of the United States of America 107, 10142–10147 (2010). 20479219 10.1073/pnas.1002257107 PMC2890464

[b13] Suga H. *et al.* The Capsaspora genome reveals a complex unicellular prehistory of animals. Nat Commun 4 (2013). 10.1038/ncomms3325 PMC3753549 23942320

[b14] Williams F., Tew H. A., Paul C. E. & Adams J. C. The predicted secretomes of Monosiga brevicollis and Capsaspora owczarzaki, close unicellular relatives of metazoans, reveal new insights into the evolution of the metazoan extracellular matrix. Matrix Biol 37, 60–68 (2014). 24561726 10.1016/j.matbio.2014.02.002

[b15] King N. *et al.* The genome of the choanoflagellate Monosiga brevicollis and the origin of metazoans. Nature 451, 783–788 (2008). 18273011 10.1038/nature06617 PMC2562698

[b16] Exposito J. Y. *et al.* Demosponge and sea anemone fibrillar collagen diversity reveals the early emergence of A/C clades and the maintenance of the modular structure of type V/XI collagens from sponge to human. J Biol Chem 283, 28226–28235 (2008). 18697744 10.1074/jbc.M804573200 PMC2661393

[b17] Exposito J.-Y., Valcourt U., Cluzel C. & Lethias C. The Fibrillar Collagen Family. Int J Mol Sci 11, 407–426 (2010). 20386646 10.3390/ijms11020407 PMC2852846

[b18] Hynes R. O. The evolution of metazoan extracellular matrix. The Journal of Cell Biology 196, 671–679 (2012). 22431747 10.1083/jcb.201109041 PMC3308698

[b19] Srivastava M. *et al.* The Trichoplax genome and the nature of placozoans. Nature 454, 955–960 (2008). 18719581 10.1038/nature07191

[b20] Ivanova V. P. & Krivchenko A. I. A current viewpoint on structure and evolution of collagens. I. Fibrillar collagens. J Evol Biochem Phys 48, 127–139 (2012). 22645972

[b21] Exposito J.-Y. & Lethias C. in Evolution of Extracellular Matrix Biology of Extracellular Matrix (eds Keeley Fred W. & Mecham Robert P. ) Ch. 3, 39–72 (Springer: Berlin Heidelberg, , 2013).

[b22] Huxley-Jones J., Robertson D. L. & Boot-Handford R. P. On the origins of the extracellular matrix in vertebrates. Matrix Biol 26, 2–11 (2007). 17055232 10.1016/j.matbio.2006.09.008

[b23] Wagenseil J. E. & Mecham R. P. Vascular extracellular matrix and arterial mechanics. Physiological reviews 89, 957–989 (2009). 19584318 10.1152/physrev.00041.2008 PMC2775470

[b24] Eyre D. R. & Glimcher M. J. Comparative biochemistry of collagen crosslinks: Reducible bonds in invertebrate collagens. Biochimica et Biophysica Acta (BBA) - Protein Structure 243, 525–529 (1971). 10.1016/0005-2795(71)90027-4 5129593

[b25] Van Ness K. P., Koob T. J. & Eyre D. R. Collagen cross-linking: distribution of hydroxypyridinium cross-links among invertebrate phyla and tissues. Comparative biochemistry and physiology. B, Comparative biochemistry 91, 531–534 (1988). 10.1016/0305-0491(88)90017-x 3233929

[b26] Molnar J. *et al.* Drosophila lysyl oxidases Dmloxl-1 and Dmloxl-2 are differentially expressed and the active DmLOXL-1 influences gene expression and development. J Biol Chem 280, 22977–22985 (2005). 15811848 10.1074/jbc.M503006200

[b27] Gansner J. M., Mendelsohn B. A., Hultman K. A., Johnson S. L. & Gitlin J. D. Essential role of lysyl oxidases in notochord development. Developmental biology 307, 202–213 (2007). 17543297 10.1016/j.ydbio.2007.04.029 PMC2467443

[b28] van Boxtel A. L. Lysyl oxidases in zebrafish development and teratogenesis, VU University Amsterdam, (2010).

[b29] Torruella G. *et al.* Phylogenetic relationships within the Opisthokonta based on phylogenomic analyses of conserved single-copy protein domains. Mol Biol Evol 29, 531–544 (2012). 21771718 10.1093/molbev/msr185 PMC3350318

[b30] Krebs C. J. & Krawetz S. A. Lysyl oxidase copper-talon complex: a model. Biochim Biophys Acta 1202, 7–12 (1993). 8104038 10.1016/0167-4838(93)90056-w

[b31] Wang S. X. *et al.* A crosslinked cofactor in lysyl oxidase: redox function for amino acid side chains. Science 273, 1078–1084 (1996). 8688089 10.1126/science.273.5278.1078

[b32] Lopez K. M. & Greenaway F. T. Identification of the copper-binding ligands of lysyl oxidase. Journal of neural transmission 118, 1101–1109 (2011). 21190048 10.1007/s00702-010-0559-4

[b33] Clarke M. *et al.* Genome of Acanthamoeba castellanii highlights extensive lateral gene transfer and early evolution of tyrosine kinase signaling. Genome biology 14, R11 (2013). 23375108 10.1186/gb-2013-14-2-r11 PMC4053784

[b34] Ogata H. *et al.* Genome sequence of Rickettsia bellii illuminates the role of amoebae in gene exchanges between intracellular pathogens. PLoS genetics 2, e76 (2006). 16703114 10.1371/journal.pgen.0020076 PMC1458961

[b35] Schmitz-Esser S. *et al.* The genome of the amoeba symbiont “Candidatus Amoebophilus asiaticus” reveals common mechanisms for host cell interaction among amoeba-associated bacteria. Journal of bacteriology 192, 1045–1057 (2010). 20023027 10.1128/JB.01379-09 PMC2812958

[b36] Andersson J. O. Gene transfer and diversification of microbial eukaryotes. Annual review of microbiology 63, 177–193 (2009). 10.1146/annurev.micro.091208.073203 19575565

[b37] Xu Y., Keene D. R., Bujnicki J. M., Höök M. & Lukomski S. Streptococcal Scl1 and Scl2 Proteins Form Collagen-like Triple Helices. Journal of Biological Chemistry 277, 27312–27318 (2002). 11976327 10.1074/jbc.M201163200

[b38] Rasmussen M., Edén A. & Björck L. SclA, a Novel Collagen-Like Surface Protein ofStreptococcus pyogenes. Infection and Immunity 68, 6370–6377 (2000). 11035747 10.1128/iai.68.11.6370-6377.2000 PMC97721

[b39] Lukomski S. *et al.* Identification and Characterization of thescl Gene Encoding a Group A StreptococcusExtracellular Protein Virulence Factor with Similarity to Human Collagen. Infection and Immunity 68, 6542–6553 (2000). 11083763 10.1128/iai.68.12.6542-6553.2000 PMC97748

[b40] Aravind L., Burroughs A. M., Zhang D. & Iyer L. M. Protein and DNA modifications: evolutionary imprints of bacterial biochemical diversification and geochemistry on the provenance of eukaryotic epigenetics. Cold Spring Harbor perspectives in biology 6, a016063 (2014). 24984775 10.1101/cshperspect.a016063 PMC4067991

[b41] Rasmussen M., Jacobsson M. & Björck L. Genome-based Identification and Analysis of Collagen-related Structural Motifs in Bacterial and Viral Proteins. Journal of Biological Chemistry 278, 32313–32316 (2003). 12788919 10.1074/jbc.M304709200

[b42] Sebe-Pedros A. & Ruiz-Trillo I. Integrin-mediated adhesion complex: Cooption of signaling systems at the dawn of Metazoa. Communicative & integrative biology 3, 475–477 (2010). 21057645 10.4161/cib.3.5.12603 PMC2974085

[b43] Sebe-Pedros A. *et al.* Regulated aggregative multicellularity in a close unicellular relative of metazoa. eLife 2, e01287 (2013). 24368732 10.7554/eLife.01287 PMC3870316

[b44] Fairclough S. R. *et al.* Premetazoan genome evolution and the regulation of cell differentiation in the choanoflagellate Salpingoeca rosetta. Genome biology 14, R15 (2013). 23419129 10.1186/gb-2013-14-2-r15 PMC4054682

[b45] Heinemann S. *et al.* Ultrastructural studies on the collagen of the marine sponge Chondrosia reniformis Nardo. Biomacromolecules 8, 3452–3457 (2007). 17944515 10.1021/bm700574y

[b46] Boute N. *et al.* Type IV collagen in sponges, the missing link in basement membrane ubiquity. Biology of t*he cell* 88, 37–44 (1996). 9175266 10.1016/s0248-4900(97)86829-3

[b47] Kim S. N. *et al.* ECM stiffness regulates glial migration in Drosophila and mammalian glioma models. Development 141, 3233–3242 (2014). 25063458 10.1242/dev.106039

[b48] Thomassin L. *et al.* The Pro-regions of lysyl oxidase and lysyl oxidase-like 1 are required for deposition onto elastic fibers. J Biol Chem 280, 42848–42855 (2005). 16251195 10.1074/jbc.M506832200

[b49] Horiguchi M. *et al.* Fibulin-4 conducts proper elastogenesis via interaction with cross-linking enzyme lysyl oxidase. Proceedings of the National Academy of Sciences of the United States of America 106, 19029–19034 (2009). 19855011 10.1073/pnas.0908268106 PMC2776456

[b50] Papke C. L. & Yanagisawa H. Fibulin-4 and fibulin-5 in elastogenesis and beyond: Insights from mouse and human studies. Matrix Biol (2014). 10.1016/j.matbio.2014.02.004 PMC4156930 24613575

[b51] Cronshaw A. D., Fothergill-Gilmore L. A. & Hulmes D. J. The proteolytic processing site of the precursor of lysyl oxidase. Biochem J 306 (Pt 1), 279–284 (1995). 7864821 10.1042/bj3060279 PMC1136513

[b52] Trackman P. C., Bedell-Hogan D., Tang J. & Kagan H. M. Post-translational glycosylation and proteolytic processing of a lysyl oxidase precursor. J Biol Chem 267, 8666–8671 (1992). 1349020

[b53] Uzel M. I. *et al.* Multiple bone morphogenetic protein 1-related mammalian metalloproteinases process pro-lysyl oxidase at the correct physiological site and control lysyl oxidase activation in mouse embryo fibroblast cultures. J Biol Chem 276, 22537–22543 (2001). 11313359 10.1074/jbc.M102352200

[b54] Borel A. *et al.* Lysyl oxidase-like protein from bovine aorta. Isolation and maturation to an active form by bone morphogenetic protein-1. J Biol Chem 276, 48944–48949 (2001). 11684696 10.1074/jbc.M109499200

[b55] Moali C. & Hulmes D. J. in *Extracellular Matrix:* Pathobiology *and Signaling*. (ed Karamanos N. ) 539–561 (Walter de Gruyter, 2012).

[b56] To W. & Midwood K. Plasma and cellular fibronectin: distinct and independent functions during tissue repair. Fibrogenesis & Tissue Repair 4, 21 (2011). 21923916 10.1186/1755-1536-4-21 PMC3182887

[b57] Fogelgren B. *et al.* Cellular fibronectin binds to lysyl oxidase with high affinity and is critical for its proteolytic activation. J Biol Chem 280, 24690–24697 (2005). 15843371 10.1074/jbc.M412979200

[b58] Maruhashi T., Kii I., Saito M. & Kudo A. Interaction between periostin and BMP-1 promotes proteolytic activation of lysyl oxidase. J Biol Chem 285, 13294–13303 (2010). 20181949 10.1074/jbc.M109.088864 PMC2857065

[b59] Kudo A. Periostin in fibrillogenesis for tissue regeneration: periostin actions inside and outside the cell. Cellular and molecular life sciences 68, 3201–3207 (2011). 21833583 10.1007/s00018-011-0784-5 PMC3173633

[b60] Rinke C. *et al.* Insights into the phylogeny and coding potential of microbial dark matter. Nature 499, 431–437 (2013). 23851394 10.1038/nature12352

[b61] Finn R. D., Clements J. & Eddy S. R. HMMER web server: interactive sequence similarity searching. Nucleic acids research 39, W29–37 (2011). 21593126 10.1093/nar/gkr367 PMC3125773

[b62] Punta M. *et al.* The Pfam protein families database. Nucleic acids research 40, D290–301 (2012). 22127870 10.1093/nar/gkr1065 PMC3245129

[b63] Katoh K. & Standley D. M. MAFFT multiple sequence alignment software version 7: improvements in performance and usability. Mol Biol Evol 30, 772–780 (2013). 23329690 10.1093/molbev/mst010 PMC3603318

[b64] Darriba D., Taboada G. L., Doallo R. & Posada D. ProtTest 3: fast selection of best-fit models of protein evolution. Bioinformatics (Oxford, England) 27, 1164–1165 (2011). 10.1093/bioinformatics/btr088 PMC5215816 21335321

[b65] Stamatakis A. RAxML version 8: a tool for phylogenetic analysis and post-analysis of large phylogenies. Bioinformatics (Oxford, England) 30, 1312–1313 (2014). 10.1093/bioinformatics/btu033 PMC3998144 24451623

[b66] Lartillot N., Lepage T. & Blanquart S. PhyloBayes 3: a Bayesian software package for phylogenetic reconstruction and molecular dating. Bioinformatics (Oxford, England) 25, 2286–2288 (2009). 10.1093/bioinformatics/btp368 19535536

[b67] He D. *et al.* An alternative root for the eukaryote tree of life. Curr Biol 24, 465–470 (2014). 24508168 10.1016/j.cub.2014.01.036

[b68] Derelle R. & Lang B. F. Rooting the eukaryotic tree with mitochondrial and bacterial proteins. Mol Biol Evol 29, 1277–1289 (2012). 22135192 10.1093/molbev/msr295

[b69] Dunn C. W. *et al.* Broad phylogenomic sampling improves resolution of the animal tree of life. Nature 452, 745–749 (2008). 18322464 10.1038/nature06614

[b70] Finn R. D. *et al.* Pfam: the protein families database. Nucleic acids research 42, D222–230 (2014). 24288371 10.1093/nar/gkt1223 PMC3965110

[b71] Petersen T. N., Brunak S., von Heijne G. & Nielsen H. SignalP 4.0: discriminating signal peptides from transmembrane regions. Nat Meth 8, 785–786 (2011). 10.1038/nmeth.1701 21959131

[b72] Krogh A., Larsson B., von Heijne G. & Sonnhammer E. L. Predicting transmembrane protein topology with a hidden Markov model: application to complete genomes. J Mol Biol 305, 567–580 (2001). 11152613 10.1006/jmbi.2000.4315

[b73] Jones P. *et al.* InterProScan 5: genome-scale protein function classification. Bioinformatics (Oxford, England) 30, 1236–1240 (2014). 10.1093/bioinformatics/btu031 PMC3998142 24451626

[b74] Smoot M. E., Ono K., Ruscheinski J., Wang P. L. & Ideker T. Cytoscape 2.8: new features for data integration and network visualization. Bioinformatics (Oxford, England) 27, 431–432 (2011). 10.1093/bioinformatics/btq675 PMC3031041 21149340

