# Supplementary material for: Origin and evolution of lysyl oxidases
Source: Sci Rep. 2015 May 29;5:10568. doi: 10.1038/srep10568 (PMC4448552; doi:10.1038/srep10568)

Origin and evolution of lysyl oxidases

Xavier Grau-Bové[1], Iñaki Ruiz-Trillo[1,3,4], Fernando Rodriguez-Pascual[2]

[1] Institut de Biologia Evolutiva (CSIC-Universitat Pompeu Fabra), Barcelona, Spain

[2] Centro de Biología Molecular "Severo Ochoa" Consejo Superior de Investigaciones Científicas (C.S.I.C.) / Universidad Autónoma de Madrid (Madrid), Madrid, Spain

[3] Departament de Genètica, Universitat de Barcelona, Barcelona, Spain

[4] Institució Catalana de Recerca i Estudis Avançats (ICREA), Barcelona, Spain

Supplementary Material

Tables S1 and S2.

Files S1 to S8.

Figure legends

Table S1. Taxon sampling used in the eukaryotic+prokaryotic phylogenies. It includes the taxonomic classification, the acronym used in the present study (in brackets) and the source of the proteome data.

Table S2. Extended sampling of animal genomes, used in the holozoan phylogenies. It includes the taxonomic classification, the acronym used in the present study (in brackets) and the source of the proteome data.

File S1. Maximum likelihood (ML) phylogenetic tree of 154 eukaryotic and prokaryotic LOX, using dataset from supplementary Table S1 and prokaryote genomes (see Materials and Methods). Trees rooted using the midpoint-rooted tree option. Bootstrap supports are indicated at each node. Sequences are color-coded according to taxonomic assignment (see legend).

File S2. Bayesian inference (BI) phylogenetic tree of 154 eukaryotic and prokaryotic LOX, using dataset from supplementary Table S1 and prokaryote genomes (see Materials and Methods). Trees rooted using the midpoint-rooted tree option. Bayesian Posterior Probabilities are indicated at each node. Sequences are color-coded according to taxonomic assignment (see legend).

File S3. Maximum likelihood (ML) phylogenetic tree of 129 metazoan and ichthyosporean LOX, using dataset from supplementary Table S2 and ichthyosporean genomes (see Materials and Methods). Trees rooted using the midpoint-rooted tree option. Bootstrap supports are indicated at each node. Sequences are color-coded according to taxonomic assignment (see legend).

File S4. Bayesian inference (ML) phylogenetic tree of 129 metazoan and ichthyosporean LOX, using dataset from supplementary Table S2 and ichthyosporean genomes (see Materials and Methods). Trees rooted using the midpoint-rooted tree option. Bayesian Posterior Probabilities are indicated at each node. Sequences are color-coded according to taxonomic assignment (see legend).

File S5. Fasta file with 154 eukaryotic and prokaryotic LOX sequences (complete proteins), extracted from dataset from supplementary Table S1 and prokaryote genomes (see Materials and Methods).

File S6. Fasta file with 129 metazoan and ichthyosporean LOX sequences (complete proteins), extracted from dataset from supplementary Table S2 and ichthyosporean genomes (see Materials and Methods).

File S7. GFF (General Feature Format) file showing molecular features in the protein domain architectures of sequences in File S5 (154 eukaryotic and prokaryotic LOX sequences).

File S8. GFF (General Feature Format) file showing molecular features in the protein domain architectures of sequences in File S6 (129 metazoan and ichthyosporean LOX sequences).

Table S1

Taxonomic classification		Species	Source
**METAZOA**	Deuterostomia	*Homo sapiens (Hsap)*	Ensembl
		Ciona intestinalis (Cint)	Ensembl
		Saccoglossus kowalevskii (Skow)	NCBI
		Strongylocentrotus purpuratus (Spur)	NCBI
	Ecdysozoa	*Drosophila melanogaster (Dmel)*	Flybase *http://flybase.org/*
		Caenorhabditis elegans (Cele)	Ensembl
	Lophotrochozoa	*Capitella teleta (Ctel)*	JGI
		Echinoccocus multilocularis (Emul)	Wellcome Trust Sanger Institute *http://www.sanger.ac.uk/resources/downloads/helminths /echinococcus-multilocularis.html*
		Lottia gigantea (Lgig)	JGI *http://genome.jgi.doe.gov/*
		Crassostrea gigas (Cgig)	Oyster Genome Project webpage *http://www.oysterdb.cn*
	Cnidaria	*Nematostella vectensis (Nvec)*	NCBI
		Acropora digitifera (Adig)	*Acropora digitifera* Genome Project webpage *http://marinegenomics.oist.jp/*
		Hydra magnipapillata (Hmag)	NCBI
	Placozoa	*Trichoplax adhaerens (Tadh)*	NCBI
	Ctenophora	*Mnemiopsis leidyi (Mlei)*	*M. leidyi* Genome Project webpage *http://research.nhgri.nih.gov/mnemiopsis/*
	Porifera	*Oscarella carmela (Ocar)*	Courtesy of Scott Nichols
		Amphimedon queenslandica (Aque)	NCBI
		Sycon ciliatum (Scil)	Courtesy of Maja Adamska
		Leucosolenia complicate (Lcom)	Courtesy of Maja Adamska
**CHOANOFLAGELLATA**		*Monosiga brevicollis (Mbre)*	NCBI
		Salpingoeca rosetta (Sros)	Broad Institute *http://www.broadinstitute.org/annotation /genome/multicellularity_project/MultiHome.html*
**FILASTEREA**		*Capsaspora owczarzaki (Cowc)*	Broad Institute
		Ministeria vibrans (Mvib)	RNASeq data
**ICHTHYOSPOREA**		*Sphaeroforma arctica (Sarc)*	Broad Institute
		Creolimax fragrantissima (Cfra)	Beijing Genome Institute (Beijing, PRC)
		Abeoforma whisleri (Awhi)	RNASeq data
		Pirum gemmata (Pgem)	RNASeq data
		Amoebidium parasiticum (Apar)	RNASeq data
	Incertae sedis	*Corallochytrium limacisporum (Clim)*	The Genome Analysis Centre (Norwich, UK)
**FUNGI**	Ascomycota	*Schizosaccharomyces pombe (Spom)*	NCBI
		Tuber melanosporum (Tmel)	NCBI
		Neurospora crassa (Ncra)	Broad Institute *http://www.broadinstitute.org/scientific-community/science/projects/fungal-genome-initiative*
		Saccharomyces cerevisiae (Scer)	Broad Institute
	Basidiomycota	*Cryptococcus neoformans (Cneo)*	NCBI
		Coprinopsis cinerea (Ccin)	Broad Institute
		Ustilago maydis (Umay)	NCBI
	Incertae sedis	*Mortierella verticillata (Mver)*	Broad Institute
	Mucoromycotina	*Phyomyces blakesleeanus (Pbla)*	JGI
		Mucor circinelloides (Mcir)	JGI
		Rhizopus oryzae (Rory)	Broad Institute
		Umbelopsis ramanniana (Umar)	JGI Fungi Portal *Fueling the future with fungal genomics*, I Grigoriev *et al.*, Mycology 2011
	Blastocladio-mycota	*Allomyces macrogynus (Amac)*	Broad Institute
		Catenaria anguillulae (Cang)	JGI Fungi Portal *Fueling the future with fungal genomics*, I Grigoriev *et al.*, Mycology 2011
	Chytridiomycota	*Batrachochytrium dendrobatidis (Bden)*	JGI
		Spizellomyces punctatus (Spun)	Broad Institute
	Glomeromycota	*Rhizophagus irregularis (Rirr)*	JGI Fungi Portal *Fueling the future with fungal genomics*, I Grigoriev *et al.*, Mycology 2011
	Kickxellomycotina	*Coemansia reversa (Crev)*	JGI Fungi Portal *Fueling the future with fungal genomics*, I Grigoriev *et al.*, Mycology 2011
	Entomophthoro-mycota	*Conidiobolus coronatus (Ccor)*	JGI Fungi Portal *Fueling the future with fungal genomics*, I Grigoriev *et al.*, Mycology 2011

	Monoblepharido-mycota	*Gonapodya prolifera (Gpro)*	JGI Fungi Portal<br>*Fueling the future with fungal genomics*, I Grigoriev *et al.*, Mycology 2011
	Neocallimastigo-mycota	*Piromyces sp. E2 (Pisp)*	JGI Fungi Portal<br>*Fueling the future with fungal genomics*, I Grigoriev *et al.*, Mycology 2011
	Microsporidia	*Encephalitozoon cuniculi (Ecun)*	NCBI
		Nematocida parisii (Npar)	NCBI
	Cryptomycota	*Rozella allomycis (Rall)*	JGI
**NUCLEARIIDAE AND FONTICULA GROUP**		*Nuclearia spp. (Nspp)*	RNAseq data
		Fonticula alba (Falb)	Broad Institute<br>*http://www.broadinstitute.org/annotation/genome/multicellularity_project/Downloads.html*
**APUSOZOA**		*Thecamonas trahens (Ttra)*	Broad Institute<br>*http://www.broadinstitute.org/annotation/genome/multicellularity_project/Downloads.html*
**AMOEBOZOA**	Mycetozoa	*Dictyostelium discoideum (Ddis)*	NCBI
		Polysphondylium pallidum (Ppal)	NCBI
	Archamoeba	*Entamoeba histolytica (Ehis)*	Wellcome Trust Sanger Institute<br>*http://www.sanger.ac.uk/resources/downloads/protozoa/entamoeba.html*
	Centramoebida	*Acanthamoeba castellanii (Acas)*	*Ab initio protein prediction*
**VIRIDIPLANTAE**	Embryophyta	*Arabidopsis thaliana (Atha)*	NCBI
		Mimulus guttatus (Mgut)	Phytozome (JGI)<br>*http://www.phytozome.net/*
		Aquilegia coerulea (Acoe)	Phytozome (JGI)
		Brachypodium distachyon (Bdis)	Phytozome (JGI)
		Sorghum bicolor (Sbic)	Phytozome (JGI)
		Selaginella moellendorffii (Smoe)	NCBI
		Physcomitrella patens (Ppat)	NCBI
	Chlorophyta	*Chlamydomonas reinhardtii (Crei)*	NCBI
		Volvox cartieri (Vcar)	NCBI
		Chlorella variabilis (Cvar)	NCBI
		Ostreococcus tauri (Otau)	NCBI
		Micromonas pusilla (Mpus)	JGI
	Rhodophyta	*Cyanidioschyzon merolae (Cmer)*	*C. merolae* Genome Project webpage<br>*http://merolae.biol.s.u-tokyo.ac.jp/*
		Chondrus crispus (Ccri)	*C. crispus* Genome Project webpage<br>Courtesy of Mark Cock
		Pyropia yezoensis (Pyez)	*P. yezoensis* Genome Project webpage<br>*http://nrifs.fra.affrc.go.jp/ResearchCenter/5_AG/genomes/nori/*
	Glaucophyta	*Cyanophora paradoxa (Cpar)*	*C. paradoxa* Genome Project webpage<br>*http://cyanophora.rutgers.edu/cyanophora/*
**HETEROKONTA/ STRAMENOPILES**	Brown algae/ Phaeophyceae	*Ectocarpus siliculosus (Esil)*	EMBL
		Nannochloropsis gaditana (Ngad)	*N. gaditana* Genome Project webpage<br>*http://nannochloropsis.genomeprojectsolutions-databases.com/*
		Aureococcus anophagefferens (Aano)	Uniprot
		Phaeodactylum tricornutum (Ptri)	NCBI
		Thalassiossira pseudonana (Tpse)	NCBI
	Oomycota	*Phytophthora infestans (Pinf)*	NCBI
	Labyrinthulo-mycetes	*Aplanochytrium kerguelense (Aker)*	JGI
		Schizochytrium aggregatum (Sagg)	JGI
**ALVEOLATA**	Apicomplexa	*Toxoplasma gondii (Tgon)*	NCBI
		Plasmodium falciparum (Pfal)	Uniprot
	Ciliophora	*Paramecium tetraurelia (Ptet)*	Uniprot
		Tetrahymena thermophila (Tthe)	NCBI
	Perkinsidae	*Perkinsus marinus (Pmar)*	NCBI
	Dinoflagellata	*Symbiodinium minutum (Smin)*	*Symbiodinium minutum* Clade B1 Genome Project webpage *http://marinegenomics.oist.jp/*
**RHIZARIA**		*Bigelowiella natans (Bnat)*	JGI
**HAPTOPHYTA**		*Emiliania huxleyi (Ehux)*	JGI
**CRYPTOPHYTA**		*Guillardia theta (Gthe)*	JGI
**EXCAVATA**	Heterolobosea	*Naegleria gruberi (Ngru)*	NCBI
	Kinetoplastida	*Bodo saltans (Bsal)*	Wellcome Trust Sanger Institute<br>*http://www.sanger.ac.uk/resources/downloads/protozoa/bodo-saltans.html*
		Trypanosoma cruzi (Tcru)	NCBI
		Leishmania major (Lmaj)	NCBI
	Metamonada	*Trichomonas vaginalis (Tvag)*	NCBI
		Giardia lamblia (Glam)	NCBI

Table S2

Taxonomic classification			Species	Source
**DEUTEROSTOMIA**	**Chordata**	**Vertebrata**	*Homo sapiens (Hsap)*	Ensembl
			Mus musculus (Mmus)	Ensembl
			Gallus gallus (Ggal)	Ensembl
			Anolis carolinensis (Acar)	Ensembl
			Xenopus tropicalis (Xtro)	Ensembl
			Danio rerio (Drer)	Ensembl
			Latimeria chalumnae (Lcha)	Ensembl
			Callorhinchus milii (Cmil)	Elephant Shark Genome Project<br>*http://esharkgenome.imcb.a-star.edu.sg/*
			Petromyzon marinus (Pema)	Ensembl
		**Tunicata**	*Ciona intestinalis (Cint)*	Ensembl
			Ciona savignyi (Csav)	Ensembl
			Oikopleura dioica (Odio)	Genoscope<br>*http://www.genoscope.cns.fr/externe/GenomeBrowser/Oikopleura/*
		**Cephalochordata**	*Branchiostoma floridae (Bflo)*	JGI
	**Ambulacraria**	**Hemichordata**	*Saccoglossus kowalevskii (Skow)*	NCBI
		**Echinodermata**	*Strongylocentrotus purpuratus (Spur)*	NCBI
**ECDYSOZOA**	**Arthropoda**	**Hexapoda**	*Drosophila melanogaster (Dmel)*	Flybase<br>*http://flybase.org/*
			Tribolium castaneum (Tcas)	Beetlebase<br>*http://www.beetlebase.org/*
		**Crustacea**	*Daphnia pulex (Dpul)*	JGI
		**Arachnida**	*Ixodes scapularis (Isca)*	NCBI
	**Nematoda**		*Caenorhabditis elegans (Cele)*	Ensembl
			Trichinella spiralis (Tspi)	NCBI
			Brugia malayi (Bmal)	NCBI
**LOPHOTROCHOZOA**	**Spiralia**	**Mollusca**	*Lottia gigantea (Lgig)*	JGI<br>*http://genome.jgi.doe.gov/*
			Aplysia californica (Acal)	NCBI
			Crassostrea gigas (Cgig)	Oyster Genome Project<br>*http://oysterdb.cn/*
		**Annelida**	*Capitella teleta (Ctel)*	JGI
			Helobdella robusta (Hrob)	JGI
	**Platyhelminthes**		*Echinoccocus multilocularis (Emul)*	Wellcome Trust Sanger Institute<br>*http://www.sanger.ac.uk/resources/downloads/helminths/echinococcus-multilocularis.html*
			Schistosoma mansoni (Sman)	NCBI
**CNIDARIA**			*Nematostella vectensis (Nvec)*	NCBI
			Acropora digitifera (Adig)	*Acropora digitifera* Genome Project<br>*http://marinegenomics.oist.jp/*
**PLACOZOA**			*Trichoplax adhaerens (Tadh)*	NCBI
**CTENOPHORA**			*Mnemiopsis leidyi (Mlei)*	*M. leidyi* Genome Project<br>*http://research.nhgri.nih.gov/mnemiopsis/*
**PORIFERA**			*Oscarella carmela (Ocar)*	Courtesy of Scott Nichols
			Amphimedon queenslandica (Aque)	NCBI
			Sycon ciliatum (Scil)	Courtesy of Maja Adamska
			Leucosolenia complicata (Lcom)	Courtesy of Maja Adamska

Figure S1

ML 154 eukaryotes+prokaryotes

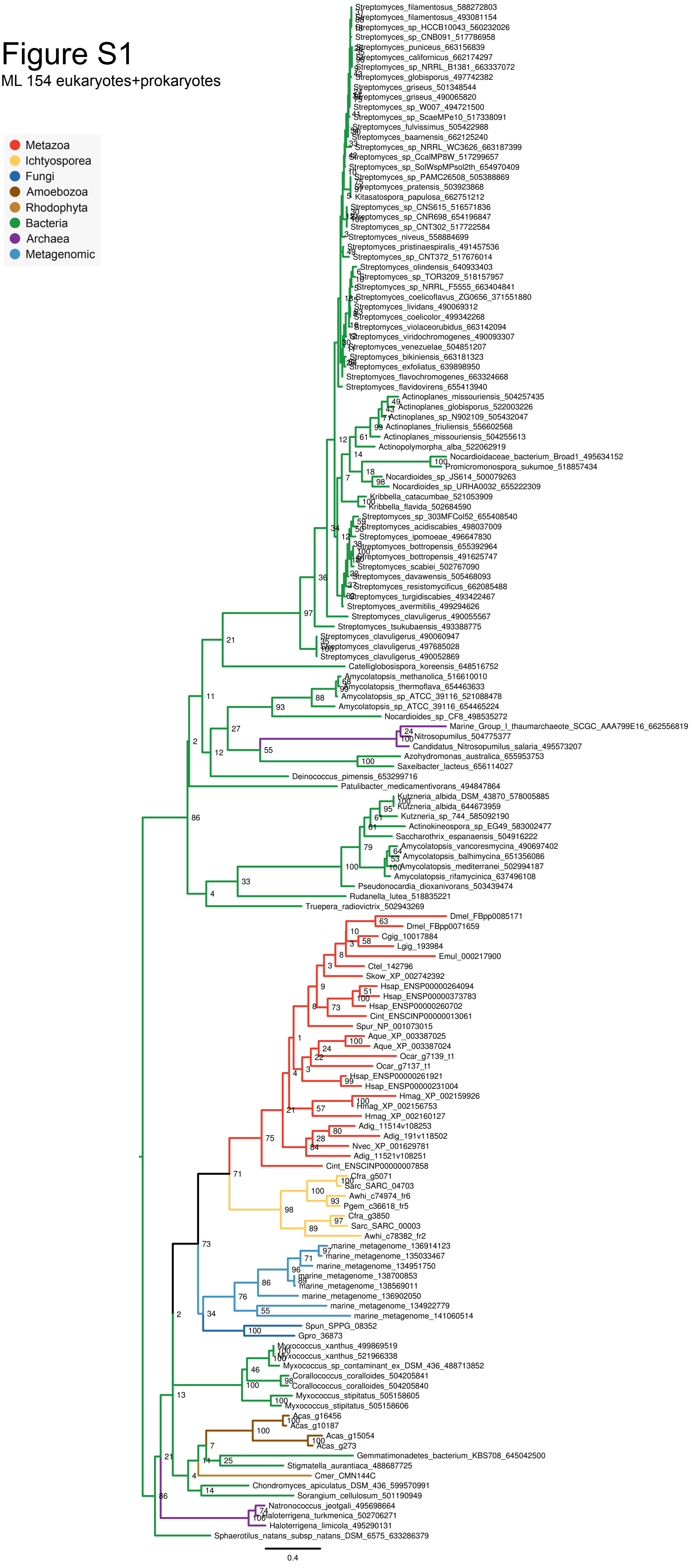

Figure S2

BI 154 eukaryotes+prokaryotes

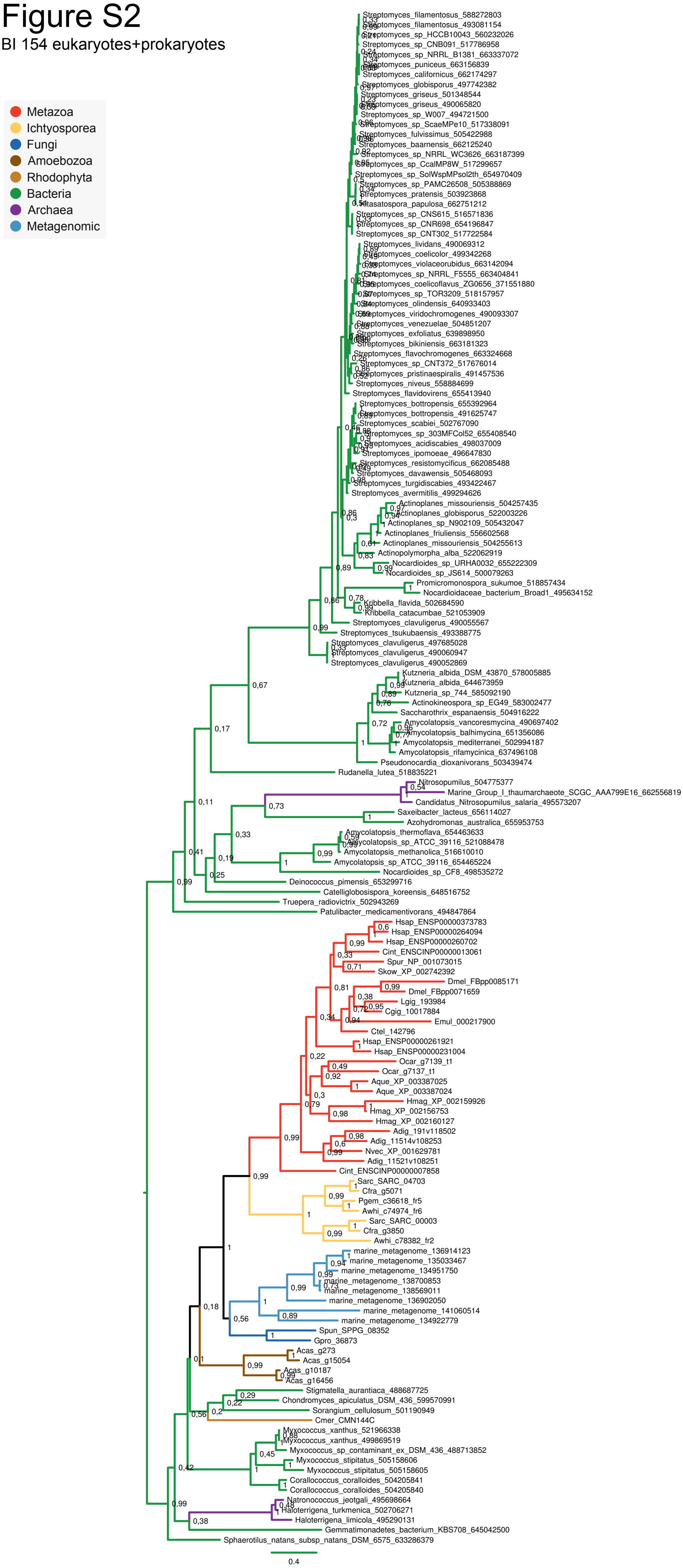

Figure S3

ML 129 holozoans

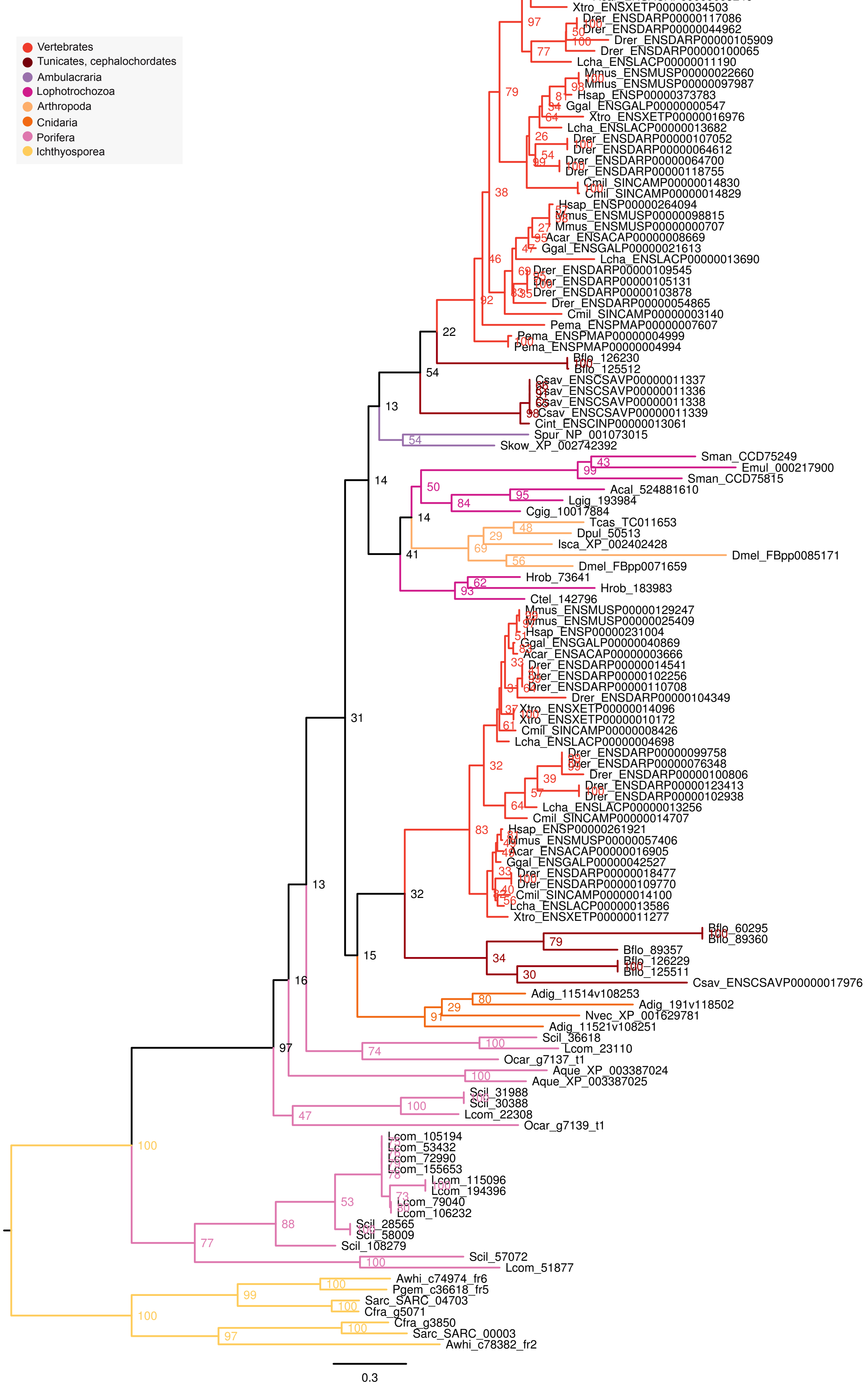

Figure S4

BI 129 holozoans

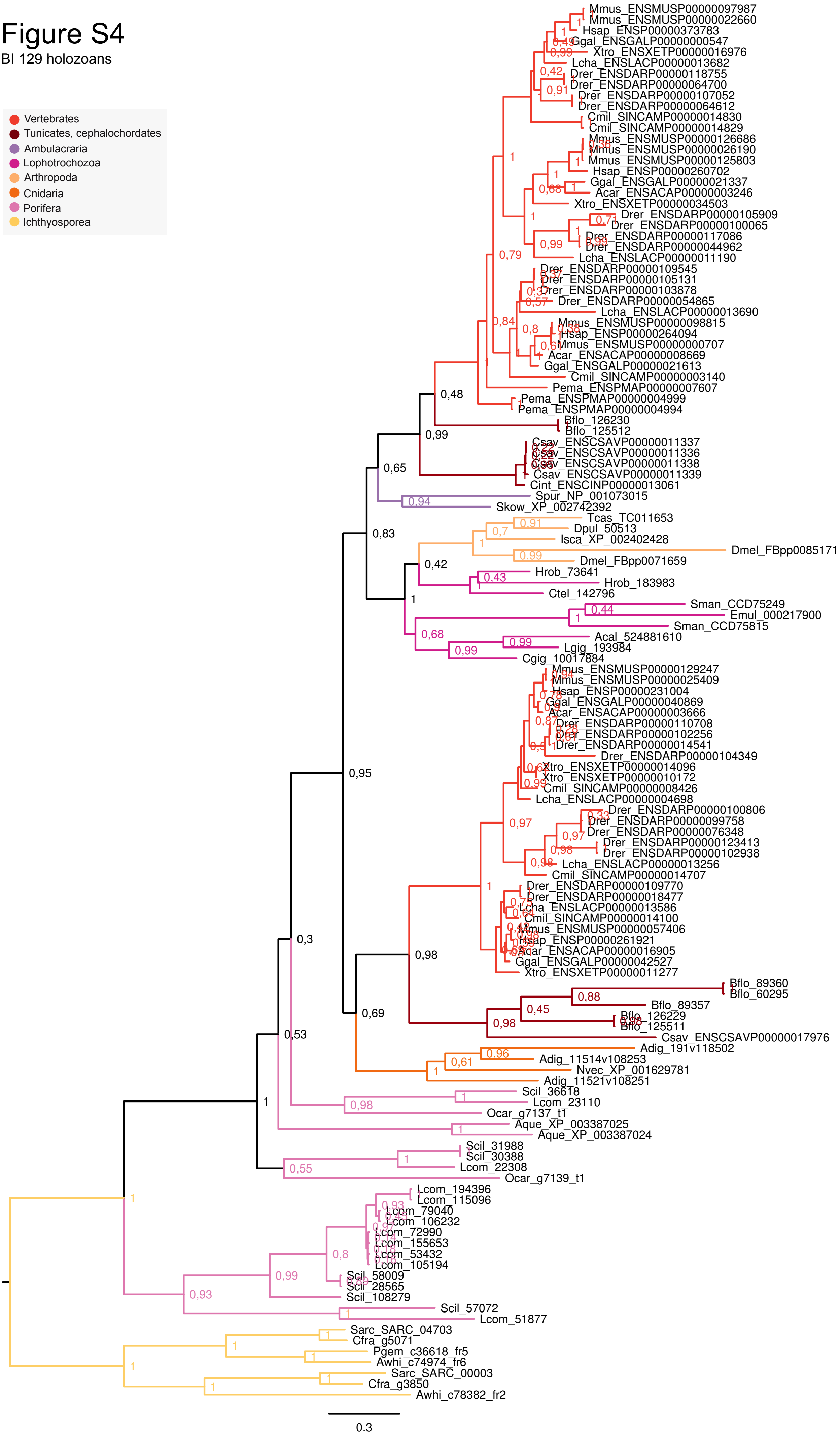

Supplement: Supporting Information [file srep10568-s1.pdf]
