# Supplementary material for: Origin and evolution of lysyl oxidases
Source: Sci Rep. 2015 May 29;5:10568. doi: 10.1038/srep10568 (PMC4448552; doi:10.1038/srep10568)
Supplement: File S5 [file srep10568-s2.doc]

>Hsap_ENSP00000264094

MRPVSVWQWSPWGLLLCLLCSSCLGSPSPSTGPEKKAGSQGLRFRLAGFPRKPYEGRVEIQRAGEWGTICDDDFTLQAAHILCRELGFTEATGWTHSAKYGPGTGRIWLDNLSCSGTEQSVTECASRGWGNSDCTHDEDAGVICKDQRLPGFSDSNVIEVEHHLQVEEVRIRPAVGWGRRPLPVTEGLVEVRLPDGWSQVCDKGWSAHNSHVVCGMLGFPSEKRVNAAFYRLLAQRQQHSFGLHGVACVGTEAHLSLCSLEFYRANDTARCPGGGPAVVSCVPGPVYAASSGQKKQQQSKPQGEARVRLKGGAHPGEGRVEVLKASTWGTVCDRKWDLHAASVVCRELGFGSAREALSGARMGQGMGAIHLSEVRCSGQELSLWKCPHKNITAEDCSHSQDAGVRCNLPYTGAETRIRLSGGRSQHEGRVEVQIGGPGPLRWGLICGDDWGTLEAMVACRQLGLGYANHGLQETWYWDSGNITEVVMSGVRCTGTELSLDQCAHHGTHITCKRTGTRFTAGVICSETASDLLLHSALVQETAYIEDRPLHMLYCAAEENCLASSARSANWPYGHRRLLRFSSQIHNLGRADFRPKAGRHSWVWHECHGHYHSMDIFTHYDILTPNGTKVAEGHKASFCLEDTECQEDVSKRYECANFGEQGITVGCWDLYRHDIDCQWIDITDVKPGNYILQVVINPNFEVAESDFTNNAMKCNCKYDGHRIWVHNCHIGDAFSEEANRRFERYPGQTSNQII

>Hsap_ENSP00000373783

MERPLCSHLCSCLAMLALLSPLSLAQYDSWPHYPEYFQQPAPEYHQPQAPANVAKIQLRLAGQKRKHSEGRVEVYYDGQWGTVCDDDFSIHAAHVVCRELGYVEAKSWTASSSYGKGEGPIWLDNLHCTGNEATLAACTSNGWGVTDCKHTEDVGVVCSDKRIPGFKFDNSLINQIENLNIQVEDIRIRAILSTYRKRTPVMEGYVEVKEGKTWKQICDKHWTAKNSRVVCGMFGFPGERTYNTKVYKMFASRRKQRYWPFSMDCTGTEAHISSCKLGPQVSLDPMKNVTCENGLPAVVSCVPGQVFSPDGPSRFRKAYKPEQPLVRLRGGAYIGEGRVEVLKNGEWGTVCDDKWDLVSASVVCRELGFGSAKEAVTGSRLGQGIGPIHLNEIQCTGNEKSIIDCKFNAESQGCNHEEDAGVRCNTPAMGLQKKLRLNGGRNPYEGRVEVLVERNGSLVWGMVCGQNWGIVEAMVVCRQLGLGFASNAFQETWYWHGDVNSNKVVMSGVKCSGTELSLAHCRHDGEDVACPQGGVQYGAGVACSETAPDLVLNAEMVQQTTYLEDRPMFMLQCAMEENCLSASAAQTDPTTGYRRLLRFSSQIHNNGQSDFRPKNGRHAWIWHDCHRHYHSMEVFTHYDLLNLNGTKVAEGHKASFCLEDTECEGDIQKNYECANFGDQGITMGCWDMYRHDIDCQWVDITDVPPGDYLFQVVINPNFEVAESDYSNNIMKCRSRYDGHRIWMYNCHIGGSFSEETEKKFEHFSGLLNNQLSPQ

>Hsap_ENSP00000260702

MAWSPPATLFLFLLLLGQPPPSRPQSLGTTKLRLVGPESKPEEGRLEVLHQGQWGTVCDDNFAIQEATVACRQLGFEAALTWAHSAKYGQGEGPIWLDNVRCVGTESSLDQCGSNGWGVSDCSHSEDVGVICHPRRHRGYLSETVSNALGPQGRRLEEVRLKPILASAKQHSPVTEGAVEVKYEGHWRQVCDQGWTMNNSRVVCGMLGFPSEVPVDSHYYRKVWDLKMRDPKSRLKSLTNKNSFWIHQVTCLGTEPHMANCQVQVAPARGKLRPACPGGMHAVVSCVAGPHFRPPKTKPQRKGSWAEEPRVRLRSGAQVGEGRVEVLMNRQWGTVCDHRWNLISASVVCRQLGFGSAREALFGARLGQGLGPIHLSEVRCRGYERTLSDCPALEGSQNGCQHENDAAVRCNVPNMGFQNQVRLAGGRIPEEGLLEVQVEVNGVPRWGSVCSENWGLTEAMVACRQLGLGFAIHAYKETWFWSGTPRAQEVVMSGVRCSGTELALQQCQRHGPVHCSHGGGRFLAGVSCMDSAPDLVMNAQLVQETAYLEDRPLSQLYCAHEENCLSKSADHMDWPYGYRRLLRFSTQIYNLGRTDFRPKTGRDSWVWHQCHRHYHSIEVFTHYDLLTLNGSKVAEGHKASFCLEDTNCPTGLQRRYACANFGEQGVTVGCWDTYRHDIDCQWVDITDVGPGNYIFQVIVNPHYEVAESDFSNNMLQCRCKYDGHRVWLHNCHTGNSYPANAELSLEQEQRLRNNLI

>Skow_XP_002742392

MELVCRPAPWLATIAMVVIWTLSSNTNGQSIGNDEDLKIQLRLMDGRSIYEGRVEIFVNDEMGTICDDYFTLTTANVICKDLGFAGAESFYYGGHFNPGTGRIWLDSLRCDGNESSIKECSHAPWGVTDCSHDEDVGVRCKSSMIANTDFEDSESESNQLTSDNEDSVGRREHGPMRVDLNVGSLKVRLRGRRTAYPKSEGYVEVYFRNRWRPVCADGWDMADSRVVCGQMGFSEAIPIKLLEFKDKMRTRMQNAWLTNVTCRGVESTLQECSYHTLSNAEQCISGMPATARCERGHFVEDSRGKKTDNVLSEYLGDDAILQKPIVRLKSGPYLGSGRVEVFYDGKWGTVCGDSWNMASANVICRELGFGTAKEISLSSDTYGQGTRNVWLDKVNCTGDENSIFDCPHSTWNYAGESLNGFCTHANDAGVVCHVPGQRGLLKIRLVGGRTEMEGRLEVQHGTTWGSVCSDEWDMRSAMVACRQLGLGFAHQPLRDVTYFGGLDVPIIMSGVSCRGDELSLNQCKHDGWHTPKCDQYHIAGVVCSTSLPDLVPDLGLIESTAYIDERPFGLLQCAMEENCVSSSAYDVIPGTMPFIFGTRRLLRFSSSIANRGTSDFIPVTDKRYWEWHQCHQHHHSMEVFATYDLLNSEGYKVAEGHKASFCLEDTHCDIGVSKHYSCLNYGDQGISVNCIDEYKHTIDCQWIDISDIEPGFYILRVHVNPNVFVAESDYSNNDVLCNLAYNGYEISVNNCRFASEE

>Adig_11514v108253

MRWILLASIALLSVELSCSVKVRLVGGKHKYEGRIEILYKSEWRAVCDHKWNKNGARIVCRMLGYPDVLRFTKGPHAFGRGNGKFWLDDVVCSGEEESIASCSHRPWGRNNCLSFNQAGVVCKRHMSDMVAVPKPSIASGEVRKLNIRLQGPVVDDYISEGVVQVQHEGIWGYICPSTWTKANSFVLCGDLGFPNMEIQGSNSAASQEEQPVYWLNKVTCQGWESSIVSCDHAGFARHQCDDDGVLRIKCVRRHITKPLDVRLRSGVLVSEGRVEVQYQDFWGTVCDDHWTLKEANVVCRSLGYGSAAMAATNAYFGRGMGRVLLDDVNCHGTERHITQCRHRGWRRSNCNNHEDASVSCHAPALQGHQIRIQGSTNPKEGRVEVFHDGKWGTVCGDTWGIEEAMTVCRQLNLGYAGRALTENNFTATEHRVIMSGVRCRVDEVSLYNCQHDEWTNTTCSSQKSVAGVVCVNELPDLVINTDMLKTYMEVNTIALQYLQCAMEENCLSASSAWMMRDSVWNRRRLLRFSVQVENRGLDHFRPAVDKSKWQWHKCHKHYHSMETFSSYDLLSQRTGKKVAQGHKASFCLEDTKCDPGFERVWNCTDMGDQGISPGCFDIYHYNIDCQWVDVSDLTHGAFYLRVQVNPGNQVAESDFRNNVAKCQVYDYGRYVIVGSCRIENCDSGVDTHGGNAGGDCCVFPFKFRGKLFHDCTTDSYSKKWCSTTFDFKKDRKWGLCHD

>Nvec_XP_001629781

KHYHSMEVFSTYDLIDDRGQKVAEGHKASFCLEDTGCDAGIHRHWNCTDGGDQGVKPNCYDEYKWTIDCQWIDVTDRPHGNYRLQIKVNPNQMVAETDYDNNIAMCDAYDYGSFLLMQNCYLGE

>Adig_11521v108251

MLHDVHCQGNETNISHCKNKGWKGSGCNHYEDASVRCHAPQLQGHKIRLSGGANAYEGRVEVFRSGAWGTVCADDWRIESAMVVCRQLKLGYAAHAVTQNYFGHTNLRVIMSGVQCHVDEISIFNCQRDHWENVTCSRSNKLAGVICSKALPDLVIDTAELQKTIIQEYRTLYDLRCAHEESCLSKSADALFKYGSLSRDYRKLLRFTTKIENRGWEDFRPDSPRGSWDYHRCHAHFHSMETFATYDLLGKRYSAIQAEGHKASFCLEDTECDPGFDKRWNCTRGGDQGISPGCYDVYKSTIDCQWVDFTDVRQRGSYILRIRLNPGNQVAETDFRNNIAKCSVIYYGRFVLPSHCWIEDCQSGVDTYGGNSRGNCCVFPFNYNGKVYEACTTDGFAKKWCATTKNYSKDKKWGLCFD

>Dmel_FBpp0071659

MCAIEDRKVLKMGLTLVCLTLLAIHMADAVVQHRSLEDARQERQRLVHRYTKVLNKEEGAIRLVGGDNEYEGNIEVLHNGKWGAVCDDEWDSTEADIVCRQLGFPGMRRYTRSGFFGPARRRFWMDNLFCEGHEQELVDCHFEGWGENDCEPGEAAGVVCYPPENALIPMATPIIRDEDLPKYPIHSRSRLYVRLRGGRSRIEGRVEVSLDGGRWGSVCADGWSLLEANVVCRQLGLGYASEAFQTDFFGGFNVSRPVLSGSECYGNETELADCLHHDASQGIISCHGNRQHVAAVICDYIAPDLVVDYLEIEQTAHLEDRPMLLMQCAMEENCVANEAYQIQRDDPHWRYRSRRLLKFTAAAINAGNADFRPFKEKSQWEWHMCHMHFHSMEVFATFDIFNLRGIKVAQGHKASFCLEDSNCLPGVAKKYNCANYGDQGISINCSDVYLYNLDCQWVDVTDLIPGTYVLKIAINPEFKVAEMNYDNNAAICDLIYTANFARVQNCQLGRP

>Lgig_193984

MLNLNCETMLNVCVALLVFLAATVDGLREGQIRLVGGKNDNDGTVVIYHNRKWGSICDDEWDMRDARVTCQQLGFPGAKKAARRSEFGRGRRRMWMSRVRCSGFESQLRYCMFFGWGRIRHRCRGGWRSAGVVCLPKKNPGTDGTPIYSTTPTPATTTEALHNSGVQIRIRGGRYFWEGRLEVKRNPEETAWGTVCGKVWSIRETMVACRELGLDFGKQALQVNYFGGQNMDKIYNEVKCTGRESRLDECFRVETADSVSCAGKHLVAGIVCSKFLPDLIPSLHRLEESILLQDRPLYYLQCALEENCLSSSATTIRNSSKNWMGASRRLLKFSTVVHNRGVADFRPYRAKGQWEWHQCHMHYHSMEVFAHYDIIDEHGNRLAEGSKASFCLEDSRCENGITPKYDCTGFGDQGLSVNCSDNYMNDIDCQWIDITDIPVGKYIFKMEVNPKLLVAELDYDNNVAVCDLTYTMYNAFLQNC

>Adig_191v118502

MTVCRQLNLGFASEAVINDRFHGRDPRVIMSGVTCRVDAISIYNCQHDPWTNTTCSSKGSSAGVVCVDELPDIVPDTEVLQRDMKINSIPLQYLRCPLEENCLSETADYEISDMTYYMRRLLRFSVKTDNVGLADFRPNVPRSKWKWHKCHKHYHSMETFSSYDLIKQDSGQKIAKGHKASFCLEDTNCYEGFDKKFNCSMDGGQGISPGCYDLYGWRIDCQWVDCTDFTHGAFYLRVHINPGNRRIVTVELTHMAAIQEEIVVSFHSFTMANYITTVPQMALSSDGAQQHSILEETVNGGFATTRARDEAKRQISQHIM

>Spur_NP_001073015

MQFSVGLFVQYFIFILITCGHGLPSNNETNTNNPTINTESTPIEPTTIAATTTTTSKPTTAKQEETTTQPPKTTQPTTVQPTTTQPTTTPTTFPPTTGEPTTRPPAKIRARLADGMHPWEGRVEVSMHGGSWGTVCDDGFNMNAANVVCKMVGYKKAVQYFFGSSQFGRGPGRILLDEVECLGTEKNLLACEHNPIGVTDCSHSEDVGIRCIDVVRKVTPPEKKLKQYKFRLVGDPTPKPQSEGVVEVMMTGKWRTICSDGWDMEDARVLCGSLGFKEATSVEPNRKNKSRQRKKSIVASNFECTGNEHNVSDCERTILKSHKDCLSKRAAEAKCERGPFVMDADKPYSPPWSDVRLKAGAAFGEGRLEVFHAGRWGTVCGEDFDKVAASVVCRKLGFGTAETVLENASAFGQGIGPIWLWEVDCKGTEASIMECGHGAYNRSKCDHSQDVGIKCNIPDLGVKEDIRLVQGRFDTEGRVLINYEGQWGHPCGGDSWDMFDAKVACRQLGLGYAHQPMRSTRFMGRSGYPILMLDVNCTGNERTLSECPHVRSDNGTCMADYKAGILCAGVLPDVLMDVHVLQSSIYMQDWPMYMLQCAAEENCLSSESHVRGARRLLRFASAIMNRGTEEFRPVLGRSSWEWHACHRHYHSMDVFSTYDLLDADGVRVAEGHKASFCLEDVYCDSGAQKVYSCDAGTQGISVNCVDIYRNDIDCQWIDVTGVPQGSYVLRVDVNPNHIVGETDFTNNEVLCDVMLTYRVRIQNCRYADDS

>Ctel_142796

MIIAVGLIVLLASAHGDVPADGSLRLTGGAMETTGNVMVFYQGKWGIVCDDGWSLRAADVVCRSLGYLRALGHTDQAYFGTPNEDVFLDNVRCRGDESSLLECDRNPWHDHDCEKSEAAGVFCAPLPGSLSSPLRVNVSNSTPRITSPHEQHTPTQGPETNSIRMPIRLRGGRNEDEGRVEVRVQGHWGAICGDHWTLLEATVVCKQVGKGHARSAVMSSVFGGEHLAKVLSGIDCMGEEDSLDECSHARRSHDIACPEQDFIAGVVCTHELPDLVPNATLLESSTYLQDKQMYYLTCAMEENCASASAYEIKKTVRDWHIHQRRLMRFSSSTWNFGTADFRPESNKADWEWHLCHMHFHSMEVFAHYDLLDLQHNKVAEGHKASFCLEDVQCLPGKQKKFACKGYGDQGISVGCADDYLHDIDCQWIDITDLKPGTYIFRVHINPNNHVAELSYSNNAVHCDLSYTGLSVTVTNCENGPI

>Cint_ENSCINP00000013061

EGKTENEGRVEIYHGGEWGTICDDDWNIEAANVVCRMLGYDGAWEYLHSGKFGPGEGDILMDNVQCTGDESIISSCQFNGWKNHDCSHHEDAGVKCNKIRLPGFHRNLQVSLYLPANTGDEGLDFIRLKPPRGHRKRLPQLDGYVEFYHQKKWRKVCSTGWDLNTASVICGQLGFPRAEEITDRSQYLARARKKHYYWLSNITCIGTENKVSQCKFQNVDISAYNTCPGEPLITRCIPGPKYARGNFKKGGKRRKHRKHNVLRQETIRLKVGSAEGEGRLEVMRSGRWGTVCHRGWNLWAANVACRELGFGTAKREIINSYFGAGHGPIWLTDLNCRGNETQLSDCQHGYISTSAEGYEDSELTECNHEHDAGVACYVPQFNANQRIRLVGGRNPMEGRVEVKIRKRWGAVCSNDWTMKEAMVVCRQLGLGFALHSLKDVYFFPGTENITEIAMTGIHCKGDELALQFCPSDGRSLSVCGDPHRSSTPFAGVICTELSPDLMQDIPLLQQSLHLEDRPLHNLYCASEEGCLSPSAAKMDWPYGSRRLLRFSTRVWNRGRADFRPARSQDQWIWHQCHGHYHSMAEFTHYDILDLNFTKVAEGHKASFCLEDSECSPGVSPRFDCDQPGGGVQGIAVGCADNYQYNIDCQWIDISDITAGNYLIRIRVNPGTLVAESDFGNNEVICNLQYDGSRVWAWNCH

>Cgig_10017884

MFGILCVCFGLIPLINAVPEFEDGDLRLLGGSTENEGTVLIYHNGRWGSICDRGWDIRDGNVACYQLGFQRALQTLRYSPFGPGRTFRWLTGLRCRGRESRLDQCHPRQWGIERDQRYCSRYSRSAAVVCLPHSTTTSTTTPSTTTTKTTTTTTAKQTTQSTKKSTVASVGLAVNNIIPTRRAIDAIEHNRDKEEEKEEETKTVVERYSNNTIIFDDKDRDDIDNDDQDYESDEEDAEDNNENAIQSTARSRSQPRRSHLQFDAEWSETRRTKDRDVITTASPLKESTSTSRPPTTKEHSRAAAPAPVGPNRYDEEVYSISLPSRRWTKDPLLKCWVLLTYREEILIAKPGQRPSRITKEEYYKKRRAEIIKQRGEETIPNEINRSPLNDASGSRTGNTGNNQIVDRGRVPTTTTKCHIMTTTTTTEAAAPAPVSAYENEVFSVELVTSKWIKDEELNMWVMLSRSGGVSVAKPGQRAVKIDEDEYYQMRREAIIKARDRLKADDPSPGQVPAPSTRQLSFGRGGGTIRSRNEDASSSSNIVISSDLNITREHARGNHHRNRHGHGSKKIETRLGGSRSNRGRLEVRLKGREEWGVVCGDHWTIKEAMVVCRDMNAGYGQQAIKRAVFGGINMKKYFSRVRCKGTEKRLEDCQWEDHEMSIQCTSSDSVAGVICASALPDLEPSIYMLETSSFLQDRHLYYLQCAMEEKCLAPSAYEAQRSRGWRAHTRRLLRFSSVVKNKGTADFRPMLNRDEWEWHACHMHYHSMDVFAHYDILDTNGNRVAEGLKASFCLEDSACDRGVRPKYSCQNYGQQGISVGCSDNYMADIDCQWVDITDLKQGKYVFKVEINPNLIVAEISYDNNVVVCDLNYTGYYARIYNCKHEGLL

>Hmag_XP_002156753

MSILFLLVAFSSVLLNTCVKLETSRLRLSGSNNSYEGNVEVFLDGKWKYVCDDSWDLRDAKVVCRQLGFTKAISATVKSAFLVGINVSYWLDDVLCWGSESDLLHCWHRGIGSHNCNDLKEIAGVICSTVIENDKVKPSENKSEKFNIRLNGNVVEGFISSGYLEVEYDGKWGLFYTQSWTYENGHVSCGNLGYSAVEHDNVQHDNVLRGFYMNSSDHFNIWQANITCVGTESTLYECKHGFWKNYSDSEFYPVYLTCKRSKVVKNQRFDVEFIEGQTVRLRAGVVGSEGRVEVKHKNQWGSICDDGWNIVNANVVCRQLGFGTALEATHYGSFGQGTGKIWLDQVKCNGTESDIKSCVHLGWGVGDCGHQEDAGVKCHFPLNQKNLKVRVIGGPHKRIGRVEIFTDGEWGGICGMKWSIREAEVTCRQAGLGYAKRGFTSSEYGLQRRLVMFNVMCSGDEMALNQCFYKGYGKGRCRFYDMASVECSETAPDLVMDFKELEDSIHVESKFLLELSCAYEENCLSSSAARFTYYPELYSRTLLRFTSLFWNRGTANFLPSTAKRDWIWHECHAHYHSLERFSDYDLTDLKTGLKVAEGHKASFCLEDSKCEGGVKKVFNCTNRGDQGISVNCGDVYKSNIDCQWIDITDIKYGKYKLRVILNPLRNVVESDYSNNIVTCEIDFLSQSKVNVTSKCVIDGCERMSHGGTGDGACCKFPFVYKNRQYNHCTTDGFKENVLWCATTSNYDKDKLWGLC

>Hmag_XP_002159926

SEKFNIRLNGNVVEGFISSGYLEVEYDGKWGLFYTQSWTYENGHVSCGNLGYSAVEHDNVQHDNVLRGFYMNSSDHFNIWQANITCVGTESTLYECKHGFWKNYSDSEFYPVYLTCKRSKVVKNQRFDVEFIEGQTVRLRAGVVGSEGRVEVKHKNQWGSICDDGWNIVNANVVCRQLGFGTXIWLDQVKCNGTESDIKNCVHLGWGVGDCGHQEDAGVKCHFPLNQKNLKVRVIGGPHKRIGRVEIFTDGEWGGICGMKWSIREAEVTCRQAGLGYAKRGFTSSEYGLQRRLVMFNVMCSGDEMALNQCFYKGYGKGRCRFYDMASVECTETAPDLVMDFKELEDSIHVESKFLLELSCAYEENCLSSSAARFTYYPELYSRTLLRFTSLFWNRGTANFLPSTAKRDWIWHECHAHYHSLERFSDYDLTDYSKSRNFCSHQCSQAGFGSISAAILQSDFYYPVVFHQVKLWSYMFQMRLFSKKDTAGDIQGHLSNLNVRQLF

>Hsap_ENSP00000261921

MALARGSRQLGALVWGACLCVLVHGQQAQPGQGSDPARWRQLIQWENNGQVYSLLNSGSEYVPAGPQRSESSSRVLLAGAPQAQQRRSHGSPRRRQAPSLPLPGRVGSDTVRGQARHPFGFGQVPDNWREVAVGDSTGMARARTSVSQQRHGGSASSVSASAFASTYRQQPSYPQQFPYPQAPFVSQYENYDPASRTYDQGFVYYRPAGGGVGAGAAAVASAGVIYPYQPRARYEEYGGGEELPEYPPQGFYPAPERPYVPPPPPPPDGLDRRYSHSLYSEGTPGFEQAYPDPGPEAAQAHGGDPRLGWYPPYANPPPEAYGPPRALEPPYLPVRSSDTPPPGGERNGAQQGRLSVGSVYRPNQNGRGLPDLVPDPNYVQASTYVQRAHLYSLRCAAEEKCLASTAYAPEATDYDVRVLLRFPQRVKNQGTADFLPNRPRHTWEWHSCHQHYHSMDEFSHYDLLDAATGKKVAEGHKASFCLEDSTCDFGNLKRYACTSHTQGLSPGCYDTYNADIDCQWIDITDVQPGNYILKVHVNPKYIVLESDFTNNVVRCNIHYTGRYVSATNCKIVQS

>Hsap_ENSP00000231004

MRFAWTVLLLGPLQLCALVHCAPPAAGQQQPPREPPAAPGAWRQQIQWENNGQVFSLLSLGSQYQPQRRRDPGAAVPGAANASAQQPRTPILLIRDNRTAAARTRTAGSSGVTAGRPRPTARHWFQAGYSTSRAREAGASRAENQTAPGEVPALSNLRPPSRVDGMVGDDPYNPYKYSDDNPYYNYYDTYERPRPGGRYRPGYGTGYFQYGLPDLVADPYYIQASTYVQKMSMYNLRCAAEENCLASTAYRADVRDYDHRVLLRFPQRVKNQGTSDFLPSRPRYSWEWHSCHQHYHSMDEFSHYDLLDANTQRRVAEGHKASFCLEDTSCDYGYHRRFACTAHTQGLSPGCYDTYGADIDCQWIDITDVKPGNYILKVSVNPSYLVPESDYTNNVVRCDIRYTGHHAYASGCTISPY

>Hmag_XP_002160127

MHTYLFIVFLSGQFSFGSFLYIRLVNSEGSAGYHEGYLQFNFHREWKYLCDTFWDFRNSKVVCRELGFAKAVLRNRTSYDNVQQSKFHHFMVHCDGNEKSITQCPLKNVESYVFDKKCYYSSQVFIYCSETRDNDVTISLNKKYELRLWNNSSKSKKLSLQGELQYFNGDSWVNVCSSGWNDENSFVACSTLGFTKHESKNQIRLRGGYRAGEGRVEIFNGTTWGTICDDGWDLIDASVVCKELGFGSAAEASHWADHGEGLGAILATDIQCSGSERYLHQCIQSKNTSLCSHSEDAGVRCNVPHIVYEDVRLIGGINNFEGRIEVTFDGSTWGGICGIGFSEREGKVLCRQLSLGYVSWASKSYKFGINYKKLMYNVKCMGNENSIKDCSYKTFGSCRYYDMASVICTKFETKGYDNMDSQTSESLVFKESGQPAKRTMLNSYFDTISKDEIDKIDKSYARTFFHTGVPFALPDSSAWKQHHANLRPAYKPPSSKLTDGFSNINVNHLVSYSIHVENRTMKPIAYKIEPTGQEQQTGINIAKRIENVILEIGVDKVTTIVTDNASNMRAAWDIIEKKYPKIFCNGCAAHTINLLVKDICLLPEFVDILQKSPKLTAFVKQRTSLINQFHYLNIVQDNFWEECQRFTNVMKPLSKLVGKLESDSCLLSEVYIEFVNLMEIWKEDAVLKTLVLSRWEFIHTPSMGFAYFLDPRNHGGRNMYTESPPSSKSDLFIVLELLPKLCANPTPDFVTQIKLMDPDIWWGVEGASKFPNLAKVAQIVFTIPTSQAANKAIGLVLMYANATQQKEANIADIMLGNTSDHDESDESDEDGVNVDFTLETSVDTNVPDLVMDISTFEQSIKVESHIMSQLKCSFEEKCLSPIVDNSYFLNRNIKRKLLKFTSRFLNKGTVAFRPNVHKENWKWHKCHQHYHSQETFAVYDLIDQDGHPVSEGHKASFCLEDSDCEPGYKQVYNCTDKNDQGVSVNCADNYKNTLDCQWIDITEVSEGNYSLRVFVNPLELVTESDHLNNRAVCVVHYYNNVVEVKECSTEPCYQESFGGNSFGHCCHFPFTYNGQLHYNCIRNDDHRNKLWCGTTPDVDKDVAKTILRSYVNSGNNLLTAKDIHKAMYYSFGAKDVTVAVAQISNGKAVVNGPKIKNISNYHSFEFGEKIMKMWCYFDIGEGIEQEYGYLKIQLSIKLLLPYSKTDNSIKSNKSLKKKQKRSDKQLCSLGFCTEINCTLSFENDAELEEHMLSGLNTVPKS

>Dmel_FBpp0085171

MASFRFQLLQLLVVLSQGWANLNVQNNYRNMMVRLATNKAALAGIQVLREGRVEVSFDFGASWGTICSTSWSMREANVVCRQLGLGYASKASQGTEHGDSRKYPWGMVGTLCRGTERRLADCIRESHYPNLCNARNHNVSIAACVSHSADLEIGLVDIERTARLEAVPMSRLTCAMEEHCVSADAYEIRRTNPHAARILLRFSVKASNVGTADVSPYANYKEWVWHQCHRHYHSMNVFATFDVYDLNYRKVAQGHKASFCLMDSECRPGVRQKYTCGNTTQGISVGCADTYTDVLDCQWVDVTRVPINRRYILRVALNPEYKLGEISFENNGAECLLDYTGVRQTTRIFNCRRKPLWFKI

>Ocar_g7137_t1

MPCGKLISRLDFASSAARLDALKLSDDGKLCLVTDEALEVLLSSSESPCQLAFRTAAWSPVGCDSVGGSSDCLMRQAADISIVPTEESNSVITLPCCSFACQLQYSGMGQKVKKEKEKIDSILYEYRMDLLASVAISWSPCVSDSQSVLLAIATRNQIPVHVLKWTAATLSADEVLVAVKNSAFLQISYDVEKETASLDEKPDFRVLRGLHGMSVSGVAETDAGIWFTTSLDGRVGTFGLTDDLNSVTLTGNFDCLVSAFHGCAVSPNGLFLYLVAYPNLPTGFKRMNVHMKSQVWNKQPAISPLTEEEKDIVDKIEAICNHVEDFNECGGEASRGVRIVGPTEGAGRVEVLVKGQWGTVCDDGFDLIDAGVVCRLLGFESAIEAVPNSWYGARGPAVIVPAHEXXXXXXXXXXXXSDPKEHEWSESRDFGVRLKGSSKPRTGYVEVGFGGRWGTVCPDGWGSREARVVCGQMGYRKGRPRLYVGSKSWNKSSKKPITTTSTNVTENALMADINCNGLEINLALCSHAGFGRHRCRSPGIAGVLCWRPSKSLGTLKHQLWGDKQVRLRGSALPWEGRVEVFWNNEWHKVCDRGWDVQDASVVCRQLGYGSAYEAVTRSRGFFGQGFTRILMNNVRCKGNEPKLLDCQYDDTFRQSCRHWENAGVRCNPPPVNHIPARLIGGGENDGRVEMLQSTSSRTRWVAVCGDGWNEEAAAVVCRQLRLGFALENGKGGKYGNGVEYLVMNVLCNGDEDSLEHCQQGGVASVLNLADLIPNFKSTEDSLNEHSVSFVRLSELTCAWEENCLSKSAAQYMAEGQHMEEARQFHSRRLLRFSVQVFNFGTRDFLPIVDRKDWIWHSCHRHYHSFENFATYDLINSSSGKSVAEGHKASFCLEDGHCVEGGQKEYLCADNHQGISVNCADTYNFNIDCQWIDISDVSYGNYTLVVTVNPSHLVAEMDYSNNVLKCSMQFYPTAVHQEGTMHKVQCQMG

>Ocar_g7139_t1

MRGIKCIGNESSLMECHLPPPKHDPSGCKHFMDSSVECYKPETSNKIVRLSSFSRETDAIPAKHGFVEVRKNVDGPWFPICDVSWGHKEAQVVCRQLELGYAEAARQRPYNKELVKYFQADYTCEGNEVDLLELPDLLPDVEALRFSLRQQVIAVRLSSYQCAIEEKCFSASAYNRTDDPLRYLIRFTTRIMNLGRADFEPHANRDEWHWHQCHNHYHSFEAFSFYEVINSAGVRQVAGHKASFCLEDSDCLENGPPPRYYYYCSTGHAGRGGHQGISANCADSYFSHLDCQWVDVTGLAHGNYRLVVAVNPHRKVAEMDFENNAVFCEFTYSGHGFLKLGECGFMPDFEQLF

>Emul_000217900

MNAILSLLLLLSETTLVDVSAGAIKKPVDGDLKLLDGDNFASGTVAVYRGYAWGRVCDDNWSIREANVVCRQLGLGFAVRALKRNKFHSVSGRNYFMDNVRCLGNETRLIDCKFDGWARHDCAEHEDAGVQCAQDTSPRAKISWNPLRLDYTREELEKLAKVSFTLKNRGTSGVYQVKELNTTQAVDDAQILIIQPKDGEAGALCPDDFTTMDAIVACKQTNSGIGGRIAEIPLASDVFPAMKHVAIIGHCFGNETSLDQCKHYVDPNGVKCKSTKAVAVACQDKLPDLTSDIEQLENSVHIQRLRLWHLQCALEEHCFPDSVYTYIANNPGRYYWDARTLIRFSSITKNVGTAPFLPALIPEHWEWHPCHAHYHSMKVFGSYEVIDIMERLVSYGHKASFCLEDNHCDKNVTKHFFCSNVMDIKGEQGISPGCQDEYFFNYDCQWVDITDLPVGDYTYQVTYNPHYLVPESNYFNNAVTCKMQYRGNWGRFYGCKIVHPFELL

>Cint_ENSCINP00000007858

MATSMRRFVSYSFVLVCLGTFSLVHSQSIVDVRLFPLTNSYPVLPLSSGALDVQINELSSYSWLYVCYDDWSDANSFVACGNLGYPGYTSHSPIAPLSPTALLISCTGTESSLDECQHVTTSTCRSGHTYVSCSLRDNQVQRYIRLRAIGLTGVGRVQIAVNSVWGSICGHNWDRASANVACRQLGYGTAREHYSGVQFGQGHGTITVSSINCHGNETNLEQCQYELIPLMSDYTNYSMDGCSQRNLAGVSCNTPNYGHQESIALVDSPAGQPSVGRVVVRVGSVWGSICSNRWTIREAAVVCRQLGLGYAHSSLQDVWQMTASGQGLRTVATNVVCTGNEDALSECEYFINYDPFGADCGSGWTTTAAYVGVVCSNELPDLQLDMADLTNSMWVDVLHMNYLRCAAEERCLSTSGYYPSNSNSTRKLLRFTTSVWNRGTAAFRPWLQSNAWEWHACHGHFHSMAEFAHFDILSIDTFERAAEGHKASFCLEDSVCQEGVSKRYRCTTETTPTPQGISAGCADTYFAQYDCQWIDVTDVSNGEYYLRVNLNPLESVGELDFDNNHNVCTITLNDRSVVVNRCFIPSDQRETS

>Aque_XP_003387024

MLCFQFLFFLILSSLVVNGQKPGDVRLVGSTLPFEGRLEVFFFGAWGTVCDDRFTIVDGAVACRQLGYNDGAERTETTGLASSRFSPGTGDILMDGLECKGDEKKLGDCPFNGWGLNDCVHYEDVAVICKFSKPSLPKPNVTSVRIACPESDYGLGTCINTCSLPSTTCNNETEVAQIGIVERLVNNSWYPIPSSDWDQASAQVVCNQLGYPRAGPIPSIDEIFPRRRCGRPRNAARCAAIRNFNDRLSKTVTEGLACIGSETSIDDCIITSYAVKQSSALNVATIQCLHDSTRIADCENYNYTNSSRELYRLRGGPVPWKGRVEVKVGGVWGSICDLSWDVKDANVVCRNLGFGYAEAVFTRSQFGRSSATTHFSGLRCNGNEDILSDCSSNTGGLLRRDAHSYCVFHSGDVGVECGMPACNKEPEVRFATTGSETMIEVNGPLGWGRLCFDNKLKYTEAHVLCRDISGELAASFGPITDLSYLGLSYSAQYNCTGDELHSSECRESITRTFCFDYTSIECAQGLPDLVPMVDQFERSLRSYPYVHLIPMYYLTCALEEQCLSSSAYRHADNLYHIRRLLRFDSLTMNYGTSQFLPSLPSSEWEWHTCHQHYHSFEAFISYDVLNKDGEKVAEGHKASFCLEDSTCRGLGAYMYYRCSTGNQGISKNCGDLYGSHLDCQWIDITDLSPGEYIVRQIVNEEAGVGESDYRNNVIQCEVEYMSNLYQLKVGKCSHSDHI

>Aque_XP_003387025

MLPLGSNTLNSGLLLLKAVAFCLVIQCVKTQDRPSDGTIRLQGTLPYQGRLEVYYRGSWGTVCDDRFQKPDADVVCRELGFSGGSDRVITTGFSNEFASGTGEIWMDGVSCRGSEEKLSQCSFNGWGIHDCRHYEDVAIICKYTAPTLPPSTSLPTLSDNNVRVTCPGPNYRLGECNNCSTYTSACRAPDPRRPAIIGVVEMLIDGKWYPIPRKNWNVNAAKVVCGQLGYPRAGPSPNINRIFPTKSCSNSHTSAQCDSINDFNDRLSRTVTEGLTCTGGENKLNDCYFQSYNINPSSPASVATVQCYFDDLRTEKCKDRNQNNKELYRLRGGPVPWKGRVEVKVGGVWGSICDLFWDLNDADIVCRNLGFGTAKKVETRSFYGRASDSVHFSGLKCTGTEQMLSQCTRTTGAQLPREINSYCYQHSGDAAVECNIPQFCPKEKEVRFTGTGVDKFTEVKGPSGWGRLCFSSISTYNEAHVMCREVSDELAARYIPIKDPQYRGRSYVVEFKCTGDEISTSECRRTFRTSSCINYTRIDCTAGMPDLVPNLKKFQSSLESFAYTSPTPMFYLSCALEENCLSSSAHQHANNPSHIRRLLRFDSLTMNYGTATFLPNLEPHEWEWHACHQHYHSFEAFIHYDVLNRAGEKVAEGHKASFCLEDSMCDFGGYSSYRCSTGRQGISVNCGDLYARHLDCQWIDITDLPINRNYIVRQIVNADNLVGESDSKNNVIQCEIFFRGDSFPFIVSNCTHSDH

>Acas_g273

MRRHHAVGLSAVLLLALIAQSARTQSCTGRAVRLDGSNWLDAGVPNVWNVPQTYTLEGWIYPTASTAYTGVFGNVWDSGSTESGIGFQLLSGGIVFLHRAPFDYTFTGSAPLNRWTHVAGTYNAASGQAALYINGALVATRGGGARQVNYSRQRNFMIGRFVDDNEDIRFVGQIAEVRLWNVVRTATQISQSWKVPIGQATNLVASWRMTQMDVLQATTTGPVLDLSGNGRNANLGGTPAFTTYTCTSADCTAYSSCVPAQVASVSPSPVTRRLPDITLDAVRLKNSMEIITRTFDSASCAVNEGCVEGTGVRTLLRFDTATPNRGTADLVLGDPSGNPKFHYDECHRHYHYEGYARQVWVNSNGFLSWQEASGGPTAHEENTPNLPFPNAISVLWDDHDPTRCGRVYYTIKGTAGSRRLIVTWYQVCHYGGATNDVVTFQVQLQEGSGKISMFYPDTATTTSAYTNGASATVGLGAILPSDSILYSYHASPPVRNGASVVFTVQTEQINAAPLASINGPYQGTVNEPIKFDSRGSKDPDSRMLEYLWIFGDNTVGQTTAFPEHTFAQAGEYDVSLVVYDNFQEISKALISNMVHAYREQESPQAAYKRQDHNIFFISAHTGQTHSQQASQLAVTTTMRYHHLVGLSAALLLALIAPCARSQSCSGNAVLLDGSNWLDAGPPSAWNVPQTYTLEGWINPTEGSDWVGVFGNLWDSGATESGIGFQVLAVWRACGVPPSRTVRLHLHRLGALNRWTHVAGTYDAASGQAALYINGQPVATRGGGAQQVNYNQQRNFMIGRFVDDNEDSKFVGQIAEVRLWSVARSASQISQTWKVPIRQASNLVVSWRMAQSGNWVDDESGNGHAAGFGESPQFRTYGCSSADCSAYSSCVPTQSDPGGRLLPDITLDADRLQRSMEIITRTFDSTSCAVNEGCVEGTGPRTLLRFDTATPNRGTADLVLGDPSGDPRFHYDSCHRHYHYEGYARYLIYDDSGTQVGRGRKMAYCLLDSEQDSGYTGPGPVRDSAQYSCSNQGISVGWLDVYHKDLDCQWIDITNLSPGDYYMNIDLNVDHLFEELDYSNNAFNISFTVEQPCYPKPRYATDTSGSNYAYEDPTTNGHTAADPSDDGSFSIDLPWSFPYFCKSYSEVWVNSNGFLSWQEASGAPTAHEENTPNLPFPNVRHSKDESVDADVAAAARLAESRRLEAEGRRLGEEEGRWDAAFDAWAEAAALTPDRAGPHEGRARAALLLAQPRPRVALRAAGQAACLDPTPFLTLGRAQMACDEPVLALRSFNTVLRLEPTHATLHEDMIRAEKLVHQLHIRAKELEEQRLEQEAERIQSELKRKRDRRKAAAERWYADHRERDEAGKRGAVSAVVGDEAEGDEEVENEEEDEEEEEDELEEGYSREEWAQLRRVLATQGGDLLHDFQRELARKRQQDREERLREREEEVEEEEEEEDDNDDDDKKK

>Acas_g15054

MAYCLLDSAQDPGYTGPGPVRSSSQFSCGNQGISIGWLDVYHKNLDCQWIDITNLPSGDYYMNIELNVDRLFEELDYSNNAFNVTFSVEQPCLPKPRYATDTTAANYAYENPTTNGHTAATRTDDGSFAINLPWSFQYFCKSYSCRTPLAVCQVWVNSNGFLSWQEASGGPTAHEENTPNLPFPNAISVLWDDHDPTRCGRVYYTIKGTAGSRRLIVT

>Acas_g10187

MMRLSFLLLACFAIASLLVPSGQAFNGGCEGYAVRFDGTNWLDAGHGAWSQPKDYTLEFWIKPYLTQPNALAVLGNFLRMERQGFGFGFLMEGDRIIFMDSQPFDGQLSASIPFNEWTHVTGTYGADGSALYINGRRVAHRPGRAALRYYTTDGFLLGRTLGKEAGFVGELAEVRLWDRQRAESEVAEDWNRPVSASSTGLVAYWAMRTSGDFFIVDETDRAQASATLAGPSRLPLISRACVTGCSNESRCVPAQGSINKRQSSALPDITVDANRAQSTMYIEQRSFLSTDCAVREGCAAAGTRVLLRFDTATPNIGRADLTLGNPAAGGGFVYDACHRHYHFSGYANYRLYSYDRTLTRVGRKQAFCLEDFEPISGWTGSRSLLPKFDCNNQGISVGWQDVYVANLDCQWIDITGITLGSYWLYVEINPVVNGARAFTESDYTNNVFWIPFRIVNP

>Acas_g16456

QSSALPDITVDANRAQSTMYIEQRSFLSTDCAVREGCAAAGTRVLLRFDTATPNIGQADLTLGNPAAGGGFVYDACHRHYHFSGYANYRLYSYNRTLTRVGRKQAFCLEDFEPISGWTGTLDYHHGAQ

>Sphaerotilus_natans_subsp_natans_DSM_6575_633286379

MTALTALRSGPLLARILLAAALALPGLTVTVPAVAQTASVPQALASGTAVGGIAGAYGSQRHYTIEVPAGASTLSISIVGSSGDADLYVRRGAPPTLSTWDYRPYRSGSNEAVSVSAPAAGTWHVMLRGYSAYSGLTLKATHDGGGGSAATVAAPTFSPAPGTHSGRVSVSLASATPDAVIRYTLDGSTPSTGSEVYTAPILVTATTQVRAAAFAGSQSVSSVSAGTYTIINPVQTLAIGASMANLAGAQGSVTNFRVAVPAGVSSVSFSISGGSGDADLHVKYGQLATTSTWDQRPYLSGNAETVTIATPRAGDYFLMLHGYRAYSGVTLKATQSGTASAGRPDITISMAAANPRITTETFAANACEIEEGTITAGTHRLLRFNTQTRNIGSADLVLGNPASNSSFEWGGCHGHYHFRSFAQYRLLDASGAVVRTGKKVGFCLMDITRIDSGANPSARYTCSNQGIQAGWADVYSSNLSGQWVDITGVPAGNYVLEIVMDPMNLIDELDESNNTGRLNVTIP

>Cmer_CMN144C

MGCVLGTGPRKLLKLAGAIGNNGPDDFVVGNPADHPNIYTWDTCTGQFITRKPMLNYSIWDSAGNLVRSGKKESFCMEDLARFPEYTGNPALVPAEPVHTCTFQGISVGWCDIYDDSLPCQWIDITGVPSGHYTLRLECNAYHIFKETTYANNVVETPVTIT

>Stigmatella_aurantiaca_488687725

MRKALMFWAGLVLLMTGCDDDATYVAAGEVPGTALHVPPDGPGEVQLRMKNEGSATWKPDQVKLALREQQGWSGGPLVLTEQVKPGQVATFRGNITAPAQAGLHKLGWVPQRKGTAFEKAFETDVEVTCSNGEFCDGEERLANGRCVSGPPPCDDGAACTEDVCDPDKRTCQHIPIGSCAVCMATCNPDCSGKLCGEDGCGGQCGTCPAGQACAQGIFECRPDSQAGTCRNPLPLVADGTPLVGDHIIQGDTSNGFHQLIPSCNRTSTAVEAVYTFTTAEKLGLEARVSGYDTVLHLRKKRGADGTADCLDNTAARTVACSDDSSPPGDYGSRVSVSLDPGTYYLIVDGFDSTQAGPFTLSARFAANGCVPKCDGLYCGGSDGCGGNCGVCTGDESCVKGRCLPNPCIPNCDGKACGDNGCGGQCGFCPNDELCVPATGTCETFAACDHLRPSCTPSCGTSEFCGTDCACHPVSKQLPDLIVDEARLRDEILFDTIFVTENSCARVEECVEGTGERRVLRFSVEAVNQGSATLTVPAPAERPDLFTFSPCHGHYHFSGFATYALVDADGRTVLAGRKQAYCMEDTQRVATGPDVPCAKKFTCDDQGIQRGWSDLYGNTLDCQWLDITDVPPGDYRLQVTLNPSRAFQETTLDNNTSSVPVTLPAR

>Chondromyces_apiculatus_DSM_436_599570991

MRKAIATLLLGALIGVGCSGEQDNLFNNGTGGSGASGVGGDGNTGGQGGQGAGPSEGCFGGCSGDTPVCVDGDHCADLCPGGREACNAANDPDAPPVCCPEGQQCCEAQIFGYTGADLCRPSGEACPIGCPGGDIACPLHEYCQLNPQLEDYSCVMGCPLTSVCGFNLCCPLGSSCLNGECILPDLTIDQARVASTLSFSQVNADSDPCLVSEDCLAGPGLRNVLRFSSRTQNVGAADFVIGSPNGNPDFHYDQCHGHYHYQQYAEYRLIDPSNNNVVLTGKKQGFSIIDMGRIDPDDPNTPTTSKYNGAFQGIQRGWWDEYGAGLQCQWIDVTGVPAGDYILEVEVNPERRIGETSYDNNVASVPVTLPDPTCLGVDCSFLDSDCTQGVCEAGQGCVEVNVNEGGACEDGQFCTTGETCQAGACGGGTPRLCAPPNGCYAATCDENADTCSAVPANDGAACDDGSPCTDGTTCANGACLGGAPANDGMTCDDGASCTTNTICTAGVCGGGDGPTVYFSEDFSDNSQGWVLGPEWEIGPAQASTGAHGGANDPANDHTDTADNGVAGVNIGGFAAKIDHAYYYLESPPFNTAVASGPVVLGYQRWLNSDFIPYMSNTVEVWNGTGWISLWTSGPTITDATIGWNYHQFDLTPYKNAGMKVRFGFMVSGAQVLWDVGSWNLDDILVASLACP

>Spun_SPPG_08352

MGNSFPLAKMRCLPILLGAFCILFAPVYGQNSSIAGPGRNATVAGPGDTLPDFFPDADHLQKHLYIDYVDAAEDPCLINEGCLTGNGTRMLLRFPSMVHNNGTADAFLGKPPADRDDPNNPPYWHWDTCHEHWHFTAYANYRLLSADRSTVILNGHKNGFCLEDVGCDEDGLEPFYNCTNQGVTMGCHDLYDETLPCQWIDITDLHLQANYTPETEYTLEIVINEEGFFPEMSTANNAAYSSVVIGNVPPYTGPSLAEVQAPNGPGSRGNRRPNNNESEAGSGDAAGTEDGRGNSNGMRGAGSGASASRNAESVTAAASASTSASAASATSTMSPSRVRHRRWLTKWW

>Gpro_36873

MVHRMYTPLTLPSRRLTVKSREGLGATSSSPPSGPGPPPPGMNPAPFGAIMKCVIASSTPARPATRTTRGRSAVRTFDTTECAIRSGMNIPGRRIGAAGRLSSFVLPPAQIVPTGEEIWAALKVMGFSHLSTLPLQLTLPPSLGTMISPRRAGWSVRSLPGLLAVAVSMALTAAVRSDGPSPTVAPAYTQTSQQGRTPTAAFTVPVTGPLPDFQPDADWLAKTLHIDHIDGTKDPCLIKEGCVDGPGNRTVLRFDTLVHNNGPGPAHIGEPPASPNSSNIPPWYYYDTCHHHWHFIAYANYQVLNVNGSEVVAQGHKNGFCLMDSLCPPGVANPYDCNDQGIHAQCADLYDAGLGCQWIDITHLPTLPGYSPNTEYLISVILNPEKAIPETDYSNNRAVVKFKISQLAVKATNAWDLPVGADPVSQAAFAAHSRNDPIGSLSRKFGVVNKNGDDLYLMKLHHEERISSLGQLNSGSPTDLDLGGLAGRVGVASRGGGSNGVVRGKRGCQRR

>marine_metagenome_134951750

MESAQMLVLLEEGVTYWVRFADLDGGCSGFEWTLTYDGPPEGCTDETACNYNPSAEVDNGSCIYPGDPLCTGPDLMVVESAIESSLQAETMMVDENNCYIVEGCLNGYGERELVRFTTHIKNIGDLDYYIGTTAQTDSTEQFEWGDCHNHWHYKGYAKYD

>marine_metagenome_138700853

ICCAYGQGAYTLYLEGVAVATGGDYGQQDQVQFDCAPGATCNDAVVLSDADYGLVTQAEDNFWYTFTPPANGMYQFSSCGAACNTTLYIYEYCNMGNFDDTNEGSIYYDDNQGGCGEEAALTILLEGGVQYWVRFASLDGSCSGFDWTFDYAGQPTGCTDSGACNYSPAAEVDDGSCVYDGDPSCTGPDLIVLADVVSSSLSTTTMNVSQTDCYIEEGCLNGFGTRELIRFTTHIKNIGEIDYYIGTTSQANQTGQFEWGECHNHWHYKGYAKYDLFTMDGALIPIGF

>marine_metagenome_135033467

MYTMILLPSRALWSLYVFSMFLGVVGLNQASAQCSESESALLVEVITDGYPSEISWEVVMGGEVVLSGGPYAEGGTMYADTLCFPSSDEPCIQFEIFDSFGDGIFAPGGYTVYLDGQTVASGGDYGDSDGALFACAPGETCNDAIALTDSDYGTVASPGDSYWYTFTPASNGMYSFSSCGNGCDTRLYIYDYCQMGNFDDTNEGSIYYDDNQGGCGEEAQLTVLLEAGVTYWIRWASFDGPCAEAWGWEFGFAGPPVGCMDPEACNYNPMAEEDNGSCIYPGDPACNGPDLVVVEEAIINSLQTQVMQVSESDCYIDEGCLNGYGDRELVRFTTHIKNIGDLDYYIGVPSDDGNNQFEWGDCHGHWHHNGYAKYDLFDLNGDLIPIGFKNGFCVMDLECSDGGTYQYGCGNMGISAGCGDIYSSGLSCQWIDVTDVEDGTYYLVVRANYEFIPDALGREEMDYDNNHAAV

>marine_metagenome_138569011

VYDGDPSCTGPDLIVLADVVSSSLYTTTMNVSQTDWYIEEGCLNGFGTRELIRFTTHIKNIGEIDYYIGTTSQANQTGQFEWGECHNHWHYKGYAKYDLFTMDGALIPIGFKNGFCVMDLECSDGGSATYGCSNMGIAAGCGDIYSSGLSCQWIDVTDVEDGQYRLVVRVNWDYDPDALGR

>marine_metagenome_136914123

MAELDNGSCVYPGDPECNGPDLVVVEEAIINSLSTEVMQVNESDCYIDEGCLNGYGARELVRFTTHIKNIGDLDYYIGVPTQEGNNQFEWGDCHGHWHHNGYAKYDLFDLDGGLIPIGFKNGFCVMDLECSGGGTGQYGCGNMGISAGCGDIYGAGLSCQWIDVTNVEDGTYYLVVRANYEFIPDALGRAENSYENNHAAVCIQLDRSSGALVVDHVDGCEPFYDCNGVLYGTADTDCNGECGGTALVGDLDNNDAQEFADVVAYVEGILGNDLAPSTCTDIDQDGEITVSDA

>marine_metagenome_136902050

LTLWGCPIGSSCDDPLPMSIGADDAPLDLIAPMASSWYLLNVDTTGQYRFSTCDLTGCDTRLHLYDYCDMAVFETASEAFITMSDDDCGLQSQVTPVLVSGQTVYIHVEGDGDCNGGATGVPFVAKYLGGIPGCMDIEACNYLPIATTPDTCYLSGDPECPNIGPDLIINGPRAYSTLEMTTENSSDGCMIEEGCIQGYGTREIVRFDTEIANIGTEDYFIGAPSAQPDQFEWDACHNHYHYEGYAEYALYTSGGDPLPTIGFKNGFCVLDLGSCNYGGGPQKYTCSNMGITAGCQDI

>Corallococcus_coralloides_504205840

MRIRGALTCAVMLSLSAGCKDDPKPQPDAGVPDAGADAGSQDDAGTAGPTLSETPRWEVEGDGLEPKECFGRGVALGDVNGDGRRDLVVISPPCTSAPTNPGRVMVYPGEGAYFSKTPVTSKLSWVHPSPRTSGYQMVVATGDVDGDAYADVLVKSYYGVSVFKGGPDLSQVFAQPLFRAPDSSIVRFHSARLLDLDGDGLDDLVVTTFSGSITLYRATPGGAEGPFTNVRVLSGYASPAGDTDGDGTQDLLVSLLDEPSGQGLFLGCKADSTRACEGPLTTQPVWKGTAESMRGIPDLNGDGRPEVLAGLRGSMRLHLSDASVQGYSATPVWTLMDDPAFPNLAAQSATVGSMAEGGTGHDFVLVSLGRVYLFRPTQDVSGPLEPVWSWPRANHLLPQTMLGFTLPSVASPGDLDGDGYDDLVVGLSQEHDGTRAPGRVRVYGGGVVPDSQEPAPALMPTKTCNLQVDPVNGKPDLTVDRDVIARSLYIERRAFAADSCEVREGCVPAGGERRLLRFTTSIMNMGTAPAVVPSPQERPDLFVYDECHQHDHLVNFAGYDLKDASGNSLSVGRKQGFYMVDYTQYCADGTPFSWYDPGTGISPGWSDVYTADTACQWLDVTDTPDGDYTVRVGVDENHIIDELDALPNEATVKVRLKGDTVTVLP

>Corallococcus_coralloides_504205841

MEGRPFIPRGNNTMRMRFARSCAVVGLLVLTGCKDDPKPQPDAGTPDAGSTLSETPRWQVTGDGANPRECFGRTVALGDVNGDGHKDLLVTSAPCASFQRDPGRVMVYAGEARDFSKTPVTTTLTWVHPSPLASSYKMTVSTGDIDGDAYADVLVATSFGVSVFKGGPDLSQVLTQPVFRAPDASTLRFSGAQLLDLDGDGRDELTVSTTPNRNLTVYRATPGAPEGAFTAVRTLSGVPVPAGDADGDGVQDLAVTHTDATVDLYLGCKAGSARVCEGPLTAQPVWTGQAESFAALPDLNGDGRSEALLLLNGSQRLHLSDAAGPGYAPQITWQVMDDAAFPLFGQSFLTFSQTVVPVGSMVEGGTGHDFAIGAIGRAYLFRPTANVSGPLEPVWAWPRANRLDLRTSMGTDYLGMASAGDLDGDGYDDLVVGVSSGLSGTPAGGDGTPAPGRVMVFGGGAVPDSEEPAPALAPTKTCNLQVDPVNGKPDLTVDRDVLARTLYIDRRSFTADSCEVREGCVPAGGERRLLRFTTSIMNMGTAPLVVPSPEERPDLFVYDECHDHHHLTNFAGYDLKDAAGNSLSVGRKQGFYMIDFTQHCADGTPFSVYDPGTGISPGWSDVYTADLPCQWLDVTDTPDGEYTVRVGVDENHIVDEADTLPNEVTVKVRLTGDTVTVLP

>Myxococcus_sp_contaminant_ex_DSM_436_488713852

MHRTRALALTALLAVSLCACDDPDDPGPSDAGSLDAGSGDGGEPDGGAPDASSGDAGPFLSEEPLWEVRHDPAYAQGCFGRAIALGDVNGDGQRDLIVAAPPCLTTTRDPGQVSIFAGEAPYFSTQGVTTVMDWHNTNSRTSGNRMVVFTGDVDGDGHADVLVSGQYGALVFKGQADLSAVFAAPAFVVPGGGIYNNAVFADVNGDGLDDVVSVRGTQVSVFLATPNAPGGPFTLAERSQPLYATAVRRAGDYDGDGAQDLLLLRGSVIADALTLVRGCKADSTEPCDGALMTQPLWDVNTLSLSLVPDMNGDGVPERFTGPGGGTVRLHLSEAGGYSETPIWSAMGDPVFAGFGSTIVPVRDMDGDGAGHDFVVSADGRLYLYTPEQGVSADLRPVWAWPRAERLPPNYEGFNRFTVLAPGDLNGDGYADLVAGVAPPVGLVDGPAGRVVILGGGKVPASPASPPSLPTVAGCGLTVDPEHGLPDLAVDADVLARSLYVERRTFAADSCEMLEGCVAGPGERRLLRFSTSIQNFGSAAASVPLSDVRPDLYVWDECHGHEHLTNFAQYALRDAQGNEVVEGRKQGFYLVDYHRQCDDAAPVFFPSDRMSISPGWTDIYVADIPCQWIDITGLADGTYSLRVGVDEQDIIEEADVHPNEALLNVHIQGDSVTVLP

>Myxococcus_xanthus_499869519

MQRISAVALTSLLAVSLCACDDPVEPGDPDSGTPDAGQPDGGDAGAGDGGTFLSEVPLWQVEHDPAHATGCFGRSIALGDVNGDGQKDLVVAAPPCLSLTRDPGRVSLFAGEAPYFSTQGVTAVMDWRNANSRTNGTRMTVSTGDVDGDDSADVLVSGQYGALVFKGQTDLASVFTEPAFVVPGGGVYNNAVFADVNGDGLDDVVSVKGLVLSVFLATPGAPGGPFTLVERTEPLYTGAVHRAGDVDGDGAQDLLLLDSMGDVYSLLHGCKAGSPGPCDGGLAAQPFWTVDSRTLSLFPDVNGDGVPERFTGPGGGTVRLHLSEAGGGYASAPTWTAMGDPVFANFGATITAVGDVDGDGQRQDFVAGSEGRLYFYSPAEGVSSALSPVWSWPRADALPQGYEGFHRFTVIAPGDLNGDRFDDLVAGVAPSPGLVDGPGGRVVIFGGGRVPAEPTTAPHLPPVAGCGLTLDPVHGKPDLSVDADVLARTLYVERRTFAADSCEMLEGCVPAPGERRLLRFSTSIQNLGAAAASVPNMDERPDLYVWDECHGHEHLTNFAQYVLRDAQGNEVVEGRKQGFYLVDYQRQCDDAAPFHFPLDRMSISAGWADIYVADIPCQWIDITDLTDGTYSLRVGVDEQDIIEEADVLPNEAILNVRIQGDSVTVVP

>Myxococcus_xanthus_521966338

MEPGDPDSGTPDAGQPDGGDAGAGDGGTFLSEVPLWQVEHDPAHATGCFGRSIALGDVNGDGQKDLVVAAPPCLSLTRDPGRVSLFAGEAPYFSTQGVTAVMDWRNANSRTNGTRMTVSTGDVDGDDSADVLVSGQYGALVFKGQTDLASVFTEPAFVVPGGGVYNNAVFADVNGDGLDDVVSVKGLVLSVFLATPGAPGGPFTLVERTEPLYTGAVHRAGDVDGDGAQDLLLLDSMGDVYSLLHGCKAGSPGPCDGGLAAQPFWTVDSRTLSLFPDVNGDGVPERFTGPGGGTVRLHLSEAGGGYASAPTWTAMGDPVFANFGATITAVGDVDGDGQRQDFVAGSEGRLYFYSPAEGVSSALSPVWSWPRADALPQGYEGFHRFTVIAPGDLNGDRFDDLVAGVAPSPGLVDGPGGRVVIFGGGRVPAEPTTAPHLPPVAGCGLTLDPVHGKPDLSVDADVLARTLYVERRTFAADSCEMLEGCVPAPGERRLLRFSTSIQNLGAAAASVPNMDERPDLYVWDECHGHEHLTNFAQYVLRDAQGNEVVEGRKQGFYLVDYQRQCDDAAPFHFPLDRMSISAGWADIYVADIPCQWIDITDLTDGTYSLRVGVDEQDIIEEADVLPNEAILNVRIQGDSVTVVP

>Myxococcus_stipitatus_505158605

MRSWWRWLAVVGAVFGGSGCEDNEPSERLLSETPLWRVTAAPNNSGECFGGSLALADFNGDGRKDLVVGTELCQEPWFASKFPGRVSIFPGQESFFSTQHVSALMTWRSTSPLASGVNLGVSAGDVNGDGFADLLVHSRFGVNVFLGQADLETMLREPAFRVAGGNQLSASSFLDLNGDGLGDFIVNHLGQEEFYLATPGAPSGLFTRALVREGFHFSLPMGDLNGDGAEDVLLSMGPGQPRGYFLGCKPGSAFSCNGPISSEPWHTESEEVVSRLIPDMNGDGYPEAFLGGQGGVSELRLSEPDGRLASSAVWSMLGDPVYPLFGDVFRMVGDLDGDGHRQDFVVGSIGRLYVFSPQGDVSEALQSVWAWPRADTLPNGYDAYRRYVVEAPGDLDGDGLEDLIVASRTYGEDMSQSRGDVSVYGGGKRPARPVDPPRLLAFEACGLGRGAETGKPDLTVDKDALQRSVHVTWRTFAAEGCEVKEQCVNAPGRRKLLRFSTVIQNLGNRSAILPSIAENPDLYVYDECHRHDHLINFAAYELRDTSGQSVLTGRKQGFRLLDLFSYCADAAPQGVYGSMGISPGWADIYTVDTPCQWVDITDLPDGTYDFQVSVDTRDIVDEGTVHPNTVSFPVRLEGNAVTVLP

>Myxococcus_stipitatus_505158606

MRSFWRTLAVASVLCVGCSDDEPGPPAKPEVLLSQTPLWQVKADPSRTNECFGGSVALADFNGDGRKDLVVGTEPCSRLMRGTPHPGRVSVFVGQESYFSTQEVSALMSWPSTHPRASGTALTVVAGDVNGDRYADLLVRSRYGASVFLGQENLEAMLAEPSFRVPGADRFQLWGGHFVDLNGDGRDDLIISRSNEQRFYLSTPGAAEGPFTLVRTRPGFLSATPVGDLNGDGADDVLLPTEDGQRGYFLGCKTGAAFACDGPISATPWRLEPLGRGGLSLADMNGDGHPEAFVSTERGPLQLHLSQSDGTLSASPIWSTMGDPTFPLLGTGAWSVGDLDGDGQRQDFVMGALGRLYFFSPDAGVSQDLEPVWSWPEENTIPNGYDVYRRYAVAVPGDLNGDGIDDLIVANTASGDSLEQPVGDVAIYSGGKVPPTRREPPYMPAPRACGLALDPVNGKPDLTVDADVLKRTVHVMWRTFGADTCEVQEQCVGAAGRRKLLRFSTSILNLGTKAAALPPIGENPDMYVMDECHGHYHLNNFAAYELRDASGNTVLSGRKQGFYLVDFQSYCTDASPADYTFDPMGISPGWADIYTLDTPCQWVDVTDLPDGDYTFQVSVDTRDIVDEGTVHPNTVGFPVRLEGDTVTVLP

>marine_metagenome_134922779

NPNFYWDDCHGHAHYEGYANYRLYNYPSLEPSETIGHKNGWCVMDLGAAVSSETPEYITNAPSCSFTYGCSIMGISKGCSYTYGSGISCQWVDVTDLADGEYVLAVSTNMETDNYVPQYEINFENNIVYVLFELETNDGLTTVVSASEFDGSAISDICAPNADATDLGFNFS

>Sorangium_cellulosum_501190949

MLRGRALELPSTARAPTMRALLRCAQRRRDTARFTPLLDRPHPVTMKKLVRTLLIGTLLAAAACGDGEPPSGSAGDGGSGNGGVSGSVRCPGSDIVCPGGARCQIDAEGKPSGCAAACDEARACGDAACCSPGAACVRGGCKAADLVLGPPERGEPVTFATVRVDEDACELLHGCFGASGRRALMRFHVSLENAGDVPLDLGVPSESDVFEPNFCEDSYVVPRFFRARLRQGGAVVAEAELDGRCVAMPGGQYGCDAQGLGPGEHVEQPGTLACNALDVTGVPAGLYEIEITVNPNRTLAEARFDNNTIVLPVDYPACDGAICGGACCPSGHACEAGVCIVPDLRPDRQRIVDTIRFSYQTFPRDACELMERCVGGPGRRHLLEFEGRVENAGPGHLDPGPEFNSPLFEYAQCHQHFHFLDFTDYRLVDAEGEVVSLGHKQAFCLVDMEPLDPSSPAPRGYPTSGGMGCNFLSAGWADTYDVGTPCQWVDVTDVPEGDYTLRVTVNPVGAIAEQAIENNVVEVPVHVPAFAPCEPEPEICGDAVDQDCDGLPDDGCEGEGCASPVPEVCGNAVDENCNGVADDGCAPIPGADSCETPGELAGSMDYAAEITAGNTSGVALPCGGGGGGEVFFRFTLPSDEVVYLGTLASDVDTALAVLEGDGCGGPPVGCAASSCGDAAPGAHLAARLRAGTYTVVVKAAHPEALGTVRLRFQRASCLGERAGVISGSGLISGDTTGAPIPPSSLCGASSPEQTFYVPVCPGMSTVVSSCGLTDFPMTIKTADDACNPINIECFGPLSACAGEREGVNSRIYGARPGGSLGAITVKGRTPVDFGPYQLFVAPFQ

>Gemmatimonadetes_bacterium_KBS708_645042500

MRRSLCSLVTLVGALYSMACAGDATPTALSPAPSRPALSSGVPDLSAPPDLIVDSKATQNNWVTRVEDFPADFCSVIEGGVTPGTHTVIRFTVTTPNIGKGDVFVGSPLAHMDPNGDGDFSDQDGLFEFASCHQHFHFQHYATYKLIGADGQVWKAAKRGFCMLDTDPYNVNNGDGTWTYRSCGTLTRDGFQGVSSGWADTYVFKLGGQYFVLDGGDGQPVVPPGVYTIQVEVNPAFAPDRRGNCPRVKDPATGLCHNFAEARYDNNVGSATVIITDHPGRSGYGPLKNDNSKITKEDEIEK

>Haloterrigena_limicola_495290131

MKIKRIKGRKRAAALIGVFLIVVAGIGIITLGGVAVDNPFTVSDRSTDTPTTSESEGTTDEGATPADEENAATPPPTTVESDPKPSDDHVEDRTGVNFVPGVENFNVSTEVFDESSPDVEDGFVTPGEHRLLRFDMIIYNMGDADAELGRPENRPDLFEYSESHGHAHLKGFNNYILLDESGERTGAVRKQTFCLRDLYQTRSTASSSPQFDCEYQGISAGWADEYDASLPGQYIVIDDLPDGEYTLQATTNAAGTINETCDGDNTVRVDLSINNDTVTVHTPQSHYVRPSAC

>Haloterrigena_turkmenica_502706271

MILNWIKERKRATALVGVLLIVVAGVGMITLGGVAADNPFTVSDSSTDTSTTSGSEDTANEEATPVDEENSTTPSTTESDSEPSDDQVEDDQVEDQPEVNFVPGVRNFDVSIEEFDESSADVEDGFVTPGEHRLLRFDMIIYNVGDADAELGHPENRSDLFEYSDSHNHAHLKGFNKYKILDEAGNEMNAGKKQTFCLRDNFQTRSNASSSAKFDCDYQGISAGWADVYPASLPGQYLVIDDLPDGEYTLQATTNAEGTIDEKCDDDNTVRVDLRINNDTVTVHSSQDDYVKPPSC

>Natronococcus_jeotgali_495698664

MKDRKRATVLVGIILIAVVGAGIITLGDVTVNNPFTVNDSNTDTSTTSESEGTANEEATPVDKENPATSSTPAESNSEPSDAQVEDKPEVNFVPGVRDFSISTEEFDESSTDVEDGFVTPGEHRLLRFDTIIYNLGDADAELGHPENRSDQFEYSDSHNHAHLKGFNKYALFDESGNEMDMGKKQTFCLRDDFQTRSNASSSAKFNCDYQGISAGWADVYPASLPGQYLVIDGLPDGEYTLHATTNAEGTIDEKCDDDNTVRVDLRINNDTVTVLSSQEDHVKPSAC

>marine_metagenome_141060514

LSVLADGAVNSSNPYPGWSFHTCHNHWHFDNYAHYALHPLHEAGAEAAANDRPPVVGHKNGWCVMDSGTWDVWAGDADYVPPSPPPPITPPVSPLAGRLLNEVHEGASPLREGASPLDASQRARRRMSHDSHLPQCARKFTCANMGISVGCYDIYDSLTDCQWIDVTDVPDGEYILSVATNWDPALRSASMEEVSYENNEARVAVQIHGATVDVLSADSACLRVNTTDPASCLSPRPPAPPSTPAPTQPPLPSAPPLPQSTSPSPRTPCPRAPHSPPPSDRLMDTSEDALLTGPALST

>Pgem_c36618_fr5

LLLLLSFIIYLLIYYLYYLLFVYFNMKSILFISSAFVFAADGYNSVPNCYHNDGGFQYAGNNNVAFSGKECLAWDDFSLHNTANRQNFLPELWPDEDLNGEVCRNPDKRPGGPWCFTKHKRKEWETCDVAPCKCDKAAIGNGVCDFVNNHKDCGYDGGDCCLSTCHCKDLGTCKGNCDCSNLHLEECGRFQEGNCRDPSVMSPESHRYIISAQTGHLRWADHKGRIKLTMFFADGSSQYTYLAGDEFKRGEVSQTELILPPVPIIKVNVAVENINDGWYLQSGINIEAEKTNRGYIFMYDGWLNEHNPSATIPRAPHNYASYTVIFHTGWAFLGESQQDLYVEVEGEGPAIGTAGIRAPKVLLGGGYEQGQEVRIHFFTVDVGDVSHLDIYGTNSEDRVYLTGGMDVIKRGVVAKMPGSGWIPKAQDEDSEACLVNNEEVPNRYITGSYSISNFEVEVTTGDHFGSGRRGGVAVTFLSHTDYPTREAYLGESFAPDQTIKTLAMVQNQGIFQAKLTLKDDFFDDVFIKQLTLKKTDKDEIKTFIFNSWLNATHPEIIVDSTNRATEAYNDELPDLEFDQYQFFINFRYKEIHVENDKLECALEEGCLYQQGEVVHGVKMDNNRRILNFETTTWNVGDVTFYPPPQQEAVYHECHKHYHGMRGYATYHLTTAGSWQNDAIILTGHKASHCVMDSTCARSGKEVRHKCVNQGIQPGCADIYNAGLDCQWIDITPLKSGIYSLNVFINRDGLVPEKSFDNNAGHILFKYDTSQTEVHKKIVWACINNDEHATECPPNSPKFDQLRTGIDRYGKVDLEPGSRRWPVRENYNDGDRWDREYEIVTINLDYNKYIYLCTFFAFKDYIYIWVFIVFLFYVNKCDYPYIYVHHFRNIK

>Awhi_c74974_fr6

RLPPDTAMYTVRVRPGFAYLGASDGDLYIEITGEGPQLGIAGETTERLLLSGGFETNVEATTKVFAKNIGDISHADLYGSNSEDRVFLNGGIEVSKTSQVVFMPGSKWIPELLADEGATCLINNEADPTRYIAGSYNVEPFHVILTIGDDVGAGRKGQINVVFKSHSDYPTRVASLGQDFERGATMTFPIFVQNQGTYQCEISLEGNTSDDVFIKTLVLHRPTSNFRKEFYFEQWLNTANPIAVTDSHDRDEASDDGLADLEFDTYQFFQNFRFKEVDIENDKLECALEEGCLYQMNEIVKGEKMGNIRALLNFETTTWNVGDVTFFAPPTQTAVYHECHKHYHGMQGYASYHITNVGSWQKEAIVLTGHKASHCVMDSTCARSGRERLHKCNNQGIQPGCADIYNAKLDCQWIDITPLKEGVYNLNVYINVEASVPEKSYDNNEGHILFKYEPHHHQAHHKIAWACLSTDESATECPPDAPKFNQLRLGITKEGEVDPEPGSRRWPLRENYNDGDRWNKEKGSKRIRRKTKNQKENFSLSLYFSIVALCLHLLGSKDSFLGAKKKKKK

>Sarc_SARC_04703

MRTLILALAVAASISNVLGQIFEEERFTCRTELMHNGACDHHNNVLECGFDGGDCCLATCDCTSNEEYVCLPGCHCNNLDSAACGSPVWTEADCKDPNIGKPNAEHLVAIWTGNGYNHQRETQIRITFNFLDGKKIQVTGGWDTSAPGRQSERSLNLEHKAIESVTVTLLDDYTDGWFMHSGVRITLPESGRVMYFPYQGWLNSDLPEVEIGVGPSDMVDYQITLWTGNNYLGDRREPLYLILYGEGPEIGILGENTTRIELNGYFSLSSKLSFTVFANEVGDVSHLDIIAPGEDAWFMQAGILITHHSVEFGVRSKYMPANGWVSSSTNSADDCTVSADGEGVKRVVSGSGNIVEYLVDIALSDAADAGREGPVSIQFYDKTDYYSRAAELGSRFPKDGHVKVTVFAQKIQVAKIRLQFGEESDDPMLVDSITVTRPDLPLQDPIQFTIGEWMTGGESDQTFTRVDFEQIPSDIEMADLDFDTIQFLNNFRAYEVDIQGDDLECALEESCLLPIGERVMGETMGDERIIIRFETTTWNVGNDVFYPPPEGEFVWHACHNHYHGMTGYATYYITTAGSFSNIILRGHKASHCVMDSTCERSGNDMQHRCTNQGIAVGCADTYSRGLDCQWVDVTPLPAGWYVMNVNINLDKRVREYSYFNNDGHILFQWDPSQPKNDMIVQACLIIDESLTECPPDTPQFDQMRPGTDGGNGGTIEPEKRFYADVDNGRW

>Cfra_g5071

VIPPLISNMSDISCEPTVTNTVHEVAVDTVPSVDTHFTEPAVSASKPSIELDALNHVAHADATDSQEPVISAPQTENTDEAEDIHVSATSTENVTNVENQSNECLGTESSSSEDGDWHPGMGSDIEDIEPSSESGESDNEEEEAAMDIDALEARYMNMCRSSKIPSAEEVMTPEQYREQLGERGNVDAHRVWRANNKLKDTDLTLLHEKVVAQLCGGEYGFEMRTPKDNEFTTALKLFTQCNCDLEQLSERLMESTVKSPTWLEVDILAFERELQLYGKDFERFKVEAKSYDDVINFYFQWMQSPRSADFVALHPESWKRFDVGPIHNCNLCVRPWKGDPTRVVEAILNRRKNGTRWEYEIFEEEKFKCIDALLHNGACDLHNNVKECGYDAGDCCLSTCDCVSHSSYTCLPGCDCNNLSSEMCSEPVWTEAMCKDPNVGKHDAEHVVSIWTGNGRNHERNGPIQLTFHYFDGSSGTETGFDTSSPGKQNDRRLYLAHKAIVAVTVSLVGDLNDGWFMHSGVRISIPDSGRTEYFPFQGWINKDNPKQQISVGDKVLSDYTVTLWTGNNYLGDREDPMYLIFYGEGPEIGILGLESEKVEINGYFALNSKLTFTVFATEVGDVSHLDIIAPGEDAWFMQAGVHISHQSVEFGLREKYMPENGWVSSSANNEAECEISADGEGIKRVVSGSGNIVEYIVDVEVDDAPDAGREGDVTIQFLDKTDYYSRTATLGSHFPKRGKIRITVFAQKIQVAKIKLKLDESFLDDPMLLRRVTVIRPDLPLQDPIVFDLNDEWMTSSRNTRTFTRVDFDTVPSDIDMADLDFDTVQFLNNFRAYDVEIDDDDLECALEENCLLPMNERVMGMTMGNRRSIIRFETTTWNVGNEVFYPPPESQFVWHDCHNHYHGMTGYATYYITTAGSFSNIILRGHKASHCVMDSTCARSGNDQEHRCTNQGIAVGCADTYSRRLDCQWVDVTPLPAGWYTMNVIINLDKRVREYSYFNNGGHILFLWDPSQGTNDKIVEACLVINEDLTECPPGTKPFDQMRKGTDGGRGGEIFPEKRFYTAQDNGRW

>Sarc_SARC_00003

MRIPSLLVAVLAVASSTLVQAHQHEGEVEADAEADILVPLNLTNTLSDESDELVEYKLTFYTGNGPEDGTKGPVRVMIQGEQVTFAEHDDDTLPHQTIEGEFLPGTSKTVIFKSLPLPEHAAWIDIWNDNKDGDDWYLLAGVKLEYKGISRVFRYEGPVHAWKAMKRASAVGGNIVPHILTITTGDAIDAELEGGDLYGKLLSDGSYESSTQMISHGVPLQRNQTIKTYMMAPEDATDALNIWFNGSNTDMWLLQTVTVQQEPTTEVPEPPITTIDFKWWLQNKIPILMHRDFDDDALSLQPNEEEGVEGRLRLPDLQIESDNMLRNLVLSAGHIWNDHRCAAEEGCFLPMGSKVNGVIMGSWRRFLRFSATYWNYGDADFFPNPDDNPEWHECHNHYHALVDFAKYTITKAGSAGNQVELASAKQSHCAVDSICEDSDDYNYKCANQGITKNCADQYGEWTDCQWIDITPLKSGWYVLNAYVNMNRRVRESDYSNNGAHVLFRFNADGGIDDRGEIDRACVLEDWEWTECPGGEDASYDRSNRCDSSSSCLQTGHLGSKSRWAD

>Cfra_g3850

MLRLYKSKKDLRRRKGEKDTKSDTGEGYDSDDDGAVLASKKEKIFKDAVVGPIVYTLYYPQDHCLLISSIVNCVIYRLSEFCTREITFCKCSHRFHTTRVLDTTNYLHIHVVVVDPPAAEVVSSAESKNEGPVGQKGYVSEVESNRLIHVLVVEEMNFFSLLVITVVIISSSGVLADHEHGHIHVTGVDTQPESPADDLVPMDDSSGMNDEEQAEYKLTFYTGNGPDDGTEGPIRVMIQGEQIEFGGHDDTSVPHQTITGNFEPGEPRTVTFTSKKLPEHAAWIDIWNDNINNDDWYLFGGVKLEYRGVSRVFRFEGPVHAQKARQRASAVGGNIVPHVIAIATGGEKDAELDGGEVYATVASDARLESATQLVSHGRSLAKNSILKSYIMAPYDAHDAITIYFVGDKTDLWLLHEVTIQREPTKEEHQPPITPITFRWWLQNNRPVLMHREFDEDALSLSENEANDFAARASLPDLTIDSQNMLDNLVLSSAYIWDDFKCAAEEGCFLPMGSKINGKTMGAKRRFLRFSATYWNNGDAEFYPNPEDDPEWHECHNHYHALKDFAKYTITKVGSGGNRVEVVAAKQSHCAVDSVCYDEDDYHFKCSNQGISRGCADQYGEWLDCQWIDITPLNSGWYILNAYLNLNRRVKEMSYKNNDAHVLFRYNAGGGFNDRGEIDRACILGDWNWLECPGGTDAEYVRSNRCDSNKSCLQRGHKGWKSRWAD

>Awhi_c78382_fr2

ICDWLQNVPQCDYDGGDCCMESCKLEEHSHTHDDSRDVNELCVCGESLKLPQCGEWLPIDCQDPNFSEPREEVLEYKITLHTGNTHSMDYTGSLEISLVGEPRHFCLENKIAKSTLFDFTEPLHPSSTVVINPKSDNVERVSYVKLRNMDPEKTFVLQAGMIIDYNNEVFYYAGNGILKPNGENTVMVKPGGGDLVWYNITIQFGDEADAGFEDGTLSIQMEDLSGRKTVLRPLLYGPVKPSSTFTVPILAQAMVPAEIELILKTNDETDSYHVLDVEVKLVDTQKKLLGDFHTAQYDAYDWLEPNVLEEIDLNGYRETLGEDTTEYATTAPWEVTKSQEVDIDLGPDYRRAVDRNSQHIFKVHKYKTNRYPDMEPDAIQMLRNMVYTVVDIDDDDLKCATEECLLPKGSYLNGEKVPDVRKLLRFETTVWNIGKVDMYPPPNNDPEWHSCHNHWHALIGFTRYTVTPAGTYDGSLLKLVKNSHCAVDSYCNRGESDKKFKCVNQGISKGCADRYGEWLDCQWVELTTLETGWYNLNVNVNSDRTLPEMDYSNNEVHVMFYFDSEGDVDDTIRYICFSEDEDQMKCPSKTKKFKNKRGDCYTDADCIRDGHKNPRSTYYKKG

>Candidatus_Nitrosopumilus_salaria_495573207

MFAAPMIMDAAAAKGGNGNGNNNGGNDETTIPTNALLPDVSPGVPKHLNIHNQQQKEFLRFTNVWANLGPGTLEFEPLFPDPDADEGTTQDAFQNLYDDEGNFGLTDQNVWHENVSQFIFHEAHNHWHIDNVGEFAVRAYDPNNPDVPGDIVDDAASIKVGFCITNVFKYNGEESPTSQRIYWDCEVGLQGIQPGWVDQYHQSVEGNEINITKVPNGTYFLTHTWNPANAFVDADNSNNVSWMKFELTDDGNGNRKINEIEGFAPECQDDDSTPGICGDINKNS

>Nitrosopumilus_504775377

MTYTKKIFRKTTLIPVLLAIGFMFTTPMLLDVAAAPGGNGNGNGGSTSIPSDALLPDISPGVPKHLNIHNQQQNEFLRFTNTWNNVGVGALEFEPVFPDSDAVEGTTQDAFQNLYDDAGNFAIPSQKIWSTTVSEFIFHETHNHWHISDIGEFSIRSDDNGVPGEIAKNVNGDDVAAVKVGFCIADVYKYNGDNSPTSQRVYWDCEVGLQGIAPGWADQYHQSVEGNEINITDLPNGTYFLVHKWNPANAFVDADVSNDESWMKFDLTDDGNGNRKIVEIEGFAPECQGDGSTPGICGEINKNN

>Marine_Group_I_thaumarchaeote_SCGC_AAA799E16_662556819

MTYTKKIFRKATILPVLLAIGFMFTTPMLLDVAAAPGGNGNNNGVPDSALLPDIIPSIPKHLQVQNAQQTETLRFTNAWGNTGVGNLEFFPIIPGVDVVETETQDSLQRLYDENGNLVWAEVVSEFEFHAEHNHWHIADIGEFAVRESTPNGPGDIITLPDGETASSIKVGFCIIDVYKIDGENSPTRDRVYWDCEVTEQGIQAGWMDQYHQSTEGNEVPITDLAPGEYYLTNEWNPANNFIDEDETNDLSWMKFELTRDSNGNAKITEIEGYSPGCNPESPGLCGEITRNN

>Actinokineospora_sp_EG49_583002477

MCLVSEVENPNAACMSRGRARAVRLRFTTAEDNVGAGPLLLYGWRPGVDVPTMRVRQAFQSGVDGRIPDSFAAAQQATATSAYYEPAQAHQHWHLMGFEHFELRTPGGGVLVADRKNGFCLGDRYATADAGVLPAVVRDDDTPQGRLGRVLAANTCNMHDPAALTVTQGISVGRGDDYRYTVDFQWLDITAVPSGTYDVVNTVNGDRTLRESDYGNNSSSIAISVRWPGGARSAPEVITRAPQVRLLRSCPGRDRCAGT

>Kutzneria_albida_644673959

MRPRTRLALVMSALLVLGLAPSAQATEPFLLPDLRQAPVGCPGGYRGDPVQCKDWDVCMVADVSNPNAPCVRDGEVRAVRLRFTSAEENIGDGPLLFYGHRDSTAQPEMTVRQAFQVGYHGNIPRSYDQAQRSSRTSAYYEPAPAHVHWHLLHFERFQLRTLSGNTLVTDRKNGFCLGDRYSPADADTMPHRVHEGDTPEAQLHEFLGPNMCGHHNPQALDVTEGISVGSGDDYTYKVDFQWLDITTVPSGIYTLVNTVNPERTLIEKDYGNDSSSVALSIQWPGAAKAAPARITAPPVVQLLRSCPGEEFCAA

>Kutzneria_albida_DSM_43870_578005885

MRSYLVAMRPRTRLALVMSALLVLGLAPSAQATEPFLLPDLRQAPVGCPGGYRGDPVQCKDWDVCMVADVSNPNAPCVRDGEVRAVRLRFTSAEENIGDGPLLFYGHRDSTAQPEMTVRQAFQVGYHGNIPRSYDQAQRSSRTSAYYEPAPAHVHWHLLHFERFQLRTLSGNTLVTDRKNGFCLGDRYSPADADTMPHRVHEGDTPEAQLHEFLGPNMCGHHNPQALDVTEGISVGSGDDYTYKVDFQWLDITTVPSGIYTLVNTVNPERTLIEKDYGNDSSSVALSIQWPGAAKAAPARITAPPVVQLLRSCPGEEFCAA

>Kutzneria_sp_744_585092190

MAAVLTLLATTPPAEAAAPMLVPDLRQAPVGCAGNHRGDPQDCKDWDVCMVTDVNAPNAPCVTSGDIRAVRLRFTSSEENIGDGPLLFYAHRDSTLFSTMSVRQAFQNGVHGPIPDTFRNAQKPSGTSAYYEPAPAHMHWHLLNFERFQLRTLSGSAVVSDRKNGFCLGDRYDVPDKASLQHRVHEDEEPEAKLHQVLIYNMCGHEQPGATDVTEGISVGSGDDYRYTVDFQWLDITGVASGVYDVVNTVNPDRTLTEKNYDNNSSSIAVSVQWPDGAKGAPSRITAPPVVRLLRSCPGSETCSG

>Amycolatopsis_balhimycina_651356086

MLKKHAILAAVVLLVTALVTASAPRADAASEVLLPDLRQAIPGCDGGSSGDLAQCDAWDVCPVIDPAVPSGRCVPVNVAKAVRLRFTSAEENIGDGPLLLYGRRDTTNQDTMSVRQALRNGPNGSIPADYVSAQRVTRAFTYYEPAVAHQHWHLMNFEHFALVSPQGKTIVTDRKNGFCLGDRFTVADAGRLSHVPGDTGPDADLAETLRANQCRHHEPTALDVLEGISVGAGDDYKYTVDFQWLDITHVPAGTYDLVNTVNADRTLLESNYRNNSTAVALTIAWPQGMPGPGAIPAAPVVKFLRACPGQPRCA

>Amycolatopsis_mediterranei_502994187

MLKKFAVLALVALSATVSASAPRADAAPDLLLPDLRQAVPGCAGGSSGELPLCTAWDVCPVVDPAAPNGRCVAPSVAKAVRLRFTSAEENVGDGPLLLYGRRDSTNQQTMTVRQALRNGADGSIPDSYAAAQRATGAFTYYEPAPAHQHWHLMNFERFALVSPQGETVVTDRKNGFCLGDRFPVHDVGRLKNVPGGSGADASLADTLRGNMCRHHEPTALEVVEGISVGAGDDYKYPVDFQWLDITHVPTGTYDLVNTVNADRTLLETNYGNNSSAVALAIAWPLGMPGADGTIPAAPVVKLLRSCPGQPRCA

>Amycolatopsis_rifamycinica_637496108

MLRKFAVLALVALAASVSASAPRADAAPDLLLPDLRQAPPGCPGGSTGELPQCTAWDVCPVIDAAAPNGRCVTPSVAKAVRLRFTSAEENVGDGPLLLYGRRESTKQDTMSVRQALRDGTDGSIPAGYAAAQRATGAFTYYEPALAHQHWHLMNFEHFALVSRQGKTVVTDRKNGFCLGDRFRVHDAARLTHVPGATGADAELAANLRDNMCRHHEPTALEVLEGISVGAGDDYKYTVDFQWLDITHVPAGTYDLVNTVNADRTLLERNYGNNASAVALSIAWPQGMPQPDGTIPAAPVVVLLRSCPGQPRCA

>Amycolatopsis_vancoresmycina_490697402

MLKKLVVLAVVVLSAVVSASAPQAGEGPDFLLPDLRQAVPGCPGGSAGPLAACTAWDVCPVIDPATPNGPCVDASVAKAVRLRFTSAEENVGAGPLLLFGRRDSTKQDTMTVRQALRTGTDGSIPDGYAAAQRATHAFTYYEPAPAHQHWHLMNFEHFALVSPQGKTVVTDRKNGFCLGDRFTVADAGRLTHVPGDTGPDADLAATLRDNMCRHHEPGALDVLEGISVGSGDDYKYTVDFQWLDITRVPAGTYDLVNTVNADRTLLETDYRNNSSAVALSISWPRGPVKPGQAIAAAPVVRFLRSCPGQPRCA

>Saccharothrix_espanaensis_504916222

MIRKFKAAAAAALTAVVLGAPASEGPLLPDLRQAPVGCPGGFSGNPDKCEAWDVCAVAERYAANGTCLGSGRAAGVRLRFTTSVDNVGDGPLLIYGKRDSTATQTMSARQAFQSAGDRSIPGSYDEARNPIPATMYYEPAPAHTHWHLMDFERFQLRAPDGTTLVLDRKNGFCLGDRYTVRDAADLPSRPRDPDSPEGLLAEFLRENRCRHHEPRALDVVEGISVGAGDDYKYDVDYQWLDLTDVPSGVYDVVNLVNVDRSFLEKDYGNNASSMAISIQWPGGGARPSVITKPPKVQLLRNCPGQERCAQP

>Pseudonocardia_dioxanivorans_503439474

MRTRAIGLMLSIGIAAVLLGACQDLDPPLLPDLIQAPVGCAPATSVAPGSCTAWDVCPVADAQNPHAACVRSGPIRQVRLRFTSSEENVGDGPLLLYGHRDSTATPTMAVRQAVQPGYDATLPASFAAAQQALPGASMYYEPAVMHQHWHLLDFDRFRLVGPDGTTLVTDRKTGFCLGDRYTSNDAYQLGYAPTDDGSPQDVLGNALAHNDCKMHDPNALDVAEGISVGSGDNYDYTVDFQWLDVTTVPSGTYTLVNTVNPDKQIQEKDYDNDSSSIAISLTWPGGGPAPRTITAPPVVHLLRSCPGHATCTATDAS

>Actinoplanes_friuliensis_556602568

MAGAVATAVGLTAAGMGVANAATDPPPLAFTLATPNVVAERYIWDDDGQAYLYFDLGVNVIAGKDPFEIRATRKSYADPIVANQVVVKNGKKKNVQLPAGMVTGWGGLKDFTTVTIKDAAGAQVANYTTDFCPNSYNSARTRRDAPAENPYPQGCSDYNPFNLGAVWGIQAGWNAATDSMPKGEDFDLADGSYTATVSLNKQYQDYFKIAADSGTLKINVKVETIDERETADGEAVVAKSKAARVGQDTSPEAAVAEHGEHAGQGDPSLQISAYKPNFRPPAKKPKAVKASSIAKGPRPDLKSLPAWGISLNQEGDKSYVAFGATVWNAGTSPLLVDGFRQTGTDLMDAYQYFFDANGKETGSVAAGTMEWDAREGHRHWHFTDFAQYNLLAADQKLAVRSGKEAFCLANTDAVDYTIPNAKWRPENTDLSSSCGQNTAVAVREVLDIGNGDTYTQDRPGQSFEVTDLPNGTYYIETKANPSNKLTELSTTNNTSLRKIILGGTPTARTLEVPPVNGIEG

>Actinoplanes_sp_N902109_505432047

MANAATDPAPLAFTLATPNVTAERYTWDGQAYLYLDLGVHVIAGKDPFEIRATRKSYADPIVAKQIIGKKSVTLPAGMVTGWGGLKDFTTVSLKDGSGAEVKTYTTDFCPNSYDSVRTRRDAPAETPYPQGCSGENPFNLGAVWGIQAGWNATTDSMPRSDDFDLPAGKYTATVTLNQKYRDYFKISAADATQTINLTVVDVAEDEKGATVAAAQVKAAKLGADPDPQVAVAEHGEHGGQGIAYQPSLRPPAKAPKAVKATSLAKGPRPDLRSLPAWGISLSQDGDKSYVNFGATVWNAGTSPLLVDGFRRTGTELMDAYQYFFDAKGNQVGSSEAGTMEWDPREGHRHWHFTDFAQYNLLAADKKLAVRSGKEAFCLANTDAVDYTIADAKWRPDNTDLSTSCGQNTAVAVREVLDIGNGDTYSQARPGQSFEVTDLPNGTYYIEVKANPANKLTELSTTNNTALREIHLGGTPTERTLEVPSVNGLD

>Actinoplanes_globisporus_522003226

MSDPKNHRHRRSRLLARGAAAGVLVVTAAGVGVAQAAANPPPLSFVAASGAVTAERYVFDGEVWLDFDLGLHVIAGKDPFEIWAHRTGYDQPVTAEQTVVQNGKKKKVALPAGTVTDFSGLKDFVSITIADRSGAVVKQYTTSFCGNSWGSARTRPDAPATTPYPTQCGNYNPFTLGAVWGVQAGWDSAVPAYDGDSFDLAPGSYAATATVNPRYQQLFGIPASQSTVHVAVTVEDADLGKSSASASLKDHEGQQVSEYVPSLRAPAKRPATTLKAAPKTGPRPDLRSLPAWGISLSQEADGKWYVNFGATVWNAGTSPLLVDGFRRSGTDLMDAYQYFFDAKGNQVGSQQAGTMEWDDRVGHKHWHFTDFAQYNLLDSTQKLAVRSGKEAFCLANTDAVDYTIPNAKWRPENTDLSTSCGANTVVAVREVLDIGNGDTYTQDRPGQSFEITDLPNGTYYIQVMANPAKKLAETNTANNTALRQIILGGTPAERTLTVPPVSGIAG

>Actinoplanes_missouriensis_504257435

MIEFRWRAVTAAAGAIALTVAGVGVAEAAAAPPAFTFLAATPNVTAERYSDSEGVYIALDLGLHVIAGKDAFEIRAKRAGYDKPVTAHRMVVKGGKKTAVALPAGTVTDFSGLKDFTTIVFKDKAGKVVTQYKTPFCGNSYSSGRTRRDAPATNPYPTRCGGENPFTLGAVWGVQAGWDATVTDQPVNGAGLSKLKAGKYTVTATVNAKYQKMFAIPANKATAKVTVTVADVKNEEKRASAAAAAKPKTAGGHGLHHGEGDASRQVSSRLAEFRAAARRPAQLKAAPATGPKPDLKSLPAWGISLAEGVNSKTGKPDGKWYVNFGATVWNAGTSPLLVDGFRRTGTELMDAYQYFFDAKGKQVGSRPAGTMEWDAREGHLHWHFTDFAQYNLLAADKKLAVRSGKEAFCLANTDAVDYTMAGAKWRPENTDLATSCGANTVVAVREVLDIGNGDTYSQDRPGQSFDITKLKNGTYYIQVLANPSKKLAELSTSNNTALRQIIIGGTAKKRTLTVPKVYGITG

>Actinoplanes_missouriensis_504255613

MTGTARFGRRWRPFVALALGATVVATGSAVANADTTPPPPLSLVTTATDVTLTRYVYEFGSFFLDPNTGVYLMAGKDPFEIEVKRKSYADPIVAQQVTTRNGRKTKAPLPAGMVTDFSGLQDFTELTMTDANGTTVVSGTQDFCPNTYETSRARPDAPDTTPYPADCFNGNPFQIGNVWGIQSGWTAAIAPSELEDLPDGTYQARIDITQRYRDHFKIAGAGTNLTITIESIFEGALSAADRVRAEAKVKATQPAANHEHLTEGDATAQASAYSPDLRPAARRPETVRSRAALPKGPRPDLRSLPAWGISLADGADLGDVPGRKYIVFGATVWNAGTSPLVVDGFRRTGTDLMDAYQYFYDAKGKQVGSANAGTMEWDKREGHLHWHFTDFAQYNLLTADQQEAVRSGKEAFCLVNTDAVDYTIPNAKVRPSNTDLSSSCGANTAVAVREVLDIGNGDTYTQFLPGQSFDVTDLPNGTYYIEVKANPSDRLAELSTTNNTSLRKVILSGSGDDRKLEVPAVHGLDGPTG

>Actinopolymorpha_alba_522062919

MASRFRYGGRLLAAATVTALLGSGTAFALPAKSEPQFRLVRASTSVILDRWEEEGVYLDLGTHVVAGKNPFEIWAKRATYKDPVVATQVIRTGGSKRRVPLPAGVVTDFTGFRDFTHVTIKNAAGKKVFERDDDFCPNSYSSARTRPDAPDKSPYPMGCPSNPFTLGSVWGIQAGWSAATSEAWEGGPVDLPVGNYTATVTLNKTYRELFKIPTTQSSATVKVTVRTPPDDCGMPQGAAKVGPTGCLRAGTAQSHRAKPTSPVPQPAAIRPTGKPRIPKGPKPDLRSLPAWGIQLTDDWDREGVRAKTPAQYLAFNATVWTAGTSPLVVDGFRRSGEDVMDAYQYFFDSKGKQVGYAPVGEMEWDARDGHEHWHFRDFAQYRLLDASKKVAVRSGKEAFCLANTDAVDYTIPNANWRPEGTDLHTACGDKSSLAVREVLDIGSGDTYAQYLPGQSFDITTLPNGAYYIEVAANPDHRLYESDTTNNVSYRKVILGGTPEARTVTVPPYDGIDA

>Kitasatospora_papulosa_662751212

MSTRTPRNRLWRPAVAAAAAIAVTAGVAAATPDTRKAAVPPRLGLVAASDSVTLDSWKEEPGVYLDLGTYLTASGAPLELRVTRKSYKDPVVATQVIRQGTRTRTRTLPEGLVKDFSGLPGFIEVTVRDTAGRTVAVRTESFCPNNASGRVDPGAPATSKYPESCPVNPFTLGSVWGVEKGWASNTYAGYYSAPVALPAGKYTAEVSVSKRYRDIFGISDAPKTVKVTVRERSWEEESPAPSPAARSASGHAGHGAGHSAHAGHPATRPPAPVSATTGAGTSYNVGHGPYAPAPAALPWAAARQGLRAAEGDGRGRTDGSRQAPAVRPNAQRPTGRAAVPDVPKPDLRSLPAYGITIGAGDGGAPGRDYLAFSANVWNAGPAELVVDGFRKPGNALMDAYQYFYDASGKQVGYTPTGTMEWDPRPGHEHWHFTDFASYRLLKADKKETVRSGKEAFCLANTDAVDYTVKNANWHPDNTDLSTACGQENSISVREVLDVGSGDTYTQDLPGQSFDITGLPNGTYFIQVLANPENRLKETDLGNNSALRKVVLGGKPGARTVTVPRHDLVDAP

>Streptomyces_sp_PAMC26508_505388869

MSTRTPRNRLWRPAVAAAAAIAVTAGVAAATPDTRKAAVPPRLGLVAASDSVTLDSWKEEPGVYLDLGTYLTASGAPLELRVTRKSYKDPVVATQVIRQGTRTRTRTLPEGLVKDFSGLPGFIEVTVRDTAGRTVAVRTESFCPNNASGRVDPGAPATSKYPESCPVNPFTLGSVWGVEKGWASNTYAGYYSAPVALPAGKYTAEVSVSKRYRDIFGISDAPKTVKVTVRERSWEEESPAPSPAARSASGHAGHGAGHSAHAGHPATRPPAPVSATTGAGTSYNVGHGPYAPAPAALPWAAARQGLRAAEGDGRGRTDGSRQAPAVRPNAQRPTGRAAVPDVPKPDLRSLPAYGITIGAGDGGAPGRDYLAFSANVWNAGPAQLVVDGFRKPGNALMDAYQYFYDASGKQVGYTPTGTMEWDPRPGHEHWHFTDFASYRLLKADKKETVRSGKEAFCLANTDAVDYTVKNANWHPDNTDLSTACGQENSISVREVLDVGSGDTYTQDLPGQSFDITGLPNGTYFIQVLANPENRLKETDLGNNSALRKVVLGGKPGARTVTVPRHDLVDAP

>Streptomyces_pratensis_503923868

MSTRTPRNRLWRPAVAAAAAIAVTAGVAAATPDTKKAAVPPRLGLVAASDSVTLDSWKEEPGVYLDLGTYLTASGAPLELRVTRRSYKDPVVATQVIRQGTRTRTRTLPEGLVKDFSGLPGFIEVTVRDTAGRTVAVRTESFCPNNASGRVDPGAPATSKYPESCPVNPFTLGSVWGVEKGWASNTYAGYYSAPVALPAGKYTAEVSVSKRYRDIFGISDAPKTVKVTVRERSWEEESPAPSPAARSASGHAGHGAGHSAHAGHPAARPPAPVSATTGAGTSYNVGHGPYAPAPAALPWAAARQGLRAAEGDGRGRTDGSRQAPAVRPNAQRPTGRAAVPDVPKPDLRSLPAYGITIGAGDGGAPGRDYLAFSANVWNAGPAQLVVDGFRKPGNALMDAYQYFYDASGKQVGYTPTGTMEWDPRPGHEHWHFTDFASYRLLKADKKETVRSGKEAFCLANTDAVDYTVKNANWHPDNTDLSTACGQENSISVREVLDVGSGDTYTQDLPGQSFDITGLPNGTYFIQVLANPENRLKETDLGNNSALRKVVLGGKPGARTVTVPRHDLVDAP

>Streptomyces_baarnensis_662125240

MMTRSQQGRTHRLTRPSIAIAAAVAVTAGVVAAAPDAKTAEPTPKLGLVAASTSVTLDSWKEDPGVYLDLGTYLTSENGAFELKVTRKSYKDPVVAQQILRNGKKTTTKALPAGLVKDFSGLPDFAKIAITDAAGKTVLSKTEAFCPNNASGRIRPDAPANSKYPQSCPVNPFTLGSVWGVENGWASNTYAGYYSDPVQLAAGTYTAKISVTKKYRDLFGIANKPQTVKVVVRERSWENEGEGGGEGPAAEPRSAAPKSGASAASASGHEGHAGHGGHAGHGQVADAPAAPSAHASHGAPARPEAPAARTTGAAPTFNVGHGPYPPAPPALPWALKKESLQRQAFSAAKVGDRAGQTDGSRQAPGATPNAKRPTGKATVPDVPKPDLRSLPAYGITVSDGYEDVPGKDYLAFSANVWNAGPAKLVVDGFRSPGKELMDAYQYFYDADGKQVGYTPTGTMEWDPRPGHEHWHFTDFASYRLLKADKKETVRSGKEAFCLANTDAVDYTVKNANWHPDNTDLSTACGQENSISVREVLDVGSGDTYTQDLPGQSFDITDLPNGTYYIQVLANPENRLKETNHNNNSALRKVVLGGKKGARTVKVPAHHLVDAN

>Streptomyces_filamentosus_493081154

MTRSPQGRTSRLTRPSIAVAAAVAVTAGVVAAAPDAKTAEPSPKLSLIAATTSVTLDSWKEEPGVYLDLGTYLTSENGAFELKVTRKSYKDPVVATQVLRNGKKTTTKALPAGLVKDFSGLPDFAQISVTDAAGKTVLKQTEAFCPNNASGRIRPDAPANSKYPQSCPVNPFTLGSVWGVENGWASNTYAGYYAQPVKLAAGTYTAKISVTKKYRDLFGIANKPQTVKVTVRERSWEEEEQGGDAAARSAAPQSSKSAKSAAGHQRHQGHGGHEGHQQAADAPAAASAHAAHGAPARPEAPATRTTGATPTFNVGHGPYPPAPPALPWALKKESLQRQAFSAAKVGDRPGQTDGSRQAPAAEPNAKRPTGKATVPDVPKPDLRSLPAYGITVNDGYEEKKSGKDYLAFSANVWNAGPAKLVVDGFRSPGKELMDAYQYFYDAKGKQVGYTPTGTMEWDPRPGHEHWHFTDFASYRLLKADKKESVRSGKEAFCLANTDAVDYTVKNANWHPDNTDLSTACGQENSISVREVLDVGSGDTYTQDLPGQSFDITGLPNGTYYIQVLANPENRLKETNYKNNSALRKVVLGGKKGARTVKVPAHHLVDAN

>Streptomyces_filamentosus_588272803

MMTRSPQGRTSRLTRPSIAVAAAVAVTAGVVAAAPDAKTAEPSPKLSLIAATTSVTLDSWKEEPGVYLDLGTYLTSENGAFELKVTRKSYKDPVVATQVLRNGKKTTTKALPAGLVKDFSGLPDFAQISVTDAAGKTVLKQTEAFCPNNASGRIRPDAPANSKYPQSCPVNPFTLGSVWGVENGWASNTYAGYYAQPVKLAAGTYTAKISVTKKYRDLFGIANKPQTVKVTVRERSWEEEEQGGDAAARSAAPQSSKSAKSAAGHQRHQGHGGHEGHQQAADAPAAASAHAAHGAPARPEAPATRTTGATPTFNVGHGPYPPAPPALPWALKKESLQRQAFSAAKVGDRPGQTDGSRQAPAAEPNAKRPTGKATVPDVPKPDLRSLPAYGITVNDGYEEKKSGKDYLAFSANVWNAGPAKLVVDGFRSPGKELMDAYQYFYDAKGKQVGYTPTGTMEWDPRPGHEHWHFTDFASYRLLKADKKESVRSGKEAFCLANTDAVDYTVKNANWHPDNTDLSTACGQENSISVREVLDVGSGDTYTQDLPGQSFDITGLPNGTYYIQVLANPENRLKETNYKNNSALRKVVLGGKKGARTVKVPAHHLVDAN

>Streptomyces_sp_HCCB10043_560232026

MPDVPKPDLRSLPAYGITVNDGYEEKKSGKDYLAFSANVWNAGPAKLVVDGFRSPGKELMDAYQYFYDAKGKQVGYTPTGTMEWDPRPGHEHWHFTDFASYRLLKADKKESVRSGKEAFCLANTDAVDYTVKNANWHPDNTDLSTACGQENSISVREVLDVGSGDTYTQDLPGQSFDITGLPNGTYYIQVLANPENRLKETNYKNNSALRKVVLGGKKGARTVKVPAHHLVDAN

>Streptomyces_griseus_490065820

MMTRSPQGRTNRLTRPSIAVAAAVAVTAGVVAAAPDAKTAAAATPKLSLIAASTSVTLDSWKEDPGVYLDLGTYLTSENGAFELKVTRKSYKDPVVASQVFRNGKKTTTKALPAGLVKDFSGLPGFAQIKLTDAAGKTVLSQTEAFCPNNASGRVRPDAPANSKYPQSCPVNPFTLGSVWGVENGWASNTYAGYYSKPVQLAAGTYTAKINVTKKYRDLFGIANQTRTVKVTVRERSWEEPTPVPAGSRSAASAHQGHGGSGGHEGHEGHGKAAAPAAPSAHAHGAPARPEAPAARTTGAAPTFNVGHGPYPPAPPALPWALKKESLQREAFSAAKVGDRAGQTDGSRQAPGAKPNAKRPTGKATVPDVPKPDLRSLPAYGITVSDGYEEVPGKDYLAFSANVWNAGPAKLVVDGFRSPGKELMDAYQYFYDANGKQVGYTPTGTMEWDPRPGHEHWHFTDFASYRLLKADKKESVRSGKEAFCLANTDAVDYTVKNANWHPDNTDLSTACGQENSISVREVLDVGSGDTYTQDLPGQSFDITDLPNGTYYIQVLANPENRLKETNHKNNSALRKVVLGGKKGARTVKVPAHELVNAN

>Streptomyces_griseus_501348544

MTRSPQGRTNRLTRPSIAVAAAVAVTAGVVAAAPDAKTAAAATPKLSLIAASTSVTLDSWKEDPGVYLDLGTYLTSENGAFELKVTRKSYKDPVVASQVFRNGKKTTTKALPAGLVKDFSGLPGFAQIKLTDAAGKTVLSQTEAFCPNNASGRVRPDAPANSKYPQSCPVNPFTLGSVWGVENGWASNTYAGYYSKPVQLAAGTYTAKINVTKKYRDLFGIANQTRTVKVTVRERSWEEPTPVPAGSRSAASAHQGHGGSGGHEGHEGHGKAAAPAAPSAHAHGAPARPEAPAARTTGAAPTFNVGHGPYPPAPPALPWALKKESLQREAFSAAKVGDRAGQTDGSRQAPGAKPNAKRPTGKATVPDVPKPDLRSLPAYGITVSDGYEEVPGKDYLAFSANVWNAGPAKLVVDGFRSPGKELMDAYQYFYDANGKQVGYTPTGTMEWDPRPGHEHWHFTDFASYRLLKADKKESVRSGKEAFCLANTDAVDYTVKNANWHPDNTDLSTACGQENSISVREVLDVGSGDTYTQDLPGQSFDITDLPNGTYYIQVLANPENRLKETNHKNNSALRKVVLGGKKGARTVKVPAHELVNAN

>Streptomyces_sp_W007_494721500

MMTRSPQGRTNRLTRPSIAVAAAVAVTAGVVAAAPDAKTAEPTPKLSLIAASTSVTLDSWKEDPGVYLDLGTYLTSENGAFELKVNRKSYKDPVVATQVFRNGKKTTTKALPAGLVKDFSGLPDFAQIKLTDAAGKTVLSQTEAFCPNNASGRVRPDAPANSKYPQSCPVNPFTLGSVWGVENGWASNTYAGYYSKPVQLAAGTYTAKINVTKKYRDLFGIANQTRTVKVTVRERSWEEPPPVPAGGRSAASAHEGHEGHGKAAAPSAASGHGAHGAPARAEAPAARTTGAAPTFNVGHGPYPPAPPALPWALKKESLQRQSFSAAKVGDRSGQTDGSRQAPGVKPNTKRPTGKATVPDVPKPDLRSLPAYGITVSDGYEEVPGKDYLAFSANVWNAGPAKLVVDGFRSPGKELMDAYQYFYDANGKQVGYTPTGTMEWDPRPGHEHWHFTDFASYRLLKADQKESVRSGKEAFCLANTDAVDYTVKNANWHPDNTDLSTACGQENSISVREVLDVGSGDTYTQDLPGQSFDITDLPNGTYYIQVLANPENRLKETNHKNNSALRKVVLGGKKGARTVKVPAHHLVDAN

>Streptomyces_sp_CNB091_517786958

MMNRPPQGRTNRLTRPSIAVAAAVAVTAGVVAAAPGAETAEPTPKLSLIAASTSVTLDSWKEDPGVYLDLGTYLTSENGAFELKVTRKSYKDPVVASQVFRNGKKTTTKALPAGLVKDFSGLPDFAQIKLTDAAGKTVLSQTEAFCPNNASGRVRPDAPANSKYPQSCPVNPFTLGSVWGVENGWASNTYAGYYSKPVQLAAGTYTAKINVTKKYRDLFGIANQTRTVKVTVRERSWEEPPPVPAGSRSAASAHQGHGENAGHAGHAGHEGHGKAAAPAAPSAHAHGAPARTEAPAARTTGAGSTFNVGHGPYPPAPAALPWALKKESLQREAFSAAKAGDRAGQTDGSRQAPGVKPNAKRPTGKAVVPDVPKPDLRSLPAYGITVSDGYQEVPGKDYLAFSANVWNAGPAKLVVDGFRSPGKELMDAYQYFYDAAGKQVGYTPTGTMEWDPRPGHEHWHFTDFASYRLLKADQKESVRSGKEAFCLANTDAVDYTVKNANWHPGNTDLSTACGQENSISVREVLDVGSGDTYTQDLPGQSFDITGLPNGTYYIQVLANPENRLKETNHNNNSALRKVVLGGKKGARTVKVPAHHLVNAN

>Streptomyces_globisporus_497742382

MMTRSPQGRTNRLTRPSIAVAAAVAVTAGVVAAAPDAKTAEPTPKLSLIAASTSVTLDSWKDDPGVYLDLGTYLTSENGAFELKVTRKSYKDPVVATQVLRNGKKTTTKALPAGLVKDFSGLPGFAQVSVTDAAGKTVLKQTEAFCPNNASGRIRPDAPAQSKYPQSCPVNPFTLGSVWGVENGWASNTYAGYYAKPVQLAAGTYTAKISVTKKYRDLFGIANKPQTVKVTVRERSWEEEEGSGSAARSAAPQSAKSAAGHQGHTGHSGHGGHEGHQQATDAPAASAHAAHGAPARPEAPAARTTGAAPTFNVGHGPYPPAPPALPWALKKESLQRQAFSAAKVGDRPGQTDGSRQAPGAKPNAKRPTGKATVPDVPKPDLRSLPAYGITVSDGYEDVPGKDYLAFSANVWNAGPAKLVVDGFRSPGKELMDAYQYFYDAKGKQVGYTPTGTMEWDPRPGHEHWHFTDFASYRLLKADKKESVRSGKEAFCLANTDAVDYTVKNANWHPGNTDLSTACGQENSISVREVLDIGSGDTYTQDLPGQSFDITGLPNGTYYIQVLANPENRLKETNHKNNSALRKVVLGGKKGARTVKVPAHDLVNAN

>Streptomyces_californicus_662174297

MMTRFPQGRTNRLTRPSIAVAAAVAVTAGVVAAAPDAKTTEPAPKLRLIAASTSVTLDSWKEDPGVYLDLGTYLTSENGAFELKVTRKSYKDPVTVSQVFRNGKKTTTKALPAEIVKDFSGLPDFAQIQLTDAAGKTVLKQTEAFCPNNASGRVRPDAPANSKYPQSCPINPFTLGSVWGVENGWASNTYAGYYSKPVQLAAGTYTAKIDVTKKYRDLFGIASKPQTVKVVVRERSWEEPAGAGRSAAATRQGHQGHGAHQGHEAHGQQAAPSHGAHAAHGAPARTEAPAARTTGAGATFNVGHGPYPPAPPALPWALKKDSLQRQSFAAATVGDRAGQTDGSRQAPAVQPNAKRPTGKATVPDVPKPDLRSLPAYGITVSEGYEEAPGRDYLAFSANVWNAGPAKLVVDGFRSPGKELMDAYQYFYDANGKQVGYTPTGTMEWDPRPGHEHWHFTDFASYRLLKADKKETVRSGKEAFCLANTDAVDYTVKNANWHPDNTDLSTACGQENSISVREVLDVGSGDTYTQDLPGQSFDITGLPNGTYYIQVLANPENRLKETNHKNNSALRKVVLGGKKGARTVKVPAHELVNAN

>Streptomyces_puniceus_663156839

MMTRFPQGRTNRLTRPSIAVAAAVAVTAGVVAAAPDAKTAEPAPKLRLIAASTSVTLDSWKEDPGVYLDLGTYLTSENGAFELKVTRKSYKDPVTVSQVFRNGKKTTTKALPAGIVKDFSGLPDFAQIQLTDAAGKTVLKQTEAFCPNNASGRVRPDAPANSKYPQSCPINPFTLGSVWGVENGWASNTYAGYYSKPVQLAAGTYTAKIDVTKKYRDLFGIASKPQTVKVVVRERSWEEPAGAGRSAAATRQGHQGHGAHQGHEAHGQQAAPSHGAHAAHGAPARTEAPAARTTGAGATFNVGHGPYPPAPPALPWALKKDSLQRQSFAAATVGDRAGQTDGSRQAPAVQPNAKRPTGKATVPDVPKPDLRSLPAYGITVSEGYEEAPGRDYLAFSANVWNAGPAKLVVDGFRSPGKELMDAYQYFYDANGKQVGYTPTGTMEWDPRPGHEHWHFTDFASYRLLKADKKETVRSGKEAFCLANTDAVDYTVKNANWHPDNTDLSTACGQENSISVREVLDVGSGDTYTQDLPGQSFDITGLPNGTYYIQVLANPENRLKETNHKNNSALRKVVLGGKKGARTVKVPAHELVNAN

>Streptomyces_sp_NRRL_B1381_663337072

MMTRFPQGRTNRLTRPSIAVAAAVAVTAGVVAAAPDAKTAEPAPKLRLIAASTSVTLDSWKEDPGVYLDLGTYLTSENGAFELKVTRKSYKDPVTVSQVFRNGKKTTTKALPAGIVKDFSGLPDFAQIQLTDVAGKTVLKQTEAFCPNNASGRVRPDAPANSKYPQSCPINPFTLGSVWGVENGWASNTYAGYYSKPVQLAAGTYTAKIDVTKKYRDLFGIASKPQTVKVVVRERSWEEPAGAGRSAAATRQGHQGHGAHQGHEAHGQQAAPSHGAHAAHGAPARTEAPAARTTGAGATFNVGHGPYPPAPPALPWALKKDSLQQQSFAAATVGDRAGQTDGSRQAPAVQPNAKRPTGKATVPDVPKPDLRSLPAYGITVSEGYEEAPGRDYLAFSANVWNAGPAKLVVDGFRSPGKELMDAYQYFYDANGKQVGYTPTGTMEWDPRPGHEHWHFTDFASYRLLKADKKETVRSGKEAFCLANTDAVDYTVKNANWHPDNTDLSTACGQENSISVREVLDVGSGDTYTQDLPGQSFDITGLPNGTYYIQVLANPENRLKETNHKNNSALRKVVLGGKKGARTVKVPAHELVNAN

>Streptomyces_fulvissimus_505422988

MLFVRVPSRTRKPEVRGMMTRSQQGRTNRLTRPSIAIAAAVAVTAGVVAAAPDAKTAQPGPKLSLIAASASVTLDSWKEDPGVYLDLGTYLTSENGAFELKVTRKSYKDPVVAAQIIRDGKKTQTKALPAGLVKDFSGLPDFAQISVIDAAGKSVLEKTEAFCPNNASGRVRPDAPANSKYPQSCPVNPFTLGSVWGVENGWAANTYAGYYSDAVQLPAGTYTAKISVTKKYRDLFGIEDKPQSVKVTVRERSWEGEGGGEGVSASSESSEYSAGASSKTDAHAGHGSAASEHAGHAAAAPAAPSGHAGHGAPARTDAPAAATTGAGSTFNVGHGPYPPAPPALPWALKKQATEKGALGRQGFSAAAVGDRTGQTDGSRQAPGVQPNAKRPAGKATVPDVPKPDLRSLPAYGITVSDGYEDVPGKDYLAFSANVWNAGPAKLVVDGFRSPGKELMDAYQYFYDADGKQVGYTPTGTMEWDPRPGHEHWHFTDFASYRLLKADQKETVRSGKEAFCLANTDAVDYTVKNANWHPDNTDLSTACGQENSISVREVLDVGSGDTYTQDLPGQSFDITDLPNGTYYIQVLANPENRLKETNHDNNSALRKVVLGGKKGARTVKVPAHHLVDAN

>Streptomyces_sp_CcalMP8W_517299657

MMTRSQQGRTNRLTRPSIAIAAAVAMTAGVVAAAPEAKTAQPGPKLSLIAASASVTLDSWKEDPGVYLDLGTYLTSENGAFELKVTRKSYKDPVVAAQIVRDGKKTRTKALPAGLVKDFSGLPDFARISVIDAAGKSVLERTEAFCPNNASGRVRPDAPANSKYPQSCPVNPFTLGSVWGVENGWAANTYAGYYSDAVQLPAGTYTAKISVTKKYRDLFGIADQPQSVKVTVRERSWEGEGDDEGVSAPSAGAAASKTAALSGHDAHAGHGAPAPATSAHAGHGAAAPASAHAGHGAPARTDAPAAATTGAGATFNVGHGPYPPAPPALPWALKRQAAEKGALGRQGFSAAAIGDRAGQTDGSRQAPGAQPNATRPTGRATVPNVPKPDLRSLPAYGITISDGYEDVPGKDYLAFSANVWNAGPAKLVVDGFRSPGKELMDAYQYFYDAQGKQVGYTPTGTMEWDPRPGHEHWHFTDFASYRLLKADRKETVRSGKEAFCLANTDAVDYTVKNANWHPENTDLSTACGQENSISVREVLDVGSGDTYTQDLPGQSFDITDLPNGTYYIQVLANPENRLEETDLSNNSALRKVVLGGKKGARTVTVPAHHLVDAN

>Streptomyces_sp_SolWspMPsol2th_654970409

MMTRSQQGRTNRLTRPSIAVAAAVAMTAGVVAAAPDATTAQPGPKLSLIAASASVTLDSWKEDPGVYLDLGTYLTSENGAFELKVTRKSYKDPVVAAQIIRDGKKTLTKALPAGLVKDFSGLPDFARISVIDAAGKPVLERTEAFCPNNASGRVRPDAPANSKYPQSCPVNPFTLGSVWGVENGWAANTYAGYYSDAVQLPAGTYTAKISVTKKYRDLFGIADKPQSVKVTVRERSWEGEGDGEEVSAPSAGAAASKTAAPSGHDAHAGHGAAPAAASAHAGHGAPARTDAPAAATTGAGATFNVGHGPYLPAPPALPWALKRQATEKGVLGGQGFSAAAIGDRAGQTDGSRQAPGARPNAERPAGKATVPDVPKPDLRSLPAYGITISDGDEDVPGKDYLAFSANVWNAGPAKLVVDGFRSPGKELMDAYQYFYDAQGKQVGYTPTGTMEWDPRPGHEHWHFTDFASYRLLKADRKEAVRSGKEAFCLANTDAVDYTVKNANWHPENTDLSTACGQENSISVREVLDVGSGDTYTQDLPGQSFDITDLPNGTYYIQVLANPENRLKETDLSNNSALRKVVLGGKKGARTVTVPAHHLVDAN

>Streptomyces_sp_ScaeMPe10_517338091

MMTRSQQSRTSRLTRPSVAIAAAVAVTAGVVAAAPDAKTAAVAGPKLSLIAASKSVTLDSWKEEPGVYLDLGTYLTSEKGAFEFKVTRKSYKDPVVASQIIRNGKKTTTKALPAGLVKDFSGLPGFMEVNVVDAAGKSVLKKTETFCPNNASGRVRPDAPAQSKYPQSCPVNPFTLGSVWGVENGWAANTYAGYYSAPVQLAAGTYTAKISVTKKYRDLFGIANKPQSVKLTVRERSWEAPEGGSGGAAAKVASSSLHSGHAGHAGHQAAADPAPSGHAGHGAPARTDAPASATTGAGATFNVGHGPYPPAPPALPWALKKQAAEKQSGKDAFGAQGFSAARSGDRAGQTDGSRQAPGAQPNAKRPTGKAAVPNVPKPDLRSLPAYGITVSDGYEDVPGKDYLAFSANVWNAGPAKLVVDGFRSPGAELMDAYQYFYDAKGKQVGYTPTGTMEWDPRPGHEHWHFTDFASYRLLKADKKETVRSGKEAFCLANTDAVDYTVKYANWHPSNTDLSTACGQENSISVREVLDVGSGDTYTQDLPGQSFDITNLPNGTYYIQVLANPENRLKETNHKNNSALRKVVLGGKKGARTVKVPAHHLVNAN

>Streptomyces_sp_NRRL_WC3626_663187399

MTKLQKGRPSHLNRPYLAVAAAVAVVAGVVAAAPDAKSEEPTPKLSLIAASTSVTLDSWKEDPGVYLDLGTYLTSENGALELKVTRKSYKDPVVAQQILRNGKKTRTRTLPAGLVNDFAGLPDFTQITLTDEAGSTVLEKSEAFCPNNASGRVRPDAPANSKYPESCPTNPFTLGSVWGVENGWASNTYSGFYTAPVQLAAGTYTAEINVAEKYRDLFGIADKPQTVKVVVRERSWEDEESAGARGSAKTTASGHSAHAEHDAHAEHSAHARHGAGADTPAPGAVTSGAGPSYNAGHGPYPSAPPALPWAMKRKALGRQALTTAEAGDSPGQTDGSRQAPGALPNAKRPAGKASVPDVPKPDLRSLPAYGITVSDGYEEVPGKDYLAFSANVWNAGPAKLVVDGFRSPGKEVMDAYQYFYDTDGTQVGYTPTGGLEWDPRPGHEHWHFLDFASYRLLTADQKETVRSGKEAFCLANTDAVDYTVKNADWHPGNTDLSTACGQENSISVREVLDVGSGDTYTQDLPGQSFDITDLPNGTYYIQVLANPENRLKETDHSNNSALREVVLGGEKGARTVTVPPHHLVDAN

>Streptomyces_pristinaespiralis_491457536

MTRSHHARLWRPAIAAGAAIAVTAGVVAAAPDARKAQTKPKFSLIAASTSVTLDSWKEEPGVYLDLGTYLTAENGPLELKVTRKSYKDPVVATQIIRQGNKAPRTKTLPAGLVKDFSGLPGFVRITVTDAAGKTVVTKDEAFCPNNASGRVRPDAPAKSKYPQSCPTNPFTLGSVWGVENGWASNTYSGFYTAPVALPAGKYTAKVSVAKRYRDLFGIADKPQTISVTVRERSWEEGARASAAKEHAGHAAGHSGHSATRAAAPAAPTSGAGPSYNAGHGPYPSAPPAVPWAIKKAALKAAQAGDGAGRTDGSRQAPALSPNANRPTGKASVPDVPKPDLRSLPAYGIQISDGGDDVPGKDYLAFSANVWNAGPAQLVVDGFRSPGKKLMDAYQYFYDAKGKQVGYAPTGTMEWDPRPGHEHWHFTDFASYRLLKADQKEAVRSGKEAFCLANTDAVDYTVKNANWHPENTDLSTACGQENSISVREVLDVGSGDTYTQDLPGQSFDITDLPNGTYYIQVLANPEKRLQETDLDNNSALRKVVLGGKPGARTVKVPAHDLVDAN

>Streptomyces_flavidovirens_655413940

MTRFQQARPWRAVIAAAAAITVTAGVVAAAPDAQTQAQPTPKLSLIAASTSVTLDSWKEEPGVYLDLGTYVTAENGPLELKVTRKSYKDPVVATQVIREGTRTRTRTLPQGLVKDFAGLPDFAKVTITDASGRKVMDKLEAFCPNNASGRVRPDAPAKSKYPESCPINPFTLGSVWGVENGWASNTYAGYYSAPVALPAGKYTAKVSVTKRYRDLFGMENKPRTIKVTVRERSYDDERPGAGSAAAMRHGSQGSQGSHGSHAAGHSGHGAMRAPAPAAATSGAGPSYNVGHGPHTPAPPALPWSLKKAALRAAPVGDAAGQTDGSRKAPAVKPAAKRPAGTPSVPDVPKPDLRSLPAYGITISKGQGDTSRKDYLAFSANVWNAGPAQLVVDGFRTPGKELMDAYQYFYDTDGKQVGYTPTGTMEWDPRPGHEHWHFTDFASYRLLKADKKESVRSGKEAFCLANTDAVDYTVKNANWHPGNTDLSTACGQENSISVREVLDVGSGDTYTQDLPGQSFDISDLPNGTYYIQVLANPEKRLKETSLNNNSALRKVVLGGRAGARTVTVPAHDLVDAN

>Streptomyces_niveus_558884699

MTRTHQARYRRSAMAATAALAVTAGVVAAAPDSASAQAAPKLSLIAATNSLTLDSWKEDPGVYLDLGTYLTAEGGPFELKVTRKSYKDPIVVKQLIQDGDKTTSKTLPAGLVKDFSGLPDFAQISITDTNRKVVVKKSEKYCPNNAAGRIDPDGPAKSKYPESCPPNAFTLGSVWGVEKGWASNTYAGFDSEPVKLAAGKYTAKVTVNKKYRDLFGISSKPQIIKLTVRERSWEDENGGGVGMRAKGADHSGHDMSAMKGMAGHGSMRPKAPAAPTSGAVPSYNVGHGPLIPAPPALPWALKKSSLRSAVAPVGDGPGRTDGSRQAPGAEPNAKRPTGKASVPDVPKPDLRSLPAYGIEISDGGQDVKGKDYLAFSANVWNAGPAQLVVDGFRKPGKELMDAYQYFYDADGKQVGYTPTGTMEWDPRPGHEHWHFTDFASYRLLKADQKETVRSGKEAFCLANTDAVDYTVKNANWHPGNTDLSTACGQENSISVREVLDVGSGDTYTQDLPGQSFDITNLANGTYYIQVLANPENRLKETNLDNNSALRKVVLGGKPGARTVKVPAHDLVNSN

>Streptomyces_bikiniensis_663181323

MTTTRTTRLRRPLLAGTTAVAAMAVTAGFMTAAPEQAKAATGPKLSLVAATGSVTLTSWKEDPGVYLDLGTYLTAEGTPLELKVTRKSYKDPVTVTQTVYEGGKAKAKALPAGTVKDFSGLPGFAEITLTDKAGKKVVSRTESFCPNNASGRIRPDAPATSKYPESCPTNPFTLGSVWGVEKGWASNTYAGSYTRPVKLAAGTYTARIGVAKKYRDLFGIADKPATVKVTVQERSWEEGEGVSAARSAGAGEHAGHGTAGGHAGHGAAHHAPAGHAGHGGPVKPVQAGAPETSGATPSYNVGHGPLTAAPPALPWALKKQQAALAGARGADAAGARAGDVPGRTDGSRKAPALAPAAERPTGKPSVPDVPKPDLRSLPAYGITISDGTENVPGKDYLAFSANVWNAGPAQLVVDGFRSPGKAKMDAYQYFYDAKGKQVGYTPTGTMEWDPRPGHVHWHFTDFASYRLLKADKKEAVRSGKEAFCLANTDAVDYTVKNANWHPNNTDLATACGQENSISVREVLDVGSGDTYTQDLPGQSFDITDVPNGTYYIQVLANPAKRLKETNLNNNSALRKVVLGGKPGKRTVTVPAHDLVNAN

>Streptomyces_exfoliatus_639898950

MTTTRNHRLRRPLLAGTTAVAAMAVTVGFMAATPEQAKAAAGPKLSLIAATGSVTLTSWKEEPGVYLDLGTYLTAEGTPLEFKVTRKSYKDPVTITQTVYEGGKAKAKTLPLGTVKDFSGLPGFAEITVTDKTGKKVVSRTESFCPNNASGRVRPDAPSTSKYPESCPTNPFTLGSVWGVEKGWAANTYGGSYTQPVKLAAGTYTAKIGVAKKYRDLLGIADRPATVKLTVQERSWEEGPGAAPARSSAAGEHAGHGTAHQAPAGHAGHGSAHAETPAQAGAPETSGAGPSYNVGHGPLKAAPPAVPWAVKRQQAARADMQAADTAGQTDGSRQAPALTPRSQRPSGTPTVPNVPKPDLRSLPAYGITISDGGQNVPGKDYLAFSANVWNAGPAQLVVDGFRTPGKAKMDAYQYFYDAAGKQVGYTPTGTMEWDPRPGHVHWHFTDFASYRLLKADKKEAVRSGKEAFCLANTDAVDYTVKNANWHPFNTDLATACGQENSISVREVLDVGSGDTYTQDLPGQSFDITDVPNGTYYIQVLANPAKRLKETNLANNSALRKVVLGGAPGRRTVTVPAHDLVNAN

>Streptomyces_venezuelae_504851207

MTTTRTTRLRRPLLAGTTAVAAMAVTAGFMAATPEQATAATGPRLSLIAATDSVTLTSWKEEPGVFLDLGTYLTAEGTPLELKVTRKSYKDPVVVTQTVYEGGKAKAKVLPQNTVKDFSGLPGFAEITVTDSTGKKVVSRTESFCPNNASGRVRPDAPATSKYPESCPTNPFTLGSVWGVEKGWAANTYSGAYTQPVKLAAGTYTAKVGVAKKYRDLFGIADKPATVRVTVQERSWEDGGGAAARQTAGEHAGHGAGQQAPAAHAGHGPGHAPSPAQAGAPETSGAGPSYNVGHGPLKAAPPALPWALKRQEAARAEARAADTTGQTDGSRKAPALTPQAKRPTGTPSVPNVPKPDLRSLPAYGITISDGGQDVPGKDYLAFSANVWNAGPAQLVVDGFRSPGKPTMDAFQYFYDANGKQVGYTPTGTMEWDPRPGHVHWHFTDFASYRLLKADQKEAVRSGKEAFCLANTDAVDYTVKNANWHPFNTDLSTACGQENSISVREVLDVGSGDTYTQDLPGQSFDITDVPNGTYYIQVLANPAKRLKETNLDNNSALRKIVLGGTPGKRTVTVPAHDLVNAN

>Streptomyces_flavochromogenes_663324668

MSTTRTNRLRRPLLAGATAVAAVAVTVGLMTAAPEQATAATGPTLSLVAATGSVTLTSWKEEPGVYLDLGTYLTAEGRPLELKVTRKSYKDPVTVTQTVFEGGGSKAKTLPLGTVKDFSGLPGFAEITVTDRDGKVVVSRAESFCPNNASGRIRPDAPATSKYPESCPTNPFTLGSVWGVEKGWAANTYGGSYTEPVKLAAGTYTAKVRVAKKYRDLFGIADKPASVKLTVEERSWENGRSAAAGEHAGHGPAHQAPSAHAGHGAGHAPTAAQAGAPQTSGAGPSYNVGHGPLKAAPPALPWALKVQQAGRAGVRAAGDTPGQTDGSRQAPALAPRPARPTGTPSVPNVPKPDLRSLPAYGITISDGGAQDIPGKDYLAFSANVWNAGPAQLVVDGFRSPGKAMMDAYQYFYDAQGKQVGYTPTGTMEWDPRPGHVHWHFTDFASYRLLKADQKEAVRSGKEAFCLANTDAVDYTVKNANWHPNNTDLSTACGQENSISVREVLDVGSGDTYTQDLPGQSFDITDLPNGTYYIQVLANPAKRLKETNLDNNSALRKVVLGGTPGRRTVTVPAHDLVNAN

>Streptomyces_coelicoflavus_ZG0656_371551880

HAPTPAQAAAPVTSGAGPSYNVGHGPLRAAPPALPWALKKKQALRSLPLGDKGGQTDGSRKALALQPLAKRPAGKPSVPDVPKPDLRSLPAYGIVVSDGEQDVPGKDYLAFSANVWNAGPAQLVVDGFRSPGKAKMDAYQYFYDSKGKQVGYTPTGTMEWDPRPGHVHWHFTDFASYRLLKADKKEAVRSGKEAFCLANTDAIDYTVKNANWHPFNTDLSTACGEENSISVREVLDVGSGDTYSQDLPGQSFDITDVPNGTYYIQVLANPEKRLKETNLGNNSALRKIVLGGKPDARTVTVPPHDLVNAN

>Streptomyces_sp_NRRL_F5555_663404841

MTRTRTTRLRRPLLVGTTAVAAMAVTGSLVAATAEPAKAATGPKLSLVAATGSLTLTSWKEDPGVYLDLGTYLTAEGRPLELKVTRKSYKDPVTVTQTVYEGGKAKARTLPKGTVKDFSGLPGFAQITITDKAGKKVLGRTEDFCPNNASGRVRPDAPATSKYPESCPTNPFTLGSVWGVEKGWAANTYGGSYSEPVALAPGTYTAKVGVAKKYRDLFGIADKPATVKVTVVERSYDDEEWAAQSAAARSATAGEHAGHGAAHQAPAAHAGHGPGHAPTPAQAAAPVTSGAGPSYNAGHGPLRAAPPALPWALKKQQAARSLPVGDKSGQTDGSRKALSLQPLAKRPAGIPSVPDVPKPDLRSLPAYGIVVTDGEKDIPGKDYLAFSANVWNAGPAQLVVDGFRTPGKAKMDAYQYFYDAKGKQVGYTPTGTMEWDPRPGHMHWHFTDFASYRLLKADKKEAVRSGKEAFCLANTDAIDYTVKNANWHPFNTDLSTACGEENSISVREVLDVGSGDTYSQDLPGQSFDITDVPNGTYYLQVLANPEKRLKETDLGNNSALRKIVLGGKPDARTVTVPAHDLVNAN

>Streptomyces_coelicolor_499342268

MTRTRTTRLRRPLLTGSTAVAAMAVTAGLVAATAEPAKAATGPKLSLIAATTSLTLTSWKEDPGVYLDLGTYLTAEGRPLELKVTRKSYKDPVTVTQTVYEGGKAKAKTLPKGTVKDFSGLPGFAEITVTDKAGKKVLNRTEDFCPNNASGRVRPDAPATSKYPESCPTNPFTLGSVWGVEQGWAANTYAGSYTEPVALAAGTYTAKVGVAKKYRDLFGIANKPATVKVTVVERSYEDDQGAAGSAASRSATAGEHTGHEAAHQAPAAHAGHGPGHAPTPAQAAAPVTSGAGASYNVGHGPLRAAPPALPWALKKQQAARSAPVGDKGGQTDGSRKAPALQPLAERPAGKASVPDVPKPDLRSLPAYGIVVTDGEEDIPGKDYLAFSANVWNAGPAQLVVDGFRSPGKAKMDAYQYFYDAKGKQVGYAPTGTMEWDPRPGHVHWHFTDFASYRLLKADKKEAVRSGKEAFCLANTDAIDYTVKNANWHPFNTDLSTACGEENSISVREVLDVGSGDTYSQDLPGQSFDITDVPNGTYYIQVLANPEKRLKETNLDNNSALRKIVLGGKPDARTVTVPAHDLVNAN

>Streptomyces_lividans_490069312

MTRTRTTRLRRPLLTGITAVAAMAVTAGLVAATAEPAKAATGPKLSLIAATTSLTLTSWKEDPGVYLDLGTYLTAEGRPLELKVTRKSYKDPVTVTQTVYEGGKAKAKTLPKGTVKDFSGLPGFAEITVTDKAGKKVLNRTEDFCPNNASGRVRPDAPATSKYPESCPTNPFTLGSVWGVEQGWAANTYAGSYTEPVALAAGTYTAKVGVAKKYRDLFGIANKPATVKVTVVERSYEDDQGAAGSAASRSATAGEHTGHEAAHQAPAAHAGHGPGHAPTPAQAAAPVTSGAGASYNVGHGPLRAAPPALPWALKKQQAARSAPVGDKGGQTDGSRKAPALQPLAERPAGKASVPDVPKPDLRSLPAYGIVVTDGEEDIPGKDYLAFSANVWNAGPAQLVVDGFRSPGKAKMDAYQYFYDAKGKQVGYAPTGTMEWDPRPGHVHWHFTDFASYRLLKADKKEAVRSGKEAFCLANTDAIDYTVKNANWHPFNTDLSTACGEENSISVREVLDVGSGDTYSQDLPGQSFDITDVPNGTYYIQVLANPEKRLKETNLDNNSALRKIVLGGKPDARTVTVPAHDLVNAN

>Streptomyces_violaceorubidus_663142094

MTRTRTTRLRRPLLAGTTAVAAMAVTAGLVAATAEPAKAATGPKLSLIAATNSLTLTSWKEDPGVYLDLGTYLTAEGRPLELKVTRKSYKDPVTVTQTVYEGGKAKAKTLPKGTVKDFSGLPGFAEITITDQKGKKVLNRTEDFCPNNASGRVRPDAPATSKYPESCPTNPFTLGSVWGVEKGWAANTYAGSYTDAVPLKAGTYTAKVGVAKKYRDLFGIANKPATVKVTVVERSYEDDQGAATPSAASRSATAGEHAGHGTAHRTPAAHADHGPGHAPTPAQAAAPVTSGASPSYNVGHGPLKAAPPALPWALKKKQAARSMPVGDTAGQTDGSRKAPALQPLAKRPAGKPAVPDVPKPDLRSLPAYGIVVTDGEKDIPGKDYLAFSANVWNAGPAQLVVDGFRSPGKSKMDAYQYFYDAKGKQVGYTPTGTMEWDPRPGHVHWHFTDFASYRLLKADKKEAVRSGKEAFCLANTDAIDYTVKNANWHPYNTDLSTACGEENSISVREVLDVGSGDTYSQDLPGQSFDITDVPNGTYYIQVLANPAKRLKETSLDNNSALRKIVLGGKPDARTVTVPAHELVNAN

>Streptomyces_sp_TOR3209_518157957

MTRPRTTRLRRPLLAGATAVTALAVTAGLVAASSEPADAAAKPELSLIAATGSLTLTSWKEDPGVYLDLGTYLTAEGTPLELKVTRKSYKDPVTVTQTVYEGGRAKAKKLPAGTVKDFSGLPGFVRVTLTDKAGRKVLDRTEDFCPNNASGRVRPDAPATSKYPESCPTNPFTLGSVWGVEKGWAANTYGGSYSAPVQLAAGTYTAKVGVAKKYRDLFGIADKPATVKVTVQERSYEDESGSAGAAGRSATAGGHAGHGAAHAPQTAHAGHGPGHAPTPARAAAPVTSGAGPTYNAGHGPLRAAPPALPWALKKDQAARATRVGDTSGQTDGSRKAPAGRPQPQRPAGKPSVPDVPKPDLRSLPAYGIVISDGERDVPGKDYLAFSANVWNAGPAQLVVDGFRSPGKAKMDAYQYFYDAKGKQVGYTPTGTMEWDPRPGHMHWHFTDFASYRLLKADQKEAVRSGKEAFCLANTDAVDYTVKNANWHPYNTDLSTACGEENAISVREVLDVGSGDTYTQDLPGQSFDITGVPNGTYYIQVLANPEKRLKETDLNNNSALRKIVLGGKPGKRTVTVPAHELVNAN

>Streptomyces_olindensis_640933403

MTRRRTTRLRRTLLAGTTAIAVTAVTAGIVAAAQEPAKAATGPELSLVAATTSVTLTTWKEEPGVYLDLGTYLIAEGTPLELKVTRKSYKDPVTVTQSVYEGGKARAKKLPTGTVKDFSGLPGFTEITLTDDAGRKVLTRSESFCPNNASGRVRPDAPSTSKYPESCPTNPFTLGSVWGVEKGWAANTYGGSYTEPVQLPAGTYTAEVGVAKKYRDLFGIADKPATVKVTVVERSVEDEESTTGAAPRSAKVGEHAGHAGHQAPTAHAGHGPGHAPAPAEAAAPVTSGAGPSYNVGHGPLKAAPPALPWALKKKQKSSLSPQAGDRGGQTDGSRQAPALEPLSKRPTGKPSVPDVPKPDLRSLPAYGITIADGGKDNPGKDYLAFSANVWNAGPAQLVVDGFRSPGKATMDAYQYFYDAKGKQVGYTPTGTMEWDPRPGHEHWHFTDFASYRLLKADQKEAVRSGKEAFCLANTDAIDYTVKNANWHPYNTDLATACGEENSISVREVLDVGSGDTYTQDLPGQSFDITDVPNGTYYIQVLANPEKRLKETDLGNNSALRKIELGGKPGARTVTVPAHDLVDAN

>Streptomyces_viridochromogenes_490093307

MTRRRTARLRRSLLAGTTAIAVTAVTAGIVAAAQEPAKAVTGPELSLIAATTSVTLTSWKEEPGVYLDLGTYLIAEGTPLELKVTRKSYKDPVTVTQTVYEGGKAKAKKLPTGTVKDFSGLPGFAEITLTDKAGKKVVTRAESFCPNNASGRVRPDAPSVSKYPESCPTNPFTLGSVWGVEKGWAANTYAGSYTEPVQLPAGTYTAKVGVAKKYRDLFGIADRAATVKVTVVERSYEDEEGTNGAAPRSAKAGEHAGHAAGHQAPTAHAAHGPEHAPAPAEAAAPVTSGAGPSYNVGHGPLKAAPPALPWALKKKQKSALSPQAGDSGGQTDGSRQAPALEPLSKRPTGKPSVPDVPKPDLRSLPAYGITISDGDKDIPGKDYLAFSANVWNAGPAQLVVDGFRSPGKAKMDAFQYFYDAKGKQVGYTPTGTMEWDPRPGHVHWHFTDFASYRLLKADKKEAVRSGKEAFCLANTDAIDYTVKNANWHPNNTDLATACGEESSISVREVLDVGSGDTYTQDLPGQSFDITDVPNGTYYIQVLANPAKRLKETNLDNNSALRKIVLGGKPGARTVTVPAHDLVNAN

>Streptomyces_sp_CNR698_654196847

MTTTRTTRLRRPLIAGTTAIAAIAAVAVTAGFAATASDSARAAAAGPDLRLIAASSSITLTSWKEDPGVYLDLGTYLTAEGTPLELKVTRKSYRDPVTITQTVYEGGKARAKALPNGTVKDFSGLPGFAEITVTDKAGKQVLRRAESFCPNNASGRVRPDAPATSKYPESCPTNPFTLGSVWGVEKGWAANTYAGSYTAPVELAVGTYTAKVSVAKKYRDLFGIANRPATVKVTVQERSFEDGRGAAARTAGSGEHAGHGAAHPAPSAAGAAHAGHGPGHAPAPAQAAAAVTSGAGPSYNVGHGPLKAAPPALPWALKKQQTARSARVGDTEGQTDGSRKAPALGPLAKRPAGKPSVPNVPKPDLRSLPAYGITISDGGEDVPGKDYLAFSANVWNAGPAQLVVDGFRSPGKATMDAYQYFYDAKGKQVGYTPTGTMEWDPRPGHMHWHFTDFASYRLLKADKKETVRSGKEAFCLANTDAVDYTVKNANWHPFNTDLATACGQENSISVREVLDVGSGDTYTQDLPGQSFDITSLPNGTYYIQVLANPENRLKETSTANNSALRKVVLGGKPGKRTVTVPAHELVNAN

>Streptomyces_sp_CNT302_517722584

MTTTRTTRLRRPLIAGTTAIAAIAAVAVTAGFAATASDSARAAAAGPDLRLIAASSSITLTSWKEDPGVYLDLGTYLTAEGTPLELKVTRKSYRDPVTITQTVYEGGKARAKALPNGTVKDFSGLPGFAEITVTDKAGKQVLRRAESFCPNNASGRVRPDAPATSKYPESCPTNPFTLGSVWGVEKGWAANTYAGSYTRPVELAVGTYTAKVSVAKKYRDLFGIANRPATVKVTVQERSFEDGRGAAARTSGSGEHAGHGAAHPAPSAAGAAHAGHGPGHAPAPAQAAAAVTSGAGPSYNVGHGPLKAAPPALPWALKKQQTARSARVGDTEGQTDGSRKAPALGPLAKRPAGKPSVPNVPKPDLRSLPAYGITISDGGEDVPGKDYLAFSANVWNAGPAQLVVDGFRSPGKATMDAYQYFYDAKGKQVGYTPTGTMEWDPRPGHMHWHFTDFASYRLLKADKKETVRSGKEAFCLANTDAVDYTVKNANWHPFNTDLATACGQENSISVREVLDVGSGDTYTQDLPGQSFDITSLPNGTYYIQVLANPENRLKETSTANNSALRKVVLGGKPGKRTVTVPAHELVNAN

>Streptomyces_sp_CNS615_516571836

MTTTRTTRLRRPLIAGTTAIAAIAAVAVTAGFAATASDSARAAAAGPDLRLIAASSSITLTSWKEDPGVYLDLGTYLTAEGTPLELKVTRKSYRDPVTITQTVYEGGKARAKALPNGTVKDFSGLPGFAEITVTDKAGKQVLRRAESFCPNNASGRVRPDAPATSKYPESCPTNPFTLGSVWGVEKGWAANTYAGSYTAPVELAVGTYTAKVSVAKKYRDLFGIANRPATVKVTVQERSFEDGRDAAARTSGSGEHAGHGAAHPAPSAAGAAHAGHGPGHAPTPAQAAAAVTSGAGPSYNVGHGPLKAAPPALPWALKKQQTARSARVGDTEGQTDGSRKAPALGPLAKRPAGKPSVPNVPKPDLRSLPAYGITISDGGEDVPGKDYLAFSANVWNAGPAQLVVDGFRSPGKATMDAYQYFYDAKGKQVGYTPTGTMEWDPRPGHMHWHFTDFASYRLLKADKKETVRSGKEAFCLANTDAVDYTVKNANWHPFNTDLATACGQENSISVREVLDVGSGDTYTQDLPGQSFDITSLPNGTYYIQVLANPENRLKETSTANNSALRKVVLGGKPGKRTVTVPAHELVNAN

>Streptomyces_sp_CNT372_517676014

MTRSHKARIGRAAIAGGAAIAVAAGVIAAAPDARQAAGPKLGLIAATPSVTLDSWKEDPGVYLDLGTYVTAENGPLELKVTRKSYKDPVVARQIVRQGKKTTTRTLPKGTVKDFGGLPGFTEIAITDTKGKQVLKRQESFCPNNASGRVRPDAPSTSKYPESCPVNPFTLGSVWGVEKGWASNTYAGFYASPVALKPGKYTAKVSVTKRYRDLFGIANKPQTIKVTVRERSWEDQGRAGAGTRGAAKHGDHHAAGHGGGAAHGGHGQDKTPGRAPDRAGTASGHAAHAEGGHTAAHPEGHPAVRAEAPEAPTTGAAPTYNVGHGPYAPAPPALPWAAKKAGLRTAQKPGDGPGRTDGSRLAPALKANAAPPKGKAKVPDVPKPDLRSLPAYGIGIGEGFDEAPGKDYLSFSANVWNAGPAQLVVDGFRKPGKDKMDAYQYFYDAKGKQVGYAPTGTMEWDPRPGHEHWHFTDFASYRLLKADKKEAVRSGKEAFCLANTDAVDYTVKNANWHPQNTDLSTACGQENSISVREVLDVGSGDTYTQDLPGQSFDITDLPNGTYWIQVLANPEKRLKETDLDNNSALRKIVLGGTKGARTVKVPAHDLVDAN

>Streptomyces_acidiscabies_498037009

MTSQQDRKQLKKRAALAAGAALTVVAAVAATAPGAGAATAAKPGQPQLKLVAASKAVVLDKYEGDPGVYLDLGTYLTVDNAPLEFKVTRKSYKDQIVAQQVLRDGKKVTTKTLPTGLVKDFKGLPDFIEVSVKDAAGKEVTTSKGTFCPNNASGRLRPDAPATSHYPQSCPTNPFTLGSIWGVEKGWAANTAAVDYDRPVNLPAGEYTAKATVAKKYRDLFGIPNDTATVKMTVREIKDGGGEGLRSQHTGHTATGTTEGANHHYGPRGADAPTPPALSHALEARGQAHHLGDGKGHTDGSRIAPGLKANAAKPTGKAGAPAGVPKPDLRSLPAWDISITDGEDGDVPGKDYLAFSANVWNAGPAPLVVDGFRKPGAELMDSYQYFYDANGKQVGYTPAGTMEWDPRIGHEHWHFTDFASYRLLSEDRTKEVRSGKEAFCLANTDAVDYTVKNANWHPYNTDLSTACGQQNSISVREVLDVGSGDTYTQYRPGQSFDVTGLPNGTYYIEVKANPANRLQETNLKNNVSLRKVVLGGKEGARTVSVPPVDLINAP

>Streptomyces_sp_303MFCol52_655408540

MALADEEPMTRSQDRKGLRRAAFAAGAALTVVAAVAGSAPGAGAAPASPAGTPKLKLVAASKAVTADRYEGNSGVYLDLGTYVTVDDAPLELKVTRKSYKDPIVAQQILRNGSKVTTKTLPAGLVKDFSGLPDFLEVSIKNAAGVEVQKSKSNFCLNNASGRLRPDAPATSHYPESCSTNPFTLGSVWGVEKGWAANTSAFDYDRPVDLAAGEYTVKVGVAKKYRDLFGIPDDRPTIALTVREISDGGEGGAGGGGGAGLTAHSATTGGGHGASHDMAGMEGMKGMEGMAGMGGGHHYGPRGADAPTPSALSHALEARGLAHHLGDGSGHTDGSRVAPALKSATKRPTGRAGVPANVPKPDLRSLPAWDIAVTDGEDGDVAGKDYLAFSANVWNAGPAPLVVDGFRKPGADLMDSYQYFYDAKGKQVGYAPAGTMEWDPRIGHEHWHFTDFASYRLLAADQKEIVKSGKEAFCLANTDAIDYTVKNANWHPYNTDLSTACGQQNSISVREVLDVGSGDTYTQYRPGQSFDITGLPNGTYYIQVVANPANRLQETDTKNNVSLRKVVLGGTDGARTVSVPPVDLINAP

>Streptomyces_avermitilis_499294626

MALADEEPMTSQKHRSRPRRVALAAGAALTVAVAGAGAAPGAGAASTAAKPKLKLIAASNSVTLERWEGEPGVYLDLGTYVTVDGAPLEFKVTRKSYKDPVVAQQIIRNGTSTQKKALPAGLVKDFSGLPGFLEVSVKNAAGAVVSKSKGTFCPNNASGRLRPDAPATSHYPESCATNPFTLGSVWGVEKGWASNSSTVDYDTPVDLPTGEYTAKVSVAKKYRDLFGIPNDQPTIKVTVREQSDGGGEGMTSSRSSAHHGGAHSGHSAHSAHHYGPRGADDPTPPALSHALEDRGTAHHLGDGRGHTDGSRIAPALKPAAKRPTGRAGVPANVPKPDLRSLPAWGIAITDGEDGDVPGKDYLAFSANVWNAGPAPLVVDGFRSPGKDLMDAYQYFYDAKGKQVGYTPTGTMQWDPREGHEHWHFTDFASYRLLSADQTKQVRSGKEAFCLANTDAIDYTVKNANWHPNNTDLSTACGEQNSISVREVLDVGSGDTYTQYRPGQSFDITGLPNGTYYIQVIANPEKRLQETNLNNNVALRKVVLGGTPGARTVTVPPHDLINAP

>Streptomyces_resistomycificus_662085488

AVTLDRYEWDSGVVLDLGTYVTVDDAPLEFKVTRKSYKDPVVARQILRDGTSTRTKTLPAGLVKDFAGLTGFLNVTLTDATGKEVSKTEGAFCPNNASGRLRPDAPATSHYPESCSTNPWTLGSVWGVEEGWAANSSQIDYDRPVDLPAGEYTAKVSVAERYRDLFDIPDDRPTIKVTVRENHGGGGAGLRAKASGSSAHHGGGHGAHTDAQASHHYGPRGADAPTPPALSHALEDRGAAHHLGDGPGHTDGSRIAPALTPNTKRPAGRAGAPANVPKPDLRSLPAWDIAITDGEDGDAPGKEYLAFSANVWNAGPAPLVVDGFRSPGKDLMDAYQYFYDANGKQVGYTPTGTMEWDPRLGHEHWHFTDFASYRLLSEDQTKEVRSGKEAFCLANTDAIDYTVKNANWKPRNTDLSTACGQQNSISVREVLDVGSGDTYTQYRPGQSFDVTDLPNGTYYIQVIANPEKRLQETNTDNNIALRKVILGGEPGARTVVVPPHDLINAP

>Streptomyces_davawensis_505468093

MTSQHQRKRVKHSALAASAALAVVAAVAATAPGAGAAQSGTPGTPKLKLIAASSAVTLDRWEGEGERGVFLDLGTYVTVDGAPLEFKVKRKSYKDPVVAQQVLRDGTKTTTRALPAGLVKDFSGLPGFLEVSVKNAAGQEVAKTKGSFCPNNASGRIRPDAPARSHYPESCPTNPFTLGSVWGVEKGWASNSSTVDYDKPLDLPVGQYTAKVRVAKKYRDLFGIPDDRPTIKVTVREETGGGGGMGLTARSGHGAHHAGGPAGDHYGPRGADAPTPGALSHALEDRGLAHHLGDGRGHTDGSRIAPALKAADKRPTGRAGVPANVPKPDLRSLPAWDIAITDGEDGDVPGKDYLAFSANVWNAGPAPLVVDGFRSPGKDLMDAYQYFYDASGKQVGYTPTGTMEWDPRIGHEHWHFTDFASYRLLSEDQTKEVRSGKEAFCLANTDAVDYTVKNANWHPYNTDLSTACGEQNSISVREVLDVGSGDTYTQYRPGQSFDITDLPNGTYYIQVIANPAKRLQETNTKNNVALRKVVLGGEPGKRTVKVPPHDLIDAR

>Streptomyces_turgidiscabies_493422467

MALADEEPMTTQQHSTSSSERNDSRRNTKRLQHSALAAGAALAVVVSVAGAAPGAGAAPSAAPTKPKLRLVAATDSVTAERWEGEPGVYLDLGTYVTVDDVPLEFKVTRKSYKDPVIAEQIIRKGGKAQAKRLPPGMVKDFAGLPGFLEVSFKNAAGQEVAKSKGTFCPNNASGRIRPDAPATSHFPESCSGNPFTLGGVWGVEKGWATNVSSVDYGNPVDLQAGEYTAKVSIGKMYRDFFGIPDDHPTIKVTVVPRSDDGEGDGGGGGVGLTARSSGQHAGGHGGHGGHASHGANGASGGGGSHGAAQLAAPHASHHYGPRGADMPTLPAFPNALVDLGTAHHLGDGPGHTDGSRVAPPLKAAAQRPTGRAGVPANVPKPDLRSLPAWDIAVTDGEDGDVAGKDYLAFSANVWNAGPAPLVVDGFRKPGKDLMDAYQYFYDANGKQVGYTPTGTMEWDPRIGHEHWHFTDFASYRLLSADQTKEVRSGKEAFCLANTDAIDYTVKNANWHPYNTDLSTACGEKNSLSVREVLDVGSGDTYTQYRPGQSFDITGLPNGTYYIQVIANPEKRLQETNLNNNVALRKVILGGTEGARTVTVPPHDLIDVK

>Streptomyces_ipomoeae_496647830

MTSQASHASSASTPEHRGSAGTPEPRRRLKRPALAALASLAVVAAVAGAAPGAGAAPAASTTPGTPQLKLIAATNAVVLDRYEWNDGVLLDLGTYITVDGAPLEFQVTRKSYKDPIVAKQILRDGKKTKTRTLPKGLVTDHGGLPGFLEISIKNTAGDEVLKTESTFCPNNASGRIRPDAPSTSHYPQSCSTNPWTLGSVWGVEKGWAVNTTQFRYDQTVNLPVGEYTAEVGVAKKYRDLFGIPDERPTVKVTVRQYEDGEGGGGVGLATTKSSAHHGGHGSGHAAQQDPSMPPTHGYGPRGEDVPMPPALSHALEDRGRASHLGDGPGHTDGSRIAPALKPAAKRPTGKAGAPANVPKPDLRSLPAWGIAVTDGEDGDVPGKDYLAFSANVWNAGPAPLVVDGFRKPGADLMDSYQYFYDASGKQVGYAPAGTMEWDPREGHEHWHFTDFASYRLLSEDQTKEVRSGKEAFCLANTDAIDYTVKNANWHPENTDLSTACGEESAISVREVLDVGSGDTYTQERPGQSFDITDLPNGTYYIQVIANPANRLQEADTSNNVALRKVVLGGTPGARTVQVPPHDLIDAP

>Streptomyces_bottropensis_491625747

MPPTMALADEEPMTSQMTSPTNSPNPGNDGAPEGAAVQTGQHRLSRLKRPGLAALASLTVVAAVAGAAPGAGAAPAATPGKPQLKLIAASKSVTLTRYEWDEGVQLQLGTYLTIDNAPLEFRVTRKSYKDPIVAQQILRNGKTVKTKTLPKGTVSDFSGLTGFLEVSVKDASGKEVLKKKQNFCPNNASGRIRPDAPSTNHYPQSCSTNPWTLGSVWGVEKGWAANTTTSYYGEDEVQLPEGEYTAKVGVAKKYRDLFGIPDTRPTIKVTVREDKGDEGDGGGAGLAPSTSSSSSHHGGHGGHGAGHSAQSAPAGQPAQGNHHYGPRGADAPTPPELPFALVDRGIAKHLGDGAGHTDGSRIAPALKANAKRPTGKAGVAKSVPKPDLRSLPAWDIAITDGEDGDVAGKDYLAFSANVWNAGPAPLVVDGFRKPGADLMDAYQYFYDAKGKQIGYAPTGTMEWDPREGHEHWHFTDFASYRLLSADKTKEVRSGKEAFCLANTDAIDYTVKNANWDPDNTDLSTACGSQDAISVREVLDVGSGDTYTQYRPGQSFEITDLPNGTYYIQVIANPENRLQETNHKNNVALRKVILGGTPGARTVKVPPHHLITAP

>Streptomyces_bottropensis_655392964

MALADEEPMTSQMTSPTNSPNPGNDGAPEGAAVQTGQHRLSRLKRPGLAALASLTVVAAVAGAAPGAGAAPAATPGKPQLKLIAASKSVTLTRYEWDEGVQLQLGTYLTIDNAPLEFRVTRKSYKDPIVAQQILRNGKTVKTKTLPKGTVSDFSGLTGFLEVSVKDASGKEVLKKKQNFCPNNASGRIRPDAPSTNHYPQSCSTNPWTLGSVWGVEKGWAANTTTSYYGEDEVQLPEGEYTAKVGVAKKYRDLFGIPDTRPTIKVTVREDKGDEGDGGGAGLAPSTSSSSSHHGGHGGHGAGHSAQSAPAGQPAQGNHHYGPRGADAPTPPELPFALVDRGIAKHLGDGAGHTDGSRIAPALKANAKRPTGKAGVAKSVPKPDLRSLPAWDIAITDGEDGDVAGKDYLAFSANVWNAGPAPLVVDGFRKPGADLMDAYQYFYDAKGKQIGYAPTGTMEWDPREGHEHWHFTDFASYRLLSADKTKEVRSGKEAFCLANTDAIDYTVKNANWDPDNTDLSTACGSQDAISVREVLDVGSGDTYTQYRPGQSFEITDLPNGTYYIQVIANPENRLQETNHKNNVALRKVILGGTPGARTVKVPPHHLITAP

>Streptomyces_scabiei_502767090

MALADEEPMTSQMTSPTNSPNSPGSDGAAQDRRSRLKRPGLAALASLTVVAAVAGAAPGAGAAPAAKPGTPQIKLIAASKSVTLTRWEGNSGVNLQLGTYLSVDNAPLEFQVTRKSYKDPIVAKQILRDGKTVKTRTLPAGTVDDFSGLTGFLEISVKDATGKQVAKTKGNFCPNNASGRIRPDAPSTNHYPQSCSTNPWTLGSVWGVEKGWATNTTGYDYDNTVDLPVGEYTAQVRVAKKYRDLFGIPDSKPTVKVTVRKDDDGGEGGEGLTASKSSSSHHGGHGGHGSGHSAQSAPAGAPALDGHHYGPRGADAPTPPQLPFALVDRGIAKHLGDGAGHTDGSRIAPALKANAKRPTGKAGVAKSVPKPDLRSLPAWDIAITDGEDGDVAGKDYLAFSANVWNAGPAPLVVDGFRKPGADKMDAYQYFYDAKGKQIGYTPTGTMEWDPREGHEHWHFTDFASYRLLSADQSKQVRSGKEAFCLANTDAIDYTVKNANWHPENTDLSTACGSEDAISVREVLDVGSGDTYTQYRPGQSFDITGLPNGTYYIQVIANPENRLQETNHKNNIALRKVVLGGTPGARTVKVPPHDLINAR

>Streptomyces_clavuligerus_490055567

MNTIPRARLWRSALAASAALAVTAGVVAAAPEDTARAAAPAGPKLSLLAAVPQMTWTRYPAEEGFPGGVFNADLGLHLAAEGSPFELRLKRPSYDKPIVLQQIIHEGKTKRVKNLPSGLVKDFRGLPGFIEVTYVNAAGKTVLKDSQNFCPNNASGRLRPDGPQQSRFPQSCPDNPLTVGSVWGVEKGWGTNTYSGHYYSSTEGLDLAAGTYTARATVAKKYRDLFGISGSSHSVKVTVEEYKDTEPGGPVGRSAAGPHHAAGHSGHGDGAGDHSGHGKAPAMTLAQATGGTGPMYNTGHTPYPPAPPALPWALRKSARTVANVGDRPGQTDGSRRAGGEKPNAKRPTGSPKVPDVPKPDLRSLPAFGIEIQPGENQGKPGRDFVAFSANVWNAGPAELVVDGFRAKGKDVMDAYQYFHDANGKQVGFTPAGSMEWDPREGHNHWHFLDFASYSLLKENKKEALRSGKEAFCLANTDAVDYTVKGANWFPSNTDLSSACDGPTRLSIRQRLDVGSGDTYVQDLPDQAFDITTVPNGTYWIQVKANPANRLKETSTTNNTALRKIVLGGKPGARTVTVPPIGLVDDHRPGTGGGGGRG

>Kribbella_catacumbae_521053909

MIINISRKFGRTAVAVTGAATLVALAAGAAGAASSQPRAAADAPLKLVAGSTEVTLDRYPDGGVLLDLGTHLVAGKNPIEVRATRKSYGDPIVASQLVNGKAKALPAGLVTDFGGLAKFLHLTVTDAKGKKVYDKDRTFCLNGEGSRTRPDAPATSPYPDGCTANPFTLGAVWGLQAGWSASTVGFDEAGVDLPVGKYTAKLTVNKAYRDLFKIGAYDSAVTLKVTVREAANCGHDGCRQAFAKAKAKLAAKAEGEPAKAAPKPHAQRPTGKASVPKGPKPDLRSLPAWQIVVAPGEAGTPSAGRDFLQFSANVWNAGPSTLVLDGFRQKGKDLMDAYQYFYDEKGKEVGYAKTGTMEWDARDGHAHWHFTDFARYSLLNAKQTEVVRSQKEAFCLAATDSIDYTVKNANWHPMNTDLHTACGSQGSLSVREVLDVGSGDTYVQSLPGQSFDITGLPNGTYYIEVAANPEKRLFESNLKNNVSLRKVILGGTLHHRTVKVPPVQLVDAP

>Kribbella_flavida_502684590

MLVTAGASGAAAQRTAESPLKLVAGSNSVTLERYQDDGVNLDLGTHLVAGKAPVEVRATRASYHDPIVAHQLVNGRPKALPKGAVTDFAGLGRFLHVTVTDATGKKVVDRDQTVCLNGEGSRTRPDAPDTSPYPDGCSANPFTVGAVWGLQGGWSASTFSYDTEPVDLPVGTYTAKVSVNRAYRDFFKIPASDASVSLALTVKEAEDCGHGTVEGCRAAAEGKAVKLLPGAAKEPDVARATGAAPTPNAQRPAGKASVPKGPKPDLRALPAWQISVQPGEQGTPNEGKDFLQFSANVWNAGPSTLVLDGFRQPGKDLMDAYQYFYDTSGKQVGFQKTGTMEWDARDGHNHWHFTDFARYSLLNATQSEVVRSQKEAFCLAATDAIDYTVKNANWHPMNTDLHTACGNQGSLSVREVLDVGSGDTYVQSLPGQSFDVTDLPNGTYYVQVVANPEKRLFEASTANNESLRKVVLGGTPQHRTVQVPPVGVIDAP

>Streptomyces_tsukubaensis_493388775

MSTTSRKRLWAGGLSVAAAVALAAGLVAAVPGAERSPGGFTAPDISLVVASPEVTLQQWEDADGRRYVDLGGLGAYVGVEGGPLELRATRALGAEQVRVQQFVHDGVRVIRKDLPAGLVTDLGGLPGFTEITFIGADGEAVATSRETFCPNNASARIDPGAVPVPRYPEQCHDGPFTLGMVWGVEKGWATNTSAGLTSVPLPLATGRYTARISVAEKYRELFGIADEPQTVKVNVTPPDPAPNPAVDPAPGRASAHSAHSAHSAHSGDSGALSGAAGRGTAEDSSYPPAVPEVPHALKQPARAALKLLGDGPGYTDGSRFAPRAKAAAARPAGPPVVPAHVPKPDLRALPVWDIAVETERRGEPEGRDHLAFASTTWNSGDSPLVVDGFRQSGKPLMDAYQYFYDARGRQAGHAPTGTMEWDPRVGHNHWHFTDFAAYRLLTEDRKEALRSDKEAFCLVNTDAVDYTLKRADWRPSGTDLSSACGQGEPQALSIRQSLAVGSGDTYSRDLPGQSFDITDVPNGTYWIQVVANPSGRLHEKSLANNNAYRKIVLGGRPGARTVTAAPLGADLTG

>Streptomyces_clavuligerus_490052869

MWTGGLCAASVLAVTTGVTVAAPGPERAAATQPEIRLMAAAPSVELTRYEGEPGEPPGISIDELGAFLTVRGAPLEFRAQRTPDYQRPITVRQIIRTGGKVTVKTLPQGLVKNFLGLPDFTETTFTDAKGRVVASQKAPFCPNNASARVEPGGEPERTYPDGCPSDPFTLGSVWGVHRGWATDAVDSEHISRLDLPVGTYTAKISVTKRYRDLLAVADRPLKVKVTVLPPEEGEPPAARHTRHGADHTGHGADHSGHGAMARGDGHGPDAAAPGAALDHLAGDSDSVYRAELPSVPHALRTTGRALPTSPGDGPGYSDGSRFAPPLKPAAKRPTGPAVRSAAGLKPDLRAVPAWDIAIQTAQDGHPEGQDHLGFASTTWNAGPGRMLVDGFRTPGKEGLMDAYQYFYDARGKQAGYARTGTMEWDPRPSHDHWHFTDYAAYRLLKADKKEVVRSDKEAFCLMNNAPVDYTVKNANMRPEDAMTSTCGLGEPDALAVRQSIEPGSGDTYTKETAGQSFDITTVPNGTYWIQVVANPNKALHETSLRNNTSFRKVVLGGTPGKRTVKVPPHGLVNAG

>Streptomyces_clavuligerus_490060947

MSTKGEDMNSISRKRLWTGGLCAASVLAVTTGVTVAAPGPERAAATQPEIRLMAAAPSVELTRYEGEPGEPPGISIDELGAFLTVRGAPLEFRAQRTPDYQRPITVRQIIRTGGKVTVKTLPQGLVKNFLGLPDFTETTFTDAKGRVVASQKAPFCPNNASARVEPGGEPERTYPDGCPSDPFTLGSVWGVHRGWATDAVDSEHISRLDLPVGTYTAKISVTKRYRDLLAVADRPLKVKVTVLPPEEGEPPAARHTRHGADHTGHGADHSGHGAMARGDGHGPDAAAPGAALDHLAGDSDSVYRAELPSVPHALRTTGRALPTSPGDGPGYSDGSRFAPPLKPAAKRPTGPAVRSAAGLKPDLRAVPAWDIAIQTAQDGHPEGQDHLGFASTTWNAGPGRMLVDGFRTPGKEGLMDAYQYFYDARGKQAGYARTGTMEWDPRPSHDHWHFTDYAAYRLLKADKKEVVRSDKEAFCLMNNAPVDYTVKNANMRPEDAMTSTCGLGEPDALAVRQSIEPGSGDTYTKETAGQSFDITTVPNGTYWIQVVANPNKALHETSLRNNTSFRKVVLGGTPGKRTVKVPPHGLVNAG

>Streptomyces_clavuligerus_497685028

MNSISRKRLWTGGLCAASVLAVTTGVTVAAPGPERAAATQPEIRLMAAAPSVELTRYEGEPGEPPGISIDELGAFLTVRGAPLEFRAQRTPDYQRPITVRQIIRTGGKVTVKTLPQGLVKNFLGLPDFTETTFTDAKGRVVASQKAPFCPNNASARVEPGGEPERTYPDGCPSDPFTLGSVWGVHRGWATDAVDSEHISRLDLPVGTYTAKISVTKRYRDLLAVADRPLKVKVTVLPPEEGEPPAARHTRHGADHTGHGADHSGHGAMARGDGHGPDAAAPGAALDHLAGDSDSVYRAELPSVPHALRTTGRALPTSPGDGPGYSDGSRFAPPLKPAAKRPTGPAVRSAAGLKPDLRAVPAWDIAIQTAQDGHPEGQDHLGFASTTWNAGPGRMLVDGFRTPGKEGLMDAYQYFYDARGKQAGYARTGTMEWDPRPSHDHWHFTDYAAYRLLKADKKEVVRSDKEAFCLMNNAPVDYTVKNANMRPEDAMTSTCGLGEPDALAVRQSIEPGSGDTYTKETAGQSFDITTVPNGTYWIQVVANPNKALHETSLRNNTSFRKVVLGGTPGKRTVKVPPHGLVNAG

>Nocardioides_sp_JS614_500079263

MIRSTSRSLALLASLALALPAVALSTPPATASSNPAGSPAGSGRAAEAVAPIALWAPHAVTASAYRKRTWTDLGLRLTAQGAPFELWSHRSSYDEAIRTVWHTADGDVALPAGSMSTFSGLDGFLRIDITPQRGGEPLHVVRKACLNGWSERVRPDAPARSGYPAGCWYNPFSLGSVQGIQDGWAAPILSQGRPFRLTPGSYTVTARIASKYAAVFGLGDADATRTVQLTVTAEDVGGGAGAPPATGRVAVPAARQPSGPQGRAPEAGPQPDLRSLPAWGIGLSENTNYLRFSATVWNAGDSPLVVDGFRRDGEDEMDAYQYFFDAAGEQTGYQPVGHLHWDPKPSHQHWHFEDFARYSLLDADQQETARSRKEAFCLANTDAVDLTVPAADWRPENTDLSTSCGDYSSLSIREVLAAGWGDTYAQYRAGQSFDIRGLPNGTYYIAVIANPENNLAEAATDNNVALRRIVLGGKPGHRTVRVPQVGIIDEEGYGGQG

>Nocardioides_sp_URHA0032_655222309

MRRSLLAAASLALAAPLLASVGPAATAESPKAAEVAPITLWAPSHVTAYAYRKRTWTDLGLRLIANGAPFELWSDRPSYNDPIQTVWRTSGGDVPLPAGSMSDFSGLDRFMSITVDPKKRGEDTLRMTKKGCLNGYSERVRVDAPARSPYPVGCWYNAYSLGSVQGIQTGWATPVLNQSRPLRIGPGEYSVTATIKRAYATVFGLTAEQATRTFDLTVTDESGIDRAGAAGRPSKPVAPAAHRPTGPTGRADEGGPEPDLQALPAWGISLNRKGTYLRFSATVWNAGDSPLVVDGFRRDGEDEMDAYQYFFDADGNQTGYQPVGAMEWDPRPSHSHWHFEDFARYSLLDAGKTEVSRSKKEAFCLANTDSVDLTVPAADWRPENTDLSTSCGDYSSLSIREVLVSGWGDTYTQYRAGQSFPIKDLPNGTYYVAVNANPEHNLVESSTDNNMSLRRIKLSGPPAHRKVTVPQVGIIAEEGYGGQG

>Nocardioidaceae_bacterium_Broad1_495634152

MVKPRTRTRQLVSAGIGLVAAALVLTTTVTANSGPASGATGKGTLKLVAAADTVQVEAWEGTAYFQPSVWLAAYDAAFEFRASHAAYDEPVKLTKTVIRGKKRTTTTAPASIVDGMKGFKDGLRVTLKTKSGKVILDDSRPLCPGGYDRQRVQTDGATGPVYPEFCGGNWFTKGVLYGIERGMAVPAVSDLEFETKGETRLTMTMSVSKAVADFLGLPNASRTAKQDLVIVDGCAEGGCETEGEGEMGIMAAHGADDDQGARTVVPRETLEGGENGRGRLPLGGALGIIKPGKPTKDTLPDLVSLPAWQISTEVDEGGTDRLNFAANEWNAGPAPMVVEGFRRGTSAVMDAYQMFYRDGKQVGVKRTGTMEFHEAPEHNHWHFLDFAKYELVDAKDKVVSTSGKQSWCLAPTDPVDLTVPGATLRPSQTGLQSACGEKSALWLRETMPTGWGDTYNQAQTQAFDLSKVKNGTYRIKVTVNPDGNLYERTRSNNVSYRTITIGGKAGARTVKVPPYQGVDTETWWGEEE

>Promicromonospora_sukumoe_518857434

MGVATVLALGLLGEGGATNAASAATTESATTTAPAAPTAAAKPGLRFAPASSTVQAYTYDGFAYLDSGVWLAAYGETHEFWAKRKKASAPVSLTRTVIRDGRRTTTKAPAAIVDGLNGFKNGLTVQVATTKGKVLAKDTRSLCVGGSDRQRVEPTGRTEPVYPAFCGGSWFTRSSVSGIEQGWAAKLDTYFELETTQKNLVMTVSISDPVADFLGLPAKGRTVKQQVEVVDECEIFDCGEVAEELMGTPAEGFSSVQDGDGASARAESLRAQSGHTGHGSGSGSTGAGHRTNLFTLGDAAHAGGDHPALGQRGSTPSNRKPAKDTLPDLVSAPAWQIGTEVDEAGTDRLTFNANEWNAGPAPLVVEGYRRGAGELMDAYQFFYKDGKEVGSTRTGTMEYHQAPEHNHWHFLDFASYELVTTKGKPVTPSGKQSWCLAPTDPVDLAVPGAVWRPEATGLDSTCGDRSAIWLREVLPAGWGDTYNQTQTQAFDLTKVKNGTYQIKITVNPNGALHERTRSNNVSYRTVVLGGKAGKRTVTVPPYEGVDTEVEVAPER

>Amycolatopsis_methanolica_516610010

MLRRIGLLALAAVLFVPGVASAEEELRPDLGMAPLTDLKVTTSPTGQQQLRFSATIVNVGRGPFEVEASRVSVDAPFRVVQRVARADGSRADVGVPAGLVYGGDGHDHWHIRDLETYQLVRLDGGVVSVAAKAGFCFYDTSSYRRSLPGAPRSKVYLESDCGDRGSLTVTMGLSVGWADRYASTLPDQFVDITGIPDGRYRLIATADAQGHFTEADRTNNATWVDLSLTSRNGRTSVRVLAHGPSA

>Amycolatopsis_sp_ATCC_39116_521088478

MLRRIGLLVLAALLFIPGVASAEEELRPDLGMAPLSDVKVTTSPAGQQQLRFSATIVNVGRGPFEVEASRVSVDAPFRVVQRVSRADGSRADVGVPAGLVYGGDGHDHWHIRDLETYQLVRLDGGVVSVAAKAGFCFYDTNSYRRSLPGAARSKVYSESGCGDRGSLTVTMGLSVGWADRYVSTLPDQFVDITGLPDGHYRLIATADAQGHFTEADRTNNATWVDLSLTSRKGRPSVRVLAHGPAA

>Amycolatopsis_thermoflava_654463633

MLRRIGPLVLAALLFVPGVASAEEELRPDLGMAPLTDVKVTTSPAGQQQLRFSATIVNVGRGPFEVEASRVSVDAPFRVVQRVSRADGSRADVGVPAGLVYGGDGHDHWHIRDLETYQLVRLDGGVVSVAAKAGFCFYDTSSYRRSLPGAPRSKVYSESGCGDRGSLTVTMGLSVGWADRYASTLPDQFVDITGIPDGRYRLIATADAQGQFTEADRTNNATWVDLSLTSRKGRTTVRVLAHGPAA

>Amycolatopsis_sp_ATCC_39116_654465224

MIRRLAPAVLAAALLTQVPGIASAAEPLQPDLGMARISDLKIATTASGQRQLRFSATIVNVGRGPFGLVADRASEASSFVVSQRVPQSDGSRVSVGVPASLVYGGDGHGHWHVRDLESYQLVRLDNGSKVGTSSKGGFCFFDTDAYRLTLPGAPQSSVYSPETCGHLDSLTVSMGLSVGWGDRYPWTLPDQYIDITGLANGQYRLIATADAQGLFVESNRANNSTWVDIAVTSRKSGTSVKILDYGPAA

>Nocardioides_sp_CF8_498535272

MCHDPGMPPAGSRITRQLAALSVVALLSGCGGDAEARWAEEPVGSALLPDFAPVPPSDIHTKQIEGAWTVEFSSTLVNVGEGDFHATADKQLDDSWVLTQDIEYDGGGAEHVTTDAQAVWGGDGHEHWHVERYVVYHLFALDEAGEVTGPARTDHKVGFCIYDFERAEVDLGSDEPTYDRKGCGEEDSTHLVMGLSPGWADHYNWDLPGQSIEIDGLADGDYRIFAVADEGTVFREETTENNETWVDFTLSSDPQGNRYALLGDVGPSPA

>Catelliglobosispora_koreensis_648516752

MRIVLSALLLAVFVPAPAHAASDVLPSLEPVRSDLARHDLRESGGKTYLRFTGTVANKGKGGLHVIGRRNARDLSLTAYQQIEQSGGGTREVKIGTIVYHDEHNHYHLDGVSRYRLFNASGDVVRAAPKVTFCLTDSEPVSGGGVPVYLQCTPSRDASTVKMGISPGWMDIYGKDLPGQSFDVTELMKLPKQDYTLEMTSNPLGLLHETNRDNPRTARVTVTLGS

>Deinococcus_pimensis_653299716

MAALLLAGALLLSGEGQRLAVPPVRVDEDGWLLPDLVVSPAAHLWVEVRDDEKGNRRLLHFATTVWNYGSGPLELVGDVPDDPAVSRVRAWQRVTRRGGATMDLRVGDFVYHPAHQHWHLNTFARYDLYALADGRPPRLVRTSGKVTFCILSSDRAPTTPVYPVDQEGCGLRRQVLEPGWGDTYGSYTPGQDIDVTSLPDGEYELRTTADPEGALRELDEDDNVSVTRLLLTGPSVRPLME

>Truepera_radiovictrix_502943269

MLVAYVLLGPLTMTHEERGRAEGALPMASWNLAEIEARNRAGLQHVAATGAAPSYTRETLPERLLPDLVPLEPSDLSTVGSRAEGNLRLKFTTLIWNAGLGPLETRGARNPETGELEVYQYVYRRVGEAASAGASPAALQTGGAQTGGTFPELRAEQGRWVGTFNYEHRHGHLHFDGFAHYGLWRVGEGGELVELIAENAKVGFCLMDIKHMASNLTHLESGLIEVPGGPVYAGCREDVQGISVGWGDEYLSLLLEQDLDLTDAPSGSYAVVVTTNPERRIEELDYDNNAAVTYFRLEDERVVWSSVDDGGGG

>Azohydromonas_australica_655953753

MLVAHIVPRLRGLAAALLGAQLVLLAGAAPPAHADPEAATLRYPDLSVIIPPGRMSVVGSGAERVFQYTHNTFNAGPGPLVIQPAYNASSGAYLGMQYVYAFEAGRWTLQRRVPVAGGFVFHAAHGHFHFPFARFGLYAVGSDGRPGRPVAVSAKNGFCIHDSFLYAPDLPNAGDLGNLGSCADPTSLQGLNIGAVDEYDRSDPGQSISLAGVPDGTYWLRAVVDPDDFLAESDESNNETDVLVRISGDSVTELRRVVPRLPPPPAVTLLSPASLSQVTGTVSLRAGSVAGSSVQFLLDGKPLGRRVAAPFTLAWNTSTVPDGAHWLAVQTTDPVSGRTGTSAVARVKVANGGTVAPQVRLTSPEVGATVSAVTALGATVASSSRITGVQFLMDGAPVGERLTAPPYVLYWDSRKVSDGAHEISARATDVHGLSGTSPAVRVTVDNSHPPAALRIDALVFRDGSGALATPAFSTTDKSDLLVAFVAYDGPADARQGARVSGAGLEWKLVMRANTQRGTSEIWAAKASWVLSRVSVTARPEASGHHGSLVVMAFSNAAGTGITGRSGAPEGAPEVYLPGISAGNWVFAVGNDWDRAVGRVPVAGQVLVHQRVDAAVGDTFWVQATARPSAANALVTIRDTAPTTDQWNYAAVEIVARRP

>Saxeibacter_lacteus_656114027

MLSLVAVGAATGTTPSVAAPAGTLHYPDLQNIIPPSDMSIVQTGTGRELRYTHLLYNAGSGPLEIKPLYNNASGDYQGLQRIYTHDAAGNWTIAKQVRIGGAFVYHAEHGHFHFPMAAFGLYTVAPGGGVGNPVTMSPKNGFCIADSYILDSTLPHSGTFGYSGGTCTDPTAIRGISVGGADEYDYRDPGQSVPIDGVPDGTYWFRALVDPFDYFQESNKANNETDVKVQISGTTVNTLETVHPDSTPPPATLTAPIDGSRVAGTVTLAAATPPVGSKGVQFIVDGAPVGSEVTAPPFSYSWDTNGLTNGAHWLAAQVTDANGTIGTSPIAEVTVSNGAAPPPPPPAGVLALDQQASSDGTGPVTAELSTGAAGDLLLAFVGGDGPGSAAESAKISGGGLQWSLVKRANSQSGTSEIWQATAPGRLSDSAITSTLGYGGYDQSLTVVAFSGAAGVGAAVGGGSRTGAPGVNLTTTADGSWVFGVGNDYDRGMPRTVGDGQRLVHQWVDSRTGDTFWAQSRVAPTQASGTLVPLGDPQPTGDRWNLAAVEVLAGSPPPPPDTTAPSVTISDPAAGATVSGNVPVAAVASDDVGVVGVAFRVDGSPIGVPVTSPPFMTTWATGSLSQGQHTLTAVASDAAGNTTTSAPVSVNVDNSAAPISQIRIDAQVSRDTSNTMTTPAFSTTQPGDVLVGFVAYDGPSSASQAATVSGAGLTWTLVKRSNIQAGTAEIWSAKATAVLTGVTVTAVPGRTGYHGSLTVIAFKNAGDVGVAGAASAPTGEPDVYLPGVEPGSWAFAVANDWDRAVARTPVSGQMLVHQRVDTSVGDTFWVQSTAAPSTTPGVVDIHDSAPANDRWNYSAVAVRPAATG

>Patulibacter_medicamentivorans_494847864

MSKLQIRRVAGVALAAIVVPGAAGVAIVAGEPAARPAPAAATATVNPCLGPEAATLRCPDLMMSRPFGIRVDRRARRGRTLLRAGNSIDSVGAGPAELRGRRTRSGWMAARQYVDRVGGGHLKLDNGGRLQFKKAHLDRRWWKFHDAARFELWTLDAQGERAERVRVGPKVAYCLRDLERTEPHLARSPRRAVYPACSTDRKRRRATLGTSPGWSDVYPPDYPEQWIDVSGLRGCFAYVHIADPGNAILESDEENNQAQVIVRLPFHRKDRRGGCPGRDFGRRYVERDAGDGY

>Rudanella_lutea_518835221

MFLVFSLPPTVGQGQTFGAQGGPIRDFNGVAKADTFPIRVQGLPIRINRTFGLAKVCLSVFHARSSDIKVELVNPAGASIWLTNRNGGDNGQHYANTCFRSNGFSGYIHLGTAPFEGEYIPDGRFTFLNNDTDPNGLWYLLVTDLRAGVRGNLNTVSLAFETDPTPNAGQPPCSFENPAYCQCPPGTNCELLPDLVILPRFTQTQIKEYAWNDPNYPGQLRFAASIGNIGDGPLETWGKQEWYCGDKRVDSTLRCPDGSYARQRIYQRIYQKKGDTLIATDRPAGTNYYDNQPGHNHYHVDDWVEFRLVKITPGTRASPRRRVIAKGRKVSYCLFDSGICNNSDSLCTCNGTVYGERNLPNYGLGNYGECKSMKQGISVGGYDTYGVMYEGQFIKLPRGLPKGTYQLEIEIDPTGSIRERNRANNLFTMPIQLSKQ
