# Supplementary material for: Origin and evolution of lysyl oxidases
Source: Sci Rep. 2015 May 29;5:10568. doi: 10.1038/srep10568 (PMC4448552; doi:10.1038/srep10568)
Supplement: File S6 [file srep10568-s3.doc]

>Hsap_ENSP00000264094

MRPVSVWQWSPWGLLLCLLCSSCLGSPSPSTGPEKKAGSQGLRFRLAGFPRKPYEGRVEIQRAGEWGTICDDDFTLQAAHILCRELGFTEATGWTHSAKYGPGTGRIWLDNLSCSGTEQSVTECASRGWGNSDCTHDEDAGVICKDQRLPGFSDSNVIEVEHHLQVEEVRIRPAVGWGRRPLPVTEGLVEVRLPDGWSQVCDKGWSAHNSHVVCGMLGFPSEKRVNAAFYRLLAQRQQHSFGLHGVACVGTEAHLSLCSLEFYRANDTARCPGGGPAVVSCVPGPVYAASSGQKKQQQSKPQGEARVRLKGGAHPGEGRVEVLKASTWGTVCDRKWDLHAASVVCRELGFGSAREALSGARMGQGMGAIHLSEVRCSGQELSLWKCPHKNITAEDCSHSQDAGVRCNLPYTGAETRIRLSGGRSQHEGRVEVQIGGPGPLRWGLICGDDWGTLEAMVACRQLGLGYANHGLQETWYWDSGNITEVVMSGVRCTGTELSLDQCAHHGTHITCKRTGTRFTAGVICSETASDLLLHSALVQETAYIEDRPLHMLYCAAEENCLASSARSANWPYGHRRLLRFSSQIHNLGRADFRPKAGRHSWVWHECHGHYHSMDIFTHYDILTPNGTKVAEGHKASFCLEDTECQEDVSKRYECANFGEQGITVGCWDLYRHDIDCQWIDITDVKPGNYILQVVINPNFEVAESDFTNNAMKCNCKYDGHRIWVHNCHIGDAFSEEANRRFERYPGQTSNQII

>Mmus_ENSMUSP00000000707

MRAVSVWYCCPWGLLLLHCLCSFSVGSPSPSISPEKKVGSQGLRFRLAGFPRKPYEGRVEIQRAGEWGTICDDDFTLQAAHVLCRELGFTEATGWTHSAKYGPGTGRIWLDNLSCRGTEGSVTECASRGWGNSDCTHDEDAGVICKDQRLPGFSDSNVIEVEHQLQVEEVRLRPAVEWGRRPLPVTEGLVEVRLPEGWSQVCDKGWSAHNSHVVCGMLGFPGEKRVNMAFYRMLAQKKQHSFGLHSVACVGTEAHLSLCSLEFYRANDTTRCSGGNPAVVSCVLGPLYATFTGQKKQQHSKPQGEARVRLKGGAHQGEGRVEVLKAGTWGTVCDRKWDLQAASVVCRELGFGTAREALSGARMGQGMGAIHLSEVRCSGQEPSLWRCPSKNITAEDCSHSQDAGVRCNLPYTGVETKIRLSGGRSRYEGRVEVQIGIPGHLRWGLICGDDWGTLEAMVACRQLGLGYANHGLQETWYWDSGNVTEVVMSGVRCTGSELSLNQCAHHSSHITCKKTGTRFTAGVICSETASDLLLHSALVQETAYIEDRPLHMLYCAAEENCLASSARSANWPYGHRRLLRFSSQIHNLGRADFRPKAGRHSWVWHECHGHYHSMDIFTHYDILTPNGTKVAEGHKASFCLEDTECQEDVSKRYECANFGEQGITVGCWDLYRHDIDCQWIDITDVKPGNYILQVVINPNFEVAESDFTNNAMKCNCKYDGHRIWVHNCHIGDAFSEEANRRFERYPGQTSNQIV

>Mmus_ENSMUSP00000098815

MRAVSVWYCCPWGLLLLHCLCSFSVGSPSPSISPEKKVGSQGLRFRLAGFPRKPYEGRVEIQRAGEWGTICDDDFTLQAAHVLCRELGFTEATGWTHSAKYGPGTGRIWLDNLSCRGTEGSVTECASRGWGNSDCTHDEDAGVICKDQRLPGFSDSNVIEVEHQLQVEEVRLRPAVEWGRRPLPVTEGLVEVRLPEGWSQVCDKGWSAHNSHVVCGMLGFPGEKRVNMAFYRMLAQKKQHSFGLHSVACVGTEAHLSLCSLEFYRANDTTRCSGGNPAVVSCVLGPLYATFTGQKKQQHSKPQGEARVRLKGGAHQGEGRVEVLKAGTWGTVCDRKWDLQAASVVCRELGFGTAREALSGARMGQDVSKRYECANFGEQGITVGCWDLYRHDIDCQWIDITDVKPGNYILQVVINPNFEVAESDFTNNAMKCNCKYDGHRIWVHNCHIGDAFSEEANRRFERYPGQTSNQIV

>Ggal_ENSGALP00000021613

MAASCVILTTRAPGEAALAGARASLPRSSQGRTLSPGCAVGVSLESCRHCCQWGTAVTSFATHLRSSHPDSCTSCTHCCPRTRESCTKCAGRVWTVTPALQRSSQPTPTYWVTGCSGRSTALLPHKAAARAAARAATLPLPGAAGHGPASAGHGCWLQSGCQPAECPQNQRAMGRCGTRAWQELLVLLGGLWMWGSGAQPTPPSPTSSSVPPLKFRLAGYPRKHNEGRIEVFYNEEWGTICDDDFTLSNAQVLCRQLGFVGATGWAHSAKYGKGVGRIWLDNVNCAGNEKSIRDCKHRGWGNSDCSHEEDAGVICKDERIPGFKDSNVIETEQSHVEEVRLRAVVSGAQRRLPVTEGIVEVRYKDGWAQICDEGWDSKNSHVICGMMGFPTEKKVNRNFYKRLKRAARTRGRSMRPGGRLASKSQPKQKRREDVGSRKRLFAERQQLNYRLHSVSCTGTEVHISMCTFQFYRGNSSAACSTGMPAVVSCLPGPLFATSSTQKKKQRQQQQSQPRIRLKGGAKAGEGRVEVLKNNEWGTICDDRWNLLSASVVCRELGFGSAKEALTGARMGQGMGPIHLNEVQCLGTEKSLWSCPFKNITQEDCKHTEDAAVRCNIPYMGYENLIRLSGGRSRFEGRVEVAVGAAEGDEPRWGLVCGEGWGTLEAMVACRQLGLGFANHGLQIRLAGGRTEFEGRVEVKRGSKWGTVCSDGWTTKEAMVVCRQLGLGYSLHAVTETWYWDASNVTEMVLSGVKCAGHELSLSHCQHHGSSLNCRNTGTRFAAGVICSETASDLLLHAPLVQETAYIEDRPLHMLYCAAEENCLSSSARHANWPYGHRRLLRFSSQIHNNGRADFRPKAGRHSWVWHECHRHYHSMDIFTHYDILTPNGTKVAEGHKASFCLEDTECEEDVAKRYECANFGEQGITVGCWDLYRHDIDCQWIDITDVKPGNYILQVVINPNFEVAESDFTNNAMKCNCKYDGHRIWVHSCHIGDALSEEASKRFEQYPGQLNNQIS

>Drer_ENSDARP00000105131

MVMSGVKCTGDEMSISQCQHHRTVNCQKAAARFAAGVICSETASDLVLNAPLVQQTTYIEDRPLHMLYCAAEEDCLSKSAASANWPYGHRRLLRFSSQIHNIGRADFRPKAGRHSWVWHACHGHYHSMDIFTHYDLMSANGTKVAEGHKASFCLEDTDCDEGVSKRYKCANFGEQGITVGCWDLYRHDIDCQWIDITDVKPGNYILQVVINPNYEVSESDFTNNAMKCNCKYDGHRIWVHNCHIGDAFSEEAEKKFEKYPGQLNNQIS

>Drer_ENSDARP00000109545

MELHQWCRHIIVFLLNVWIPSCFAQTTPPARSSPTPTPQTADNPDSLKFRLSGFPRKHNEGRIEVFYKGEWGTICDDDFSLANAHVLCRQLGFVSATGWTHSAKYGKGAGKIWLDNVQCSGSERSVSVCKSRGWGNSDCTHDEDAGVICKDERLPGFVDSNVIEVQVDENRVEEVRLRPVFTTATKRMPVTEGVVEVKNKDGWAQICDIGWTPKNTHVVCGMMGFPHEKKVNKNFYKLYAERQKNFFLVHSVACLGTEVHLAACPLEFNYGNATESCPGGMPAVVSCVPGPLYTQSPTMKKKLKMPPTTRLKGGAKYGEGRVEVLKGSEWGTVCDDRWNLVSASVVCREMGFGSAKEALTGASMGKGLGPIHMNEVQCTGNERSLWSCRYKNITAEDCKHTEDASVRCNVPYMGYEKTVRILGGRTRYEGRVEVLHREADGTLRWGLICGEGWGTQEAMVLCRQLGLGYANHGLQVRLSGGRSPYEGRVEVRVGQRWGSVCSEGWSTKEAMVLCRQLGLGFSMHAITETWYWDSSNVTDMVMSGVKCTGDEMSISQCQHHRTVNCQKAAARFAAGVICSETASDLVLNAPLVQQTTYIEDRPLHMLYCAAEEDCLSKSAASANWPYGHRRLLRFSSQIHNIGRADFRPKAGRHSWVWHACHGHYHSMDIFTHYDLMSANGTKVAEGHKASFCLEDTDCDEGVSKRYKCANFGEQGITVGCWDLYRHDIDCQWIDITDVKPGNYILQVVINPNYEVSESDFTNNAMKCNCKYDGHRIWVHNCHIGDAFSEEAEKKFEKYPGQLNNQIS

>Drer_ENSDARP00000103878

MDIFTHYDLMSANGTKVAEGHKASFCLEDTDCDEGVSKRYKCANFGEQGITVGCWDLYRHDIDCQWIDITDVKPGNYILQVVINPNYEVSESDFTNNAMKCNCKYDGHRIWVHNCHIGDAFSEEAEKKFEKYPGQLNNQIS

>Acar_ENSACAP00000008669

MGRHWALLGLLWGAWLWAGLAAQRSTPSPPRQTTPTPGGATPAPKLQFRLAGYPRKHNEGRIEVFYRQEWGTICDDDFSLANAEVLCRHLGFTGATGWAHSAKYGKGVGRIWLDNLNCGGSEKSVADCPSRGWGNSDCSHEEDAGVICKDERIAGFVDSNVIETEQNQLEEVRLRTVVSHAKRPLPVAEGIVEVRHRNAWAQICDENWSSRNSRVVCGMLGFPAEKKVNRNFYKLSLERQQNQLNFRLHSVACTGNEVHLSLCSFEFYKGNNTGACKGGMPAIVSCVPGPLFAAGSNPQAKKKRRQQQQNDRQQCVRLKAGAKPGEGRVEVLKNGEWGTVCDDRWNLISASVVCRELGFGSAKEALTGARMGQGFGEIYMNEVQCLGSEKSLWSCPHKNITQEGCKHSEDAAVRCNIPYMGYETMIRLSGGRSRFEGRVEVALGNGGLGTRRWGLICGDNWGTLEAMVACRQLGLGYANHGLQETWYWDASNVTEMVLSGVKCAGHEMALSQCQHHTTVTCRKTNTRFAAGVICSETASDLLLHAPLVQETAYIEDRPLHMLYCAAEENCLSSSARNATWPYGYRRLLRFSSQIHNNGRADFRPKAGRHSWVWHECHGHYHSMDIFTHYDLLTPNGTKVAEGHKASFCLEDTECDEDVAKRYECANFGEQGITVGCWDLYRHDIDCQWIDITDVKAGNYILQVVINPRYEVAESDFTNNAMKCNCKYDGHRIWIHNCHIGDALSEEANRRFEQYPGQLNNQIA

>Lcha_ENSLACP00000013690

MPVVVSCVPGPLFSHNTSQRKKIQAEPRVRLKGGAKLGEGRVEVLKNSEWGTVCDDRWDLLAASVVCRQLGFGSAKEALTGARMGQGMGPIHMNEVQCSGYEKSIWNCQFKNITEEDCQHPEDAAIRCNIPYMGFESSIRLSGGRTRFEGRVEVLVGLDGNERQKKWGLICGEGWGTMEAMVVCRQLGLGYANHGLQETWYWDASNLTEMVLSGVKCNGDEMSIHHCQHHTESINCKRTGTRFAAGVICSERGGVMLLRLSQISPPTYVERRRDSQLSPSPRESCLTHTSLGGGWQGHDPRFGNYLYSCKRKMTRPPPIKRSSFTNTSTHYHSMDIFTHYDVLTLNGTKVAEGHKASFCLEDTECQEGVSKRYECANFGEQGITVGCWDLYRHDIDCQWIDITDVKPGNYILQVVINPNYEVAESDFTNNGMKCNCKYDGHRIWVHNCHIAGDAFSEEAEKKFEQYPGQLNNQIQ

>Drer_ENSDARP00000054865

MLRSELRDMVVAMVLWGILLPFCLSQTTSPSQDGKIKFRLAGYPRKHNEGRIEVFYNREWGTICDDDFTLANAHVLCRQLGFVEALSWSHSAKYGPGSGKIWLDNVICGGSENSIEKCVSRGWGNSDCTHQEDAGVICKDERLPGFAESNIIEMQVDEKRMEKIRLRPLKGAHAGRLPVTEGVVEVKFKEGWGHICNTGWTIKNSRVVCGMMGFPSQRSVGKKPNSLKSAYRIHSVTCSGNEAHLSACTMEFSRANSSAPCPGGGAAVVSCVPGLQFTQGRVRKAKLNPVPQMRLKGGARAGEGRVEVLKGSEWGTVCDDHWNLQSASVVCRELGFGTAKEALTGARMGQGMGPIYMNEVQCGGDEKSLWDCPHQSITAEDCKHTEDASVICNIPYMGFEKLMRLTGGRTRLEGRVELLLPAGGGVRDWGLICGDGWTSREAMVVCRQLGLGHASSGLRETWYWDSSNVTEMVMSGVKCKGDEMTLTDCQHHSVVSCKRAGAQFSAGVICSDMASDLVLNAPLVEQTVYIEDRPLHLLYCAAEENCLAKSAAQASWPYGHRRLLRFSSEIHNIGKADFRPRLGRHSWVWHECHRHYHSMDIFTYYDLLSLNGTKVADGHKASFCLEDTECHEGVSKRYECANFGEQGITVGCWDLYRHDIDCQWIDITDVSPGNYILQVIINPNFEVAESDFTNNAMRCNCKYDGHRVWLHKCHLGDSFSEEAEKEFEHYPGQLNNKIS

>Pema_ENSPMAP00000004999

VLMSGVQCSGTEMSLAHCRHHGSRVKCKRGGGRYAAGVVCTESAPDLVLNSRIVQESAYLEDRPLHLLYCAAEENCLSASASAMNWPYGSRRLLRFSSQIHNVGRTDFRPKAGSHAWGWHVLHGHYHSMEVFTHYDLLTLNGTKVAEGHKASFCLEDTSCEPGVQKRYECANFGDQGIAVGCQDIYRHDIDCQWVDVTDVAPGDYIFQVVINPNYEVSETDFTNNAMKCNCKFDGHRVWMYNCHNGDAFSAEVEKLFDLYPGENNLIS

>Pema_ENSPMAP00000004994

SQIRLTGGRNEWEGRLEVRRSKRWGTVCGDGWTTLEAMVACRQLGLGFAVHALTETWYFQGDSGVEEVLMSGVQCSGTEMSLAHCRHHGSRVKCKRGGGRYAAGVVCTESAPDLVLNSRIVQESAYLEDRPLHLLYCAAEENCLSASASAMNWPYGSRRLLRFSSQIHNVGRTDFRPKAGSHAWWHQCHRHYHSMEVFTHYDLLTLNGTKVAEGHKASFCLEDTSCEPGVQKRYECANFGDQGIAVGCQDIYRHDIDCQWVDVTDVAPGDYIFQVVINPNYEVSETDFTNNAMKCNCKFDGHRVWMYNCHNGDAFSAEVEKLFDLYPGENNLI

>Pema_ENSPMAP00000007607

MKLPLITLPLLLLLLIIHLCLAHELLRRRSQRRENGRVIRQIQRPRQSSQARQPSQAGQPGQPGQPGQPGQPGQPGQSGQPGEQANGGYKLRLAGYPRKPFEGRVEVFHQGEWGTVCDDDFSMEAANVVCQEAGFIGANGWAHSAKYGRGIGRIWLDNVHCRGSEQSLAECVSRGWGISDCNHDEDVGVVCKETRLKGTPEFNVIEVPIGGGVIQCEDVRLRPILPAAKKRLPVTEGVVEVRYMGSWRHVCDENWTPRNSRVICGMMGFPAERKVNRVLYRHFSERQQERPSFLVHSLNCTGTESHITACQHDLLPLGPKAHNSCLAGMPAIVSCVPGPAYSPGVAFKKIFKSEVPMVRLKGGAMTGEGRVEVQRGGEWGTVCDQRWSTVSASVVCRELGFGSAKEALTSGRMGQAMGPIHMAMVQCTGQERSIFDCPHGNASDHGCGHHEDAAVRCHVPAMGFEKRNFRQCAKGFHNSWNWVDVNLGFCPVRELDSGLSEGWGMLEASVACRQLGLGYASMPPLQETWYFEGSPLHDQVVMSGVKCVGSEMSLQYCRHHNARIPKRSVAPDLVLNAPLVQVSSYLEDRPLHLLYCAAEEDCLAQSARNMHWPYGQRRLLRFSSEIHNLGRADFRPKAGRHSWVWHECHRHYHSMEVFTHYDILSTNGTKVAEGHKASFCLEDTDCQEGGQKVYECANFGEQGITVNCHDTYRHDIDCQWVDVTDLKPGNYIFQVVLNPNYEVAETDFTNNAMKCNCKYDGHRIWMYNCHVGDAFSDEVEKIFENYPGQLNNLISSF

>Drer_ENSDARP00000064700

MLALWSISFVLLCSWRLSYAQYEHLGFAIAYQEPEQDLYTPPELPADTPRIQLRLAGEKRKHNEGRVEVFYEGEWGTVCDDDFTIHAAQVICRELGYFEAISWSPSSKYGKGEGRIWFDNVHCKGKEKSLAQCESNGIGVSDCKHSEDVGVVCSDKRIPGFKFVNTLTNNINSLNIQVEDVRIRPILASYRKRIPVTEGYVEVKDGGKWKQICDDEWTQMNSRVICGMFGFPGQKRYNTRVYKMFARRRKPSYWDYTINCTGKEAHLSSCTLGHTLSNSTCEEGTPVVVSCIPGRAFAPTPMTGYKKAFRQEQPLVRLRGGAVVGEGRVEVLKNGEWGTICDDNWNLLAATVVCRELGFGSAKEALSGGQLGQGMGPVHMNEVQCSGFEKSVTECSFNMEKDSEGCSHEEDAGVKCNVPAMGFQQRLRLSGGRNPFEGRVEVLVERNGSLVWGTVCGEGWTTMEAMVVCRQLGLGFASNAFQETWYWPGAVNADAVVMSGVRCAGTEMSLSHCLHHGEYLSCPKGGGRFAAGVSCSETAPDLVLNPQVVEQTTYLEDRPMFMLQCAYEENCLASTSSATPANSPRRLLRFSSQIHNNGQSDFRPKISRENWVWHDCHRHYHSMEVFTHYDLLSTNGTKVAEGHKASFCLEDSECDEGIEKRYECANFGEQGITVGCWDTYRHDIDCQWVDITDVKPGDYIFQIVINPNYEVAESDYTNNIVKCRCRYDGHRIWMYNCHIGGSFSAETEDTFPGLINNQVTHR

>Drer_ENSDARP00000118755

MLALWSISFVLLCSWRLSYAQYEHLGFAIAYQEPEQDLYTPPELPADTPRIQLRLAGEKRKHNEGRVEVFYEGEWGTVCDDDFTIHAAQVICRELGYFEAISWSPSSKYGKGEGRIWFDNVHCKGKEKSLAQCESNGIGVSDCKHSEDVGVVCSDKRIPGFKFVNTLTNNINSLNIQVEDVRIRPILASYRKRIPVTEGYVEVKDGGKWKQICDDEWTQMNSRVICGMFGFPGQKRYNTRVYKMFARRRKPSYWDYTINCTGKEAHLSSCTLGHTLSNSTCEEGTPVVVSCIPGRAFAPTPMTGYKKAFRQEQPLVRLRGGAVVGEGRVEVLKNGEWGTICDDNWNLLAATVVCRELGFGSAKEALSGGQLGQGMGPVHMNEVQCSGFEKSVTECSFNMEKDSEGCSHEEDAGVKCNVPAMGFQQRLRLSGGRNPFEGRVEVLVERNGSLVWGTVCGEGWTTMEAMVVCRQLGLGFASNAFQETWYWPGAVNADAVVMSGVRCAGTEMSLSHCLHHGEYLSCPKGGGRFAAGVSCSETAPDLVLNPQVVEQTTYLEDRPMFMLQCAYEENCLASTSSATPANSPRRLLRFSSQIHNNGQSDFRPKISRENWVWHDCHRHYHSMEVFTHYDLLSTNGTKVAEGHKASFCLEDSECDEGIEKRYECANFGEQGITVGCWDTYRHDIDCQWVDITDVKPGDYIFQIVINPNYEVAESDYTNNIVKCRCRYDGHRIWMYNCHIGGSFSAETEDTFPGLINNQVTHR

>Drer_ENSDARP00000107052

GRIWLDNVHCTGRENSLAACPSNGFGVSDCRHSEDVGVICNQKRIPGHRFINIMNNNVETLEERVEEIRIRPISSHLKRIPITEGYVEVKERGKWRQICDEEWTPLNSRVVCGMYGFPGEKNYNNKVYRSLSMRKKKNYWGFLVNCTGNEAHMSSCRLGKALEPKRNGTCGRGLPVVVSCVPGRAFAPSSSIGFRKAYRPEQPLVRLRGGANVGEGRVEVLKNGVWGTVCDDNWNLKAATVVCRELGFGSAKEALTGAKLGQGMGPVHMNEVECSGFEKSLTDCYFNNDALGCSHEEDAAVRCNVPAMGFQKRIRLSGGRNPFEGRVEVLAEKNGSLVWGTVCSENWGIIEAMVVCRQLGLGFASHAFQETWYWAGDANADNVVMSGVRCSGTEMSLPQCLHHGKHINCPKGGGRFAAGVSCSDTAPDLVLNAQLVEQTTYLEDRPMYALQCALEENCLSSTARKNDHSSYRRLLRFSSQIHNVGQSDFRPKLGYHAWTWHECHRHYHSMEVFTHYDLLSLNGTKVAEGHKASFCLEDTHCDEGISKRYHCANFGEQGITVGCWDTYRHDIDCQWIDVTDVKPGDYIFQVVINPNYDVAESDYTNNVMKCKCRYDGYRIWTYSCHIGGSRSSDMDEYSGMSNQLNHLR

>Drer_ENSDARP00000064612

MAVSSALCIFSLLVLAQAQSELQQPKIELRLAGDKRKHYEGRLEVFYNNEWGTVCDDDFSIEAAHVACRQLGFLGAVAWSPSAKFGQGEGRIWLDNVHCTGRENSLAACPSNGFGVSDCRHSEDVGVICNQKRIPGHRFINIMNNNVETLEERVEEIRIRPISSHLKRIPITEGYVEVKERGKWRQICDEEWTPLNSRVACGMYGFPGEKNYNNKVYRSLSMRKKKNYWGFSVNCTGNEAHVSSCRLGKALEPKRNGTCGRGLPVVVSCVPGRAFAPSSSIGFRKAYRPEQPLVRLRGGANVGEGRVEVLKNGVWGTVCDDNWNLKAATVVCRELGFGSAKEALTGAKLGQGMGPVHMNEVECSGFEKSLTDCYFNNDALGCSHEEDAAVRCNVPAMGFQKRIRLSGGRNPFEGRVEVLAEKNGSLVWGTVCSENWGIIEAMVVCRQLGLGFASHAFQETWYWAGDANADNVVMSGVRCSGTEMSLPQCLHHGKHINCPKGGGRFAAGVSCSDTAPDLVLNAQLVEQTTYLEDRPMYALQCALEENCLSSTARKNDHSSYRRLLRFSSQIHNVGQSDFRPKLGYHAWTWHECHRHYHSMEVFTHYDLLSLNGTKVAEGHKASFCLEDTHCDEGISKRYHCANFGEQGITVGCWDTYRHDIDCQWIDVTDVKPGDYIFQVVINPNYDVAESDYTNNVMKCKCRYDGYRIWTYSCHIGGSRSSDMDEYSGMSNQLNHLR

>Ggal_ENSGALP00000000547

MEGFLGFNHNHCFIVLFFVSLSLAQYEHWPYLPGYPEPPPQVYQPPRRPADVPKIQLRLAGQKRKHNEGRVEVFYNGEWGTVCDDDFSIHAAHVICRELGYVEAVSWLPSSKYGKGEGKIWMDNVHCNGKEATLAACTSNGWGVTDCKHTEDVGVVCSEKRIPGFKFDNSLLNQIENMNIQVEDIRIRPILATYRKRVPVTEGYVEVKDEGTWKQICDKHWTMKNSRVVCGMFGFPSERKYNTKVYKMFASRRKQHYWAYSMDCTGNEAHISSCKLGNHLNVDTEKNATCDNGMPAVASCVPGRAFAPSSHSGFRKAFRQEQPLVRLKGGANTGEGRVEVLKNGEWGTVCDDNWNLVSASVVCRELGFGSAKEAITGARLGQGMGPIHLNEIDCTGFEKSLTDCKFNMESQGCNHEEDAAVRCNVPAMGFQNQLRLVGGRNPYEGRVEVLAERNGTLRWGTVCSQGWSTVEAMVVCRQLGLGFASHAFQETWYWHGDVSADSVVMSGVKCSGTEMSLAHCRHDGADVSCPRGGGRFGAGVSCSETAPDLVLNAELVEQTAYLEDRPMFMLQCAQEENCLASSAVNTSVTSGYRRLLRFSSQIHNNGQSDFRPKNGRHAWVWHDCHRHYHSMEVFTHYDLLNLNGTKVAEGHKASFCLEDTECEADVQKQYECANFGEQGITVGCWDVYRHDIDCQWIDITDVPPGDYLFQVVINPNYEVAESDYSNNVMKCRSRYDGQRIWMYNCHTGGSFSDETEQKFDHFSGLTNNKVSTR

>Hsap_ENSP00000373783

MERPLCSHLCSCLAMLALLSPLSLAQYDSWPHYPEYFQQPAPEYHQPQAPANVAKIQLRLAGQKRKHSEGRVEVYYDGQWGTVCDDDFSIHAAHVVCRELGYVEAKSWTASSSYGKGEGPIWLDNLHCTGNEATLAACTSNGWGVTDCKHTEDVGVVCSDKRIPGFKFDNSLINQIENLNIQVEDIRIRAILSTYRKRTPVMEGYVEVKEGKTWKQICDKHWTAKNSRVVCGMFGFPGERTYNTKVYKMFASRRKQRYWPFSMDCTGTEAHISSCKLGPQVSLDPMKNVTCENGLPAVVSCVPGQVFSPDGPSRFRKAYKPEQPLVRLRGGAYIGEGRVEVLKNGEWGTVCDDKWDLVSASVVCRELGFGSAKEAVTGSRLGQGIGPIHLNEIQCTGNEKSIIDCKFNAESQGCNHEEDAGVRCNTPAMGLQKKLRLNGGRNPYEGRVEVLVERNGSLVWGMVCGQNWGIVEAMVVCRQLGLGFASNAFQETWYWHGDVNSNKVVMSGVKCSGTELSLAHCRHDGEDVACPQGGVQYGAGVACSETAPDLVLNAEMVQQTTYLEDRPMFMLQCAMEENCLSASAAQTDPTTGYRRLLRFSSQIHNNGQSDFRPKNGRHAWIWHDCHRHYHSMEVFTHYDLLNLNGTKVAEGHKASFCLEDTECEGDIQKNYECANFGDQGITMGCWDMYRHDIDCQWVDITDVPPGDYLFQVVINPNFEVAESDYSNNIMKCRSRYDGHRIWMYNCHIGGSFSEETEKKFEHFSGLLNNQLSPQ

>Mmus_ENSMUSP00000022660

MELHFGSCLSGCLALLVLLPSLSLAQYEGWPYQLQYPEYFQQPAPEHHQRQVPSDVVKIQVRLAGQKRKHNEGRVEVYYEGQWGTVCDDDFSIHAAHVVCRQVGYVEAKSWAASSSYGPGEGPIWLDNIYCTGKESTLASCSSNGWGVTDCKHTEDVGVVCSEKRIPGFKFDNSLINQIESLNIQVEDIRIRPILSAFRHRKPVTEGYVEVKEGKAWKQICNKHWTAKNSHVVCGMFGFPAEKTYNPKAYKTFASRRKLRYWKFSMNCTGTEAHISSCKLGPSVTRDPVKNATCENGQPAVVSCVPSQIFSPDGPSRFRKAYKPEQPLVRLRGGAQVGEGRVEVLKNGEWGTICDDKWDLVSASVVCRELGFGTAKEAITGSRLGQGIGPIHLNEVQCTGTEKSIIDCKFNTESQGCNHEEDAGVRCNIPIMGFQKKVRLNGGRNPYEGRVEVLTERNGSLVWGTVCGQNWGIVEAMVVCRQLGLGFASNAFQETWYWHGNIFANNVVMSGVKCSGTELSLAHCRHDEEVACPEGGVRFGAGVACSETAPDLVLNAEIVQQTAYLEDRPMSLLQCAMEENCLSASAVHTDPTRGHRRLLRFSSQIHNNGQSDFRPKNGRHAWIWHDCHRHYHSMEVFTYYDLLSLNGTKVAEGHKASFCLEDTECEGDIQKSYECANFGEQGITMGCWDMYRHDIDCQWIDITDVPPGDYLFQVVINPNYEVPESDFSNNIMKCRSRYDGYRIWMYNCHVGGAFSEETEQKFEHFSGLLNNQLSVQ

>Mmus_ENSMUSP00000097987

MELHFGSCLSGCLALLVLLPSLSLAQYEGWPYQLQYPEYFQQPAPEHHQRQVPSDVVKIQVRLAGQKRKHNEGRVEVYYEGQWGTVCDDDFSIHAAHVVCRQVGYVEAKSWAASSSYGPGEGPIWLDNIYCTGKESTLASCSSNGWGVTDCKHTEDVGVVCSEKRIPGFKFDNSLINQIESLNIQVEDIRIRPILSAFRHRKPVTEGYVEVKEGKAWKQICNKHWTAKNSHVVCGMFGFPAEKTYNPKAYKTFASRRKLRYWKFSMNCTGTEAHISSCKLGPSVTRDPVKNATCENGQPAVVSCVPSQIFSPDGPSRFRKAYKPEQPLVRLRGGAQVGEGRVEVLKNGEWGTICDDKWDLVSASVVCRELGFGTAKEAITGSRLGQGIGPIHLNEVQCTGTEKSIIDCKFNTESQGCNHEEDAGVRCNIPIMGFQKKVRLNGGRNPYEGRVEVLTERNGSLVWGTVCGQNWGIVEAMVVCRQLGLGFASNAFQETWYWHGNIFANNVVMSGVKCSGTELSLAHCRHDEEVACPEGGVRFGAGVACSETAPDLVLNAEIVQQTAYLEDRPMSLLQCAMEENCLSASAVHTDPTRGHRRLLRFSSQIHNNGQSDFRPKNGRHAWIWHDCHRHYHSMEVFTYYDLLSLNGTKVAEGHKASFCLEDTECEGDIQKSYECANFGEQGITMGCWDMYRHDIDCQWIDITDVPPGDYLFQVVINPNYEVPESDFSNNIMKCRSRYDGYRIWMYNCHVGGAFSEETEQKFEHFSGLLNNQLSVQ

>Lcha_ENSLACP00000013682

MQAPFSLCHLLALLCLHGTLNLLCLAQYEHWPYYSGYPESQPEYHQAQRPADVSQIQLRLSGEKRKHNEGRVEVYYNGEWGTVCDDDFTIHAAHVVCRELGYVEAISWFPSSKYGRGEGRIWLDNIHCTGKESTLAACTSNGWGVSDCKHTEDVGVICNNKRIPGFKFSNTLINNIENMNIQVEEIRIKAILSSYRKRIPVTEGYVEVKDGGKWKQICDTNWISQNSRVICGMFGFPGERKFNRRVYKLFARRRKYNYWQYSINCTGNESHLSNCRLGPPVVDKNSSSTCENGIPVVVSCVPGRAFAPSSMVGFRKAFRQEQPLVRLRGAASVGAGRVEVLKNGEWGTICDDRWNVISASVVCRELGFGTALEAITGARFGQGMGPIHMNEVECTGFEKSITDCKFNNESLDCGHGEDAAVKCNIPQMGFEKQLRLSGGRNPREGRVEVLVETNGTLKWGTVCSDGWGTMEAMVVCRQLGLGFASHAFQETWYWQGDVNADNILMSGVKCSGTEMSLTHCRHDGNNLNCPRGGGQYSAGVSCTETAPDLVLNAEVVEQTTYLEDRPMFMLQCAYEENCLASTAAKSPFNTGSRRLLRFSSQIHNNGQSDFRPKMGRHAWIWHDCHRHYHSMEIFTHYDLMSLNGTKVAEGHKASFCLEDTACDSEDDKQYECANFGEQGITAGCWDVYRHDIDCQWIDITDVPPGDYLFQVVINPNYEVAESDYTNNVMKCQCRYDGYRIWMYSCHTGGSFSAETEGRFDHFSGLLTNQLSAR

>Drer_ENSDARP00000117086

VRLAGGRDPAEGRVEVLMEDGGRKHWGTICSENWGINEAMVVCRQLGFGFAARAHQETYYWQGDPAAEEVVLSGAHCVGTEMSIQQCRRNSYVHCPRGGGAKAAGVTCSETAPDLVLDAQLVQESSYLEDRPLHLLTCAHEENCLSSSASRMQWPYGHRRLLRFSSRIMNLGRADFRPRASRESWTWHQCHRHYHSIEVFTHYDLLTLNGSRVAEGHKASFCLEDTYCPEGLHKRFSCYNYGDQGISVGCWDTYRHDIDCQWIDITDVRPGDYIMQVEVNPSLDMAESDFMNNVMRCRCKYDGHRVFMYGCHAGDAYSAEVEDLFEHQRQISNNFI

>Drer_ENSDARP00000044962

IFSFGQLIKVFLIIVCMRRSRRAPAAKVRLAGIGHQENEGRVEVLHNGTWGTVCDDEVDIKLANVVCRELGFQSGITWAHSARYGEGEGPIWMDNVRCEGTEKALRDCRSNGWGVHDCKHSEDLGVVCSPERRLDQTYPGVSRRGHTIALRPNPVTSNRWQDIYSNPRTPAHLRQGNGHSQDQTRWDQLSRQQLPNPVNRLFQQDAFTVLFLHSMLNSRFFPLQKQLMTKCWSASRVRIEEVRLKPVLMVTKRRALITEGVVEVKHAGRWRQVCDKGWSLNSSRVVCGMLGFPDAEQPNMNTYKKIWDKKVKDSTTRLSQMAKKKGFWVEKLHCLGTEPSLAECHTQLSIPRSPAPCKNGRYAVARCVPGPQFARMSSGRPQAPHPVQLSVRLKAGPRLGEGRVEVLREGKWGTVVDHLWDRISASVVCRELGFGTAKEALTGAYMGQGTGPIHMNSVRCAGTERSILDCFFQDVQPWTFKHNRDASVKCYVPKTGVENTVRLAGGRDPAEGRVEVLMEDGGRKHWGTICSENWGINEAMVVCRQLGFGFAARAHQETYYWQGDPAAEEVVLSGAHCVGTEMSIQQCRRNSYVHCPRGGGAKAAGVTCSETAPDLVLDAQLVQESSYLEDRPLHLLTCAHEENCLSSSASRMQWPYGHRRLLRFSSRIMNLGRADFRPRASRESWTWHQCHRHYHSIEVFTHYDLLTLNGSRVAEGHKASFCLEDTYCPEGLHKRFSCYNYGDQGISVGCWDTYRHDIDCQWIDITDVRPGDYIMQVEVNPSLDMAESDFMNNVMRCRCKYDGHRVFMYGCHAGDAYSAEVEDLFEHQRQISNNFI

>Drer_ENSDARP00000105909

MGDQGITVGCWDTYRHDIDCQWVDITDVRPGNYIFQVEVNPSLDMAESDFQNNVMRCRCRYDGGRVYLYSCHTGDAYSAAAEDLFEHRQEITNNFL

>Xtro_ENSXETP00000034503

FCYNNTALHLLLLLLLYPPNCRTPQIQLRLAGSSRVPNEGRLEVFYNGIWGTVCDDDFSIEVAHVVCKQLGFEMAMNWAHSAKYGQGEGTIWLDNVRCSGTETSITECESNGWGISDCRHTEDVGVLCSTSRIRGSEQAQAIAVQMAINNQQRRQNPLGTNNSGPQPQSGPFDLNSAPSSVQGWFFQVGINGLFHTSFKPSVLSVQNMHVTVLKREISSLLANTKINASLSPEAEQIQQRVSLSHTQAHQLPCTASELGAISTGYLHKDAFRFRKASSASGVHVTAPVLLPFVRTSYTPGKYTKKIEEVRIKPILARAKASVPVTEGIVEVKHKGRWRQVCDHGWTAHNNRVICGMLGFPGKQLNTQNCYYRKLWNLKLKVPKSRLKTLSKRKMFWIHKVSCTGNEPHLSHCKVQVSPPNLKPACKNGMHAIVKCVAPPSVSNGHGNNYQKAFRTMEPLVRLRSGPHPGEGRVEVLKNGKWGTVCDDKWNLISASVVCRELGYGSAKEAITGGQLGQGLGPIHMNEVLCTGQEKSITECKFKEARLAGCQHEEDAAVRCNMPDMGYSKQVRLSGGRSNEEGLVEVLMDVKGVKKWGTICGEQWGMNEAIVACRQLGLGFASHALQQETWYWRGTPGAGEVVMSGVRCSGTEMSVQQCRHHSVVHCPRGGGRYSAGVTCTEHAPDLILNAQLVQESAYLEDRPLSMLECAHEEECLSSSADDMAWPYGHRRLLRFTSQIHNNGRADFTPRNGRHTWVWHECHRHYHSIEVFTHYDLLTMNGTKVAEGHKASFCLEDSNCGKGIQRRYECANFGEQGITVGCWDSYRHDIDCQWVDITDVLPGDYIFQVILNPKFEVAESDFRNNVMRCRCKYDGHRIWMHNCHTGDAFSADLEESFEHQLRLSNNLV

>Lcha_ENSLACP00000011190

TMKFAPSQLHWLLVFLLPFFAVSQTVPRQTGSQTSSSRIQLRLTGVGSRPNEGRVEVLHEGKWGTICDDDFSIHAAGVVCRELGFGGAVAWSHSAKYGQGDGPIWLDNVQCAGTESSVAECASNGWGVSDCKHTEDVGVLCSEQRLPNFNSIRSPTNLVVEGGIRIEDVRIKPLLARTKVKIPVTEGVVEVKHDGKWKQICDVNWTMNNTRVVCGMLGFPGEVPHNRKIYSKICTIHKKNPATRLSLFANKRSFWVHKIRCLGNEPHLSSCQAQLSAAKKKPHCKNGMHAVVSCVSGPEFSEDGTDGNRKVFRDEESLVRLKAGAQRGEGRVEVLKAGKWGTVCDYDWNMVSASVVCRELGYGTAREAVVGSLLGQGVGPIHMSEVQCSGFENSITECHFQDAGQSLCSHFQDAAVRCNIPQMGYHQKIRLAGGRTPNEGRVEVLMEVNGVEKWGAVCSDNWGLNEAMVVCRQLRFGFANHAIKETWYWAGNPVANQFVLSGSRCSGTELSIQQCPHHSVVHCPRGGKRFAAGVSCTENSPDLVLNAGLVQETAYLEDRPLDMLYCAHEENCLSKSANDMRWPEGHRRLLRFSTLIHNAGRADFRPRNGRHTWIWHQCHRHYHSIEVFTHYDLLTLNGSKVAEGHKASFCLEDTNCPAAGLHRRYGCANFGEQGITMGCYDTYRHDIDCQWIDITDVPPGEYVFQVIVNPDQEMAESDFTNNAMRCRCKYDGHRIWMFGCHTADAYSTEIEELFEHQRRLLNNQI

>Mmus_ENSMUSP00000026190

MMWPQPPTFSLFLLLLLSQAPSSRPQSSGTKKLRLVGPTDRPEEGRLEVLHQGQWGTVCDDDFALQEATVACRQLGFESALTWAHSAKYGQGEGPIWLDNVRCLGTEKTLDQCGSNGWGVSDCRHSEDVGVVCHPRRQHGYHSEKVSNALGPQGRRLEEVRLKPILASAKRHSPVTEGAVEVRYDGHWRQVCDQGWTMNNSRVVCGMLGFPSQTSVNSHYYRKVWNLKMKDPKSRLNSLTKKNSFWIHRVDCLGTEPHLAKCQVQVAPGRGKLRPACPGGMHAVVSCVAGPHFRRQKPKPTRKESHAEELKVRLRSGAQVGEGRVEVLMNRQWGTVCDHRWNLISASVVCRQLGFGSAREALFGAQLGQGLGPIHLSEVRCRGYERTLGDCLALEGSQNGCQHANDAAVRCNIPDMGFQNKVRLAGGRNSEEGVVEVQVEVNGVPRWGTVCSDHWGLTEAMVTCRQLGLGFANFALKDTWYWQGTPEAKEVVMSGVRCSGTEMALQQCQRHGPVHCSHGPGRFSAGVACMNSAPDLVMNAQLVQETAYLEDRPLSMLYCAHEENCLSKSADHMDWPYGYRRLLRFSSQIYNLGRADFRPKAGRHSWIWHQCHRHYHSIEVFTHYDLLTLNGSKVAEGHKASFCLEDTNCPSGVQRRYACANFGEQGVAVGCWDTYRHDIDCQWVDITDVGPGDYIFQVVVNPTNDVAESDFSNNMIRCRCKYDGQRVWLHNCHTGDSYRANAELSLEQEQRLRNNLI

>Mmus_ENSMUSP00000126686

MMWPQPPTFSLFLLLLLSQAPSSRPQSSGTKKLRLVGPTDRPEEGRLEVLHQGQWGTVCDDDFALQEATVACRQLGFESALTWAHSAKYGQGEGPIWLDNVRCLGTEKTLDQCGSNGWGVSDCRHSEDVGVVCHPRRQHGYHSEKVSNALGPQGRRLEEVRLKPILASAKRHSPVTEGAVEVRYDGHWRQVCDQGWTMNNSRVVCGMLGFPSQTSVNSHYYRKVWNLKMKDPKSRLNSLTKKNSFWIHRVDCLGTEPHLAKCQVQVAPGRGKLRPACPGGMHAVVSCVAGPHFRRQKPKPTRKESHAEELKVRLRSGAQVGEGRVEVLMNRQWGTVCDHRWNLISASVVCRQLGFGSAREALFGAQLGQGLGPIHLSEVRCRGYERTLGDCLALEGSQNGCQHANDAAVRCNIPDMGFQNKVRLAGGRNSEEGVVEVQVEVNGVPRWGTVCSDHWGLTEAMVTCRQLGLGFANFALKDTWYWQGTPEAKEVVMSGVRCSGTEMALQQCQRHGPVHCSHGPGRFSAGVACMNSAPDLVMNAQLVQETAYLEDRPLSMLYCAHEENCLSKSADHMDWPYGYRRLLRFSSQIYNLGRADFRPKAGRHSWIWHQCHRHYHSIEVFTHYDLLTLNGSKVAEGHKASFCLEDTNCPSGVQRRYACANFGEQGVAVGCWDTYRHDIDCQWVDITDVGPGDYIFQVVVNPTNDVAESDFSNNMIRCRCKYDGQRVWLHNCHTGDSYRANAELSLEQEQRLRNNLI

>Mmus_ENSMUSP00000125803

MMWPQPPTFSLFLLLLLSQAPSSRPQSSGTKKLRLVGPTDRPEEGRLEVLHQGQWGTVCDDDFALQEATVACRQLGFESALTWAHSAKYGQGEGPIWLDNVRCLGTEKTLDQCGSNGWGVSDCRHSEDVGVVCHPRRQHGYHSEKVSNALGPQGRRLEEVRLKPILASAKRHSPVTEGAVEVRYDGHWRQVCDQGWTMNNSRVVCGMLGFPSQTSVNSHYYRKVWNLKMKDPKSRLNSLTKKNSFWIHRVDCLGTEPHLAKCQVQVAPGRGKLRPACPGGMHAVVSCVAGPHFRRQKPKPTRKESHAEQELKVRLRSGAQVGEGRVEVLMNRQWGTVCDHRWNLISASVVCRQLGFGSAREALFGAQLGQGLGPIHLSEVRCRGYERTLGDCLALEGSQNGCQHANDAAVRCNIPDMGFQNKVRLAGGRNSEEGVVEVQVEVNGVPRWGTVCSDHWGLTEAMVTCRQLGLGFANFALKDTWYWQGTPEAKEVVMSGVRCSGTEMALQQCQRHGPVHCSHGPGRFSAGVACMNSAPDLVMNAQLVQETAYLEDRPLSMLYCAHEENCLSKSADHMDWPYGYRRLLRFSSQIYNLGRADFRPKAGRHSWIWHQCHRHYHSIEVFTHYDLLTLNGSKVAEGHKASFCLEDTNCPSGVQRRYACANFGEQGVAVGCWDTYRHDIDCQWVDITDVGPGDYIFQVVVNPTNDVAESDFSNNMIRCRCKYDGQRVWLHNCHTGDSYRANAELSLEQEQRLRNNLI

>Hsap_ENSP00000260702

MAWSPPATLFLFLLLLGQPPPSRPQSLGTTKLRLVGPESKPEEGRLEVLHQGQWGTVCDDNFAIQEATVACRQLGFEAALTWAHSAKYGQGEGPIWLDNVRCVGTESSLDQCGSNGWGVSDCSHSEDVGVICHPRRHRGYLSETVSNALGPQGRRLEEVRLKPILASAKQHSPVTEGAVEVKYEGHWRQVCDQGWTMNNSRVVCGMLGFPSEVPVDSHYYRKVWDLKMRDPKSRLKSLTNKNSFWIHQVTCLGTEPHMANCQVQVAPARGKLRPACPGGMHAVVSCVAGPHFRPPKTKPQRKGSWAEEPRVRLRSGAQVGEGRVEVLMNRQWGTVCDHRWNLISASVVCRQLGFGSAREALFGARLGQGLGPIHLSEVRCRGYERTLSDCPALEGSQNGCQHENDAAVRCNVPNMGFQNQVRLAGGRIPEEGLLEVQVEVNGVPRWGSVCSENWGLTEAMVACRQLGLGFAIHAYKETWFWSGTPRAQEVVMSGVRCSGTELALQQCQRHGPVHCSHGGGRFLAGVSCMDSAPDLVMNAQLVQETAYLEDRPLSQLYCAHEENCLSKSADHMDWPYGYRRLLRFSTQIYNLGRTDFRPKTGRDSWVWHQCHRHYHSIEVFTHYDLLTLNGSKVAEGHKASFCLEDTNCPTGLQRRYACANFGEQGVTVGCWDTYRHDIDCQWVDITDVGPGNYIFQVIVNPHYEVAESDFSNNMLQCRCKYDGHRVWLHNCHTGNSYPANAELSLEQEQRLRNNLI

>Ggal_ENSGALP00000021337

AMVMPAGLKPILLLLVLVLCQAAPSTQEPQEVQLRLAGPRGPAGEGRLEVLYQGRWGTVCDDGFDFHVATVACRQLGYTAAITWTHSATYGQGEGPIWLDNVQCGGNEVSLADCAHNGWGTSDCHHGEDAGVVCSGQRLRGDPPLTSAPAALGEGPGLSLEEVRIKPILAWAKLSMPVVEGAVEVKYNGRWRQVCDAGWTQNNSRVVCGTLGFPREKRVNTSFYRKLWNMKLKDPSSSLRNLSQKSSFWVHRVQCQGTEPHLSRCSTRLAPPAPSQNACPRGMHAIVSCVPGPAFQQNKNRDKKPPRKASVGKDLPVRLRAGAHAGEGRVEVLRHGQWGTVCGRRWDLAAASVVCRQLGYGTARQALLGAQMGQGLGPIHMSEVRCIGHERSLGECRFQDGEQSGCRHDDDAAVRCHVPHMDFQSQVRLAGGRSPEEGVVEVLVPVQGRLQWGAVCGAQWGLNEAMVVCRQLGLGFASHALQETWYWAGSPDASQVLMSGVRCTGTELALQQCQRHGPVHCPTGGGRFSAGVTCTTHAPDLVMNAQLVQETAYLEDRPLGLLYCAHEERCLSRSADTMQWPYGHRRLLRFSSQIHNLGRADFRPRMGRHAWTWHQCHRHFHSIEVFTHYDLLTLNGSKVAEGHKASFCLEDTNCPEGLQRRFACANFGEQGVSVGCWDTYRHDIDCQWIDITDVPPGSYTFQVVVNPKHEVAESDFSNNVLRCQCKYDGQRVWMHGCHTSDAYGADVVSEMERRERLANNLV

>Acar_ENSACAP00000003246

MLLLAPHHFALLSFLVCLGAPSWAQRPHIQLRLVGPRSQVGEGRLEVLYDGQWGTVCDDDFNIYVAGVACRELGYEGATNWAHSAKYGPGDGPIWLDNMRCSGTESSLAECESNGWGVSDCNHNEDIGVVCTGLRRPNPASSHFQGSKVEEVRIKPILARAKLSSPITEGAVEVKLQGRWRQVCDAGWTRNNTRVVCGMLGFPREGRVTTGFYRKLWNQKLKDPRSSLKSLSRNNTFWIHRIKCQGTEAHLGQCKLQVQPPSRNKSSCPRGMHALISCIAGPEFQPNKAKLPAKSKSKEKSQVRLRGGAHVGEGRVEVVMNGQWGTVCDDGWDLPAASVVCRQLGYGHSSRASYMNRSVGSSLGPIHLTRVRCRGYERSLAECSSQEARQTGCRHEADAAVRCHVPQTGVHKQVRLAGGRSSEEGVVEVLMSRGGTLRWGAVCGEHWGLEEAMVVCRQLGLGFASHALQESWYWQGNPGATEVLLSGTRCKGTEHAIQQCQHHGPVHCPTGGGRFAAGVTCTNSAPDLVMNAQLVQETAYLEDRPLNMLYCAHEEGCLSASANHMNWPDGYRRLLRFSSQIHNLGRADFLPKTGRHAWIWHQCHSHYHSIEVFTHYDLLTLNGSKVAEGHKASFCLEDTNCPDGLQRRFACANFGEQGVSAGCWDTYRHDIDCQWVDITDVPPGSYTFQVIVNPKHEVAESDFSNNALRCQCQYDGHRLWLHGCHTDVGDEYGANIAWDEEAQQRLASNLV

>Xtro_ENSXETP00000016976

LEMLVTHIFLLTLSLSVPTLGQYEHWLYYPEYQASQAPEPLPTPARNVPQIHVRLAGEKRKHNEGRVEVYYEGEWGTVCDDDFSMYAAHIVCRELGYQDAVSWSPSSKYGKGEGRIWLDNVNCNGREKSIASCGSNGWGVTDCKHSEDVGVQCSDRRIPGFKVSNELPGQLEGLNIQVEEVRIRAIVMPGHRRRSLGMAGYYSIKTQACWEMIEWEWINKRIFVYMNYMYPGHLRSTGLNVKFVWLFSSRRKHTYWQFSANCTGNEAHLSSCKVGGVLTPDPKTNQTCSDGSPAVVSCTPGRAFAPSPGTGFGKAFRQEQPLVRLRGGANTGEGRVEVLKNGEWGTICDDKWNLVTASVVCRELGFGSAKEALAGAQMGQGMGHIHMSEIQCNGFEKSLIDCKFNVHSQGCNHEEDAAVRCNVPAMGFENQVRLSGGRHPTEGRVEVLMERNGTLRWGTVCSDTWGTMEAMIVCRQLGLGFASHAFQETWYWQGDINADDVVMSGVKCSGTEMSLAHCRHDGANINCPRGGGRFAAGVSCVETAPDLVLNAALVEQTTYLEDRPMFMLQCAHEEQCLSSSADRTSPTTGYRRLLRFSSQIHNNGQADFRPKTGRHSWIWHDCHRHYHSMEVFTHYVKTENLSNTFSKSSVIVFFFADVQKQYACANFGEQGITVGCWDVYRHDIDCQWVDITDVAPGDYFFQVIINPNQEVAESDYTNNIMKCRCRYDGHRIWMYNCHIGGSYSTETEEKFEHFSGLMNNQLSTR

>Cmil_SINCAMP00000014829

QQPGGQTQRQPKIQLRLMGRRKKESEGRLEVYYSGEWGTVCDDEFSIAGANIACREMGFMDAVTWAPGAKYGQGEGRVWLDNVRCLGIESSIAHCPSNGWGVTDCRHSEDVGLVCSESRIPGFRFTNTLPNHIEDGLVQVDDVRVRGVFGSSRKRVPVKEGYVEVKEGGVWKQICNTGWTFNSSRVICGMFGFPAEKHFNAKVYKMFARRRHYTYWPYTVHCTGSEAHLTGCAIGLSAVDTNNTCESRMPVVVSCVAGQTFASSSSDGFQQAYRTEQPLVRLKGGGKIGEGRVEVLKNGEWGTVCDDQWTLVSASVICRELGFGSAKEALTGARLGKGIGSVHMNEVLRGFVGKSITDCSFNQESLCNHKEDAAVRCNVPTMGFENQIRLAGGRTPLEGRVEVLAKRNGTLRWGTVCNEGWSTVEALVVCRQLGLGFADHAIQETWFWEGDETADQVVMSGLKCSGTELSLFHCKHHGEELNCPNDGGRFAAGISCTETASDLVLNPQVVEETTYLEIRPLIMLQCAFEEECLASTANDMPRMTGFRKLLRFSSQIHNNGQADFRPKLGRHSWIWHECHRRCSDIPDATVHNVIIRPISFPLWKHQCKNSLKSNYECANFGEQGITVSCWDTYRHDIDCQWIDISDVPPGEYILQV

>Cmil_SINCAMP00000014830

KIQLRLMGRRKKESEGRLEVYYSGEWGTVCDDEFSIAGANIACREMGFMDAVTWAPGAKYGQGEGRVWLDNVRCLGIESSIAHCPSNGWGVTDCRHSEDVGLVCSESRIPGFRFTNTLPNHIEDGLVQVDDVRVRGVFGSSRKRVPVKEGYVEVKEGGVWKQICNTGWTFNSSRVICGMFGFPAEKHFNAKVYKMFARRRHYTYWPYTVHCTGSEAHLTGCAIGLSAVDTNNTCESRMPVVVSCVAGQTFASSSSDGFQQAYRTEQPLVRLKGGGKIGEGRVEVLKNGEWGTVCDDQWTLVSASVICRELGFGSAKEALTGARLGKGIGSVHMNEVVSTGSITDCSFNQESLCNHKEDAAVRCNVPTMGFENQIRLAGGRTPLEGRVEVLAKRNGTLRWGTVCNEGWSTVEALVVCRQLGLGFADHAIQETWFWEGDETADQVVMSGLKCSGTELSLFHCKHHGEELNCPNDGGRFAAGISCTETASDLVLNPQVVEETTYLEIRPLIMLQCAFEEECLASTANDMPRMTGFRKLLRFSSQIHNNGQADFRPKLGRHSWIWHECHRFVNDRISWSKRNSLGTVYIQGLFQLTSNSARGQPEKNYECANFGEQGITVSCWDTYRHDIDCQWIDISDVPPGEYILQV

>Drer_ENSDARP00000100065

MHAVVRCVPGAQFDTDSASRRPISAAVRLKAGARVGEGRVEVLREGKWGTVCDRRWDLSAASVVCRELGFGSARDAPRGALMGQGTGPIHMDEVQCSGQESSITECRFQDVPLYSCRHTQDASVRCNVPNTGLSSTVRLAGGRESAEGRVEVLMEVGGQQRWGSVCSENWNINEAMVVCRQLGFGFASRAHQETWFWPSSSGSEEVLLSGTHCVGSELSVQQCRRNTQVYCPRGGGARAAGVTCVDVAPDLVVDAQLVQESAYVEDRPLHLLTCAHEENCLSSSAARIDWRNRHTLAYTHRRLLRFSSRIMNLGRADFRPRATRESWTWHQCHR

>Ggal_ENSGALP00000040869

MRCAPPGLLLAQLHACIFWSGLWPAGCQSPPAAWRQRIQWENNGQVYSLLSQGAQYQPPRRRQAAEPASSPVLLLRGNGSVPRAAAAAARPQPEPQPQAQPQPRPRSQPGGSRGSGARHWFQAGYRAPSGSRAPAPRRRPRRRSRRRERAERRRAAPSGLRPGREDVMVGDDPYPYKYTDDNPYYNYYDTYERPRQGSRYRPGYGTGYFQYGLPDLVPDPYYIQASTYVQRMSMYNLRCAAEENCLASSAYRADVRDYDNRVLLRFPQRVKNQGTSDFLPSRPRYSWEWHSCHQHYHSMDEFSHYDLLDASSHRKVAEGHKASFCLEDTSCDYGYYRRYACTAHTQGLSPGCYDTYNADIDCQWIDITDVKPGNYILKVSVNPSYLVPESDYSNNIVRCDIRYTGHHAYASGCTISPY

>Lcha_ENSLACP00000004698

KMKLDFGMFGYLLVHLCFYSSCLIQVINCQHHRPPGTWRQRIQWANNGQVYSLLSQGSQYQPPRRREQGSTSNPQNPVILLSNNRTSEARAAGARPTSRQGATPSTSSRQSQSGARRSGSGYWFQAGNNNRNQGENRSSTSLGSEGARQSAQDTPVPGARRENQTRGNHDGMVGDDPYNPYKYTDDNPYYNYYDTYERPRQNGRQRPGYGTRYFQHGLPDLVPDAYYIQVSTYVQRASMYNLRCAAEENCLASSAYRSDVRDYDTRMLLRFPQRVKNQGTADFLPSRPRYSWEWHSCHQHFHSMDEFSHYDLLDAYSQRSVAEGHKASFCLEDTTCDYGYYRRYACTAHTQGLSPGCYDTYNADIDCQWIDITDVKPGNYILKVSVNPSYLVPESDYSNNVVRCDIRYTGHYVYTSNCRISHY

>Cmil_SINCAMP00000008426

QMADNHTVSSLTLGRSSAYSQDVRDYDMRVLLRFPQRVKNQGTADFLPSRPRYSWEWHSCHRHYHSMDEFSHYDLLDPTTQRQIAEGHKASFCLEDTSCEYGYYRRYACTAHSQGLSPGCYDTYNADIDCQWIDITDVKPGNYILKVSVNPSYLVPESDYANNVVRCDIRYTGQHVYTSGCRIS

>Xtro_ENSXETP00000014096

MGCALGSVFLLQLAVYLHCILLGNCQHQRNPLGPSSMWRQRLQWQNNGRVYSLLSHGSEYQPARRREQEGSAPQSPVLLLSSRHNSSVSQSAAERPQPTRSVTPSSEGQQQPASTNQATSRFWFQATRQRSQGDAQRQRGSGAAQERRQGAQPSHRVNGTGRVEGMQGDDPYNPYKYSEDNPYYNYYDSYERPRTSERQRPGYGTRYFQNGLPDLVPDPYYIQASIYVQKMSMYNLRCAAEENCLASSAYRSDIRDYDQRVLLRFPQRVKNQGTADFLPSRPRYTWEWHSCHQHYHSMDEFSHYDLLDASSQRRVAEGHKASFCLEDTSCDYGYYRRFACTAHTQGLSPGCYDTYNADIDCQWIDITDVKPGNYILKVSVNPSYLVPESDYSNNIVRCDLRYTGHYVYTSSCTISPY

>Xtro_ENSXETP00000010172

LRNNMGCALGSVFLLQLAVYLHCILLGNCQHQRNPLGPSSMWRQRLQWQNNGRVYSLLSHGSEYQPARRREQEGSAPQSPVLLLSSRHNSSVSQSAAERPQPTRSVTPSSEGQQQPASTNQATSRFWFQATRQRSQGDAQRQRGSGAAQERRQGAQPSHRVNGTGRVEGMQGDDPYNPYKYSEDNPYYNYYDSYERPRTSERQRPGYGTRYFQNGLPDLVPDPYYIQASIYVQKMSMYNLRCAAEENCLASSAYRSDIRDYDQRVLLRFPQRVKNQGTADFLPSRPRYTWEWHSCHQHYHSMDEFSHYDLLDASSQRRVAEGHKASFCLEDTSCDYGYYRRFACTAHTQGLSPGCYDTYNADIDCQWIDITDVKPGNYILKVSVNPSYLVPESDYSNNIVRCDLRYTGHYVYTSSCTISPY

>Acar_ENSACAP00000003666

MRALPLFPAWVCWLGLFWLQAEGLPPPRNPAPPPPPPAVWQQRIQWENNGQVFSLLSVGSQYQPPRRRHSSEAPQGNHVLLLRGNGTAPRRAASPAAAPSLAPAERHWFQAGGGGGGGGGGRGRGSEAASPPPPESAPSRSGNNSSSHTPPLNSLPRRRDDVMQGDDPYNPYKYTDDNPYYNYHDTYERPRQGSRYRPGYGTGYFQYGLPDLVPDPYYIQASTYVQRTSMYNLRCAAEENCLASSAYRGDVRDYDNRVLLRFPQRVKNQGTSDFLPSRPRYSWEWHSCHQHYHSMDEFSHYDLLEASSHRRVAEGHKASFCLEDTSCDYGYYRRYACTAHTQGLSPGCYDTYNADIDCQWIDITDVKPGNYILKVSVNPSYLVPESDYSNNIVRCDIRYTGHHAYASGCSISPY

>Mmus_ENSMUSP00000025409

MRFAWAVLLLGPLQLCPLLRCAPQTPREPPAAPGAWRQTIQWENNGQVFSLLSLGAQYQPQRRRDPSATARRPDGDAASQPRTPILLLRDNRTASTRARTPSPSGVAAGRPRPAARHWFQAGFSPSGARDGASRRAANRTASPQPPQLSNLRPPSHIDRMVGDDPYNPYKYSDDNPYYNYYDTYERPRPGSRNRPGYGTGYFQYGLPDLVPDPYYIQASTYVQKMSMYNLRCAAEENCLASSAYRADVRDYDHRVLLRFPQRVKNQGTSDFLPSRPRYSWEWHSCHQHYHSMDEFSHYDLLDANTQRRVAEGHKASFCLEDTSCDYGYHRRFACTAHTQGLSPGCYDTYAADIDCQWIDITDVQPGNYILKVSVNPSYLVPESDYTNNVVRCDIRYTGHHAYASGCTISPY

>Mmus_ENSMUSP00000129247

MRFAWAVLLLGPLQLCPLLRCAPQTPREPPAAPGAWRQTIQWENNGQVFSLLSLGAQYQPQRRRDPSATARRPDGDAASQPRTPILLLRDNRTASTRARTPSPSGVAAGRPRPAARHWFQAGFSPSGARDGASRRAANRTASPQPPQLSNLRPPSHIDRMVGDDPYNPYKYSDDNPYYNYYDTYERPRPGSRNRPGYGTGYFQYGLPDLVPDPYYIQASTYVQKMSMYNLRCAAEENCLASSAYRADVRDYDHRVLLRFPQRVKNQGTSDFLPSRPRYSWEWHSCHQHYHSMDEFSHYDLLDANTQRRVAEGHKASFCLEDTSCDYGYHRRFACTAHTQGLSPGCYDTYAADIDCQWIDITDVQPGNYILKVSVNPSYLVPESDYTNNVVRCDIRYTGHHAYASGCTISPY

>Hsap_ENSP00000231004

MRFAWTVLLLGPLQLCALVHCAPPAAGQQQPPREPPAAPGAWRQQIQWENNGQVFSLLSLGSQYQPQRRRDPGAAVPGAANASAQQPRTPILLIRDNRTAAARTRTAGSSGVTAGRPRPTARHWFQAGYSTSRAREAGASRAENQTAPGEVPALSNLRPPSRVDGMVGDDPYNPYKYSDDNPYYNYYDTYERPRPGGRYRPGYGTGYFQYGLPDLVADPYYIQASTYVQKMSMYNLRCAAEENCLASTAYRADVRDYDHRVLLRFPQRVKNQGTSDFLPSRPRYSWEWHSCHQHYHSMDEFSHYDLLDANTQRRVAEGHKASFCLEDTSCDYGYHRRFACTAHTQGLSPGCYDTYGADIDCQWIDITDVKPGNYILKVSVNPSYLVPESDYTNNVVRCDIRYTGHHAYASGCTISPY

>Drer_ENSDARP00000014541

MSMSLIDTFIYAFAHVCLLSCIAQTGQTQRQGNTGAAALRQTIQWQHNGKLFSILSQGSEYQPPLKRDGNKEQAQARPVAIVRNDDAATRTDSSAPSRASQSRGSVRVPSGTGARGGASRWLSGDGARTRGVHGRRNHTDPLRSINGTDRPAGDDEVMVGDDPYNPYKSTDPDNPYYNYYDTYERPRPAQRPGYGTGYFQNGLPDLVGDPYYIQASTYVQRVPMYNLRCAAEENCLASSAYRSSVRDYDMRMLLRFPQRVKNQGTSDFLPSRPRYTWEWHSCHQHYHSMDEFSHYDLLDATTHRRVAEGHKASFCLEDTSCDYGYYRRYACTSHTQGLSPGCYDTYNADIDCQWIDITDVKPGNYILKVSVNPSYQVPESDYSNNVVRCDVRYTGNYAYVSGCHISQY

>Drer_ENSDARP00000110708

MSMSLIDTFIYAFAHVCLLSCIAQTGQTQRQGNTGAAALRQTIQWQHNGKLFSILSQGSEYQPPLKRDGNKEQAQARPVAIVRNDDAATRTDSSAPSRASQSRGSVRVPSGTGARGGASRWLSGDGARTRGVHGRRNHTDPLRSINGTDRPAGDDEVMVGDDPYNPYKSTDPDNPYYNYYDTYERPRPAQRPGYGTGYFQNGLPDLVGDPYYIQASTYVQRVPMYNLRCAAEENCLASSAYRSSVRDYDMRMLLRFPQRVKNQGTSDFLPSRPRYTWEWHSCHQHYHSMDEFSHYDLLDATTHRRVAEGHKASFCLEDTSCDYGYYRRYACTSHTQGLSPGCYDTYNADIDCQWIDITDVKPGNYILKVSVNPSYQVPESDYSNNVVRCDVRYTGNYAYVSGCHISQY

>Drer_ENSDARP00000102256

MSMSLIDTFIYAFAHVCLLSCIAQTGQTQRQGNTGAAALRQTIQWQHNGKLFSILSQGSEYQPPLKRDGNKEQAQARPVAIVRNDDAATRTDSSAPSRASQSRGSVRVPSGTGARGGASRWLSGDGARTRGVHGRRNHTDPLRSINGTDRPAGDDEVMVGDDPYNPYKSTDPDNPYYNYYDTYERPRPAQRPGYGTGYFQNGLPDLVGDPYYIQASTYVQRVPMYNLRCAAEENCLASSAYRSSVRDYDMRMLLRFPQRVKNQGTSDFLPSRPRYTWEWHSCHQHYHSMDEFSHYDLLDATTHRRVAEGHKASFCLEDTSCDYGYYRRYACTSHTQGLSPGCYDTYNADIDCQWIDITDVKPGNYILKVSVNPSYQVPESDYSNNVVRCDVRYTGNYAYVSGCHISQ

>Drer_ENSDARP00000104349

MNEFCHYDLLDSRTQQRVAEGHKASFCLEDTSCDPGYHRRYACTSHTQGLSPGCYDTYSADIDCQWIDITDVQPGRYILKVTVNPGFQVSESDFSNNIVRCDVHYTGNYAHVSGCTVSPH

>Cmil_SINCAMP00000014707

FPFAYRMGPLLALLCYLLSTFGLPGARAQQAQTQAQAQAQARSRVWRQRIQWESNGQVYSFLNSGGAYRPRRRAGGSGAGRRGSGSGAGRAARPSGTRVVSPRLAEHPGWGLQEARGISGSRGVNFTHGIPSPAGGGVSFAPVGTSQQPDSGGTEQRGVSPPAPLLTHVRSTRQMLADDPRNPLKNRNSVFYNAYSSSRSSRPVSRTRQGYGTRYFQNGLPDLIPDPYYIQASTYIQRLHMYALRCAAEENCLSRSAYRSGVSDLSIRVLLRFPQRVKNQGTADFLPNKPRHTWEWHSCHQHFHSMDEFSHYDLLEATTQRKVAEGHKASFCLEDTMCDPGFSRRYACTAHTQGLGPGCYDTYNADIDCQWIDITDVRPGNYVLKVSVNPRFLVPESDVSNNVVRCDIVYTGNYVSARNCRIT

>Ggal_ENSGALP00000042527

GGGAIVWGGGIGRYEDYGEEQSPYRAQGFYPERPYAAPQDGLDRRYSHSLYHDAGAVPEHGAVISNDNLQPPVGSQPASGTAYGNQYQPYEAQPPFRALEPYGAVRPDVFVPARSPEVPQAVPDGQARLSVGSVYRPSHGGRGLPDLVPDPNYVQASTYVQRAHLYSLRCAAEEKCLASTAYTPEATDYDVRVLLRFPQRVKNQGTADFLPSRPRHSWEWHSCHQHYHSMDEFSHYDLLDATTGRKVAEGHKASFCLEDTTCDFGNLKRYACTSHTQGLSPGCYDTYNADIDCQWIDITDVQPGNYVLKVQVNPKYIVLESDFTNNVVRCNIHYTGRYVATTNCKISHF

>Acar_ENSACAP00000016905

MTVHGFFWDLWAFASFGMFLMVRGQHAQSDSNTDNGRWRQMIQWENNGRLYSLLNSGSEYVPASQVRADSSARVMLADAATHQSRRVPGNMRRQAPTLPVRAGSDTVRGQTRHPFGFGQVPDNWRNGATAEGNSNSARPRSPTTWQRQVGSSASSFSHSSFGQVYPQASYPQPPYANQYETYDPQVPRAYDESYTYYRTAGTGGSAVAAAAASAGVIYPFQPQVRYEEYTEDQNPYRTQGYYPAAERPYVPVPQQLPSSDGLDRRYSHSLYHDGGTSYDQNVQDPYSSTQNQGSPVVADNLHLNSASPAGTGTQYGNEPWARQYPPFEDAPVEPYVPSRHVEPQPPFRPIDPQAVPRHEPYLPIRNSEPPQLIPDNQGVNQGRMSVGSIYRPNQNGRGLPDLVPDPNYVQASTYVQRAHLYSLRCAAEEKCLASTAYTPEATDYDIRVLLRFPQRVKNQGTADFLPNRARHTWEWHSCHQHYHSMDEFSHYDLLDAATGKKVAEGHKASFCLEDTTCDFGNLKRYACTSHTQGLSPGCYDTYNADIDCQWIDITDVQPGNYILKVQVNPKYIVMESDFTNNVVRCNVHYTGRYVATTNCKISQQGGAPESLKSMPEG

>Cmil_SINCAMP00000014100

MGTVRLLILSVTWLMSLPWELVEAQGHPEDNNGRWRQMIQWENNGRVYSLLNTGSEYIPSGRYRPRNPRVWLGGMGRTTASNRLQPERRQAPSLPARGVSETVRGQTRHPFGFGQVPDNWRQGTLGVPSSTHRYLPSQRQEPRQRQRLPYAEPPFPQSPSSPQSPSFPQSPYQRYLLPQPSYPNSYDSNYEYGSPRRFDPVDDRSPYRRVPESFSPDAGAYPYRPRPRYDDYGEDGYRYRPQSRLPFQEHSSFSPVQPSDGLDRRYMHSLYDESRGQDQGQAADTGADSSPFLPSGGDVNPESVGAGRYPDAGPRAVPEGTGHYPSSRRAPADHHQLPRQAEPNVPFVNPEPYPVTRGDPYERVRGEEQQGRLGQASRVALGSVYRQDRTGRGLPDLTPDPYYVQASTYVQRSHLYSLRCAAEEKCLASSAYTEESTDYDIRVLLRFPQRVKNQGAADFLPNRPRHTWEWHSCHQHYHSMDEFSHYDLLDAATGRKVAEGHKASFCLEDTTCDFGHLKRYACTSHTQGLSPGCYDTYNADIDCQWIDITDVQPGTYILKLQVNPNYLVQESDFTNNVIRCNIHYTGRYVSTKNCKITQS

>Hsap_ENSP00000261921

MALARGSRQLGALVWGACLCVLVHGQQAQPGQGSDPARWRQLIQWENNGQVYSLLNSGSEYVPAGPQRSESSSRVLLAGAPQAQQRRSHGSPRRRQAPSLPLPGRVGSDTVRGQARHPFGFGQVPDNWREVAVGDSTGMARARTSVSQQRHGGSASSVSASAFASTYRQQPSYPQQFPYPQAPFVSQYENYDPASRTYDQGFVYYRPAGGGVGAGAAAVASAGVIYPYQPRARYEEYGGGEELPEYPPQGFYPAPERPYVPPPPPPPDGLDRRYSHSLYSEGTPGFEQAYPDPGPEAAQAHGGDPRLGWYPPYANPPPEAYGPPRALEPPYLPVRSSDTPPPGGERNGAQQGRLSVGSVYRPNQNGRGLPDLVPDPNYVQASTYVQRAHLYSLRCAAEEKCLASTAYAPEATDYDVRVLLRFPQRVKNQGTADFLPNRPRHTWEWHSCHQHYHSMDEFSHYDLLDAATGKKVAEGHKASFCLEDSTCDFGNLKRYACTSHTQGLSPGCYDTYNADIDCQWIDITDVQPGNYILKVHVNPKYIVLESDFTNNVVRCNIHYTGRYVSATNCKIVQS

>Mmus_ENSMUSP00000057406

MALAGAGSQLRTLVWSACLCVLVHGQQAQPGQGSDPGRWRQLIQWENNGQVYSLLNSGSEYVPAGPQRGETSSRVLLAGAPQTSQRRSQGGPRRRQAPSLPLPGRVGSDTVRGQTRHPFGFGQVPDNWREVAVGDSTGMARARTSVSQQRHGGSASSSVSASAFATTYRQPSSYPQQFPYPQAPFVNQYENYDPASRTYEQGYVYYRGAGGGMGAGAAAVASAGVIYPFQPRARYEDYGGGGGEEQPEYPAQGFYPAPERPYVPQPQPQPQPQPQPQPQPSDGLDRRYSHSLYNEGTPGFEQAYPDPSTDVSQAPAGAGGTYGGAGDPRLGWYPPYAANVPPEAYVPPRAVEPQPPFRVLEPPYLPVRSSDAPSQGGERNGAQQGRLSVGSVYRPNQNGRGLPDLVPDPNYVQASTYVQRAHLYSLRCAAEEKCLASTAYAPEATDYDLRVLLRFPQRVKNQGTADFLPNRPRHTWEWHSCHQHYHSMDEFSHYDLLDASTGKKVAEGHKASFCLEDSTCDFGNLKRYACTSHTQGLSPGCYDTYNADIDCQWIDITDVQPGNYILKVHVNPKYIVLESDFTNNVVRCNIHYTGRYVSTTNCKIVQS

>Lcha_ENSLACP00000013586

GQGGDAGNNPWRQLIQWENNGRVYSLLNTGTEYVPAGQDRQGGNPRVVVADGTRSQSRRGQGNSRRQAPSQPARGSSETVRGETRHPFGFGQVPDNWREGPVGDPSSAQRFRPSTFGRVRQSGSASSFSQPSLGQPVYPPVYPYAQQPNLSPYDPYDYQLPRVYGDGQPYYRGTGTAGGRGYPYQPRPGFEDLGEDMYPYRSQGYYPSPERPYVPGPQQPSVSDGLDRRYTHSLYHENQGGTEQLRQDGPPISQGNGGSVPAQDTMWVAPVVPGGTGSRTVPDTGYGFYPPYRNVPPEPYVPPRNADPYPPFRSLDPYPSPRLDPFSPVRTLDPAQASPNERPASQPRVNVGSVYRTDGNGRGLPDLVPDPNYVQASTYVQRAHLYSLRCAAEEKCLASSAYDSESTDYDIRVLLRFPQRVKNQGTADFLPNRPRHTWEWHSCHQHYHSMDEFSHYNLLDASTGRKVAEGHKASFCLEDTTCDFGHLKRYACTSHTQGLSPGCYDTYNADIDCQWIDITDVQPGNYILKLQVNPKYAVLESDFTNNVIRCNIHYTGRFVATTNCRISQS

>Xtro_ENSXETP00000011277

LVPRVTANGVLNMAFLGQLCLWVWGLSVALGQEGDGWGQMIQWEESGRRYRLLNSGSEYQAAGGGTGGSRVLLDGSRSGGSDLRRRQAPSSPRTSSQTIRGNTRHPFGFGQVPDNWRSVSESASTGRIVPSAGAGSSGRIRQSSSQASVGTQFFPRAQPPFVPQSDLNPQGYDEGYGYQRAGGGGGGYFAQSWAGGYEDFWEEPSPPFAQPPYFGIPPNLPPQQAPQANPVLPQDGLDRRFAHSLFRGEDPPVPASDPVRPGYGGGGGAGYPPFGGVREGDGGYYVATRPEPVQPPARVPSVPATGGQAQVEQQGRATVGSVFRGNQNGRGLPDLVPDPSYVQAATYIQRAHLYSLKCAAEEHCLSSSAYSAEATDYDVRVLLRFPQRVKNQGTADFLPTRPRQSWEWHSCHQHYHSMDEFSHYDLLDATTGRKVAEGHKASFCLEDTTCDFGNLKRYACTSHTQGLSPGCYDTYNADIDCQWIDITEVKPGNYILKVVVNPKYKVLESDFTNNVVRCNIHYTGRYASATNCRITQF

>Drer_ENSDARP00000109770

MLHVLLMSLWVLGSVTGQSQSQPDDTNPWRQMIQWENNGRVYSLLNSGAEYVPARNQERDRNHRVLLADAPNRRSQGGNVRRQAPSRGSSETVRGQARHPFGFGQVPENWRQQQGAVGRSETSRFQSQTGSRYRPSSGASSSASSSYPQYPIPQQPPFGAPYDQVSDRSYEPPFLGTGYSAGTGGGLGGGGYGGYSTGSFGGGNPANDDRYRFYPPYGQQYQAVPAQPAQPPFSDGLDHRYTHSLFNEDNPAVPNGASSNTGSSFQPAVQSPQYEQFPPYGRPQPQPPFLQPAPRNPLVSNTAENPNINVGSVYRPQQRGLPDLVPDPNYVQASTYVQRAHMYSLRCAAEEKCLASSAYNAETTDYSVRVLLRFPQRVKNQGTADFMPNRPRHTWEWHSCHQHYHSMDEFSHYDLLEVSSGRKVAEGHKASFCLEDTTCDFGHLKRYACTAHTQGLSPGCFDTYNADIDCQWIDITDVQPGNYILKLQVNPKYLVLESDFTNNIVRCNIHYTGRYAKTTNCKISQS

>Drer_ENSDARP00000018477

XLLNMLHVLLMSLWVLGSVTGQSQSQPDDTNPWRQMIQWENNGRVYSLLNSGAEYVPARNQERDRNHRVLLADAPNRRSQGGNVRRQAPSRGSSETVRGQARHPFGFGQVPENWRQQQGAVGRSETSRFQSQTGSRYRPSSGASSSASSSYPQYPIPQQPPFGAPYDQDRSYEPPFLGTGYSAGTGGGLGGGGYGGYSTGSFGGGNPANDDRYRFYPPYGQQYQAVPAQPAQPPFSDGLDHRYTHSLFNEDNPAVPNGASSNTGSSFQPAVQSPQYEQFPPYGRPQPQPPFLQPAPRNPLVSNTAENPNINVGSVYRPQQRGLPDLVPDPNYVQASTYVQRAHMYSLRCAAEEKCLASSAYNAETTDYSVRVLLRFPQRVKNQGTADFMPNRPRHTWEWHSCHQHYHSMDEFSHYDLLEVSSGRKVAEGHKASFCLEDTTCDFGHLKRYACTAHTQGLSPGCFDTYNADIDCQWIDITDVQPGNYILKLQVNPKYLVLESDFTNNIVRCNIHYTGRYAKTTNCKISQ

>Drer_ENSDARP00000123413

MFFTISNFIFFLYLLQGLNNLTSAQSSAQWRNRVRWVNNGQVFSLMSTGSEFHAPVPSRRQSRVYQSSRTDAVPGRSMQIRLEAMDRPANSAPDSALLGPDRAQYIMANSRAPGARQMQVMQRHRAPPASRNNSTVPSEYSGGGGRTGENTRRGQAVANFQQIAAPTDNSNTVNSDNGNEARTPNVPAEQGATSETMPGDDPRNRNTVFYNIYPPGGRTIIPRRPPPGTGYGTRFFQNGLPDLVPDPYSIQAGSYIQRVQMYALRCAAEENCLARTAYRPTVRDLDYRVLLRFPQKVRNMGTADFLPVKPRHQWEWHSCHQHYHSMDAFSHYDLLDISTGRKVAEGHKASFCLEDTGCDPGFHRRYACTAHTQGLSPGCHDTYAANIDCQWIDITDVPPGNYFLKVTVNPDFLVAESDFSNNVVRCEVIYTGIYIQTRNCIITGS

>Drer_ENSDARP00000102938

MFFTISNFIFFLYLLQGLNNLTSAQSSAQWRNRVRWVNNGQVFSLMSTGSEFHAPVPSRRQSRVYQSSRTDAVPGRSMQIRLEAMDRPANSAPDSALLGPDRAQYIMANSRAPGARQMQVMQRHRAPPASRNNSTVPSEYSGGGGRTGENTRRGQAVANFQQIAAPTDNSNTVNSDNGNEARTPNVPAEQGATSETMPGDDPRNRNTVFYNIYPPGGRTIIPRRPPPGTGYGTRFFQNGLPDLVPDPYSIQAGSYIQRVQMYALRCAAEENCLARTAYRPTVRDLDYRVLLRFPQKVRNMGTADFLPVKPRHQWEWHSCHQHYHSMDAFSHYDLLDISTGRKVAEGHKASFCLEDTGCDPGFHRRYACTAHTQGLSPGCHDTYAANIDCQWIDITDVPPGNYFLKVTVNPDFLVAESDFSNNVVRCEVIYTGIYIQTRNCIITG

>Drer_ENSDARP00000076348

MTKYSFIFCLSIHLCVLVFLNTAQNHGAWQHKVKWETNGQVYSLLSASSQYHAPASGKQHARFLLRRQIIPSFTGRIFMGQFAKTRMHTAGHVVRSSPVPGHIGLNAKHMISGHIRNNNQLPLHAKKVAFSSDETLRTQTWPYRTSPTGTTTHGPVKNSEISRAPKQHKITSQPVAKQPSPATGKPPTPEEKQHAKPQPISNATNKSKLSPSLDAPSASGNVRNVRNPLVQVEARGGESMIDDEPTNHQANRNSFYNLLPYGNTNRSPQRETGHGTRYFLNGLPDLIPDPYYIQAASYIQRVQMYTLRCAAEENCLSSSAYSSSVRDLDYRVLLRFPQRVKNQGTADFLPVKPHYDWEWHSCHQHYHSMDAFSNYDLLDAATGRKVAEGHKASFCLEDTSCDPGVRRRYACTAHTQQGLGPGCYDTYHANIDCQWIDITDVSLGDYILKVTVNPGFQVQESDFSNNVVRCDIRYTGLYVQTNNCRITG

>Drer_ENSDARP00000099758

MTKYSFIFCLSIHLCVLVFLNTAQNHGAWQHKVKWETNGQVYSLLSASSQYHAPASGKQHARFLLRRQIIPSFTGRIFMGQFAKTRMHTAGHVVRSSPVPGHIGLNAKHMISGHIRNNNQLPLHAKKVAFSSDETLRTQTWPYRTSPTGTTTHGPVKNSEISRAPKQHKITSQPVAKQPSPATGKPPTPEEKQHAKPQPISNATNKSKLSPSLDAPSASGNVRNVRNPLVQVEARGGESMIDDEPTNHQANRNSFYNLLPYGNTNRSPQRETGHGTRYFLNGLPDLIPDPYYIQAASYIQRVQMYTLRCAAEENCLSSSAYSSSVRDLDYRVLLRFPQRVKNQGTADFLPVKPHYDWEWHSCHQHYHSMDAFSNYDLLDAATGRKVAEGHKASFCLEDTSCDPGVRRRYACTAHTQQGLGPGCYDTYHANIDCQWIDITDVSLGDYILKVTVNPGFQVQESDFSNNVVRCDIRYTGLYVQTNNCRITG

>Drer_ENSDARP00000100806

MTKYSFIFCLSIHLCVLVFLNTAQNHGAWQHKVKWETNGQVYSLLSASSQYHAPASGKQHARFLLRRQIIPSFTGRIFMGQFAKTRMHTAGHVVRSSPVPGHIGLNAKHMISGHIRNNNQLPLHAKKVAFSSDETLRTQTWPYRTSPTGTTTHGPVKNSEISRAPKQHKITSQPVAKQPSPATGKPPTPEEKQHAKPQPISNATNKSKLSPSLDAPSASGNVRNVRNPLVQVEARGGESMIDDEPTNHQANRNSFYNLLPYGNTNRSPQRETGHGTRYFLNGLPDLIPDPYYIQAASYIQRVQMYTLRCAAEENCLSSSAYSSSVRDLDYRVLLRFPQRVKNQGTADFLPVKPHYDWEWHSCHQHYHSMDAFSNYDLLDAATGRKVAEGHKASFCLEDTSCDPGVRRRYACTAHTQGLGPGCYDTYHANIDCQWIDITDVSLGDYILKASNGKPRFSSAGVRLLQ

>Lcha_ENSLACP00000013256

PPNTLNINEAHRKRKTLIAPEINISLDQSEGSILSDDYLDTPDDLDINVDDIETPDETDSLEFLGNGNELEWEDDTPVRTTKNLPGESADPFGDGSAEEGGATNGRLWRTVIIGEQEHRIDMQIIKPYMKVVTHGGYYGEGLNAIIVFAACCLPDSSSSDYHYIMENLFLYVISSLELLVAEDYMIIYLNGATPRRRMPGLGWLKKCYQMIDRRLRKNLKSLIIVHPSWFIRTVLAISRPFISVKFINKIQYVHSLEELEQVIPMEHVQIPECVLQFDEERAKARRESIEKEYHPTGLERPKTCVTNGGKTTLTNRLLKTLPNCCVVHQDDFFKPQDQIEVGEDGFKQWDVITSLDMEAMLSTVKAWKENPVKFARSHGVNVSPASEEADSGKQIHILIVEGFLLYNYTPLIELYDQRYYLAIPYDECKRRRSTRRYTVPDPPGLFDGHVWPMYIKYRKEVEANNIDSLRLPDLIPDPYFIQASTYIQRMQMYSLRCAAEENCLARSAYRPGVSDISYRVLLRFPQRVKNQGTADFLPVKPRHAWEWHSCHQHYHSMDAFSNYDLLEVNTQRKVAEGHKASFCLEDTSCDPGFRRRYACTAHTQGLGPGCYDTYNANIDCQWIDITDVPPGNYILKVTVNPSFQVPESDFSNNVVRCDIRYTGTFVTTRNCRVTRF

>Bflo_89357

MEVFAHYLLLDVTGARAAEGHKASFCLEDTQCSFNYLRKYDCDLGSQGISAGCWDTYKHDIDCQWIDITDVKPGKYILEISINPERRVRESSFDNNRITCDVRLYRNSVQVYNCIRT

>Bflo_60295

MDAFSYYDLLDKNTGGQAAEGHKPSWCLENTGCGYTSRRPVSYNCHWGTQGISSGCWDTYTSDLDCQWIDVTDVTPGFYIMEVAVNHHRRVIESDYSNNRVRCDVILEKSTAYVYNCINT

>Bflo_89360

MDAFSYYDLLDKNTGGQAAEGHKPSWCLENTGCGYTSRRPVSYNCHWGTQGISSGCWDTYTSDLDCQWIDVTDVTPGFYIMEVAVNHHRRVIESDYSNNRVRCDVILEKSTAYVYNCINT

>Csav_ENSCSAVP00000017976

RKVAEGHKASFCLEDSSCTDRKIKVFNCDEGHQGISPGCEDTYKAAVDCQWIDVTDVPLDEYTFRVTVNPKRLVRESNFANNGIRCKVSIKRHTADIWNCSPIGV

>Cgig_10017884

MFGILCVCFGLIPLINAVPEFEDGDLRLLGGSTENEGTVLIYHNGRWGSICDRGWDIRDGNVACYQLGFQRALQTLRYSPFGPGRTFRWLTGLRCRGRESRLDQCHPRQWGIERDQRYCSRYSRSAAVVCLPHSTTTSTTTPSTTTTKTTTTTTAKQTTQSTKKSTVASVGLAVNNIIPTRRAIDAIEHNRDKEEEKEEETKTVVERYSNNTIIFDDKDRDDIDNDDQDYESDEEDAEDNNENAIQSTARSRSQPRRSHLQFDAEWSETRRTKDRDVITTASPLKESTSTSRPPTTKEHSRAAAPAPVGPNRYDEEVYSISLPSRRWTKDPLLKCWVLLTYREEILIAKPGQRPSRITKEEYYKKRRAEIIKQRGEETIPNEINRSPLNDASGSRTGNTGNNQIVDRGRVPTTTTKCHIMTTTTTTEAAAPAPVSAYENEVFSVELVTSKWIKDEELNMWVMLSRSGGVSVAKPGQRAVKIDEDEYYQMRREAIIKARDRLKADDPSPGQVPAPSTRQLSFGRGGGTIRSRNEDASSSSNIVISSDLNITREHARGNHHRNRHGHGSKKIETRLGGSRSNRGRLEVRLKGREEWGVVCGDHWTIKEAMVVCRDMNAGYGQQAIKRAVFGGINMKKYFSRVRCKGTEKRLEDCQWEDHEMSIQCTSSDSVAGVICASALPDLEPSIYMLETSSFLQDRHLYYLQCAMEEKCLAPSAYEAQRSRGWRAHTRRLLRFSSVVKNKGTADFRPMLNRDEWEWHACHMHYHSMDVFAHYDILDTNGNRVAEGLKASFCLEDSACDRGVRPKYSCQNYGQQGISVGCSDNYMADIDCQWVDITDLKQGKYVFKVEINPNLIVAEISYDNNVVVCDLNYTGYYARIYNCKHEGLL

>Acal_524881610

MEVFAHYDIMDAEGNRLAEGSKASFCLEDTVCDPGVVPQYNCRGYAEQGLSVNCSDNYMYDIDCQWIDITDIKPGDYTFLLEVNPSLLVAESDFGNNVVSCQLNYNGYFAFLKNCHYESLLEYRKPIKMG

>Lgig_193984

MLNLNCETMLNVCVALLVFLAATVDGLREGQIRLVGGKNDNDGTVVIYHNRKWGSICDDEWDMRDARVTCQQLGFPGAKKAARRSEFGRGRRRMWMSRVRCSGFESQLRYCMFFGWGRIRHRCRGGWRSAGVVCLPKKNPGTDGTPIYSTTPTPATTTEALHNSGVQIRIRGGRYFWEGRLEVKRNPEETAWGTVCGKVWSIRETMVACRELGLDFGKQALQVNYFGGQNMDKIYNEVKCTGRESRLDECFRVETADSVSCAGKHLVAGIVCSKFLPDLIPSLHRLEESILLQDRPLYYLQCALEENCLSSSATTIRNSSKNWMGASRRLLKFSTVVHNRGVADFRPYRAKGQWEWHQCHMHYHSMEVFAHYDIIDEHGNRLAEGSKASFCLEDSRCENGITPKYDCTGFGDQGLSVNCSDNYMNDIDCQWIDITDIPVGKYIFKMEVNPKLLVAELDYDNNVAVCDLTYTMYNAFLQNC

>Dpul_50513

MYLSILLAILAECEGQQQQEQLQRRQQRQPQQQQRQRPTREGTRRQRRHRTDKAKLIQHYLKKNGRLEGMVRIVDGLRENEGNVEIYHSGKWGSICDDEWDIREATVVCRELGYSDVVQETHNSMYGPARYPFWMDNLYCVGGENNLTDCRFDGWGISDCEESEAAGVICKPGPLSRLTAQQTSTTTSTTTTTASTMMQDIIGSKGKIRLRGGRSKEEGRVEVKIGNNANDWGLICGDGWSLFEAGVVCRELKLGYAQNALQTDFFGGNRSSLALSGVKCHGSEKSLMDCLHDRFGAVSCPGNANNIATVVCASEMADLVPDAQEMMRTAHLEDRQLFFLQCAMEENCLAASAYQVQKDDPYGWHLETRRLLRFTARIANMGTADFKPFIPKHMWEWHACHMHYHSMEVFAHFDIINSLGVKVAEGHKASFCLEDNQCTHNTEPVYKCANYGDQGISVNCTDTYHHNIDCQWIDITDIDPGIYTFKVVINPEFKVAEMSFDNNAVVCTLYYGQQYATLFNCSLQRP

>Isca_XP_002402428

MEENCLSTSAYALKSDPGWLYESRRLLRFTARVANVGSTTFKPFLPKHAWLWHSCHMHYHSMEVFAHFDVLDLEGRKVAEGHKASFCLEDNVCQPGIEKTYSCANYGDQGISIGCTDTYAHNIDCQWVDMSDVAHGQYIFKVSINPEYKMAEISYDNNAALCDLTYTQTFAAVTNCTLVRP

>Tcas_TC011653

MNVTILLMVLSSPSLLFASNFDNSKRKKAFLVKKHLKRLKKVEGGVKLIGGRDEFEGNVEILHEGQWGAICDDEWDSSEAHVVCQQLGYPKDSGKATVSSYFGPAKRKFWMDNVFCSGNESEIIDCRFDGWGNNDCTSTEAAGVICHEEKPKEETTKKVEAPLARHEIRLKGGRVKSEGRVEIKNEKGQWASICGDGWSLLEGLVVCKHLGLGYASDAPQTDFFGGNITDSSYSGVRCRGNETSFGQCVHDLTSRGKCQSRDVAAVSCVSQMSDLVIDHIDLMRTAHLEDRQLFFLQCAMEENCLASEAYKIQKEDGSWHLETRRLLRFTARIFNAGTADFRPTIPKHLWEWHMCHMHYHSMEVFATFDIYDTKGLRVAEGHKASFCLEDNQCLPGVKPRYACANYGDQGISVNCSDIYKYTVDCQWVDISELEPGIYTLKVAINPEFKVPEITFENNAAVCQFIYSETFGTVTNCTVQRP

>Dmel_FBpp0071659

MCAIEDRKVLKMGLTLVCLTLLAIHMADAVVQHRSLEDARQERQRLVHRYTKVLNKEEGAIRLVGGDNEYEGNIEVLHNGKWGAVCDDEWDSTEADIVCRQLGFPGMRRYTRSGFFGPARRRFWMDNLFCEGHEQELVDCHFEGWGENDCEPGEAAGVVCYPPENALIPMATPIIRDEDLPKYPIHSRSRLYVRLRGGRSRIEGRVEVSLDGGRWGSVCADGWSLLEANVVCRQLGLGYASEAFQTDFFGGFNVSRPVLSGSECYGNETELADCLHHDASQGIISCHGNRQHVAAVICDYIAPDLVVDYLEIEQTAHLEDRPMLLMQCAMEENCVANEAYQIQRDDPHWRYRSRRLLKFTAAAINAGNADFRPFKEKSQWEWHMCHMHFHSMEVFATFDIFNLRGIKVAQGHKASFCLEDSNCLPGVAKKYNCANYGDQGISINCSDVYLYNLDCQWVDVTDLIPGTYVLKIAINPEFKVAEMNYDNNAAICDLIYTANFARVQNCQLGRP

>Hrob_73641

MFLLTLSILALLGSSTYRKIVQADETASENLKELQIILEKSDRGTSESGNVLIYKDNKLWSICDDNWNINSAHVACRLLGFVGASNAHANSFFGATHYEIGLDNVVCTGLEDHLISCQHAIFGKHNCNKREAAGVTCSNNIPTSTKKPTRPTYNRESFTVPNDFSGNSVYKEPYAIRLQNGRNDKEGRVEVKVEGGEWGVVCGDGWSLLEAMVVCRQLGLNYSLSALKTSFFGGDNMAKIFNQIRCDGDEANLTECYHGQMLERVSCSKANRVAGVMCVERLPDLVPNATLIEKSAYLHDQSMYHLQCAMEENCAAPSAFEIKNTKSDWHVHRRRLLRFSSSTWNFGTADFKPILRKQDWEWHLCHMHYHSMQVFASYDVLDAYGNKKAEGHKASFCLEDVECVPNVTKKYACKGYGDQGISIGCADNYLHDIDCQWVDMTDVPPGYYIFKVHINPEFKIAELDFSNNAVTCVLYYSGSSVNLNSCKLGRG

>Ctel_142796

MIIAVGLIVLLASAHGDVPADGSLRLTGGAMETTGNVMVFYQGKWGIVCDDGWSLRAADVVCRSLGYLRALGHTDQAYFGTPNEDVFLDNVRCRGDESSLLECDRNPWHDHDCEKSEAAGVFCAPLPGSLSSPLRVNVSNSTPRITSPHEQHTPTQGPETNSIRMPIRLRGGRNEDEGRVEVRVQGHWGAICGDHWTLLEATVVCKQVGKGHARSAVMSSVFGGEHLAKVLSGIDCMGEEDSLDECSHARRSHDIACPEQDFIAGVVCTHELPDLVPNATLLESSTYLQDKQMYYLTCAMEENCASASAYEIKKTVRDWHIHQRRLMRFSSSTWNFGTADFRPESNKADWEWHLCHMHFHSMEVFAHYDLLDLQHNKVAEGHKASFCLEDVQCLPGKQKKFACKGYGDQGISVGCADDYLHDIDCQWIDITDLKPGTYIFRVHINPNNHVAELSYSNNAVHCDLSYTGLSVTVTNCENGPI

>Skow_XP_002742392

MELVCRPAPWLATIAMVVIWTLSSNTNGQSIGNDEDLKIQLRLMDGRSIYEGRVEIFVNDEMGTICDDYFTLTTANVICKDLGFAGAESFYYGGHFNPGTGRIWLDSLRCDGNESSIKECSHAPWGVTDCSHDEDVGVRCKSSMIANTDFEDSESESNQLTSDNEDSVGRREHGPMRVDLNVGSLKVRLRGRRTAYPKSEGYVEVYFRNRWRPVCADGWDMADSRVVCGQMGFSEAIPIKLLEFKDKMRTRMQNAWLTNVTCRGVESTLQECSYHTLSNAEQCISGMPATARCERGHFVEDSRGKKTDNVLSEYLGDDAILQKPIVRLKSGPYLGSGRVEVFYDGKWGTVCGDSWNMASANVICRELGFGTAKEISLSSDTYGQGTRNVWLDKVNCTGDENSIFDCPHSTWNYAGESLNGFCTHANDAGVVCHVPGQRGLLKIRLVGGRTEMEGRLEVQHGTTWGSVCSDEWDMRSAMVACRQLGLGFAHQPLRDVTYFGGLDVPIIMSGVSCRGDELSLNQCKHDGWHTPKCDQYHIAGVVCSTSLPDLVPDLGLIESTAYIDERPFGLLQCAMEENCVSSSAYDVIPGTMPFIFGTRRLLRFSSSIANRGTSDFIPVTDKRYWEWHQCHQHHHSMEVFATYDLLNSEGYKVAEGHKASFCLEDTHCDIGVSKHYSCLNYGDQGISVNCIDEYKHTIDCQWIDISDIEPGFYILRVHVNPNVFVAESDYSNNDVLCNLAYNGYEISVNNCRFASEE

>Adig_11514v108253

MRWILLASIALLSVELSCSVKVRLVGGKHKYEGRIEILYKSEWRAVCDHKWNKNGARIVCRMLGYPDVLRFTKGPHAFGRGNGKFWLDDVVCSGEEESIASCSHRPWGRNNCLSFNQAGVVCKRHMSDMVAVPKPSIASGEVRKLNIRLQGPVVDDYISEGVVQVQHEGIWGYICPSTWTKANSFVLCGDLGFPNMEIQGSNSAASQEEQPVYWLNKVTCQGWESSIVSCDHAGFARHQCDDDGVLRIKCVRRHITKPLDVRLRSGVLVSEGRVEVQYQDFWGTVCDDHWTLKEANVVCRSLGYGSAAMAATNAYFGRGMGRVLLDDVNCHGTERHITQCRHRGWRRSNCNNHEDASVSCHAPALQGHQIRIQGSTNPKEGRVEVFHDGKWGTVCGDTWGIEEAMTVCRQLNLGYAGRALTENNFTATEHRVIMSGVRCRVDEVSLYNCQHDEWTNTTCSSQKSVAGVVCVNELPDLVINTDMLKTYMEVNTIALQYLQCAMEENCLSASSAWMMRDSVWNRRRLLRFSVQVENRGLDHFRPAVDKSKWQWHKCHKHYHSMETFSSYDLLSQRTGKKVAQGHKASFCLEDTKCDPGFERVWNCTDMGDQGISPGCFDIYHYNIDCQWVDVSDLTHGAFYLRVQVNPGNQVAESDFRNNVAKCQVYDYGRYVIVGSCRIENCDSGVDTHGGNAGGDCCVFPFKFRGKLFHDCTTDSYSKKWCSTTFDFKKDRKWGLCHD

>Nvec_XP_001629781

KHYHSMEVFSTYDLIDDRGQKVAEGHKASFCLEDTGCDAGIHRHWNCTDGGDQGVKPNCYDEYKWTIDCQWIDVTDRPHGNYRLQIKVNPNQMVAETDYDNNIAMCDAYDYGSFLLMQNCYLGE

>Adig_11521v108251

MLHDVHCQGNETNISHCKNKGWKGSGCNHYEDASVRCHAPQLQGHKIRLSGGANAYEGRVEVFRSGAWGTVCADDWRIESAMVVCRQLKLGYAAHAVTQNYFGHTNLRVIMSGVQCHVDEISIFNCQRDHWENVTCSRSNKLAGVICSKALPDLVIDTAELQKTIIQEYRTLYDLRCAHEESCLSKSADALFKYGSLSRDYRKLLRFTTKIENRGWEDFRPDSPRGSWDYHRCHAHFHSMETFATYDLLGKRYSAIQAEGHKASFCLEDTECDPGFDKRWNCTRGGDQGISPGCYDVYKSTIDCQWVDFTDVRQRGSYILRIRLNPGNQVAETDFRNNIAKCSVIYYGRFVLPSHCWIEDCQSGVDTYGGNSRGNCCVFPFNYNGKVYEACTTDGFAKKWCATTKNYSKDKKWGLCFD

>Bflo_126230

MEAAVRVRVLWALLVLLVPAVVGEELETVEIRLRGGSDDNEGRVEVLYEGEYGTICDDEWNINAANVVCRQLGYKGAVDFKRSSFFGPGTGQVWLDNVVCKGNESKIGDCSHRGWGKNDCNHDEDAGVICSTEVRDDINISDRYTTSGLFSTDTRVTRLVPPHKSSGKLNSQPKVDLPPSRTGPYDARSCCVHTGVLDARYERPLLHKDAWLTRVDCTGSESTLTECRLERLIQGTVSLPCLSNMTAVVKCDLXPRYSGVSPTTHRKLSAITTQPKFVRLKGGANTGEGRVEIYYNRQWGTVCGQSWDLKQASVVCRELGYGSARSAPKGAQFGQGHGPVWLDHVVCTGNELSIVDCAHSNFSATSCTHDHDASVQCNVPDLKVKEKIRLSGGRNPREGRVEIQRGRKWGIVCSNSWTYKEAGVACRQLGMGYALHALKEVWYFEGDKLDFVMADLECDGTEEALHFCRYKSWTNKGCKSHQVAGIICTQARLPDLVPDASQVRQSIYLEDRPLYLLYCAAEENCLASSAYQIRLSGGRNPREGRVEIQRGRKWGIVCSNSWTYKEAGVACRQLGMGYALHALKEVWYFEGDKLDFVMADLECDGTEEALHFCRYKSWTNKGCKSHQVAGIICTQARLPDLVPDASQVRQSIYLEDRPLYLLYCAAEENCLASSAYQMEWPYGSRRLLRFTQAVANFGKADFRPNIDSSQWIWHQCHAHYHSMEIFTQYDLLSENGTKVAEGHKASFCLEDSHCYDGYEPKYDCNVGEQGISMGCDDTYKHSLDCQWIDITGVPSGTYTLEVIINPLRAVAEMDFENNRISCKLNYNGYQVVANECHLETCYRENFILHLTAPEGGEPLVIGDRWARTESVVGQIPDCYYTQLQDPASRPVIPGESQRLSTTMQIQCAQVFRVCGIASFVVFCVLYGSYQYYYNYYGYYGYGDSQSSNDIDSEPGLPDLVPDAYVIQVSAYLDTQPMSTLQCALSENCLARSAWNADLSDERKLLRFALKTENRGKGEFNPELPRRYWVWHSCHQ

>Bflo_125512

MAMERHLRVLFLFLVALVPSTLAWLVTLRVGDIRLRGLTKRIPQAEGYVEINYQEKWWPICADGWDYNDAKVVCGQLGFRDALTVDLRKYRYERPLLHKDAWLTRVDCTGSESTLTECRLERLIQGTVSLPCLSNMTAVVKCDLGPRYSGVSPTTHRKLSAITTQPKFVRLKGGANTGEGRVEIYYNRQWGTVCGQSWDLKQASVVCRELGYGSARSAPKGAQFGQGHGPVWLDHVVCTGNELSIVDCAHSNFSATSCTHDHDASVQCNVPDLKVKEKIRLSGGRNPREGRVEIQRGRKWGIVCSNSWTYKEAGVACRQLGMGYALHALKEVWYFEGDKLDFVMADLECDGTEEALHFCRYKSWTNKGCKSHQVAGIICTQARLPDLVPDASQVRQSIYLEDRPLYLLYCAAEENCLASSAYQMEWPYGSRRLLRFTQAVANFGKADFRPNIDSSQWIWHQCHAYEPKYDCNVGEQGISMGCDDTYKHSLDCQWIDITGVPSGTYTLEVIINPLRAVAEMDFENNRISCKLNYNGYQVVANECHLGEDELLNSIY

>Hrob_183983

MTTLIIVVLVAVKVVESDDRWRNSSRWANDSLQQIVREHRLYKDMMNMEKMTPLKSSRVIHSPQPYEVRLQGGRTSSEGRVEILIGGMWGVICPDSWTNLEARVVCYQLGLGYSRNALKTGAFKGDKMVRLASGIECDGNEEKLSDCHHRHYGDDTECTVSSKWVAAVMCSFRLPDLVPNATMIQKSMYLQDQPMAYLQCAMEENCAAPEAFVLREKRSDWQSIKRRLLRFSAMTWNFGTAEFLPHGRKDDWEWHLCHKHYHSMSNFAEYDIIDQLGNRVAHGHKASFCLEDNYCIDGVKKFYACDGFGDQGVTIGCADIYAHDIDCQWVDISEMSVGNFTFKMKVNPEYHVGELDFDNNAVLCDLGYLGGSIKVSNCKLARG

>Csav_ENSCSAVP00000011337

EGRVEVFHNGEWGTICDDDWSLAAAHVVCRMIGYDGAWEYLHSGKFGPGEGNILMDNVDCIGDESDISRCQFNGWGNHDCSHHEDAGVKCNKRRLPGFHPDSQVVATETIRLKVGSAEGEGRLEVMRSGRWGTVCHRGWNLWAANVACRELGFGTAKREIINSYFGAGHGPIWLTDLNCFGNETQLSDCRHGYISTHAEGYDELEESECTHEHDAGVACHVPQFNANQRIRLVGGRNPMEGRVEVKIRRKWGAVCSDNWTIKDAMVVCRQLGLGFALHALKDVYFFPGTENVTNILMTGIQCRGDELALQFCPNDGNTLSQCGSPGRTFTPFAGVICTELSPDLMQDIPLLQQSLHLDDRPLHNLYCASEEGCLAPSAEKMDWPYGSRRLLRFSTRVWNRGRADFRPAKSQDQWIWHQCHGHYHSMSEFTHYDILDLNFTRVAQGHKASFCLEDSECSPGVSPRFDCDQPGGGVQGIAVGCADNYQYNIDCQWIDITDVSPGNYLIRIRVNPGSLVAESDFGNNEVICNLQYDGSRVWTWNCHI

>Csav_ENSCSAVP00000011336

IQVRLREGKEASEGRVEVFHNGEWGTICDDDWSLAAAHVVCRMIGYDGAWEYLHSGKFGPGEGNILMDNVDCIGDESDISRCQFNGWGNHDCSHHEDAGVKCNKRRLPGFHPDSQVNVIDGLDFLRLKPPRGQRKRLPQLHGYVEFYHKNKWRKVCSTGWDKNTASVICGQLGFPSAEEITNKMQYLSKARRKLHYWLSNITCIGTESKVSHCNFQMVDTHTYNSCPGEPLITRCVPGFKYARGNFKKGGKRKGRKHHATRQETIRLKVGSAEGEGRLEVMRSGRWGTVCHRGWNLWAANVACRELGFGTAKREIINSYFGAGHGPIWLTDLNCFGNETQLSDCRHGYISTHAEGYDELEESECTHEHDAGVACHVPQFNANQRIRLVGGRNPMEGRVEVKIRRKWGAVCSDNWTIKDAMVVCRQLGLGFALHALKDVYFFPGTENVTNILMTGIQCRGDELALQFCPNDGNTLSQCGSPGRTFTPFAGVICTELSPDLMQDIPLLQQSLHLDDRPLHNLYCASEEGCLAPSAEKMDWPYGSRRLLRFSTRVWNRGRADFRPAKSQDQWIWHQCHGHYHSMSEFTHYDILDLNFTRVAQGHKASFCLEDSECSPGVSPRFDCDQPGGGVQGIAVGCADNYQYNIDCQWIDITDVSPGNYLIRIRVNPGSLVAESDFGNNEVICNLQYDGSRVWTWNCHI

>Csav_ENSCSAVP00000011338

EGRVEVFHNGEWGTICDDDWSLAAAHVVCRMIGYDGAWEYLHSGKFGPGEGNILMDNVDCIGDESDISRCQFNGWGNHDCSHHEDAGVKCNKRRLPGFHPDSQVNVIDGLDFLRLKPPRGQRKRLPQLHGYVEFYHKNKWRKVCSTGWDKNTASVICGQLGFPMQQTNKSSQQIKFLMLMKSFDFFNNSRSARRKLHYWLSNITCIGTESKVSHCNFQMVDTHTYNSCPGEPLITRCVPGFKYARGNFKKGGKRKGRKHHATRQETIRLKVGSAEGEGRLEVMRSGRWGTVCHRGWNLWAANVACRELGFGTAKREIINSYFGAGHGPIWLTDLNCFGNETQLSDCRHGYISTHAEGYDELEESECTHEHDAGVACHVPQFNANQRIRLVGGRNPMEGRVEVKIRRKWGAVCSDNWTIKDAMVVCRQLGLGFALHALKDVYFFPGTENVTNILMTGIQCRGDELALQFCPNDGNTLSQCGSPGRTFTPFAGVICTELSPDLMQDIPLLQQSLHLDDRPLHNLYCASEEGCLAPSAEKMDWPYGSRRLLRFSTRVWNRGRADFRPAKSQDQWIWHQCHGHYHSMSEFTHYDILDLNFTRVAQGHKASFCLEDSECSPGVSPRFDCDQPGGGVQGIAVGCADNYQYNIDCQWIDITDVSPGNYLIRIRVNPGSLVAESDFGNNEVICNLQYDGSRVWTWNCH

>Csav_ENSCSAVP00000011339

SSTIQVRLREGKEASEGRVEVFHNGEWGTICDDDWSLAAAHVVCRMIGYDGAWEYLHSGKFGPGEGNILMDNVDCIGDESDISRCQFNGWGNHDCSHHEDAGVKCNKRRLPGFHPDSQVNVIDGLDFLRLKPPRGQRKRLPQLHGYVEFYHKNKWRKVCSTGWDKNTASVICGQLGFPSAEEITNKMQYFIFCLNLIFFLSMVYFHDVHLHRSKARRKLHYWLSNITCIGTESKVSHCNFQMVDTHTYNSCPGEPLITRCVPGFKYARGNFKKGGKRKGRKHHATVMQETIRLKVGSAEGEGRLEVMRSGRWGTVCHRGWNLWAANVACRELGFGTAKREIINSYFGAGHGPIWLTDLNCFGNETQLSDCRHGYISTHAEGYDELEESECTHEHDAGVACHVPQFNANQRIRLVGGRNPMEGRVEVKIRRKWGAVCSDNWTIKDAMVVCRQLGLGFALHALKDVYFFPGTENVTNILMTGIQCRGDELALQFCPNDGNTLSQCGSPGRTFTPFAGVICTESRKCRTCYLLFIISYHLMILEHYSNTFQYNNLNFPPVSPDLMQDIPLLQQSLHLDDRPLHNLYCASEEGCLAPSAEKMDWPYGSRRLLRFSTRVWNRGRADFRPAKSQDQWIWHQCHGHYHSMSEFTHYDILDLNFTRVAQGHKASFCLEDSECSPGVSPRFDCDQPGGGVQGIAVGCADNYQYNIDCQWIDITDVSPGNYLIRV

>Cint_ENSCINP00000013061

EGKTENEGRVEIYHGGEWGTICDDDWNIEAANVVCRMLGYDGAWEYLHSGKFGPGEGDILMDNVQCTGDESIISSCQFNGWKNHDCSHHEDAGVKCNKIRLPGFHRNLQVSLYLPANTGDEGLDFIRLKPPRGHRKRLPQLDGYVEFYHQKKWRKVCSTGWDLNTASVICGQLGFPRAEEITDRSQYLARARKKHYYWLSNITCIGTENKVSQCKFQNVDISAYNTCPGEPLITRCIPGPKYARGNFKKGGKRRKHRKHNVLRQETIRLKVGSAEGEGRLEVMRSGRWGTVCHRGWNLWAANVACRELGFGTAKREIINSYFGAGHGPIWLTDLNCRGNETQLSDCQHGYISTSAEGYEDSELTECNHEHDAGVACYVPQFNANQRIRLVGGRNPMEGRVEVKIRKRWGAVCSNDWTMKEAMVVCRQLGLGFALHSLKDVYFFPGTENITEIAMTGIHCKGDELALQFCPSDGRSLSVCGDPHRSSTPFAGVICTELSPDLMQDIPLLQQSLHLEDRPLHNLYCASEEGCLSPSAAKMDWPYGSRRLLRFSTRVWNRGRADFRPARSQDQWIWHQCHGHYHSMAEFTHYDILDLNFTKVAEGHKASFCLEDSECSPGVSPRFDCDQPGGGVQGIAVGCADNYQYNIDCQWIDISDITAGNYLIRIRVNPGTLVAESDFGNNEVICNLQYDGSRVWAWNCH

>Spur_NP_001073015

MQFSVGLFVQYFIFILITCGHGLPSNNETNTNNPTINTESTPIEPTTIAATTTTTSKPTTAKQEETTTQPPKTTQPTTVQPTTTQPTTTPTTFPPTTGEPTTRPPAKIRARLADGMHPWEGRVEVSMHGGSWGTVCDDGFNMNAANVVCKMVGYKKAVQYFFGSSQFGRGPGRILLDEVECLGTEKNLLACEHNPIGVTDCSHSEDVGIRCIDVVRKVTPPEKKLKQYKFRLVGDPTPKPQSEGVVEVMMTGKWRTICSDGWDMEDARVLCGSLGFKEATSVEPNRKNKSRQRKKSIVASNFECTGNEHNVSDCERTILKSHKDCLSKRAAEAKCERGPFVMDADKPYSPPWSDVRLKAGAAFGEGRLEVFHAGRWGTVCGEDFDKVAASVVCRKLGFGTAETVLENASAFGQGIGPIWLWEVDCKGTEASIMECGHGAYNRSKCDHSQDVGIKCNIPDLGVKEDIRLVQGRFDTEGRVLINYEGQWGHPCGGDSWDMFDAKVACRQLGLGYAHQPMRSTRFMGRSGYPILMLDVNCTGNERTLSECPHVRSDNGTCMADYKAGILCAGVLPDVLMDVHVLQSSIYMQDWPMYMLQCAAEENCLSSESHVRGARRLLRFASAIMNRGTEEFRPVLGRSSWEWHACHRHYHSMDVFSTYDLLDADGVRVAEGHKASFCLEDVYCDSGAQKVYSCDAGTQGISVNCVDIYRNDIDCQWIDVTGVPQGSYVLRVDVNPNHIVGETDFTNNEVLCDVMLTYRVRIQNCRYADDS

>Adig_191v118502

MTVCRQLNLGFASEAVINDRFHGRDPRVIMSGVTCRVDAISIYNCQHDPWTNTTCSSKGSSAGVVCVDELPDIVPDTEVLQRDMKINSIPLQYLRCPLEENCLSETADYEISDMTYYMRRLLRFSVKTDNVGLADFRPNVPRSKWKWHKCHKHYHSMETFSSYDLIKQDSGQKIAKGHKASFCLEDTNCYEGFDKKFNCSMDGGQGISPGCYDLYGWRIDCQWVDCTDFTHGAFYLRVHINPGNRRIVTVELTHMAAIQEEIVVSFHSFTMANYITTVPQMALSSDGAQQHSILEETVNGGFATTRARDEAKRQISQHIM

>Emul_000217900

MNAILSLLLLLSETTLVDVSAGAIKKPVDGDLKLLDGDNFASGTVAVYRGYAWGRVCDDNWSIREANVVCRQLGLGFAVRALKRNKFHSVSGRNYFMDNVRCLGNETRLIDCKFDGWARHDCAEHEDAGVQCAQDTSPRAKISWNPLRLDYTREELEKLAKVSFTLKNRGTSGVYQVKELNTTQAVDDAQILIIQPKDGEAGALCPDDFTTMDAIVACKQTNSGIGGRIAEIPLASDVFPAMKHVAIIGHCFGNETSLDQCKHYVDPNGVKCKSTKAVAVACQDKLPDLTSDIEQLENSVHIQRLRLWHLQCALEEHCFPDSVYTYIANNPGRYYWDARTLIRFSSITKNVGTAPFLPALIPEHWEWHPCHAHYHSMKVFGSYEVIDIMERLVSYGHKASFCLEDNHCDKNVTKHFFCSNVMDIKGEQGISPGCQDEYFFNYDCQWVDITDLPVGDYTYQVTYNPHYLVPESNYFNNAVTCKMQYRGNWGRFYGCKIVHPFELL

>Sman_CCD75815

MIPLFQAQCALEENCFPPSVYNLINRNRHLALMHMRRLLRFSSIIHNVGTDVFRPHEPPERWVWHACHMHYHSMKVFSYYKVINAKQQLMAVGHKASFCLEDNACKNGYKKHFVCSTTLVTRGDQGISPGCQDNYFHDYDCQWLDITDLIPGEYTFQLILNPDFLVPEITYENNAIECRLSIGHTNHHYAALSKCRLVHPYDL

>Sman_CCD75249

MSRTNSDKTSFRNQNTIIIITTIVVDFLIANIQCSSVNYDGDIKLVDGPSEQTGTVLVYRTYGWGKVCDDHWTMKEANVVCRQLGMGHALEVHRRNRFGSSSQANYLMDEVHCTGSEKRLTDCRFNGWGVHDCRINEEAGVKCAQKAVIIDKPLWNPLKLKFTQLELEKMSKIKFKVISQQLNLSHHPQSSIFIPVLIETKNGTKGTVCSDHFHAAEAIVFCHQLNAGRGGRVIAMPIEQTGINTTNPIAILGYCYGNETDLKYCKSYIDYNGISCQSKLAAAVECTNNLPDLSPDRYSLETSAYIQKMSLWLLECALEENCLPDIVYRIIDNNPYNYTWMTRTLMRFSSIIENSGNEVFRPLEDPDNWEWHACHMHYHSMKVFSRYEVVDTEKRLVAVGHKSSFCLEDNLCKSGVAPKFRCSNVVDSKGTQGISPGCRDMYLHDYDCQWVDITDIAPGQYTFQWVLSMLVNLPKKDDREKKELHSLQ

>Cmil_SINCAMP00000003140

MVLDRQTPTYRVHSVSCVGTESHLALCQLAMNKGNTTSRCQGGMPAVVSCSLRGTGRKAKQLPVRLKGGARLGEGRVEVLKGNEWGSVCDDRWNLLSASVLCRQLGFGSAREALTGARLGQGSGPIHLTEVQCSGLERSLADCPAKNISQEDCQHVEDASVRCHTPYLGLENSIRINGGRMGHEGRVEVLTGVDSNGTQVWGLICGEGWGTLEAMVVCRQLGLGFANHGLQIRIVGGRTSREGRVEILLGKKWGIVCSDGWTTKEAMVVCQQLGLGYSLHAVTQFLGWVGGRVCDRKGWREERVSEGVVGEEGERKRDGVCERRVKTASDLILNAGLVQQSAYIEDRPLHMLYCAAEENCLSSSAKNANWPYGHRRLLRFSSQIHNIGRADFRPKASRHSWVWHQCH

>Ocar_g7137_t1

MPCGKLISRLDFASSAARLDALKLSDDGKLCLVTDEALEVLLSSSESPCQLAFRTAAWSPVGCDSVGGSSDCLMRQAADISIVPTEESNSVITLPCCSFACQLQYSGMGQKVKKEKEKIDSILYEYRMDLLASVAISWSPCVSDSQSVLLAIATRNQIPVHVLKWTAATLSADEVLVAVKNSAFLQISYDVEKETASLDEKPDFRVLRGLHGMSVSGVAETDAGIWFTTSLDGRVGTFGLTDDLNSVTLTGNFDCLVSAFHGCAVSPNGLFLYLVAYPNLPTGFKRMNVHMKSQVWNKQPAISPLTEEEKDIVDKIEAICNHVEDFNECGGEASRGVRIVGPTEGAGRVEVLVKGQWGTVCDDGFDLIDAGVVCRLLGFESAIEAVPNSWYGARGPAVIVPAHEXXXXXXXXXXXXSDPKEHEWSESRDFGVRLKGSSKPRTGYVEVGFGGRWGTVCPDGWGSREARVVCGQMGYRKGRPRLYVGSKSWNKSSKKPITTTSTNVTENALMADINCNGLEINLALCSHAGFGRHRCRSPGIAGVLCWRPSKSLGTLKHQLWGDKQVRLRGSALPWEGRVEVFWNNEWHKVCDRGWDVQDASVVCRQLGYGSAYEAVTRSRGFFGQGFTRILMNNVRCKGNEPKLLDCQYDDTFRQSCRHWENAGVRCNPPPVNHIPARLIGGGENDGRVEMLQSTSSRTRWVAVCGDGWNEEAAAVVCRQLRLGFALENGKGGKYGNGVEYLVMNVLCNGDEDSLEHCQQGGVASVLNLADLIPNFKSTEDSLNEHSVSFVRLSELTCAWEENCLSKSAAQYMAEGQHMEEARQFHSRRLLRFSVQVFNFGTRDFLPIVDRKDWIWHSCHRHYHSFENFATYDLINSSSGKSVAEGHKASFCLEDGHCVEGGQKEYLCADNHQGISVNCADTYNFNIDCQWIDISDVSYGNYTLVVTVNPSHLVAEMDYSNNVLKCSMQFYPTAVHQEGTMHKVQCQMG

>Dmel_FBpp0085171

MASFRFQLLQLLVVLSQGWANLNVQNNYRNMMVRLATNKAALAGIQVLREGRVEVSFDFGASWGTICSTSWSMREANVVCRQLGLGYASKASQGTEHGDSRKYPWGMVGTLCRGTERRLADCIRESHYPNLCNARNHNVSIAACVSHSADLEIGLVDIERTARLEAVPMSRLTCAMEEHCVSADAYEIRRTNPHAARILLRFSVKASNVGTADVSPYANYKEWVWHQCHRHYHSMNVFATFDVYDLNYRKVAQGHKASFCLMDSECRPGVRQKYTCGNTTQGISVGCADTYTDVLDCQWVDVTRVPINRRYILRVALNPEYKLGEISFENNGAECLLDYTGVRQTTRIFNCRRKPLWFKI

>Scil_58009

MLALTSSSTCSFLRMISVGCMPTSLSEFNCNFDNRQCRVQWRTGSQPGATWSWLAEQGSTPSRGTGPAVDKTTGTAQGTYMFFEASYPRRQGDRGELMSPMLPAVTNGCRLRFSYHMYGSGMGSLRVLAVHTPGQRPRPQVFSVSGNRGNRWHTATVNLPASEGDFFIIFQAIRGTRFSSDIAIDDVEFVGCTGLPSTSATTAPPGTSSRVRLVGGANAMSGRVEVYHLGEWGTVCDDQWEIRDANVVCRSLGYGLAESAQCCASAGRGSGRIWMDELRCNGQESSLTDCIFNAWGAHDCGHGEDAAVVCSGALPTRPTVDPTAPQADLVFNDAEMLSSMNLYIEQLTAEQVICSAEENCLSVTRADILQASAGGRLSSRHLLRFTTSAWNRGSADFTPPPAEPQWHACHRHYHSFDDYATYDLVDSAGVKRGFGHKASFCLTDSACDPGVQPKYECGLNNQGISINCSDAYGANLDCQWVDMTGLSPGVYWLTVHINPARIAPEERYDNNDATVMFNYTGTTITGKRLVSGPPQGNSNTQGSATPTTQPQPITTQAPSGPDVRLVSRDNNTLSGRVEVRINGVWGTVCDDRWDFNEGNVVCRQLGLGYATDVSCCGQEGNGAGQIWLDELQCAGNEQSLTGCRHSGLGIHNCLHSEDAGVTCSGESHISPVISRTIIFRLESWSNVFA

>Scil_28565

MPTSLSEFNCNFDNRQCRVQWRTGSQPGATWSWLAEQGSTPSRGTGPAVDKTTGTAQGTYMFFEASYPRRQGDRGELMSPMLPAVTNGCRLRFSYHMYGSGMGSLRVLAVHTPGQRPRPQVFSVSGNRGNRWHTATVNLPASEGDFFIIFQAIRGTRFSSDIAIDDVEFVGCTGLPSTSATTAPPGTSSRVRLVGGANAMSGRVEVYHLGEWGTVCDDQWEIRDANVVCRSLGYGLAESAQCCASAGRGSGRIWMDELRCNGQESSLTDCIFNAWGAHDCGHGEDAAVVCSGALPTRPTVDPTAPQADLVFNDAEMLSSMNLYIEQLTAEQVICSAEENCLSVTRADILQASAGGRLSSRHLLRFTTSAWNRGSADFTPPPAEPQWHACHRHYHSFDDYATYDLVDSAGVKRGFGHKASFCLTDSACDPGVQPKYECGLNNQGISINCSDAYGANLDCQWVDMTGLSPGVYWLTVHINPARIAPEERYDNNDATVMFNYTGTTITGKRLVSGPPQGNSNTQGSATPTTQPQPITTQAPSGPDVRLVSRDNNTLSGRVEVRINGVWGTVCDDRWDFNEGNVVCRQLGLGYATDVSCCGQEGNGAGQIWLDELQCAGNEQSLTGCRHSGLGIHNCLHSEDAGVTCSGSGTTPQSTTSRRVRLVGGPNQLSGRVEVFAQNQWGTVCDDHWTLTEAAVVCKQLGLGNPLAAHCCSNHGQGTGSIWLDDVRCTGTEQSLLQCSHPGLGQHNCAHSEDASVACAPASSGSSGSVGQAPLPASTTSESTFTTTTSQSTTGAAAATTTATTDTAVSSSASSVDDVSTESNHVTAPASGCFAPGIPQNGGRTSNPGEVYQVNDTVTYNCESGYSLIGDITRSCQPSGQWTGTLPTCQAS

>Scil_108279

NRGSADFKPPPGDGEYHPCHRHYHSMVEFAEYTLVDSAGLKRGFSHKASFCLRDTACDPGVRQKYKCTSTNQGISVDCSDTYGAGLDCQWVDMTGLPPREYWLTVHINPTRLVPEERYDNNNATVMFFYTGTTIRGKRLVSGPPMGI

>Lcom_155653

TPQELAATRNGARLSPHHLLRFTTAAWNRGNADFRPPPADPQWHACHRHYHSFDDYATYDLVDSRGSMRGFGHKASFCLTDSACDPGVQPKYECGLNNQGISLNCSDAYGANLDCQWVDMTGLAPGQYWLTVHINPGRVAPELSYDNNNATVAFTYNGRTITDKRLVSGPPHNPSSSSLICPACSPNSICSVRMSQCVCNEGF

>Lcom_105194

MRNGARLSPHHLLRFTTAAWNRGNADFRPPPADPQWHACHRHYHSFDDYATYDLVDSRGSMRGFGHKASFCLTDSACDPGVQPKYECGLNNQGISLNCSDAYGANLDCQWVDMTGLAPGQYWLTVHINPGRVAPELSYDNNNATVAFTYNGRTITDKRLVSGPPHNPSSSSLICPACSPNSICSVRMSQCVCNEGF

>Lcom_72990

TPQELAATRNGARLSPHHLLRFTTAAWNRGNADFRPPPADPQWHACHRHYHSFDDYATYDLVDSRGSMRGFGHKASFCLTDSACDPGVQPKYECGLNNQGISLNCSDAYGANLDCQWVDMTGLAPGQYWLTVHINPGRVAPELSYDNNNATVAFTYNGRTITDKRLVSGPPQNPSSSSPTVSVSVSPTTAPVTTSASTPDVRLVSTDNNSLSGRVEVRVNGVWGTVCDDRWDFNEGNVVCNQLGIGYATDISCCARQGQGQGQIWLDELQCRGTESSLFGCRHANIGSHDCSHSEDAGLTCSGAGTTQPTSPANRVRLVGGRDRYSGRVEVFAQNQWGTVCDDHWTLQEAGVVCRQLGFGRAVAAHCCGNHGSGTGSIWLDDVVCTGSEPDLLQCSHPGLGQHNCAHDEDASVVCAPASGGSNGDVGQGPPPTIGTASPSGAPISTTPVATTTASAAASSVGSSTVTTTVTTETAAAPAGCFQPGIPQNGGRNGTMAASDVYAVNAVVLYTCETGYSLVGVATRACLPSGQWTGALPRCEAVTSG

>Lcom_53432

MRNGARLSPHHLLRFTTAAWNRGNADFRPPPADPQWHACHRHYHSFDDYATYDLVDSRGSMRGFGHKASFCLTDSACDPGVQPKYECGLNNQGISLNCSDAYGANLDCQWVDMTGLAPGQYWLTVHINPGRVAPELSYDNNNATVAFTYNGRTITDKRLVSGPPQNPSSSSPTVSVSVSPTTAPVTTSASTPDVRLVSTDNNSLSGRVEVRVNGVWGTVCDDRWDFNEGNVVCNQLGIGYATDISCCARQGQGQGQIWLDELQCRGTESSLFGCRHANIGSHDCSHSEDAGLTCSGAGTTQPTSPANRVRLVGGRDRYSGRVEVFAQNQWGTVCDDHWTLQEAGVVCRQLGFGRAVAAHCCGNHGSGTGSIWLDDVVCTGSEPDLLQCSHPGLGQHNCAHDEDASVVCAPASGGSNGDVGQGPPPTIGTASPSGAPISTTPVATTTASAAASSVGSSTVTTTVTTETAAAPAGCFQPGIPQNGGRNGTMAASDVYAVNAVVLYTCETGYSLVGVATRACLPSGQWTGALPRCEAVTSG

>Lcom_106232

TPQELAATRNGARLSPHHLLRFTTAAWNRGNADFRPPPADPQWHACHRHYHSFDDYATYDLVDSRGSMRGFGHKASFCLKDSACDPGVQPKYECGLNTQVRWKHAVNNVVHAGCE

>Lcom_79040

MRNGARLSPHHLLRFTTAAWNRGNADFRPPPADPQWHACHRHYHSFDDYATYDLVDSRGSMRGFGHKASFCLKDSACDPGVQPKYECGLNTQVRWKHAVNNVVHAGCE

>Lcom_194396

TPQELAATRNGARLSPHHLLRFTTAAWNRGNADFRPPPADPQLHACHNHYHSFDEYATYDLVDSKGSMRGFGHKASFCLMDSACDPGVQPKYECRLNTAGRSRVNALCLVRHITRPRHH

>Lcom_115096

MRNGARLSPHHLLRFTTAAWNRGNADFRPPPADPQLHACHNHYHSFDEYATYDLVDSKGSMRGFGHKASFCLMDSACDPGVQPKYECRLNTAGRSRVNALCLVRHITRPRHH

>Scil_57072

MTPRLEKRHPNYKRAVSRTKPKETSNPKRSPEDTNNSFRVEAERSSDAETNAWVGTMRIMLTAPRPQPHNGFGDDDHDATRCCGGGWSVQIYWGLLKSMFCLLLICFAASRSKAQDANSRGRVCKTRWIPSTSQSTGQALNISSGGSSSNSTDLTARPSYRCCTGWNWNGQDCVVAGFECTECYPCIGGDICDCAGGGRAPICEAATVLCDEASGIGFDRGLFPAFNIVYDHSDWLLAGKKEVERDLDIMDSNPYTPDEQQVAVFDSLLSQVSSNGKSNARLLTVLYMQPAQGCHMSLSVLVACRQPGQQNADMMMMMGSSNSNSNDGRTASQAGAPIAPGYFQVRVVSGHSEEELLYIEAAHPCDGRQQAEWAVVELPINVSTTFQVEFEAYSSESSGLLVALDNITFTEDCCSVDGAVSLAPPPDQMHTPAMPSQAPGLPDVHVDARMLRDSLYVDTRDLTREQILCSISEDCLAPSVHNNTDGPAGVESRSLLRFTTAVWNTGKGDFSSPPGHIPIFHQCHHHFHSFSEFATYSMIDERGAEQGHGHKASFCLLDSICRNGTQPKHIICTETLQSISPGCADVYGADLDCQWVDISNFAVGCYQLTVHVNPSRVIQELDYSNNNASVAFFFNGVNVNITEACQELFAGKRSLPEPSSEASSTSTITSNITSLAMTDGGPANQAISPAIGEQPNATGNAQDENPVVVRPWRSLAATMRPGPSSYLTVLFCVAVGRLAVYVGT

>Lcom_51877

MKAWLRLRQTLVASVYVLLAVGVCLSSCQHAGSSGSVCVSEWQAPGNNVTYGYECCNGWRWDGESCLWAGRQCSGTCYPCVAGSVCSCVGGGLAALCQAATLMCEKENIITFDNGLFPTFNIPLDTSDWLTASRRALSDHLSSVLEVAGGSSDPSEEADKVALFDSRTARSGSVARLLTVLYMQPPPECRILFDLMMADARNTSDSGNSEEEMAGRIDVVLVSGRTRQVVFSTDSTETNSSAGYWSTVQLDINVTALFQLEFEAHHGSNMIVAIDNIEFSEECCSVADDLTFSPPPNQQVSTSPSQFPTLLPDLEVNRNVLVDSLRLDVRNLSTEQILCSISENCLRAPENGTDVVPTVRALLRFTTETWNRGPGEFRAPQRAIPIYHQCHNHYHSFSQFTSYQLLDERGVERGHGHKASFCLFDTVCEVGVTRKNIICTEFSQAISPGCADVYDSKLDCQWVDMTNLSTGCYQLIVTVNPDRAVPETTYDNNGARVAFHYNGYNAVTDAGCAELFLTAAVRSGTSSACYGLPQHTISMLLTMLIVRTLTKELLAV

>Lcom_22308

MQWPISTGLHVVAVVVFVSLLLLREVPGQRLEKPSKLPIRLVGGQNALIGRVEVQYNGTWGTVCDDNFDLNDAHVICHQMGFVRAISIYPSGHYKTADESAPTWIDELQCRGDEKNLLDCSFDLGHHDCSLRHREDVGVRCYVPQGRVYKLETLPVRFRGGGALEGYLQVFYRNRWGLVDASGWSQVNSKVVCGQMGYCGAEETRTVDRTSSHLNFSSAVRGDRPFFWMKGVSCLGGEDQLKECRHFGFKAYYRLPAVKVRCLKSCATYFQNGTLKPTAVRLRGGSLRHEGRLEVKINGIWGTVCGTYFFKEAATIACREMGFGSAVSSLRHTPYGRGSGYLWAQEVTCRGNEGSLLDCDMRLNQRPENEYYTSCRHVDDASIMCRVPRSNQRLRLIGGNNENEGVLQIRHRGQWGILCKSRKGQWTMTEAKIACRDLKLGYPVAHLLQFPKPAVESASRGGITHWAVGMKCSGTEDGLHACAVGGKKSYIGARYCPQGEATVKCSKELPDLVPDMDRLRRTLSERVHRESLFRLRCALEENCLSSSVYSLPYAPHANRYHQRRLLRFSARIMNRGTADFLPSRDRSRWQWHQCHRHYHSFEEFTHYNITNDDGRLMVEGHKASFCLEDVECDGDTSKRYQCSHGTQGIRKNCADVYRFDIDCQWVDVTDLQSGVFTLSLAVNPQKLVAETDHENNVAWCRFAYESQSGAVRVLQCGLSGHN

>Scil_31988

MQVQHNGTWGTICDDNFDLSDGNVICRQLGFRRALRIIVGGKYRTTDPSMPTLLDEVNCSGDEKKLVDCKFTIGQHDCDPYHREDAGVRCYFVRRKRVHVPELLVRLQGGSDDDLEGYLQVFYRNRWGLVSGEEWSQINSKVVCGQMGYCDAEETSVVDETSSYFNFSSVVGGDRPFFWMKGVSCHGGESLLKDCRHLGFNGYYMLPAAKVRCLKSCRTFFQNGTERPQKVRLRGGARNNEGRLEVHVQGRWGTVCGTYFYKETATVACRQLGFGSVKNMFSKTPYGRGIGPVWLQDVNCRGNEGDLLDCDKRVNPRPDNQFYASCRHSRDVSIECRVPRFNTRVRVYGGRTASEGFVHIRHRAQWGGLCKNSLGRWTMLEANVACRDMGLGFAVSHTPYFPPAPPTNQSAGLVTHWIAPLRCTGKEQKLHHCAQGSKNSWIGARFCPQGEATVLCSKQLPDLIPDAFRLKSTLSRTVHRQRLVRLRCAMEENCLSSSASAYNPYSSEAYYVYRKLLRFSARIMNYGTADFMPSNDRRQWQWHSCHRHFHSFEEFTHYNISDEQGKLVVEGHKASFCLEDVECDPDTSRRFHCAHGTQGISVNCADVYRYDIDCQWVDVTDLNSGVFTLTLAVNPLGLVVESDHSNNVAWCQFGYERSTGVVTMIKWGLRDH

>Scil_30388

MFANRLRIHWSLMALLLILFAATVCVGPAHGQEHEETNRIERQRPTKLSVRLVGGPNALEGRVEVQHNGTWGTICDDNFDLSDGNVICRQLGFRRALRIIVGGKYRTTDPSMPTLLDEVNCSGDEKKLVDCKFTIGQHDCDPYHREDAGVRCYFVRRKRVHVPELLVRLQGGSDDDLEGYLQVFYRNRWGLVSGEEWSQINSKVVCGQMGYCDAEETSVVDETSSYFNFSSVVGGDRPFFWMKGVSCHGGESLLKDCRHLGFNGYYMLPAAKVRCLKSCRTFFQNGTERPQKVRLRGGARNNEGRLEVHVQGRWGTVCGTYFYKETATVACRQLGFGSVKNMFSKTPYGRGIGPVWLQDVNCRGNEGDLLDCDKRVNPRPDNQFYASCRHSRDVSIECRVPRFNTRVRVYGGRTASEGFVHIRHRAQWGGLCKNSLGRWTMLEANVACRDMGLGFAVSHTPYFPPAPPTNQSAGLVTHWIAPLRCTGKEQKLHHCAQGSKNSWIGARFCPQGEATVLCSKQLPDLIPDAFRLKSTLSRTVHRQRLVRLRCAMEENCLSSSASAYNPYSSEAYYVYRKLLRFSARIMNYGTADFMPSNDRRQWQWHSCHRHFHSFEEFTHYNISDEQGKLVVEGHKASFCLEDVECDPDTSRRFHCAHGTQGISVNCADVYRYDIDCQWVDVTDLNSGVFTLTLAVNPLGLVVESDHSNNVAWCQFGYERSTGVVTMIKWGLRDH

>Scil_36618

MSRVLLFVVLLALVEVISHVSGGTPPERLRLSISIPATDRCRALGQGYVVINRMDIYGRVCTTGWDLRDATVVCRAMGYTGALPLDTSPEHGCPGSEYRRVTTTHRYWLSNVQCHGNESSLEQCAHDGWGVHDCLPQQFAMVRCTHESGDVSQDTDAATGASSQTPTEQCDSQHISAVRLYQGRRMTRPNMGYLQVRVCNEWKAVCSDDWSANLAGEVACRQLGYPRARYLFTARPDAPDRRRQAWLKRPRCSGNESFLHQCSFAGRLARQRCLRRRVAGVVCQASEWTTQSSYEAEKVATSGGTQDKQAEGSLRLRGGAFPSEGRVEVYHDGMWGVVCDRGWSLREANVVCRQLGFGSATDAPHAASRVFGRGYSPIHLSGIRCTGYERYLVQCRRNGWGERPCNRERIASVRCHVPNTQDLRKNIRLEVLPDEVGGTRTPAIRRRGLVQVRAGDNQWGRVCADDNWDLEAGAVACRQLGYGYVYSVFTVTGCNSSSAAGSKATTTVPPSTVKAEEGAADVASVVDLGSELLLPEQLLGPELSKATAAPPVKENEENADRETAFVSELRCTGQEYGLNQCAYKTYDAKGAECPNCKFAAVECAHALPDLVPDLHELEHSLHDIQYHTLASLECAHEEGCLSSSADAIFEAGLQGTYRRALLRFTVMIRNYGMADFHPVRSRPTWIWHACHSHYHSFDNFATYDLLDGSGQHVAEGHKASFCLEDTACDAEIGRIYYSCAAHRQGISLRCADIYGRDLDCQWIDITGVPDGTYTLRVGTNPNRLAAELDYTNNVAECQITLTSGSYETHYTAGVVSNVTCRGSHLH

>Lcom_23110

MSFWPAPSLLSLVTCCVYLSFVSSMAPEEKLRLRLIAPRLASSQCETSSQRIVGLQRMDLDGRICVTGWDLRDSHVVCRELGFSHALPLDPECVVREPGDGSNRNLYRFWLSNLRCHGNETKLADCRHDGWGMHDCLPQEYAMVRCTHSRGRGDDVLPAQAGNEQQGSCSGDARRISGVRLYPYRLVAKDRGGFVQIRVCDEWKAVCGNDWQASFAGEVICRQLGYPGVQFLFWARPPAADRRREAWLKRPRCNGLESYLHQCPYEGRLQGQRCRLRRAAGVVCRPSKATAATVASGAEVGSGEESVVSTRKARGTVRLRGGAFASEGRVEVFDGHQWGVVCETGWSLREAGVVCRQLGYGSASDVMGQASRVFGRGFSHVLLDDVKCTGRENKLIECRYQGWRITSCNRERIVGVRCHAPDVSSQRQQIRLQVIDDDVRSPSYRRRGMIEVLDDGKWGPVCADENWNLEAGAVACRQLGYGYAYGVFTTSQGCTTGSEDGKPPSSKTNFGVVEGIENVVDAGFSIIRPAESGPPADSEVKSKTESSLATAVGLRCAGTEFSLRQCQHAVYAASGSACTNCKYAAIECAHALPDLVPDHQVLEESLYDIEYRTLRSLQCAYEEGCLSSSADAVMESGTAGTSRRALLRFTVTMRNLGLRDFLPVRERDTWVWHSCHHHFHSYDNFATYEILDLAGNHVAEGHKASFCLEDTDCHSISGGRVFYRCIEHRQGISLRCADVYDRGLDCQWIDITDLPNGVYILRISTNNDRLAAELDYGNNVAECQMTLTHGSYERFYNTGTVTNVTCTAEHLQ

>Aque_XP_003387024

MLCFQFLFFLILSSLVVNGQKPGDVRLVGSTLPFEGRLEVFFFGAWGTVCDDRFTIVDGAVACRQLGYNDGAERTETTGLASSRFSPGTGDILMDGLECKGDEKKLGDCPFNGWGLNDCVHYEDVAVICKFSKPSLPKPNVTSVRIACPESDYGLGTCINTCSLPSTTCNNETEVAQIGIVERLVNNSWYPIPSSDWDQASAQVVCNQLGYPRAGPIPSIDEIFPRRRCGRPRNAARCAAIRNFNDRLSKTVTEGLACIGSETSIDDCIITSYAVKQSSALNVATIQCLHDSTRIADCENYNYTNSSRELYRLRGGPVPWKGRVEVKVGGVWGSICDLSWDVKDANVVCRNLGFGYAEAVFTRSQFGRSSATTHFSGLRCNGNEDILSDCSSNTGGLLRRDAHSYCVFHSGDVGVECGMPACNKEPEVRFATTGSETMIEVNGPLGWGRLCFDNKLKYTEAHVLCRDISGELAASFGPITDLSYLGLSYSAQYNCTGDELHSSECRESITRTFCFDYTSIECAQGLPDLVPMVDQFERSLRSYPYVHLIPMYYLTCALEEQCLSSSAYRHADNLYHIRRLLRFDSLTMNYGTSQFLPSLPSSEWEWHTCHQHYHSFEAFISYDVLNKDGEKVAEGHKASFCLEDSTCRGLGAYMYYRCSTGNQGISKNCGDLYGSHLDCQWIDITDLSPGEYIVRQIVNEEAGVGESDYRNNVIQCEVEYMSNLYQLKVGKCSHSDHI

>Aque_XP_003387025

MLPLGSNTLNSGLLLLKAVAFCLVIQCVKTQDRPSDGTIRLQGTLPYQGRLEVYYRGSWGTVCDDRFQKPDADVVCRELGFSGGSDRVITTGFSNEFASGTGEIWMDGVSCRGSEEKLSQCSFNGWGIHDCRHYEDVAIICKYTAPTLPPSTSLPTLSDNNVRVTCPGPNYRLGECNNCSTYTSACRAPDPRRPAIIGVVEMLIDGKWYPIPRKNWNVNAAKVVCGQLGYPRAGPSPNINRIFPTKSCSNSHTSAQCDSINDFNDRLSRTVTEGLTCTGGENKLNDCYFQSYNINPSSPASVATVQCYFDDLRTEKCKDRNQNNKELYRLRGGPVPWKGRVEVKVGGVWGSICDLFWDLNDADIVCRNLGFGTAKKVETRSFYGRASDSVHFSGLKCTGTEQMLSQCTRTTGAQLPREINSYCYQHSGDAAVECNIPQFCPKEKEVRFTGTGVDKFTEVKGPSGWGRLCFSSISTYNEAHVMCREVSDELAARYIPIKDPQYRGRSYVVEFKCTGDEISTSECRRTFRTSSCINYTRIDCTAGMPDLVPNLKKFQSSLESFAYTSPTPMFYLSCALEENCLSSSAHQHANNPSHIRRLLRFDSLTMNYGTATFLPNLEPHEWEWHACHQHYHSFEAFIHYDVLNRAGEKVAEGHKASFCLEDSMCDFGGYSSYRCSTGRQGISVNCGDLYARHLDCQWIDITDLPINRNYIVRQIVNADNLVGESDSKNNVIQCEIFFRGDSFPFIVSNCTHSDH

>Ocar_g7139_t1

MRGIKCIGNESSLMECHLPPPKHDPSGCKHFMDSSVECYKPETSNKIVRLSSFSRETDAIPAKHGFVEVRKNVDGPWFPICDVSWGHKEAQVVCRQLELGYAEAARQRPYNKELVKYFQADYTCEGNEVDLLELPDLLPDVEALRFSLRQQVIAVRLSSYQCAIEEKCFSASAYNRTDDPLRYLIRFTTRIMNLGRADFEPHANRDEWHWHQCHNHYHSFEAFSFYEVINSAGVRQVAGHKASFCLEDSDCLENGPPPRYYYYCSTGHAGRGGHQGISANCADSYFSHLDCQWVDVTGLAHGNYRLVVAVNPHRKVAEMDFENNAVFCEFTYSGHGFLKLGECGFMPDFEQLF

>Bflo_125511

MPSQCMKVFQFTGVTSFVVFCMLYGVYQYQYFYNGNYYYPYNSYQSSSSSSSSYAQQYAQYLAQNFGSHSQYASQYGSSSYGSSRVQPGSQPQYAYQQYSTGGRQAGQRTYSRRSPAYGYARRTTGRRSNSLRRSGLPDLVPDAALVQTTAYTENVPLYRLQCAKSEKCLSSSAYSRTATQDSVRRLLRFSQRTTNEGDADFLPDSDPSQWEWHSCHQHHHSMETFAKYDLLDASSGQQVAEGHKASFCLEDTGCRRTYRKYNCALGRQ

>Bflo_126229

MPSQCMKVFQFTGVTSFVVFCMLYGVYQYQYFYNGNYYYPYNSYQSSSSSSSSSYAQQYAQYLAQNFGSHSQYASQYGSSSYGSSRVQPGSQPQYAYQQYSTGGRQAGQRTYSRRSPAYGYARRTTGRRSNSLRRSGLPDLVPDAALVQTTAYTENVPLYRLQCAKSEKCLSSSAYSRTATQDSVRRLLRFSQRTTNEGDADFLPDSDPSQWEWHSCHQHFWNDKLNLQQQRGVCVVLGCSRKTKIIIVEDAKTTRQIITDIQVAKVGKSVS

>Pgem_c36618_fr5

LLLLLSFIIYLLIYYLYYLLFVYFNMKSILFISSAFVFAADGYNSVPNCYHNDGGFQYAGNNNVAFSGKECLAWDDFSLHNTANRQNFLPELWPDEDLNGEVCRNPDKRPGGPWCFTKHKRKEWETCDVAPCKCDKAAIGNGVCDFVNNHKDCGYDGGDCCLSTCHCKDLGTCKGNCDCSNLHLEECGRFQEGNCRDPSVMSPESHRYIISAQTGHLRWADHKGRIKLTMFFADGSSQYTYLAGDEFKRGEVSQTELILPPVPIIKVNVAVENINDGWYLQSGINIEAEKTNRGYIFMYDGWLNEHNPSATIPRAPHNYASYTVIFHTGWAFLGESQQDLYVEVEGEGPAIGTAGIRAPKVLLGGGYEQGQEVRIHFFTVDVGDVSHLDIYGTNSEDRVYLTGGMDVIKRGVVAKMPGSGWIPKAQDEDSEACLVNNEEVPNRYITGSYSISNFEVEVTTGDHFGSGRRGGVAVTFLSHTDYPTREAYLGESFAPDQTIKTLAMVQNQGIFQAKLTLKDDFFDDVFIKQLTLKKTDKDEIKTFIFNSWLNATHPEIIVDSTNRATEAYNDELPDLEFDQYQFFINFRYKEIHVENDKLECALEEGCLYQQGEVVHGVKMDNNRRILNFETTTWNVGDVTFYPPPQQEAVYHECHKHYHGMRGYATYHLTTAGSWQNDAIILTGHKASHCVMDSTCARSGKEVRHKCVNQGIQPGCADIYNAGLDCQWIDITPLKSGIYSLNVFINRDGLVPEKSFDNNAGHILFKYDTSQTEVHKKIVWACINNDEHATECPPNSPKFDQLRTGIDRYGKVDLEPGSRRWPVRENYNDGDRWDREYEIVTINLDYNKYIYLCTFFAFKDYIYIWVFIVFLFYVNKCDYPYIYVHHFRNIK

>Awhi_c74974_fr6

RLPPDTAMYTVRVRPGFAYLGASDGDLYIEITGEGPQLGIAGETTERLLLSGGFETNVEATTKVFAKNIGDISHADLYGSNSEDRVFLNGGIEVSKTSQVVFMPGSKWIPELLADEGATCLINNEADPTRYIAGSYNVEPFHVILTIGDDVGAGRKGQINVVFKSHSDYPTRVASLGQDFERGATMTFPIFVQNQGTYQCEISLEGNTSDDVFIKTLVLHRPTSNFRKEFYFEQWLNTANPIAVTDSHDRDEASDDGLADLEFDTYQFFQNFRFKEVDIENDKLECALEEGCLYQMNEIVKGEKMGNIRALLNFETTTWNVGDVTFFAPPTQTAVYHECHKHYHGMQGYASYHITNVGSWQKEAIVLTGHKASHCVMDSTCARSGRERLHKCNNQGIQPGCADIYNAKLDCQWIDITPLKEGVYNLNVYINVEASVPEKSYDNNEGHILFKYEPHHHQAHHKIAWACLSTDESATECPPDAPKFNQLRLGITKEGEVDPEPGSRRWPLRENYNDGDRWNKEKGSKRIRRKTKNQKENFSLSLYFSIVALCLHLLGSKDSFLGAKKKKKK

>Sarc_SARC_04703

MRTLILALAVAASISNVLGQIFEEERFTCRTELMHNGACDHHNNVLECGFDGGDCCLATCDCTSNEEYVCLPGCHCNNLDSAACGSPVWTEADCKDPNIGKPNAEHLVAIWTGNGYNHQRETQIRITFNFLDGKKIQVTGGWDTSAPGRQSERSLNLEHKAIESVTVTLLDDYTDGWFMHSGVRITLPESGRVMYFPYQGWLNSDLPEVEIGVGPSDMVDYQITLWTGNNYLGDRREPLYLILYGEGPEIGILGENTTRIELNGYFSLSSKLSFTVFANEVGDVSHLDIIAPGEDAWFMQAGILITHHSVEFGVRSKYMPANGWVSSSTNSADDCTVSADGEGVKRVVSGSGNIVEYLVDIALSDAADAGREGPVSIQFYDKTDYYSRAAELGSRFPKDGHVKVTVFAQKIQVAKIRLQFGEESDDPMLVDSITVTRPDLPLQDPIQFTIGEWMTGGESDQTFTRVDFEQIPSDIEMADLDFDTIQFLNNFRAYEVDIQGDDLECALEESCLLPIGERVMGETMGDERIIIRFETTTWNVGNDVFYPPPEGEFVWHACHNHYHGMTGYATYYITTAGSFSNIILRGHKASHCVMDSTCERSGNDMQHRCTNQGIAVGCADTYSRGLDCQWVDVTPLPAGWYVMNVNINLDKRVREYSYFNNDGHILFQWDPSQPKNDMIVQACLIIDESLTECPPDTPQFDQMRPGTDGGNGGTIEPEKRFYADVDNGRW

>Cfra_g5071

VIPPLISNMSDISCEPTVTNTVHEVAVDTVPSVDTHFTEPAVSASKPSIELDALNHVAHADATDSQEPVISAPQTENTDEAEDIHVSATSTENVTNVENQSNECLGTESSSSEDGDWHPGMGSDIEDIEPSSESGESDNEEEEAAMDIDALEARYMNMCRSSKIPSAEEVMTPEQYREQLGERGNVDAHRVWRANNKLKDTDLTLLHEKVVAQLCGGEYGFEMRTPKDNEFTTALKLFTQCNCDLEQLSERLMESTVKSPTWLEVDILAFERELQLYGKDFERFKVEAKSYDDVINFYFQWMQSPRSADFVALHPESWKRFDVGPIHNCNLCVRPWKGDPTRVVEAILNRRKNGTRWEYEIFEEEKFKCIDALLHNGACDLHNNVKECGYDAGDCCLSTCDCVSHSSYTCLPGCDCNNLSSEMCSEPVWTEAMCKDPNVGKHDAEHVVSIWTGNGRNHERNGPIQLTFHYFDGSSGTETGFDTSSPGKQNDRRLYLAHKAIVAVTVSLVGDLNDGWFMHSGVRISIPDSGRTEYFPFQGWINKDNPKQQISVGDKVLSDYTVTLWTGNNYLGDREDPMYLIFYGEGPEIGILGLESEKVEINGYFALNSKLTFTVFATEVGDVSHLDIIAPGEDAWFMQAGVHISHQSVEFGLREKYMPENGWVSSSANNEAECEISADGEGIKRVVSGSGNIVEYIVDVEVDDAPDAGREGDVTIQFLDKTDYYSRTATLGSHFPKRGKIRITVFAQKIQVAKIKLKLDESFLDDPMLLRRVTVIRPDLPLQDPIVFDLNDEWMTSSRNTRTFTRVDFDTVPSDIDMADLDFDTVQFLNNFRAYDVEIDDDDLECALEENCLLPMNERVMGMTMGNRRSIIRFETTTWNVGNEVFYPPPESQFVWHDCHNHYHGMTGYATYYITTAGSFSNIILRGHKASHCVMDSTCARSGNDQEHRCTNQGIAVGCADTYSRRLDCQWVDVTPLPAGWYTMNVIINLDKRVREYSYFNNGGHILFLWDPSQGTNDKIVEACLVINEDLTECPPGTKPFDQMRKGTDGGRGGEIFPEKRFYTAQDNGRW

>Sarc_SARC_00003

MRIPSLLVAVLAVASSTLVQAHQHEGEVEADAEADILVPLNLTNTLSDESDELVEYKLTFYTGNGPEDGTKGPVRVMIQGEQVTFAEHDDDTLPHQTIEGEFLPGTSKTVIFKSLPLPEHAAWIDIWNDNKDGDDWYLLAGVKLEYKGISRVFRYEGPVHAWKAMKRASAVGGNIVPHILTITTGDAIDAELEGGDLYGKLLSDGSYESSTQMISHGVPLQRNQTIKTYMMAPEDATDALNIWFNGSNTDMWLLQTVTVQQEPTTEVPEPPITTIDFKWWLQNKIPILMHRDFDDDALSLQPNEEEGVEGRLRLPDLQIESDNMLRNLVLSAGHIWNDHRCAAEEGCFLPMGSKVNGVIMGSWRRFLRFSATYWNYGDADFFPNPDDNPEWHECHNHYHALVDFAKYTITKAGSAGNQVELASAKQSHCAVDSICEDSDDYNYKCANQGITKNCADQYGEWTDCQWIDITPLKSGWYVLNAYVNMNRRVRESDYSNNGAHVLFRFNADGGIDDRGEIDRACVLEDWEWTECPGGEDASYDRSNRCDSSSSCLQTGHLGSKSRWAD

>Cfra_g3850

MLRLYKSKKDLRRRKGEKDTKSDTGEGYDSDDDGAVLASKKEKIFKDAVVGPIVYTLYYPQDHCLLISSIVNCVIYRLSEFCTREITFCKCSHRFHTTRVLDTTNYLHIHVVVVDPPAAEVVSSAESKNEGPVGQKGYVSEVESNRLIHVLVVEEMNFFSLLVITVVIISSSGVLADHEHGHIHVTGVDTQPESPADDLVPMDDSSGMNDEEQAEYKLTFYTGNGPDDGTEGPIRVMIQGEQIEFGGHDDTSVPHQTITGNFEPGEPRTVTFTSKKLPEHAAWIDIWNDNINNDDWYLFGGVKLEYRGVSRVFRFEGPVHAQKARQRASAVGGNIVPHVIAIATGGEKDAELDGGEVYATVASDARLESATQLVSHGRSLAKNSILKSYIMAPYDAHDAITIYFVGDKTDLWLLHEVTIQREPTKEEHQPPITPITFRWWLQNNRPVLMHREFDEDALSLSENEANDFAARASLPDLTIDSQNMLDNLVLSSAYIWDDFKCAAEEGCFLPMGSKINGKTMGAKRRFLRFSATYWNNGDAEFYPNPEDDPEWHECHNHYHALKDFAKYTITKVGSGGNRVEVVAAKQSHCAVDSVCYDEDDYHFKCSNQGISRGCADQYGEWLDCQWIDITPLNSGWYILNAYLNLNRRVKEMSYKNNDAHVLFRYNAGGGFNDRGEIDRACILGDWNWLECPGGTDAEYVRSNRCDSNKSCLQRGHKGWKSRWAD

>Awhi_c78382_fr2

ICDWLQNVPQCDYDGGDCCMESCKLEEHSHTHDDSRDVNELCVCGESLKLPQCGEWLPIDCQDPNFSEPREEVLEYKITLHTGNTHSMDYTGSLEISLVGEPRHFCLENKIAKSTLFDFTEPLHPSSTVVINPKSDNVERVSYVKLRNMDPEKTFVLQAGMIIDYNNEVFYYAGNGILKPNGENTVMVKPGGGDLVWYNITIQFGDEADAGFEDGTLSIQMEDLSGRKTVLRPLLYGPVKPSSTFTVPILAQAMVPAEIELILKTNDETDSYHVLDVEVKLVDTQKKLLGDFHTAQYDAYDWLEPNVLEEIDLNGYRETLGEDTTEYATTAPWEVTKSQEVDIDLGPDYRRAVDRNSQHIFKVHKYKTNRYPDMEPDAIQMLRNMVYTVVDIDDDDLKCATEECLLPKGSYLNGEKVPDVRKLLRFETTVWNIGKVDMYPPPNNDPEWHSCHNHWHALIGFTRYTVTPAGTYDGSLLKLVKNSHCAVDSYCNRGESDKKFKCVNQGISKGCADRYGEWLDCQWVELTTLETGWYNLNVNVNSDRTLPEMDYSNNEVHVMFYFDSEGDVDDTIRYICFSEDEDQMKCPSKTKKFKNKRGDCYTDADCIRDGHKNPRSTYYKKG
